# Reverse transcription as key step in RNA *in vitro* evolution with unnatural base pairs†

**DOI:** 10.1039/d4cb00084f

**Published:** 2024-04-23

**Authors:** Eva S. Hoffmann, Mareike C. De Pascali, Lukas Neu, Christof Domnick, Alice Soldà, Stephanie Kath-Schorr

**Affiliations:** a Institute of Organic Chemistry, Department of Chemistry, University of Cologne Greinstrasse 4 50939 Cologne Germany skathsch@uni-koeln.de; b Technical University of Munich, TUM School of Natural Sciences, Department of Bioscience Garching 85748 Germany; c Dynamic Biosensors GmbH Perchtinger Str. 8/10 Munich 81379 Germany

## Abstract

Unnatural base pairs (UBPs) augment the chemical diversity of artificial nucleic acids and can thus enable the generation of new aptamers and catalytic nucleic acids by *in vitro* selection. However, owing to a lack of methodologies, the reverse transcription of UBPs, a key step in RNA aptamer selection, has not been sufficiently characterized. Here, we present a series of versatile assays to investigate the reverse transcription of the TPT3:NaM base pair as a representative for hydrophobic unnatural base pairs. We determine the fidelity and retention of the UBP for four different reverse transcriptases (RT) in the context of RNA *in vitro* evolution. The retention of the TPT3:NaM pair during the RNA *in vitro* selection process was investigated using a novel click-chemistry based electromobility shift assay. Real-time monitoring of reverse transcription kinetics revealed considerable differences in polymerase activity processing the TPT3:NaM base pair. Our findings identified SuperScript IV RT as the most efficient RT for processing the TPT3:NaM pair. Our approach can be applied universally to study newly developed UBPs, not only at the reverse transcription level, but also during PCR and *in vitro* transcription.

## Abstract 2

Unnatural base pairs (UBPs) augment the chemical diversity of artificial nucleic acids and can enable the generation of new aptamers and catalytic nucleic acids by *in vitro* selection. Reverse transcription of UBPs as key step during RNA *in vitro* selection is investigated.

## Graphical abstract

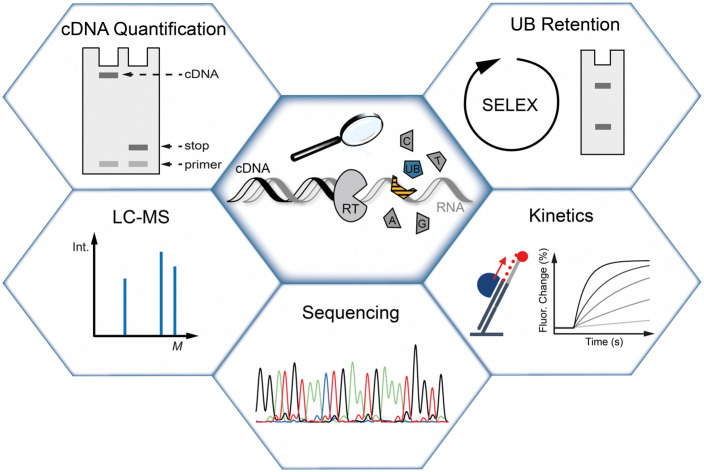

## Introduction

Expansion of the genetic alphabet with artificial base pairs enhances the storage potential of genetic information. In addition, extra base pairs allow the introduction of new functionalities at desired positions into nucleic acids by enzymatic means.^1^ Over the last quarter century, several research groups have developed unnatural base pairs (UBPs) to expand the genetic alphabet. Today, some of these UBPs can be incorporated site-specifically into DNA and RNA by polymerases with near natural-like efficiency and fidelity.^1,2^ The polymerases’ excellent recognition and acceptance of UBPs in sense of a third base pair enables its application to generate new highly functionalized artificial nucleic acids, such as aptamers.^2,3^

Aptamers, short single-stranded DNA or RNA oligonucleotides that bind with high affinity to a specific target,^4,5^ are evolved by *in vitro* selection (SELEX – Systematic Evolution of Ligands by Exponential Enrichment) using a library of up to 10^15^ chemically synthesized DNA sequences.^5,6^ Compared to proteins, aptamers possess a low chemical diversity due to the similarity of the four canonical nucleobases. Thus, in various cases, *in vitro* selection does not yield aptamers with binding affinities in the nanomolar range. To increase the chemical diversity in aptamers, different approaches were developed based on modified canonical bases bearing various side chains.^7,8^ By applying UBPs for the selection of aptamers, specific positions of the aptamer can selectively be modified to generate new possible interaction sites.^9^

*In vitro* selection of DNA aptamers based on an expanded genetic alphabet employing an UBP were first reported by the groups of Hirao and Benner.^10,11^ Hirao and coworkers applied their hydrophobic Ds:Px base pair^12^ to select DNA aptamers against various targets, such as vascular endothelial cell growth factor-165 and cancer cells.^10,13^ They demonstrated that the application of an expanded genetic alphabet to SELEX results in DNA aptamers with increased affinity as well as high specificity to their target.^10,13–17^

While genetic alphabet expansion has been successfully applied to select DNA aptamers, the transfer of UBPs to RNA-based SELEX has not been reported so far. In contrast to the generation of DNA aptamers, further steps of transcription and reverse transcription are required for the evolution of RNA aptamers as well as ribozymes, catalytically active RNA molecules.^6,18^ Compared to DNA, RNA oligonucleotides can form more complex and diverse three-dimensional structures.^19,20^ An expanded genetic alphabet can potentially further enrich the variety of folds, in addition to increasing the range of interactions between the RNA aptamer and its target. Moreover, the application of UBPs to RNA-based SELEX could dramatically enlarge the range of reactions that can be catalysed by ribozymes. However, excellent processing of the UBP during PCR, transcription, and reverse transcription is a prerequisite for the successful generation of RNA aptamers with an expanded genetic alphabet.

Romesberg's well-studied hydrophobic UBP TPT3:NaM presents a promising candidate for the selection of RNA aptamers as it is replicated and transcribed with near natural like efficiency and fidelity by OneTaq DNA Polymerase and T7 RNA polymerase respectively.^21–26^ In previous works, we showed that modified TPT3 ribonucleoside triphosphates (rTPT3 TP) are also tolerated as substrate with high efficiency and fidelity for RNA synthesis opposite NaM deoxyribonucleoside (dNaM) in DNA templates. We further demonstrated efficient post-transcriptional functionalization of RNA by click chemistry *via* a cyclopropene-modified rTPT3.^24,27,28^ Application of the TPT3:NaM pair to RNA SELEX will expand the chemical diversity of novel RNA aptamers and catalysts not only by hydrophobic nucleobases, but also by a plethora of artificial side chains adopting the Click-SELEX approach for UBPs.^29^ We and Romesberg's group recently reported initial experiments towards reverse transcription of the TPT3:NaM base pair.^23,25^ However, the complex reverse transcription process – a key step of RNA SELEX – with an expanded genetic alphabet has not been characterized until now.

**Fig. 1:**
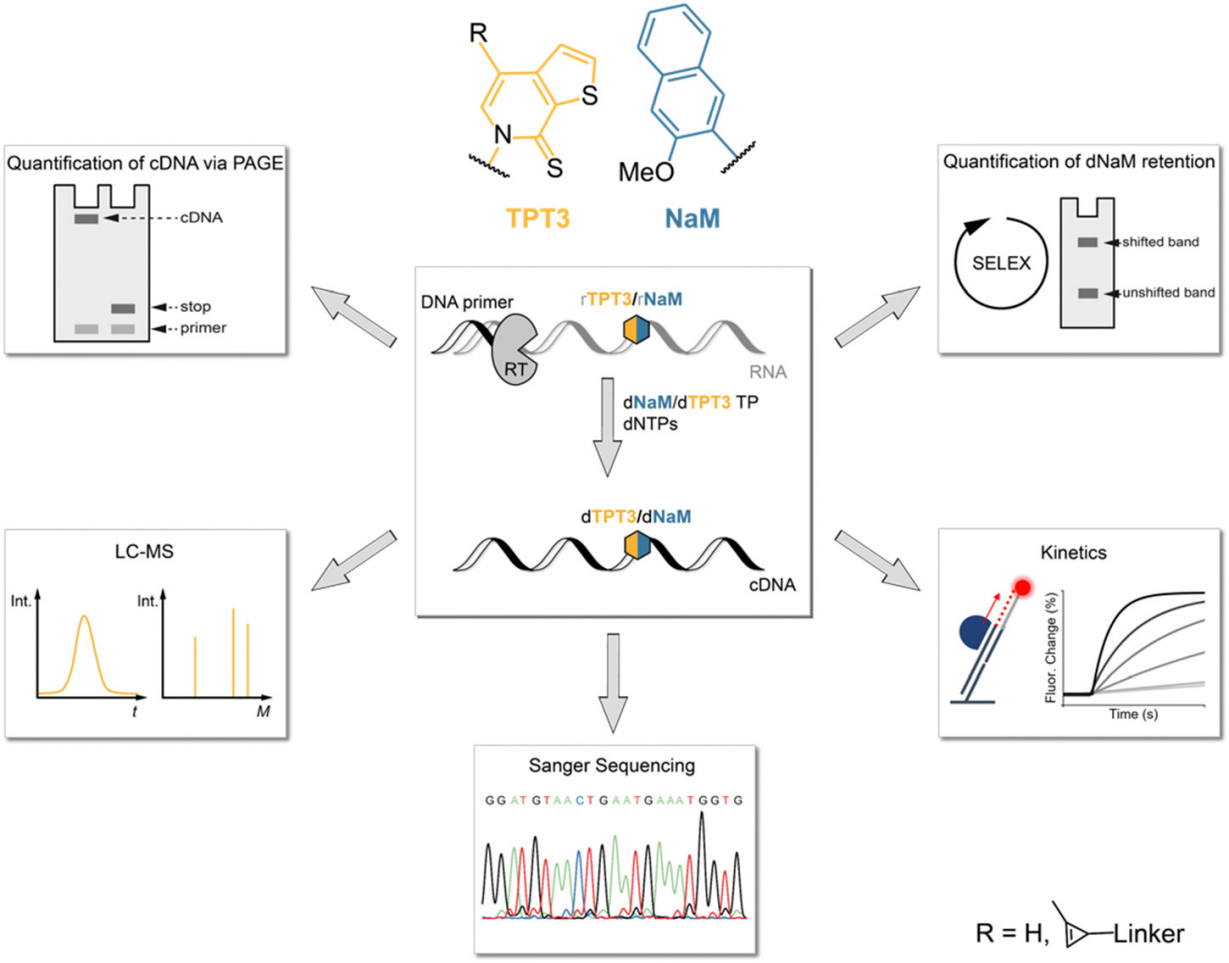
Overview of the experimental workflow evaluating the reverse transcription of the TPT3:NaM base pair. RNA bearing the unnatural nucleotide rTPT3 or rNaM was subjected to reverse transcription with different reverse transcriptases. Processivity of reverse transcriptions were analyzed with different approaches (see panels, anticlockwise): detection of full-length to truncated cDNA ratio *via* PAGE, UB incorporation into cDNA by LC-MS, Sanger Sequencing, kinetic measurements using switchSENSE® technology and a simulated SELEX cycle to explore UB retention in dependence of cycle number.

In this study, we therefore investigated the reverse transcription process of the TPT3:NaM base pair using the well-characterized reverse transcriptases (RT) AMV RT, MMLV RT and its engineered variants SuperScript III (SS III) and SuperScript IV (SS IV) RT, as initial experiments identified these RTs as potential candidates for efficient reverse transcription of TPT3:NaM.^23,25^ For this, we developed different methods to determine the incorporation of the TPT3:NaM base pair and the fidelity of reverse transcriptases (Fig. 1). We quantified the ratio of full-length products and subjected full-length cDNA to LC-MS, Sanger Sequencing and real-time monitoring enabling the determination of UB incorporation and mismatches, RT's fidelity as well as binding and elongation kinetics, respectively. To provide a depth study to investigate the UBP requirements in RNA SELEX, we developed two different approaches to follow UB retention over the number of cycles performed. Here, we also report a mobility shift assay applying inverse electron-demand Diels–Alder (iEDDA) cycloaddition for the first time to measure UB retention. Our approach can generally be exploited to probe novel UBPs in reverse transcription reactions as well as to test novel engineered polymerases.

## Results

### Preparation of RNA oligonucleotides

To prepare short 24-nt and 48-nt RNA oligonucleotides containing rTPT3 and rNaM by solid-phase synthesis, the corresponding phosphoramidite building blocks were synthesized. rTPT3-phosphoramidite was synthesized according to literature (for procedure see ESI,† chapter 9).^30,31^ While in contrast to *N*-glycosidic nucleosides, *i.e.* the rTPT3 nucleoside, the synthesis of C-glycosidic rNaM and its derivatives is more challenging. Literature procedures often use the expensive d(+)-ribono-1,4-lactone as the starting material and harmful chemicals like lithium-reagents and BF_3_⋅Et_2_O for the coupling reaction and yields of only 15–22% are common.^32^ We therefore adapted a synthetic strategy published by Li and co-workers^33^ using a Ni-catalysed reaction allowing albeit still moderate yields cheaper and safer access to rNaM even in multiple gram scale (see ESI,† chapter 9, p. 55).

Detailed information about the conversion to phosphoramidites and the following solid-support oligonucleotide synthesis can be found in ESI,† Section 9.^34,35^

Longer RNA oligonucleotides, 80-nt TPT3-RNA and SPINACH2 RNA, were enzymatically synthesized by T7 *in vitro* transcription (see ESI,† Section 1). All oligonucleotides used in this study can be found in ESI,† Tables S10–S13. An overview of natural and UB-modified RNA sequences is presented in Table 1.

**Table 1:** RNA Sequences used in this study^a^

Name	Sequence (5′-3′)
48-nt RNA	AAG UAU CGU AUC GCC CGA GUA UAC AGG GAA UCC CGA GUA GUG GGA CAG
48-nt 1xrTPT3-RNA	AAG UAU CGU AUC GCC CGA GUA UA**Y** AGG GAA UCC CGA GUA GUG GGA CAG
48-nt 1xrNaM-RNA	AAG UAU CGU AUC GCC CGA GUA UA**X** AGG GAA UCC CGA GUA GUG GGA CAG
48-nt 2xrTPT3-RNA	AAG UAU CGU AUC GCC CGA GUA U**YY** AGG GAA UCC CGA GUA GUG GGA CAG
48-nt 2xrNaM-RNA	AAG UAU CGU AUC GCC CGA GUA U**XX** AGG GAA UCC CGA GUA GUG GGA CAG
24-nt RNA	AGG GAA UCC CGA GUA GUG GGA CAG
80-nt RNA	GG GUU AAC UUU AAG AAG GAG AAA AAC AUG UAC AAA AAG UAC AAG AUG GAC UAC AAG GAC GAC GAC GAC AAG UAA GCU UCG
80-nt rTPT3-RNA	GG GUU AAC UUU AAG AAG GAG AAA AAC AUG UAC AAA AAG UAC AAG A**Y**G GAC UAC AAG GAC GAC GAC GAC AAG UAA GCU UCG
SPINACH2 RNA	GG GAU GUA ACU GAA UGA AAU GGU GAA GGA CGG GUC CAG UAG GCU GCU UCG GCA GCC UAC UUG UUG AGU AGA GUG UGA GCU CCG UAA CUA GUU ACA UC
rTPT3-SPINACH2 RNA	GG GAU GUA ACU GAA UGA AAU GGU GAA GGA CGG GUC CAG U**Y**G GCU GCU UCG GCA GCC UAC UUG UUG AGU AGA GUG UGA GCU CCG UAA CUA GUU ACA UC
rTPT3^CP^-SPINACH2 RNA	GG GAU GUA ACU GAA UGA AAU GGU GAA GGA CGG GUC CAG U**Y**^CP^G GCU GCU UCG GCA GCC UAC UUG UUG AGU AGA GUG UGA GCU CCG UAA CUA GUU ACA UC

a X = NaM Y = TPT3.

### Quantification of full-length cDNA

To quantify full-length cDNA products of reverse transcriptions with the TPT3:NaM base pair, we employed a 5′-6-carboxyfluorescein labeled primer enabling sensitive single nucleotide detection of cDNA fragments by PAGE.^25^ First, we examined the correlation between RNA concentration and full-length to stop fragment ratio in the presence of dNaM TP in reverse transcription reactions using an enzymatic prepared 80-nt rTPT3-RNA (ESI,† Fig. S1). All RNA concentrations in the investigated range of 1.25 ng μL^−1^ to 125 ng μL^−1^ resulted in similar full-length cDNA ratios for MMLV and SS IV RT showing that the overall efficiency of the reverse transcription reaction is not dependent on RNA template concentration.

**Fig. 2:**
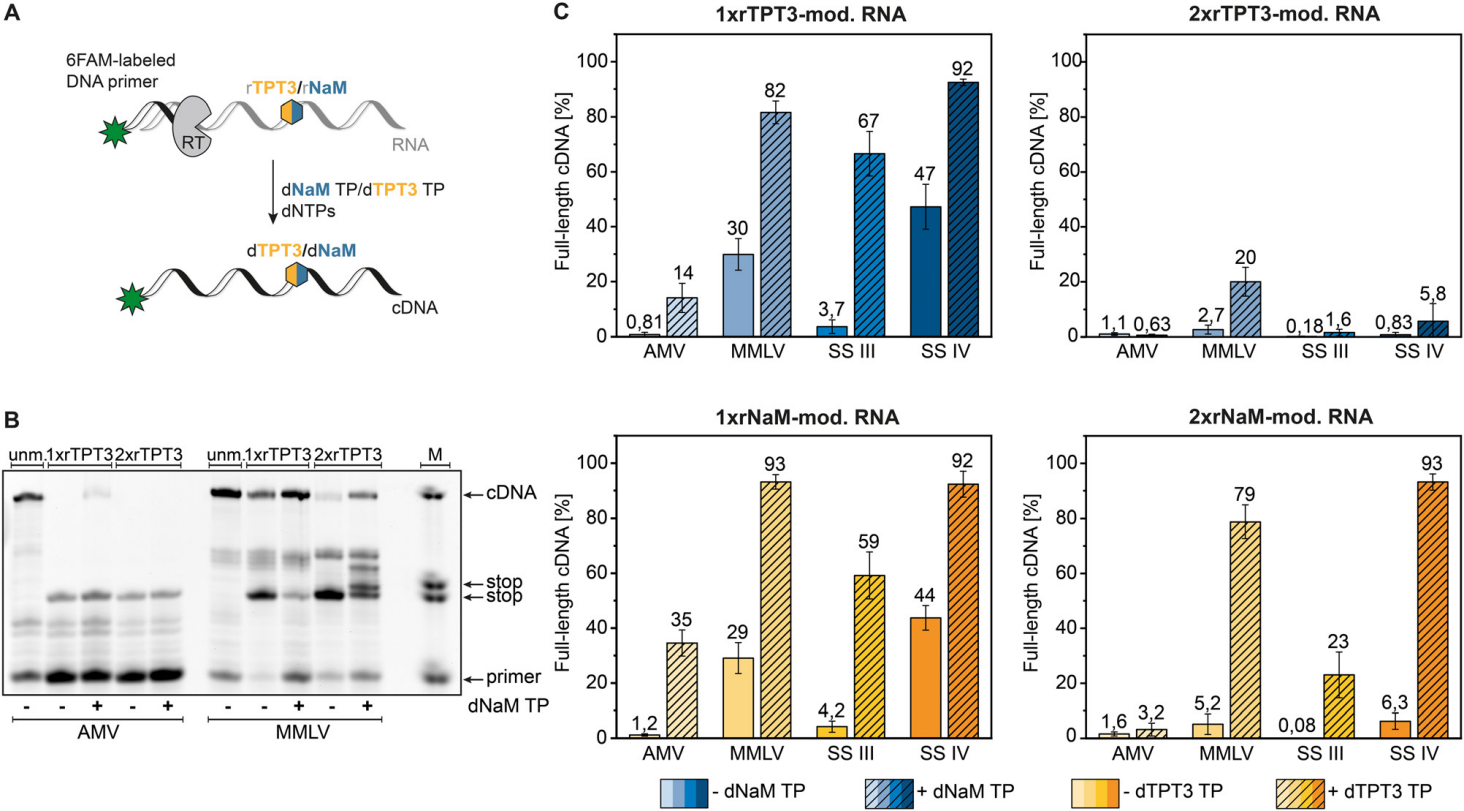
Analysis of full-length cDNA fragments. (A) Schematic representation of reverse transcription with the TPT3:NaM pair using a fluorescently labelled DNA primer for analysis *via* PAGE. (B) Polyacrylamide gel of reverse transcription reactions of unmodified, 48-nt-1x and 2xrTPT3-RNA with AMV RT and MMLV RT. M = marker containing DNA templates with the length of primer, stop events, and full-length cDNA. (C) Percentage of full-length cDNA products obtained for AMV, MMLV, SS III and SS IV RT of reverse transcriptions of 48-nt-1xrTPT3/rNaM-RNA and 48-nt-2xrTPT3/rNaM-RNA in absence or presence of the cognate deoxy UB TP.

AMV, MMLV and its engineered variants SS III and SS IV RT were subjected to reverse transcription of the chemical synthesized 48-nt RNA bearing a single or double rTPT3/rNaM modification at sequence position 25 and 26 respectively (see Table 1) in presence and absence of the cognate dUB TP (Fig. 2(A)). An exemplary fluorescent PAGE analysis of reverse transcriptions of rTPT3-modified RNA with AMV and MMLV RT is presented in Fig. 2(B) (raw data is provided in ESI,† Fig. S17–S72). While we did not visually observe full-length cDNA products for AMV RT of 1xrTPT3/rNaM-RNA without the addition of UB TP, we detected up to 1% fluorescence intensity corresponding to full-length cDNA due to background noise (Fig. 2(C) and ESI,† Tables S1–S4). Our results also show that AMV RT can be used as efficient tool to determine UB-content in RNA due to quantitative stopping at the unnatural base position in absence of its cognate TP.

In presence of the dUB TP, AMV RT generated 14% and 35% full-length cDNA in reverse transcriptions of 1xrTPT3 or 1xrNaM-RNA, respectively (Fig. 2(B) and (C)). Using 2xrTPT3/rNaM-RNA as template, AMV RT is completely stalled at the site of the first unnatural base independent of the presence of the UB TP. For MMLV RT and its variant SS IV RT, we detected full-length ratios ranging from 82% to 93% for reverse transcriptions of 1xrTPT3 or 1xrNaM-modified RNA in presence of the dUB TP. If dUB TP was not added to the reactions, both enzymes generated between 29% and 47% full-length fragments caused by mutagenic readthrough due to mismatch with canonical nucleobases. Surprisingly, MMLV RT and SS IV RT produced 79% to 93% full length cDNA in reverse transcriptions of 2xrNaM-RNA if dTPT3 TP was added to the reaction, while only minor amounts of full-length cDNA were observed for 2xrTPT3-RNA. We further observed stalling of reverse transcription with MMLV RT a few base positions located after the site of the UB (Fig. 2(B)). For all reverse transcriptions, MMLV RT shows the tendency to partially stop between the estimated base positions 29–31 after UB incorporation. Most likely, steric clashes in the post-insertion site prevent further efficient elongation of cDNA in the polymerase active site which has also been reported for RNA-dependent RNA polymerases.^36^

In contrast, SS III RT generated a lower percentage (59% to 67%) of full-length cDNA when reverse transcribing 1xrTPT3 or 1xrNaM-RNA in presence of the dUB TP, respectively (Fig. 2(C)) and nearly no full-length cDNA without addition of the cognate deoxy UB TP. Reverse transcriptions of 2xrTPT3-RNA with SS III in presence of the dNaM TP resulted in 1,6% full-length cDNA, while reverse transcriptions of 2xrNaM-RNA yielded 23% full-length cDNA when dTPT3 TP is added as substrate.

Using a more complex template, we reverse transcribed rTPT3-SPINACH2 RNA,^37^ initially prepared by T7 *in vitro* transcription, by employing the four reverse transcriptases tested before (see ESI,† Fig. S2). We verified successful and quantitative rTPT3 incorporation into SPINACH2 RNA by reverse transcription with AMV RT. The observed full-length cDNA ratio for the other RTs were comparable to the cDNA ratios for the 48-nt RNA and 80-nt RNA templates (Fig. 2 and ESI,† Fig. S1 and S2), indicating no pronounced sequence dependency of the reverse transcription reaction. In conclusion, SS IV RT seems to be capable of generating full-length cDNA nearly quantitively from rTPT3 or rNaM-modified RNA without showing sequence dependency. Comparing dTPT3 TP and dNaM TP processing, reverse transcriptions of rNaM-modified RNA generally showed slightly increased amounts of full-length cDNA suggesting reverse transcriptases might tolerate dTPT3 TP more likely as substrate than dNaM TP. This observation is in accordance with previously published data on *in vitro* transcription of the TPT3:NaM base pair.^25^

### MMLV and SS IV reverse transcription monitoring in real-time showed significant differences in their activity

**Fig. 3:**
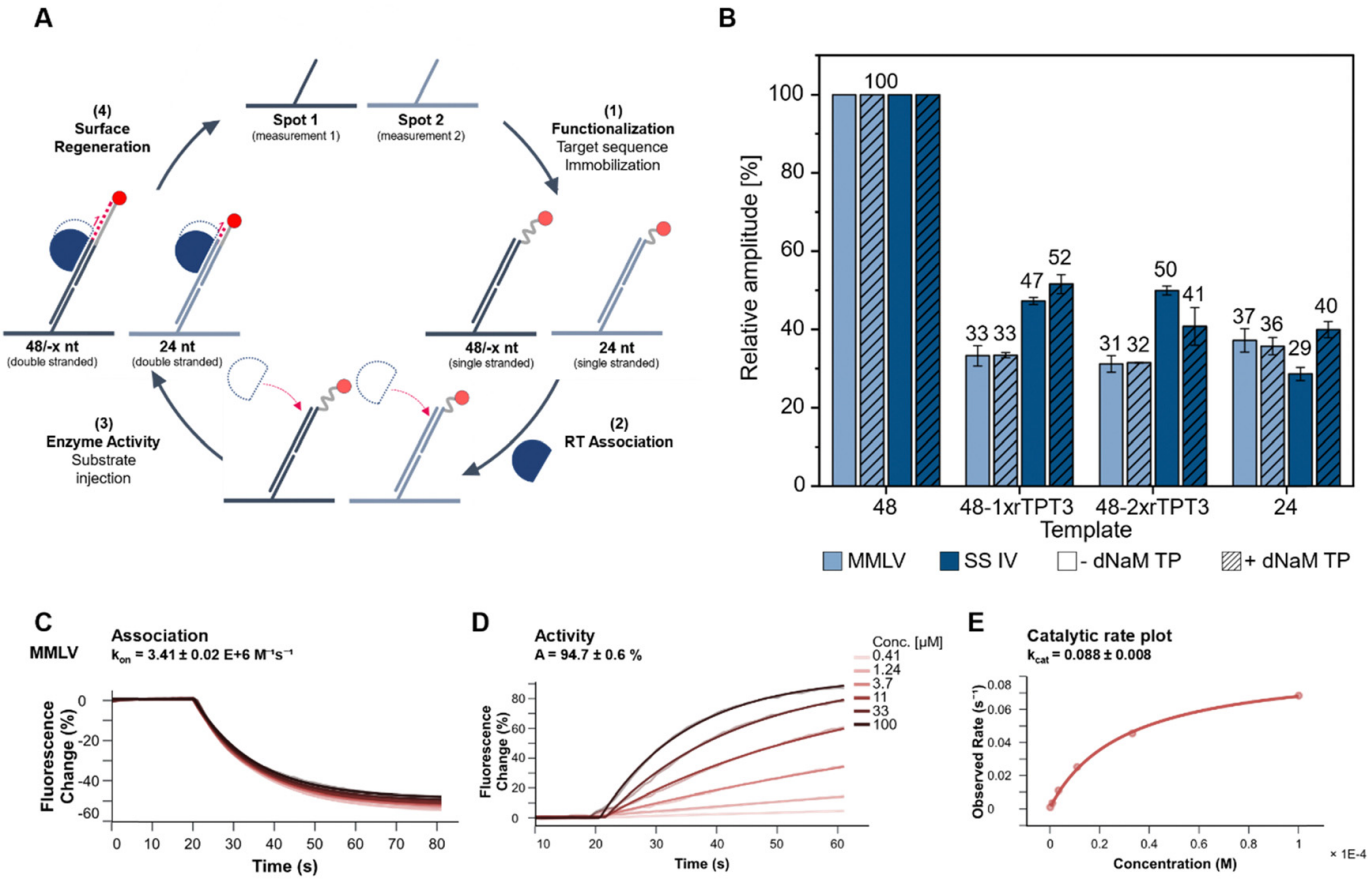
Analyzing MMLV and SS IV RT activity on biochip in a flow setup. (A) Schematic workflow of chip measurements. First, ligands are immobilized to the single stranded anchors by hybridization (1). Then, the RT is associated (2), followed by injection of dNTPs or dNTPs + dNaM TP to initiate reverse transcription (3). Finally, the surface is regenerated (4). (B) Relative amplitude of each MMLV and SS IV RT in the presence or absence of dNaM TP of the different templates including their standard deviation (*n* ≥ 3) derived from the fluorescence change of the amplitude of the elongation. Values for 48-1xrTPT3, 48-2xrTPT3 and 24-nt RNA are normalized to 48-nt unmodified RNA. (C) Fluorescence change signal of MMLV during the association phase. (D) Increase of fluorescence change during the reverse transcription (elongation) at different dNTP concentrations. (E) The catalytic rate plot where the observed rate is plotted over the substrate concentration to obtain *k*_cat_.

So far, end-point analysis of the reverse transcription reaction showed efficient cDNA synthesis by both MMLV and SS IV RT in presence of the UBP. To further gain information on binding kinetics and primer elongation by MMLV and SS IV RT, we performed kinetic measurements on a heliX^+^ biosensor tracking real-time reverse transcription. The binding and activity of the RTs were measured on a DNA-based chip surface monitoring the change in fluorescent signal of a 5′-fluorescently labeled RNA template. This setup enabled monitoring the binding affinities and the reverse transcription reaction in real-time. The different steps of the workflow are schematically shown in Fig. 3(A) and summarized as follows: (1) immobilization of the RNA template to the anchor DNA strand, (2) association of the polymerase, which ensure a stable and reproducible binding to the RNA (Fig. 3(C) and ESI,† Fig. S3), (3) elongation during reverse transcription upon binding of increasing concentrations of dNTPs with or without dNaM. (Fig. 3(D), ESI,† Fig. S4 and S5) and (4) regeneration of the surface. At the beginning of the measurement, the fluorescent dye at the 5′-end is quenched by the gold surface of the chip. During elongation of the primer, the complex of RNA and nascent cDNA becomes more structured and rigid. An increase in the rigidity of the double strand causes the fluorescent dye to move away from the gold surface, resulting in an increase in the fluorescence signal.^38^ Elongation of an unmodified 48-nt RNA template was used as a reference and was set to a relative amplitude of 100%. Data obtained for 48-nt 1x or 2xrTPT3-RNA was normalized to the unmodified template (Fig. 3(B)). In parallel to measurements on the 48 nt RNA, reverse transcription on a 24 nt long unmodified RNA was recorded in spot 2 as control, reflecting truncation directly before the UB position.

Both MMLV and SS IV RTs elongated the cDNA completely opposite to the unmodified RNA. In comparison to unmodified RNA, MMLV RT showed significantly lower activity for RNA containing one or two rTPT3 modifications. Reverse transcription of both rTPT3-modified RNAs in absence of dNaM TP resulted in a similar change of amplitude (33% and 31% for 1x- and 2xrTPT3-RNA, respectively) as observed for the 24 nt long RNA (37%). Interestingly, the addition of dNaM did not affect elongation and caused the same change in amplitude. We therefore assume that MMLV RT stops at the site of the unnatural base in real-time measurements. In comparison, SS IV RT showed a significantly enhanced activity for reverse transcription of rTPT3-RNA. We measured for reverse transcriptions of 1xrTPT3-RNA a relative change of amplitude of 47% for reactions without dNaM TP addition. A significant increase in the change of amplitude was not detected if dNaM TP was added to measurements. The elongation of the RNA with one or two rTPT3 modifications also showed no difference in amplitude, preventing differentiation between dNaM incorporation or a potential mismatch using real-time monitoring. In contrast to our previous end-point analyses of the reverse transcription which allowed rebinding of the polymerase to the RNA:DNA duplex, we now observe that SS IV RT processed the UB position while MMLV RT stopped reverse transcription prior to UB processing. The experimental setup on the chip surface in a constant flow does not allow rebinding of the RT after dissociation from the primer–template complex. Thus, the switchSENSE technology allows to gain first insights into mechanistic details how RTs handle the TPT3:NaM base pair. Pausing and stopping in case of RNA structural elements is common for the retroviral RTs used in this study.^39^ Dissociation and rebinding of the RTs seems to be crucial for efficient processing of the TPT3:NaM base pair by reverse transcriptases.

Furthermore, a three times higher catalytic rate *k*_cat_ was detected for SS IV RT (*k*_cat_ = 0.247) compared to MMLV RT (*k*_cat_ = 0.088, ESI,† Fig. S6), which is consistent with the manufacturer's information reporting higher processivity for SS IV RT.^40^ We assume, that the higher *k*_cat_ potentially correlates with an increased ability of SS IV RT to process the TPT3:NaM base pair.

All in all, these results indicate, that SS IV RT exhibits higher tolerance, conversion, and processing of the TPT3:NaM pair in comparison to MMLV RT.

### Detection of TPT3 incorporation into cDNA

**Fig. 4:**
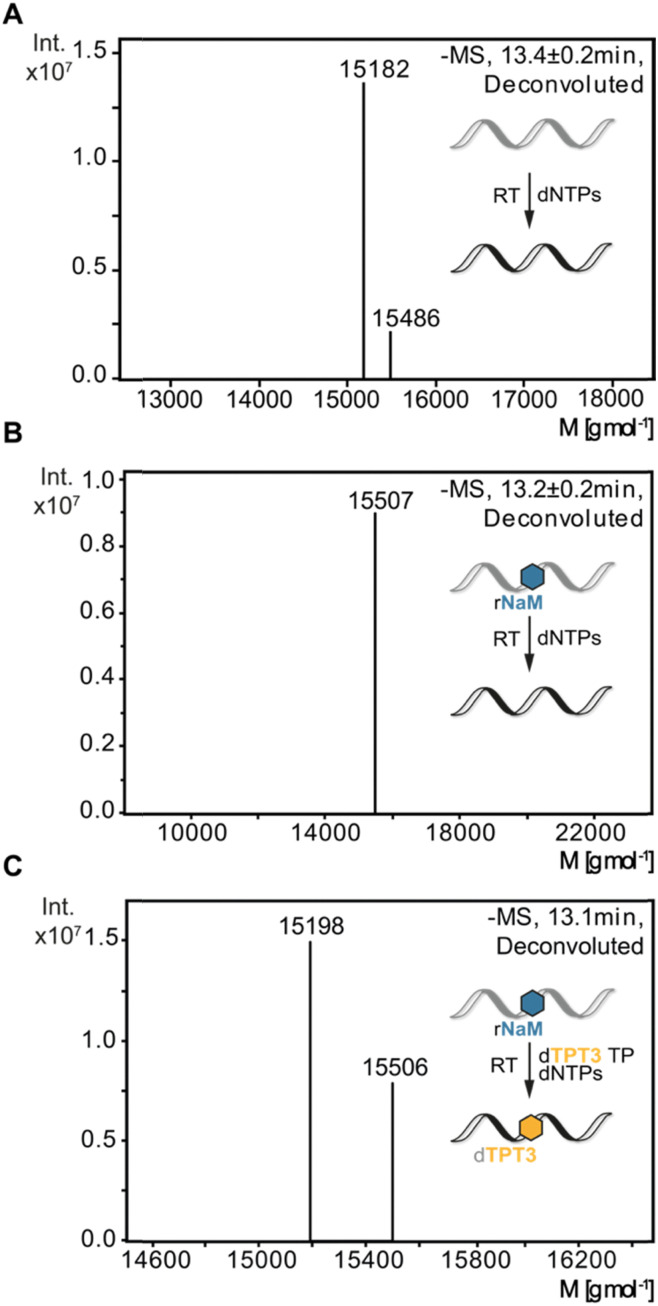
ESI-MS analysis of reverse transcriptions with SS IV RT. (A) Reverse transcription of 48-nt unmodified RNA. (B) Reverse transcription of 48-nt 1xrNaM-RNA without dTPT3 TP addition. (C) Reverse transcription of 48-nt 1xrNaM-RNA with dTPT3 TP addition. Calculated masses: M (unmodified cDNA) = 15 182 g mol^−1^, M (unmodified cDNA + dT) = 15 486 g mol^−1^, M (unmodified + dT + Na^+^) = 15 507 g mol^−1^, M (dTPT3-modified cDNA) = 15 198 g mol^−1^.

To verify sequence specific UB incorporation into full-length cDNA fragments, we examined reverse transcriptions by liquid chromatography coupled with ESI mass spectrometry (LC-MS). For control reverse transcriptions of unmodified RNA, we identified unmodified full-length cDNA (15 182 g mol^−1^) for MMLV, SS III, and SS IV RTs. Next, synthetic 48-nt 1xrNaM-RNA was reverse transcribed in the absence and presence of dTPT3 TP and subjected to LC-MS. In general, two main products were observed: truncation of the cDNA at the site of the UB prior to dUB TP incorporation or full-length cDNA containing dTPT3 or a dTPT3 → dG mutation. For MMLV and SS III RT, we detected truncated fragments (7753 g mol^−1^) corresponding to abortion one nucleotide before the UB position for reactions in absence and presence of dTPT3 TP (ESI,† Fig. S8B, C and S9B, C). In contrast, we did not identify truncated fragments for SS IV RT. Interestingly, SS IV RT adds extra nucleotides at the 3′-end of full-length cDNA (15 486 g mol^−1^ for unmodified full-length + dT and 15 507 g mol^−1^ for unmodified full-length + dT + Na^+^, Fig. 4). Measurements of reverse transcriptions without dTPT3 addition revealed a dTPT3 → dG mutation in full-length cDNA for MMLV, SS III and SS IV RT. For reverse transcriptions in presence of dTPT3 TP, we detected dTPT3-modified full-length cDNA for MMLV, SS III and SS IV RT (Fig. 4(C) for SS IV RT and ESI,† Fig. S8C and S9C for MMLV and SS III RT, respectively). However, a dTPT3 → dG mutation in full-length cDNA was also observed for reactions in presence of dTPT3 TP for MMLV, SS III and SS IV RT.

In summary, LC-MS analysis supports incorporation of dTPT3 into cDNA opposite rNaM for all three reverse transcriptases. However, dG misincorporation opposite rNaM are likewise detected, albeit the percentage of UB incorporation cannot be determined by qualitative MS.

### Fidelity of the TPT3:NaM base pair during reverse transcriptions

**Fig. 5:**
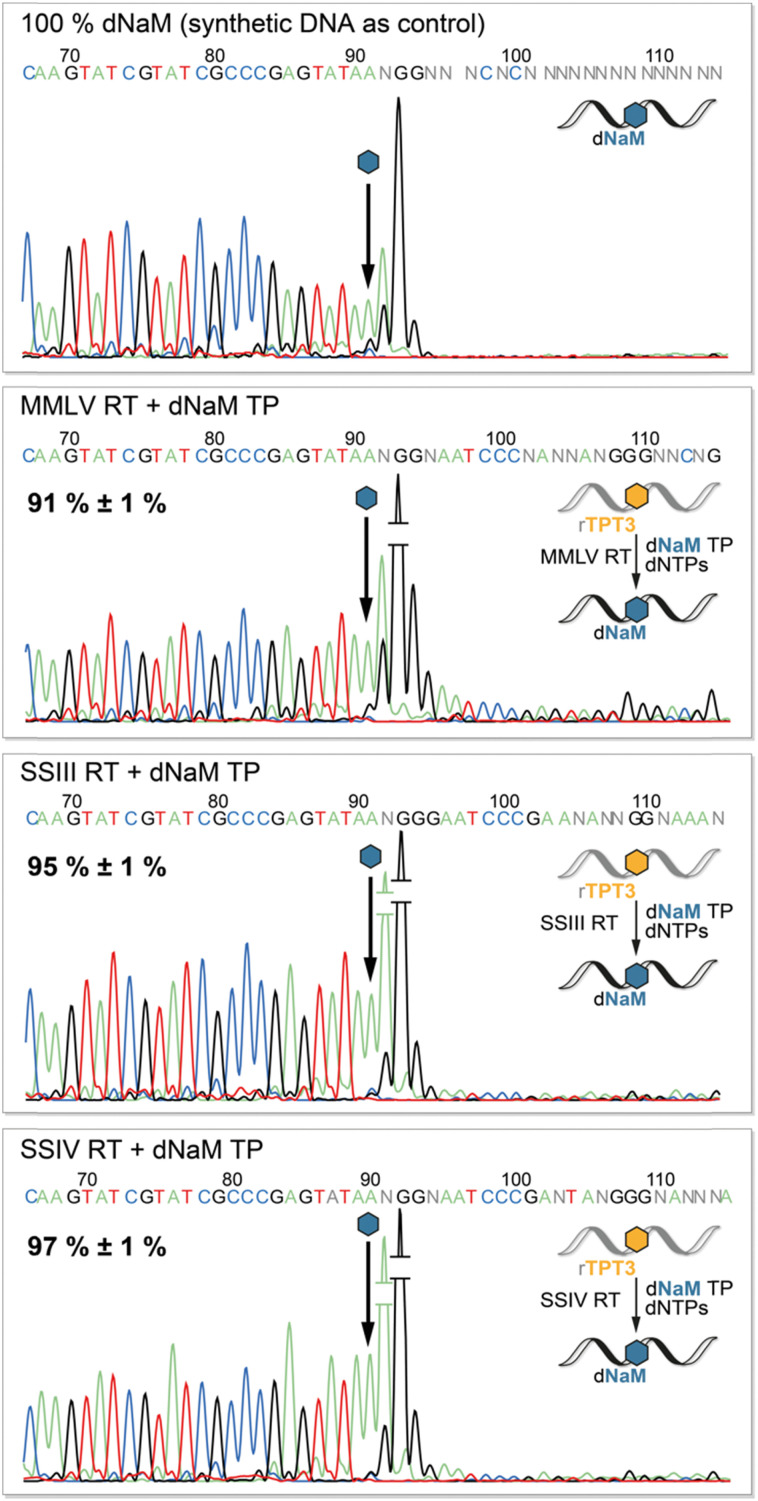
Sanger Sequencing of quantitatively dNaM-modified DNA (produced by solid phase synthesis) and cDNA obtained from reverse transcriptions with MMLV, SS III and SS IV RT in presence of dNaM TP. The arrow indicates the dNaM position in cDNA. The percentages are the calculated fidelities of the TPT3:NaM pair obtained for the different reverse transcriptases.

To determine the fidelity of UB incorporation by reverse transcriptases, we performed Sanger Sequencing of the cDNA adopting a method by the groups of Hirao and Romesberg for DNA amplification *via* PCR.^12,41,42^ The sequencing reaction stops after the UB position as the DNA polymerase in the BigDye Terminator mix cannot process the TPT3:NaM base pair.^41^ Consequently, we observe complete abortion of the sequencing reaction three positions after the UB position using synthetic DNA containing dNaM as control (Fig. 5, upper panel). Depending on the rate of misincorporation at the UB position during reverse transcriptions, the peak height of the following bases increases in the sequencing chromatogram. In contrast to termination after the UB position, the polymerase proceeds with the sequencing reaction at the mismatch position, resulting in an increase in fluorescence and thus peak height. Therefore, we determined the fidelity of the TPT3:NaM base pair during reverse transcription by comparing peak heights before and after the dNaM position. To calculate the fidelity, we generated a calibration line based on sequencing of known ratios of natural and dNaM-modified DNA (ESI,† Fig. S10–S13). We detected an overall fidelity of 91% ± 1% for MMLV RT and 95% ± 1% for SS III RT, while SS IV RT incorporates the TPT3:NaM base pair into cDNA with an overall fidelity of 97% ± 1% (Fig. 5). This is in accordance with our previous results, that SS IV RT process the TPT3:NaM base pair most effectively among the tested polymerases.

### Retention of the TPT3:NaM base pair during SELEX cycles

**Fig. 6:**
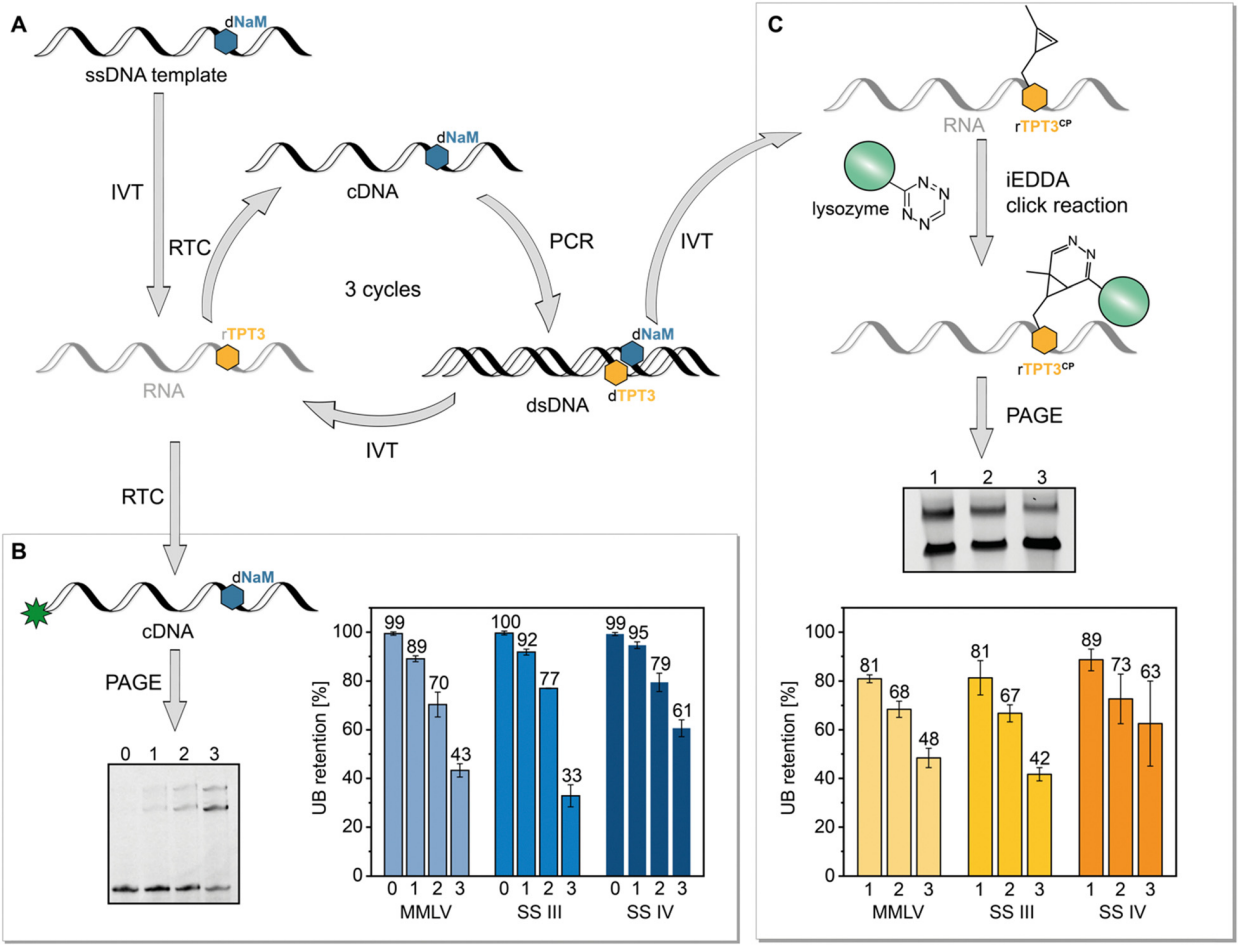
SELEX cycles with a single nucleic acid sequence to determine UB retention. (A) Schematic presentation of the performed SELEX cycle. The single-stranded (ss) synthetic DNA template containing dNaM was transcribed into RNA with rTPT3 TP. Three cycles of reverse transcription (RTC), PCR and *in vitro* transcription (IVT) with UB TP were performed. (B) RNA of each cycle was reverse transcribed with a 5′-6-carboxyfluorescein primer and AMV RT in the absence of dNaM TP to determine UB retention, (C) dsDNA of each cycle was transcribed into RNA with rTPT3^CP^ TP enabling determination of UB retention by a mobility shift assay based on iEDDA cycloaddition. For this, rTPT3^CP^-modified RNA was incubated with tetrazine-modified lysozyme, analyzed by PAGE and the UB retention was calculated as function of shifted RNA (see ESI,† Fig. S15).

Based on our previous reported results, we aimed to explore the possible application of the TPT3:NaM base pair in *in vitro* selection of RNAs using the reverse transcriptases MMLV, SS III and SS IV which can process the TPT3:NaM base pair. In particular SS IV RT, with an overall fidelity of 97%, is a promising candidate for SELEX applications. The here presented method based on a simulated SELEX cycle using a single nucleic acid sequence instead of a library of sequences also allowed to calculate retention rates of the UBP during reverse transcription. For this, we performed SELEX cycles using the SPINACH2 RNA aptamer sequence.^37^ To simulate SELEX experiments, we transcribed the dNaM modified DNA template coding for SPINACH2 into RNA with rTPT3 TP and performed three iterative cycles of reverse transcription, PCR, and transcription with UB TPs (Fig. 6(A)). To determine the incorporation rate of TPT3:NaM, we relied on our approach on reverse transcription with AMV RT (Fig. 6(B)). As described above, AMV RT quantitively stalls at the site of the UB, enabling the determination of UB incorporation into RNA. This indirectly allows to verify the incorporation rate of the UB on reverse transcription level, because TPT3:NaM is processed with natural-like fidelity greater than >99.98% during PCR with OneTaq DNA Polymerase^21,22^ and also quantitively incorporated into RNA by T7 *in vitro* transcription.^25^ To determine UB retention after SELEX cycles, values obtained for reverse transcriptions of RNA after the first, second and third cycle were then normalized to values obtained for chemical synthesized 48-nt 1xrTPT3-RNA to minimize the systematic error of fluorescence readout. By this, we calculated an UB retention of 89% for MMLV RT, 92% for SS III RT and 95% for SS IV RT after the first complete cycle. However, a significant loss of the UB can be observed over the three cycles performed. In case of MMLV and SS III RT, UB retention decreased to 43% and 33%, respectively, while 61% of RNA contained TPT3 after three cycles with SS IV RT (Fig. 6(B)).

To validate these results, we further examined UB incorporation by a mobility shift assay. After each PCR step with the TPT3:NaM base pair, we additionally subjected the DNA to T7 *in vitro* transcription using a rTPT3 TP derivative bearing a cyclopropene moiety (rTPT3^CP^, Fig. 6(C), for structure see ESI,† Fig. S15).

In previous studies, we demonstrated efficient rTPT3^CP^ incorporation into RNA allowing posttranscriptional functionalization *via* iEDDA cycloadditions.^24,27,28,43^ We now applied this strategy for the development of an electromobility shift assay based on iEDDA reactions. To our knowledge, this is the first time that a mobility shift assay based on click reactions is presented as an alternative to the frequently used strategy relying on biotin–streptavidin interaction. Compared to the required biotin functionalization of the nucleic acid, strained alkenes provide a smaller reactive handle for covalent attachment of the reporter protein, thus not interfering with the generation and function of the nucleic acid. We functionalized commercially available lysozyme with a tetrazine *via* NHS ester chemistry^44^ (see ESI,† Section 6 for further information) which is then incubated with rTPT3^CP^-modified RNA causing a mobility shift of rTPT3^CP^ modified RNA during PAGE. As a result, we can determine the ratio of unshifted and shifted RNA that correlates to the UB retention during reverse transcription. For SELEX with MMLV RT, we detected an UB retention of 81% for the first cycle, which decreased to 48% in the third cycle. For SS III RT we observed similar results with an UB retention of 81% after the first and 42% after the third cycle. Experiments, in which SS IV RT was applied for reverse transcription, resulted in 89% retention of rTPT3 after the first cycle and 63% after the third cycle.

Taken together, we clearly observe a similar trend using both methods to detect UB retention during SELEX: a significant loss of UB over three SELEX cycles is observed for all reverse transcriptases tested. SS IV RT showed the highest UB retention. However, even using SS IV RT, its processivity and fidelity is not sufficient for SELEX experiments. Our findings demonstrate the impact of polymerase fidelity on the UB retention and its crucial factor for all SELEX applications. Even a fidelity of 97% as determined for SS IV RT, with an UB retention of 95% after the first SELEX cycle leads to a considerable loss of the UB after only three cycles. Due to the exponential increase of the number of copied DNA during PCR, the UB retention during reverse transcription is critical as the cDNA serves as template in PCR reactions. A UB retention of >99% is therefore required in reverse transcription to prevent a progressive and significant UB loss during PCR. Application of SS IV RT to SELEX with the TPT3:NaM pair would result in a total loss of the UB after the typical 7–10 performed cycles, especially for sequences bearing more than one TPT3 modifications. Thus, polymerase engineering is required. For this, SS IV RT is a promising candidate due to its already efficient TPT3:NaM processing to achieve the conditions required for SELEX applications.

## Conclusion

In this study, we have presented various approaches for the characterization of the reverse transcription process of UBPs. We concentrated on four commercially available RTs, AMV RT and MMLV RT and its engineered variants SS III and SS IV. AMV RT was not able to generate notable amounts of full-length cDNA using both, rTPT3 or rNaM modified RNA templates and the cognate UB triphosphates. However, quantitative stopping of AMV RT at the UB position in the absence of dNaM TP or dTPT3 TP can be used to verify UB-incorporation in RNA, as we had reported before.^25^ We now employed this method to develop assays for the determination of UB retention during SELEX. We showed that MMLV RT and its engineered variants SS III and SS IV process both, dTPT3 TP and dNaM TP opposite rNaM and rTPT3 respectively during reverse transcription. Using mass spectrometry of cDNA products, we further identified the main mismatch causing loss of the UB in reverse transcription, which is a dTPT3 → dG mutation for MMLV, SS III and SSIV RT. The fidelity of polymerases can be efficiently determined by Sanger Sequencing with SSIV RT showing the highest fidelity (97%). Real-time monitoring of reverse transcription, using switchSENSE technology and the heliX^+^ biosensor enabled kinetic characterization of binding and activity of MMLV and SSIV RT to RNA templates making it a powerful tool that can be applied for studying other reverse transcriptases and polymerases in general.

To determine, whether currently available polymerases allow the use of the TPT3:NaM base pair in RNA SELEX, we investigated the UB retention during several rounds of *in vitro* selection. We observed a substantial loss of the UB over three cycles for all polymerases tested, although SSIV RT showed the highest retention of the TPT3:NaM pair after three cycles (61%).

The herein reported methods can be readily adapted to characterize the reverse transcription of virtually any modified or artificial nucleotide. We also provide a powerful alternative to the commonly used streptavidin–biotin shift assay to determine the incorporation efficiency and retention of artificial nucleotides based on iEDDA cycloaddition using a tetrazine modified protein. This approach also enables the universal application for UB detection in nucleic acids at PCR, transcription, and reverse transcription level.

Our studies identified SS IV RT as the most promising polymerase processing the TPT3:NaM pair reliably with the highest fidelity among the tested RTs and a high UB retention rate in a single reverse transcription step. However, we found that the fidelity and efficiency are not yet sufficient for SELEX applications. We therefore propose engineering as a next step, with SS IV RT being a potential candidate. A reverse transcriptase incorporating UBPs with natural-like fidelity is required for the selection of RNA aptamers but also paves the way towards the evolution of dual-modification XNAs based on an expanded genetic alphabet.

## Data availability

All raw data have been included in the ESI.†

## Author contributions

E. S. H. conceived the study, designed the experiments, performed the following experiments: enzymatic preparation of RNA, quantification of cDNA, LC-MS, Sanger Sequencing, SELEX and assays for UB retention, analyzed the data, interpreted the results and wrote the manuscript. M. C. P. designed and performed kinetic experiments using SwitchSENSE® technology, analyzed and interpreted kinetic data, and wrote the manuscript. L. N. synthesized rTPT3 and rNaM phosphoramidites and prepared RNA oligonucleotides by solid-phase synthesis. C. D. synthesized rNaM, rTPT3, rTPT3^CP^ and dTPT3 TP. A. S. designed kinetic experiments and interpreted kinetic data. S. K. S. conceived the study, designed the experiments, interpreted the results, wrote the manuscript, and organized and supervised the entire research program. All authors read and approved the manuscript.

## Conflicts of interest

There are no conflicts to declare.

## Supplementary Material

CB-005-D4CB00084F-s001
