# Supplementary material for: Reverse transcription as key step in RNA in vitro evolution with unnatural base pairs
Source: RSC Chem Biol. 2024 Apr 23;5(6):556–66. doi: 10.1039/d4cb00084f (PMC11151862; doi:10.1039/d4cb00084f)
Supplement: CB-005-D4CB00084F-s001 [file CB-005-D4CB00084F-s001.pdf]

Electronic Supplementary Material (ESI) for RSC Chemical Biology.
This journal is © The Royal Society of Chemistry 2024

Supporting Information

Reverse transcription as key step in RNA *in vitro* evolution with unnatural base pairs

Eva S. Hoffmann[1], Mareike C. De Pascali[2,3], Lukas Neu[1], Christof Domnick[1], Alice Soldà[3] and Stephanie Kath-Schorr[1,*]

Inhaltsverzeichnis

1. Methods 1

2. Quantification of full-length cDNA via Polyacrylamide Gel Electrophoresis 7

3. Kinetic measurements 12

4. LC-MS data 15

5. Sanger Sequencing 17

6. SELEX cycle: iEDDA shift assay and AMV RT reverse transcription to determine UB retention 20

7. Sequencing gels used for data generation in this study: 24

8. DNA and RNA oligonucleotides 50

9. Synthesis of phosphoramidites for RNA synthesis 52

10. References 109

1. Methods

General

dNaM TP was purchased from *MyChem,* USA. dTPT3 TP, rTPT3 TP, rTPT3^{CP} TP and rNaM TP were synthesized as previously reported.[1-4]

Concentrations of all purified nucleic acid samples were determined by measuring the absorption at 260 nm (A260) using a spectrophotometer DS-11 FX+ (*DeNovix*).

Synthesis of oligonucleotides

Unmodified oligonucleotides were synthesized by *ELLA Biotech* or *biomers.net*. dNaM-modified DNA and rTPT3-modified RNA oligonucleotides were synthesized by *ELLA Biotech* using dNaM cyanoethyl phosphoramidite (CEP) (*Berry & Associates Inc.,* USA) and rTPT3 CEP (synthesized in-house). rNaM-modified RNA oligonucleotides were synthesized were synthesized in-house natural CEP (*GlenResearch, LGC Biosearch Technologies, Hongene Biotech*) and rNaM CEP (synthesized inhouse). A detailed description of rNaM and rTPT3-phosphoramidite synthesis and preparation of oligonucleotides via solid-phase synthesis can be found in section 9.

In vitro Transcription

DNA templates were *in vitro* transcribed in 50 or 100 µL scale containing 40 mM Tris pH 7.9, 25 mM $MgCl_2$, 5 mM dithiothreitol (DTT), 2.5 mM dNTPs, 0 or 1 mM rTPT3 or rTPT3^{CP} TP, 0.5 U µL^{-1} RNAsin (*New England Biolabs*), 2.0 ng µL^{-1} inorganic pyrophosphatase (iPP, *New England Biolabs*), 1.5 µM Primer, and 1.5 µM DNA template. Primer and DNA template were annealed in Tris buffer and $MgCl_2$ by denaturation for 2 min at 95 °C and subsequent cooling at 2 min on ice. 5 U µL^{-1} T7 RNA polymerase (made in-house) was added last to the reaction mixture. *In vitro* transcriptions were incubated at 37 °C for 2 hours.
After transcription, the DNA template was digested by adding 0.04 U µL^{-1} DNase I in 6.25 or 12.5 µL 10 x DNase I reaction buffer (*New England Biolabs*). Reactions were incubated for 30 min at 37 °C and DNase I was subsequently inactivated for 2 min at 95 °C. RNA fragments were analyzed by 12 % PAGE and purified by gel filtration using G25 columns (*GE Healthcare, Cytiva*).

Reverse transcription

Reverse transcription reactions were performed using four different reverse transcriptases according to the manufactures protocol: AMV RT (*New England Biolabs*), MMLV RT (*New England Biolabs*), SS III (*Invitrogen*) and SS IV RT (*Invitrogen*). All reactions were prepared in 20 µL scale with final concentrations of 20 to 40 pmol primer, 0.5 mM dNTPs, 0 or 0.5 mM dNaM TP, 1 x reverse transcriptase reaction buffer, 0.25 U µL^{-1} to 10 U µL^{-1} reverse transcriptase and 300-500 ng RNA, unless otherwise specified. For SuperScript RTs, DTT was added to 10 mM to the reaction.

The primer was annealed to RNA in the reaction buffer by heating to 95 °C for 2 min and subsequent cooling on ice. Reverse transcriptions were incubated for 60 min at 42 °C for AMV and MMLV RT, 20 min at 50 °C for SS III and 20 min at 55 °C for SS IV RT. RTs were inactivated for 5 min at 85 °C for AMV RT, 20 min at 60 °C for MMLV RT and 10 min at 80 °C for SS III and SS IV RT.
Subsequently, the remaining RNA was digested by adding RNase H (*New England Biolabs*) to a final concentration of 0.1 U μl^{-1}. RNase digestion was performed by incubation at 37 °C for 20 min and enzyme inactivation at 70 °C for 10 min.

Quantification of full-length cDNA products

To quantify full-length cDNA products, a 5'-6-FaM-labeled primer was employed in reverse transcription reactions. After RNase H digestion, the samples were mixed with 2 x loading buffer and loaded onto a 20 % denaturing polyacrylamide gel (160x175 mm). After PAGE run at 120-150 V overnight, fluorescence was read-out using a ChemoStar Touch imager (*INTAS*). Each experiment with the different reverse transcriptases and RNA templates were at least performed in triplicates.

Data analysis
Fluorescence intensities of denaturing Polyacrylamide Gel Electrophoresis (PAGE) were analyzed and visualized with LabImage 1D (Kapelan). Lanes and bands were set, and the background was substracted applying the method “minimum to minimum”.
Relative full length cDNA intensities were calculated by forming the quotient of the resulting fluorescence values of full-length fragment and the sum of fluorescence intensities of full-length and stop products. For each reverse transcriptase, mean and standard deviation was calculated and ratios of full-length cDNA were plotted using Origin 2023.

Kinetic measurements

All measurements were performed on a heliX$^+$ biosensor (*Dynamic Biosensors GmbH)* using fluorescence proximity sensing (FPS - also called static mode) and the enzyme activity mode.[5, 6] A negative voltage ensured that DNA strands on the chip surface stand in upright position, so that the fluorophore is nor in contact neither in proximity of the gold surface of the chip, which can lead to quenching of its fluorescence signal.[7, 8] The chip has two electrodes (also called spots) that are spatially separated but located in the same fluidic channel and single photon detectors ensure continue monitoring of the fluorescent signal. All interactions are observed by excitation of the fluorophore in red (600-630nm excitation – 650-690nm emission) and all experiments were performed at 25 °C.

Experimental setup
All the ligands contained a 48-nt long DNA sequence (ligand strand) on the 3'-end for hybridization to the adapter on the biochip. On the 5'-end, they contained a 24-nt or 48-nt long RNA overhang for reverse transcription. The rTPT3 was placed at position 25 and 25+26 in the RNA overhang for the constructs (48-nt 1xrTPT3-RNA and 48-nt 2xrTPT3-RNA, see table 1). A red fluorescent rhodamine derivative dye is located on the 5'-end for optical detection.

The ligands were hybridized to the adapter in a final concentration of 200 nM to 250 nM for each spot separately (2 min at 650 rpm, 70 °C, then 20 min at 650 rpm, 25 °C in a horizontal shaker) and then mixed in a 1:1 v/v ratio. Spot 1 was functionalized with the 48-nt long ligand (unmod-, 1x-, 2-xrTPT3-RNA) and spot 2 always with the 24 nt RNA construct. MMLV RT was diluted to a concentration of 20 nM and SS IV RT to 50 nM in RT buffer (50 mM Tris pH 8.3, 75 mM KCl, 0.05 % Tween20, 3 mM $MgCl_2$) plus 1 mM TCEP. dNTPs were also diluted in RT buffer plus 1 mM TCEP and when dNaM was used it was mixed with dNTPs in a 1:1 v/v ratio. First, the ligands were immobilized on the chip by hybridization for 200 s. Then the RT was associated for 60 s at a flow rate of 100 µL min^{-1}. For the activity, either dNTPs or a dNTPs/dNaM mixture were injected for 40 s at a flow rate of 50 µL min^{-1}. Then the chip was regenerated by injection of a high pH solution and the next dNTP concentration was measured. Experiment, design, workflow, and data analysis were performed with the heliOS software (*Dynamic Biosensors GmbH*) and statistical analysis with Python.

Data Processing

All measurements were at least 3 times repeated and blank referenced. Data analysis was done with heliOS and Python analysis and graphs were generated with Origin 2023. The Michaelis-Menten model was the basis for all data fitting and evaluation of the real-time enzymatic activity under the assumptions that the enzyme concentration during activity was constant, the elongation was irreversible, no allosteric effects of the RT present, the concentration of the nucleotides was constant due to the flow and the equilibrium of substrate and enzyme was rapid.[9] The formula for enzyme association was applied to the binding of the enzyme to the ligand under the assumption that k_{off}= 0. The formula for the enzymatic activity was applied to all fluorescent curves and the amplitude (A) was extracted for all measurements. The change of the fluorescence amplitude of the unmodified 48-nt RNA template was set to 100 % and the other templates relative to it. The standard deviation was calculated for all of them. The observed rate k_{obs} of the exponential enzymatic activity was plotted versus the substrate concentration to obtain the rate of the catalytic step k_{cat}.

Michaelis-Menten model:

$$E + S \underset{k_{-1}}{\overset{k_1}{\rightleftharpoons}} ES \xrightarrow{k_{cat}} E + P$$

Fit for enzyme association with k_{off}= 0

$$y = y_0 + A \cdot \left(1 - e^{-(k_{on} * c + k_{off}) \cdot (x - t_{on})}\right)$$

$$y_0 = Baseline$$

$$A = Amplitude$$

$$k_{on} = Association\ rate$$

$$c = concentration$$

$$k_{off} = Dissociation\ rate$$

$$t_{on} = Association\ start$$

Fit equation for enzymatic activity:

$$y = y_0 + A \cdot \left(1 - e^{-k_{obs} \cdot (x - t_{act})}\right)$$

$$y_0 = Baseline$$
$$A = Amplitude$$
$$k_{obs} = Observed\ rate$$
$$t_{act} = Activity\ start$$

Fit equation for k_{cat} determination with the Michaelis-Menten Model:

$$k_{obs} = \frac{\mathrm{k_{cat}} \cdot [\mathrm{S}]}{K_M + [S]}$$
$$k_{obs} = Observed\ rate$$
$$k_{cat} = Catalytic\ rate$$
$$[\mathrm{S}] = Substrate$$
$$K_M = Michaelis - Menten\ constant$$

HPLC-ESI-MS

HPLC-ESI-MS was performed using an Amazon SL mass spectrometer in combination with a Elute SP liquid chromatography system (*Bruker Daltonics*). For analysis via HPLC-ESI-MS, reverse transcriptions were performed in 50 µL scale and desalted using Amicon® Ultra-0.5 centrifugal filter devices *(Merck Millipore)*. HPLC measurements were performed on a Xterra MS C18 column (2.1 x 100 mm, 5 µm, *Waters*) using 10 mM triethylamine/100 mM 1,1,1,3,3,3-hexafluoro-2-propanol as solvent A and 100 % acetonitrile as solvent B. The following protocol was applied: 3 % B for 9 min followed by a gradient from 3 to 40 % B in 11 min at a flow rate of 0.2 mL min^{-1}. Spectra were analyzed using the software Compass Data Analysis v5.2 (*Bruker Daltonics*).

Sanger Sequencing

Sample preparation

For sequencing of short cDNA fragments after reverse transcriptions, a 107-mer “adapter” DNA was attached to full-length cDNA via overlap extension PCR. To specifically target full-length cDNA, the 107-mer includes a 17 nt long region at the 5'-end that is complementary to the 3'-end of full-length cDNA. Overlap extension PCR was performed in 100 µL scale containing 10 µL of reverse transcription reaction, 1 µM 107-mer, 375 µM dNTPs, 200 µM dTPT3 TP, 1 x OneTaq reaction buffer and 0.02 U $µL^{-1}$ OneTaq DNA polymerase *(New England Biolabs)*. The following PCR conditions were used: 1: 94 °C, 120 s, 2: 94 °C, 30 s, 3: 50 °C, 20 s, 4: 68 °C, 20 s; 10 cycles of step 2 to 4. Assembled products were separated by 4 % agarose gel electrophoresis and the corresponding band was excised and purified with the MONARCH DNA Gel Extraction kit (*New England Biolabs*).

Sequencing reaction

Sequencing of cDNA was performed in 10 µL scale using 2.25 µL of 5 x Sequencing buffer and 0.25 µL Cycle Sequencing mix of the BigDye Terminator v3.1 Cycle Sequencing kit (*Applied Biosystems*). Sequencing reactions contained 1.5 ng cDNA and 6 pmol poly-T

sequencing primer according to literature.[10] The following cycling conditions were applied: 1: 98 °C, 1 min, 2: 98 °C, 10 s, 3: 56 °C, 5 s, 4: 68 °C, 2.5 min; 25 cycles of steps 2-4. Samples were purified with the MONARCH DNA and PCR clean-up kit (*New England Biolabs*). Sequencing was performed on a 3730 DNA Analyzer (*Applied Biosystems*). Sequencing results were analyzed with Seq Scanner 2.0 Software (*Applied Biosystems*).

Sequencing calibration

Sequencing calibration was performed as described previously by Hirao and co-workers.[11, 12].Chemically synthesized oligos corresponding to the unmodified and single dNaM modified full-length cDNA including the adapter sequence was used for sequencing calibration. Natural and dNaM-modified oligos were prepared in the following ratios: 0:100, 1:99, 2:98, 3:97, 5:95, 7.5:92.5, 10:90 and 15:85. Sequencing reactions were conducted as described before using 1.5 ng of each mixture. Sequencing results were analyzed with Sequence Scanner 2.0 Software by setting the start and stop points of the 48-mer region. The peak maximum was then determined for G19 and T20 located before the UB position and T31, G37, T38, G40 and G43 after the UB position. Peak heights of the points located after dNaM were normalized to G19 or T20 and the normalized data were plotted against the percentage of natural oligo contained in the mixtures reflecting replacement of dNaM with a canonical base. A calibration line for each nucleotide position was generated by fitting with a linear equation (see section 5). The five different calibration lines were then used to calculate the overall fidelity for the different reverse transcriptases by using the normalized peak heights for the five nucleotide positions of the sequencing results for cDNA from reverse transcriptions. The five resulting values obtained from the calibration lines were averaged and the overall fidelity (F) was calculated as $F = 1 - x$, where x reflects the percentage of unmodified DNA.

Determination of UB retention via SELEX

SELEX cycle

For SELEX experiments, the unmodified and dNaM modified SPINACH2[13] DNA template was *in vitro* transcribed at 50 µL scale as described above. DNase digested and purified RNA transcripts were reverse transcribed with MMLV, SS III or SS IV RT with dNaM TP as described before. 5 µL of crude cDNA was amplified in 100 µL PCR reactions containing 1 x OneTaq reaction buffer (New England Biolabs), 375 µM dNTPs, 200 µM dNaM and dTPT3 TP, 0.5 µM forward primer and reverse primer (with an overhang including the T7 promoter for transcription) and 0.02 U µL^{-1} OneTaq DNA polymerase (*New England Biolabs*). The following PCR conditions were used: 1: 94 °C, 30 s; 2: 94 °C, 30 s; 3: 68 °C, 15 s; 4: 68 °C, 15 s; 5: 68 °C, 1 min; 40 cycles of steps 2 to 4. DNA products were purified using MONARCH PCR and DNA clean-up kit (*New England Biolabs*) according to the manufacturer's procedure.
The cycle of transcription, reverse transcription and PCR was repeated three times and for each sample set of reverse transcriptase in triplicate.

Reverse transcription with AMV RT

For determination of UB retention in RNA after SELEX cycles, 500 ng of obtained RNA after each transcription step was reverse transcribed with AMV RT using the 5'-6-FaM-labeled primer without the addition of dNaM TP as described above. Data analysis was conducted as described before and the ratio of stop and full-length cDNA was determined by forming the

quotient of the intensities of stop and sum of stop and full-length cDNA bands. UB retention was calculated by normalizing obtained stop ratios to that obtained for reverse transcriptions of chemical synthesized RNA.

<u>iEDDA shift assay with tetrazine-modified lysozyme</u>

To determine UB retention via electromobility shift assay based on inverse electron demanded Diels Alder (iEDDA) cycloaddition, DNA obtained from each SELEX cycle was *in vitro* transcribed with rTPT3^{CP} TP as described before. After purification of RNA, 20 pmol of RNA were incubated with 15 equivalents tetrazine-modified lysozyme (preparation see section 6) in ddH_2O at 55 °C for 60 min for iEDDA click reaction. The reaction was stopped with 2 x denaturing loading buffer and the sample was loaded onto a 6 % polyacrylamide gel. After running for 25 min at 200 V, the gel was stained with SYBR safe for 5 minutes and visualized with an ChemoStar Touch imager (*INTAS*). The amount of shifted RNA was quantified using LabImage 1D software v4.2.3 (*Kapelan*).

To determine UB retention, eleven different mixtures of rTPT3^{CP}-modified SPINACH2-RNA and fully canonical SPINACH2-RNA were prepared in the following ratios for calibration: 0:100, 6.25:93.75, 12.5:87.5, 25:75, 37.5:62.5, 50:50, 75:25, 88:12, 94:6, 97:3, 100:0. The samples were incubated with tetrazine-modified lysozyme and were analyzed on a 6 % denaturing polyacrylamide gel as described above. Each experiment was run fifth times. The iEDDA shift was plotted as function of the rTPT3^{CP} content in RNA and the resulting data was fitted to a linear equation (R^2 = 0.992):

$$Shift\ [\%] = -0.121 + 0.595x(TPT3 - RNA)$$

A representative gel and the resulting calibration line are shown in the supplementary section. Applying this equation, the retention of the UBP was calculated from the ratios of shifted RNAs after the SELEX cycle.

2. Quantification of full-length cDNA via Polyacrylamide Gel Electrophoresis

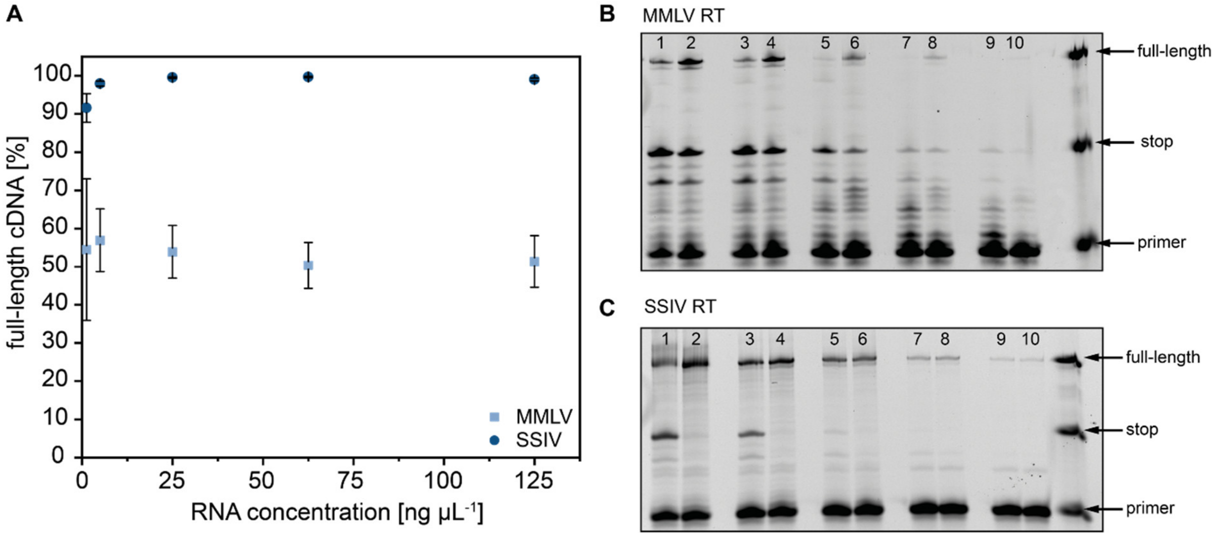

Supplementary Figure 1. Reverse transcription of 80-nt rTPT3-RNA with SS IV and MMLV RT with different RNA concentrations. A) Observed relative full-length cDNA intensities of reverse transcriptions w/ dNaM TP plotted against the RNA concentration. B) Reverse transcription of 80-nt rTPT3-RNA with MMLV RT. C) Reverse Transcription of 80-nt rTPT3-RNA with SS IV RT. 1: 125 ng μL^{-1} RNA w/o dNaM TP, 2: 125 ng μL^{-1} RNA w/ dNaM TP, 3: 62.5 ng μL^{-1} RNA w/o dNaM TP, 4: 62.5 ng μL^{-1} RNA w/ dNaM TP, 5: 25 ng μL^{-1} RNA w/o dNaM TP, 6: 25 ng μL^{-1} w/ dNaM TP, 7: 5 ng μL^{-1} RNA w/o dNaM TP, 8: 5 ng μL^{-1} RNA w/ dNaM TP, 9: 1.25 ng μL^{-1} RNA w/o dNaM TP, 10: 1.25 ng μL^{-1} RNA w/ dNaM TP.

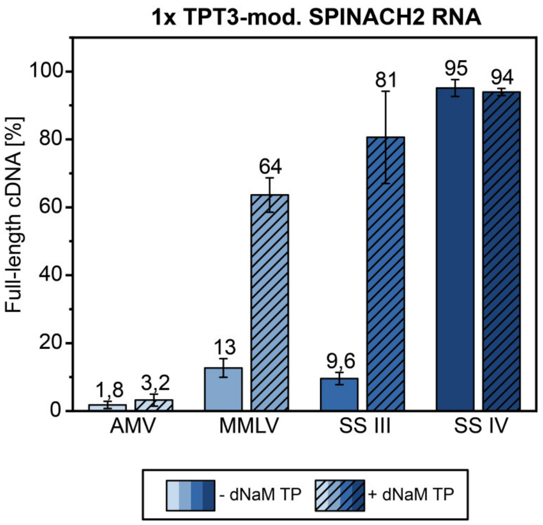

Supplementary Figure 2. Relative full-length cDNA intensities of reverse transcriptions of rTPT3-SPINACH2-RNA with AMV, MMLV, SS III and SS IV RT with and without dNaM TP. n = 3.

Supplementary Table 1. Relative full-length band intensities of reverse transcriptions from 48-nt 1xrTPT3-RNA with and without dNaM TP.

	Without dNaM TP				With dNaM TP			
**RT**	**AMV**	**MMLV**	**SS III**	**SS IV**	**AMV**	**MMLV**	**SS III**	**SS IV**
**Full length [%]**	0.00	34.59	2.06	54.41	7.65	80.04	81.29	93.36
	0.28	35.92	1.59	52.17	17.23	74.44	78.32	93.76
	2.62	16.79	3.78	24.98	10.22	84.99	64.35	90.25
	1.68	17.87	5.80	45.80	18.45	83.48	62.46	90.88
	1.24	32.36	8.90	49.76	8.16	78.37	54.45	92.85
	0.25	31.73	0.39	48.31	10.35	78.33	60.68	92.48
	0.85	35.97	1.49	49.95	20.24	81.84	62.19	93.63
	0.84	27.44	4.78	50.07	21.41	82.19	70.39	92.85
	0.52	27.08	4.33	49.79	16.02	86.97	65.19	92.28
	0.14	27.05	-	-	18.29	87.30	-	-
**Mean**	0.97	29.88	3.68	52.75	14.07	81.61	66.59	92.48
**SD**	0.27	5.76	2.49	8.18	5.25	4.06	8.12	1.13

Supplementary Table 2. Relative full-length band intensities of reverse transcriptions from 48-nt 2xrTPT3-RNA with and without dNaM TP.

	Without dNaM TP				With dNaM TP			
**RT**	**AMV**	**MMLV**	**SS III**	**SS IV**	**AMV**	**MMLV**	**SS III**	**SS IV**
**Full length [%]**	1.76	0.53	0.13	0.18	0.00	23.81	2.19	0.00
	1.00	0.78	0.05	0.14	0.63	18.82	2.06	0.72
	1.67	2.09	0.09	0.10	0.99	8.79	1.44	11.15
	0.86	2.65	0.00	0.00	0.29	10.21	0.07	0.22
	0.41	2.39	0.24	0.86	0.38	16.57	0.58	1.68
	0.65	2.27	0.17	2.16	0.18	15.87	0.35	14.01
	0.88	2.39	0.15	2.53	0.83	23.01	1.02	17.35
	1.09	2.70	0.40	0.83	1.10	24.72	3.67	3.55
	0.85	5.31	0.43	0.65	0.54	26.31	3.20	3.03
	1.34	5.52	-	-	1.05	22.09	-	-
**Mean**	1.08	2.67	0.18	0.83	0.63	20.00	1.62	5.75
**SD**	0.45	1.63	0.14	0.87	0.39	5.32	1.19	6.23

Supplementary Table 3. Relative full-length band intensities of reverse transcriptions from 48-nt 1xrNaM-RNA with and without dTPT3 TP.

	Without dTPT3 TP				With dTPT3 TP			
**RT**	**AMV**	**MMLV**	**SS III**	**SS IV**	**AMV**	**MMLV**	**SS III**	**SS IV**
**Full length [%]**	1.69	21.38	4.36	37.02	39.95	95.63	56.87	90.30
	1.19	29.33	6.28	39.82	27.68	93.69	57.39	95.02
	0.62	27.66	6.62	49.51	28.84	87.63	50.83	97.65
	1.76	25.44	4.82	48.81	39.64	92.80	48.98	97.69
	0.86	39.62	1.55	42.61	35.26	94.07	70.13	86.24
	0.80	31.08	1.64	44.72	35.97	95.19	70.97	87.05
**Mean**	1.15	29.08	4.21	43.76	34.56	93.17	59.20	92.32
**SD**	0.44	5.62	2.01	4.49	4.78	2.65	8.58	4.71

Supplementary Table 4. Relative full-length band intensities of reverse transcriptions from 48-nt 2xrNaM-RNA with and without dTPT3 TP.

	Without dTPT3 TP				With dTPT3 TP			
**RT**	**AMV**	**MMLV**	**SS III**	**SS IV**	**AMV**	**MMLV**	**SS III**	**SS IV**
**Full length [%]**	0.54	0.75	0.06	9.63	2.65	76.89	20.19	91.81
	2.02	10.03	0.08	9.27	0.50	72.73	15.09	96.71
	2.92	10.01	0.05	8.30	5.35	73.58	14.23	91.84
	1.17	1.26	0.06	4.16	1.87	76.09	19.80	91.99
	1.88	4.54	0.10	1.69	1.70	90.13	33.82	93.13
	1.13	4.43	0.15	4.46	7.28	83.22	34.91	93.57
**Mean**	1.61	5.17	0.08	6.25	3.22	78.77	23.01	93.17
**SD**	0.77	3.72	0.03	2.97	2.34	6.09	8.33	1.72

Supplementary Table 5. Relative full-length band intensities of reverse transcriptions from rTPT3-SPINACH2 RNA with and without dNaM TP.

	Without dNaM TP				With dNaM TP			
**RT**	**AMV**	**MMLV**	**SS III**	**SS IV**	**AMV**	**MMLV**	**SS III**	**SS IV**
**Full length [%]**	1.72	12.44	9.22	95.31	2.72	69.04	60.07	92.22
	1.15	9.92	9.62	97.12	1.78	65.26	62.97	93.82
	2.24	10.56	11.93	97.46	1.75	58.98	92.15	95.10
	3.89	17.44	6.25	91.77	6.99	55.37	91.18	93.69
	0.89	15.24	9.11	91.70	2.94	69.21	88.89	95.42
	0.88	10.58	11.22	97.29	3.10	63.90	88.41	93.49
**Mean**	1.79	12.70	9.56	95.11	3.21	63.63	80.61	93.96
**SD**	1.05	2.76	1.81	2.49	1.77	5.05	13.59	1.06

Supplementary Table 6. Relative full-length band intensities of reverse transcriptions of 80-nt rTPT3-RNA using SS IV RT with and without dNaM TP.

	Without dNaM TP					With dNaM TP				
**c [ng µL^{-1}]**	**125**	**62.5**	**25**	**5**	**1.25**	**125**	**62.5**	**25**	**5**	**1.25**
**Full length [%]**	43.08	69.90	96.51	98.01	92.21	99.24	99.66	96.56	97.29	94.90
	76.23	68.98	96.31	98.32	91.15	98.83	99.26	99.44	98.17	91.30
	77.16	65.91	95.09	98.03	95.19	99.09	99.71	99.31	96.94	95.10
	40.74	69.39	96.12	96.89	93.21	98.35	99.66	99.72	98.88	94.16
	80.91	77.71	98.17	94.79	94.94	99.68	99.72	99.46	98.38	84.66
	79.20	78.58	95.37	97.27	94.15	98.95	99.90	99.78	97.93	89.17
**Mean**	66.22	71.75	96.26	97.22	93.47	99.02	99.65	99.55	97.93	91.55
**SD**	17.27	4.71	0.99	1.19	1.45	0.40	0.19	0.17	0.65	3.73

Supplementary Table 7. Relative full-length band intensities of reverse transcriptions of 80-nt rTPT3-RNA using MMLV RT with and without dNaM TP.

c [ng µL^{-1}]	**Without dNaM TP**					**With dNaM TP**				
	**125**	**62.5**	**25**	**5**	**1.25**	**125**	**62.5**	**25**	**5**	**1.25**
**Full length [%]**	24.90	24.38	16.32	24.41	16.12	44.54	42.13	41.30	43.46	22.95
	22.15	18.93	16.31	6.43	47.10	44.18	41.89	42.80	49.48	57.44
	10.70	5.93	6.73	5.78	2.91	46.73	43.77	60.94	61.63	52.33
	10.51	7.94	5.03	7.73	6.06	48.34	47.25	60.38	60.92	53.60
	16.33	13.74	4.57	3.94	26.41	60.92	55.82	52.47	51.93	57.50
	13.85	12.10	4.92	5.66	11.05	60.09	56.14	57.71	54.78	36.54
	9.83	17.36	6.26	2.26	20.00	50.38	55.48	58.32	66.67	93.24
	9.06	16.87	6.14	2.52	11.32	46.21	54.56	58.50	70.89	69.15
**Mean**	14.67	14.66	8.28	7.85	17.62	50.17	49.63	54.05	57.47	55.34
**SD**	5.61	5.63	4.69	6.71	13.17	6.25	6.08	7.34	8.60	19.53

3. Kinetic measurements

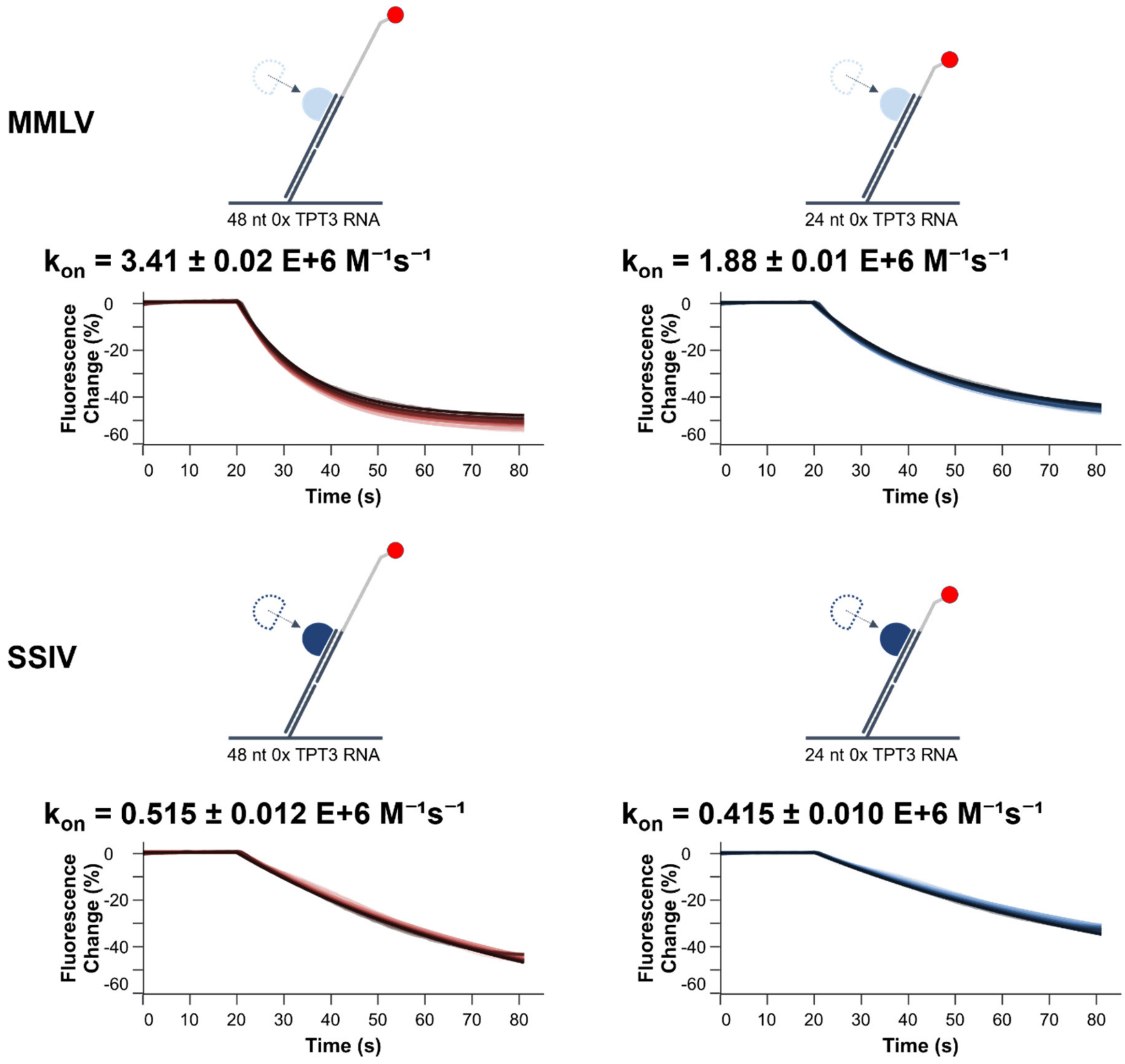

Supplementary Figure 3. Association curves of the RTs binding to the substrate. As example one dataset is show per RT with the 48 nt 0x rTPT3 RNA template that was immobilized on spot 1 and the 24 nt 0x rTPT3 RNA template on spot 2. The k_{on} represents the association rate under the assumption that k_{off}= 0, as described in the data processing.

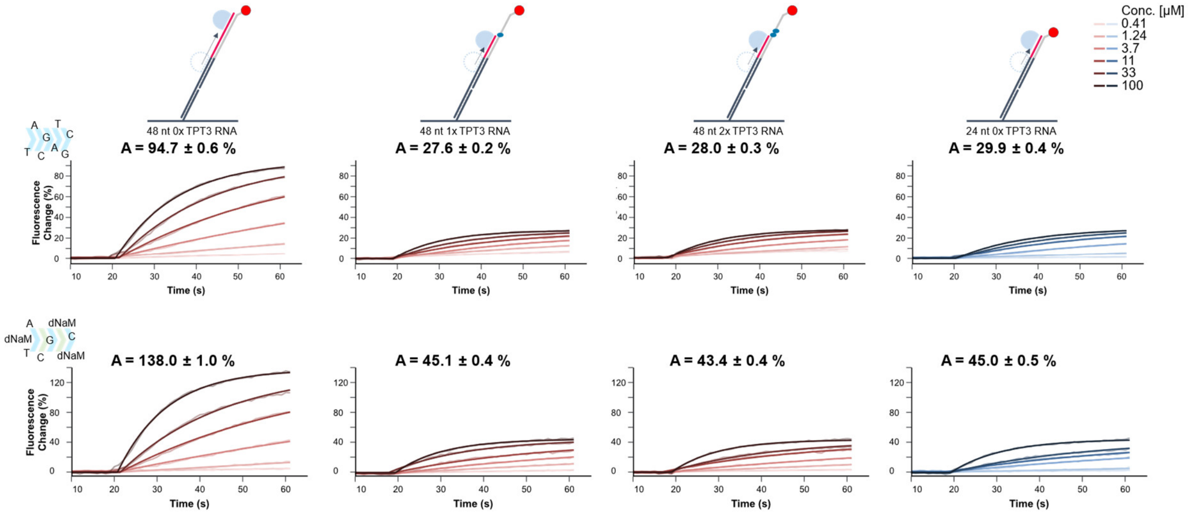

Supplementary Figure 4. Overview of all the real-time activities of MMLV RT with all different templates in the presence of dNTPs with or without dNaM. One dataset of the repetitions is shown. The fluorescence amplitude [A] was determined with a global fit model as described in the data processing.

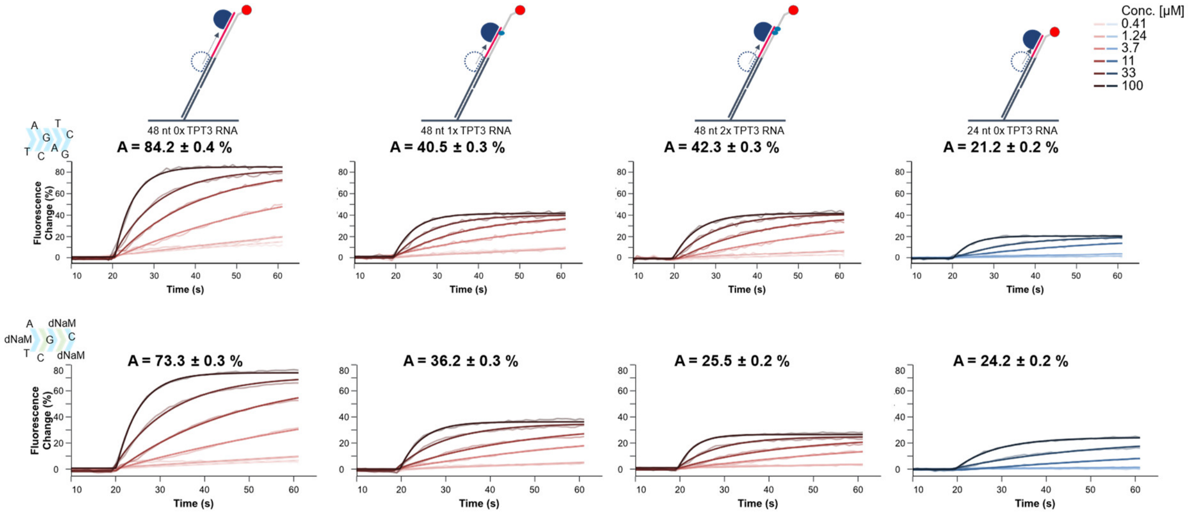

Supplementary Figure 5. Overview of all the real-time activities of SS IV RT with all different templates in the presence of dNTPs with or without dNaM. One dataset of the repetitions is shown. The fluorescence amplitude [A] was determined with a global fit model as described in the data processing.

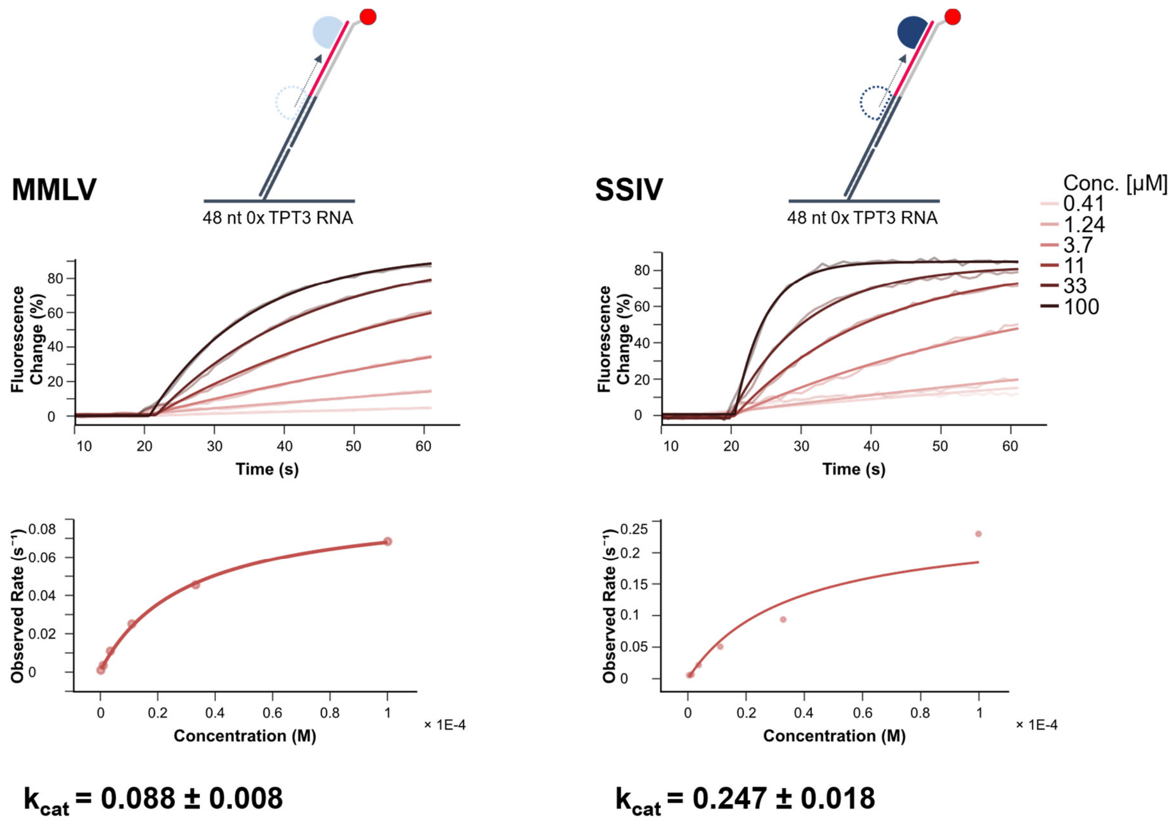

Supplementary Figure 6. Overview of the real-time activities of MMLV and SS IV RT for 48-nt unmodified RNA in presence of dNTPs. and observed k_{cat}. The observed rate was plotted over the concentration of dNTPs. The k_{cat} was calculated by Michaelis-Menten-kinetics as explained in the data processing above from n=4 measurements including the standard error of the mean.

4. LC-MS data

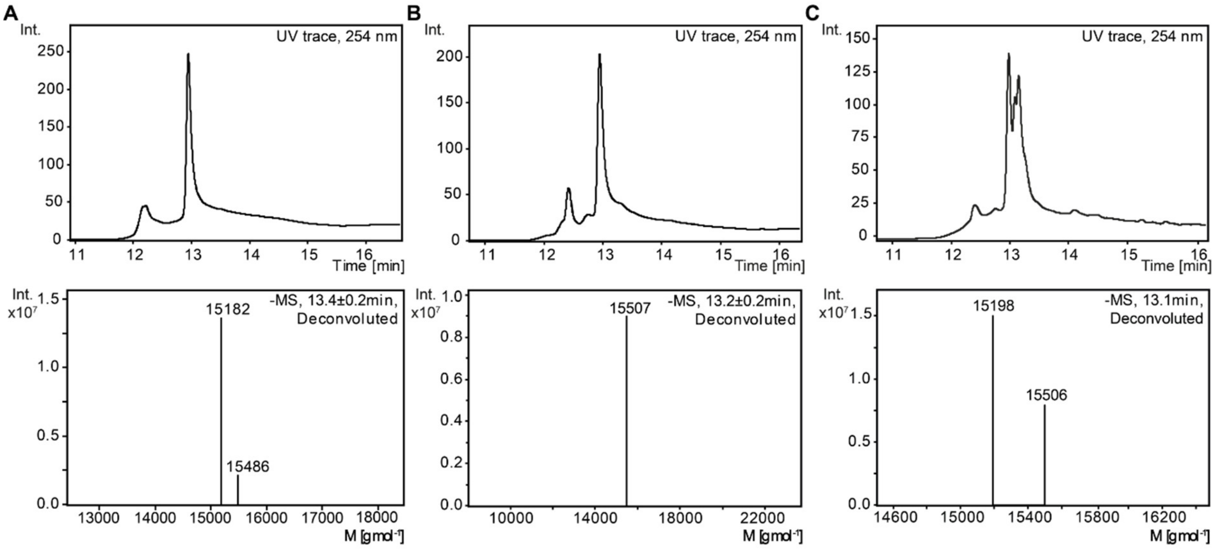

5.

6. **Supplementary Figure 7.** HPLC-ESI-MS measurements of reverse transcriptions of 48-nt-1xrNaM-RNA with SS IV RT. A) unmodified 48-nt RNA. B) 48-nt 1xNaM-RNA w/o dTPT3 TP. C) 48-nt 1xNaM-RNA w/ dTPT3 TP.

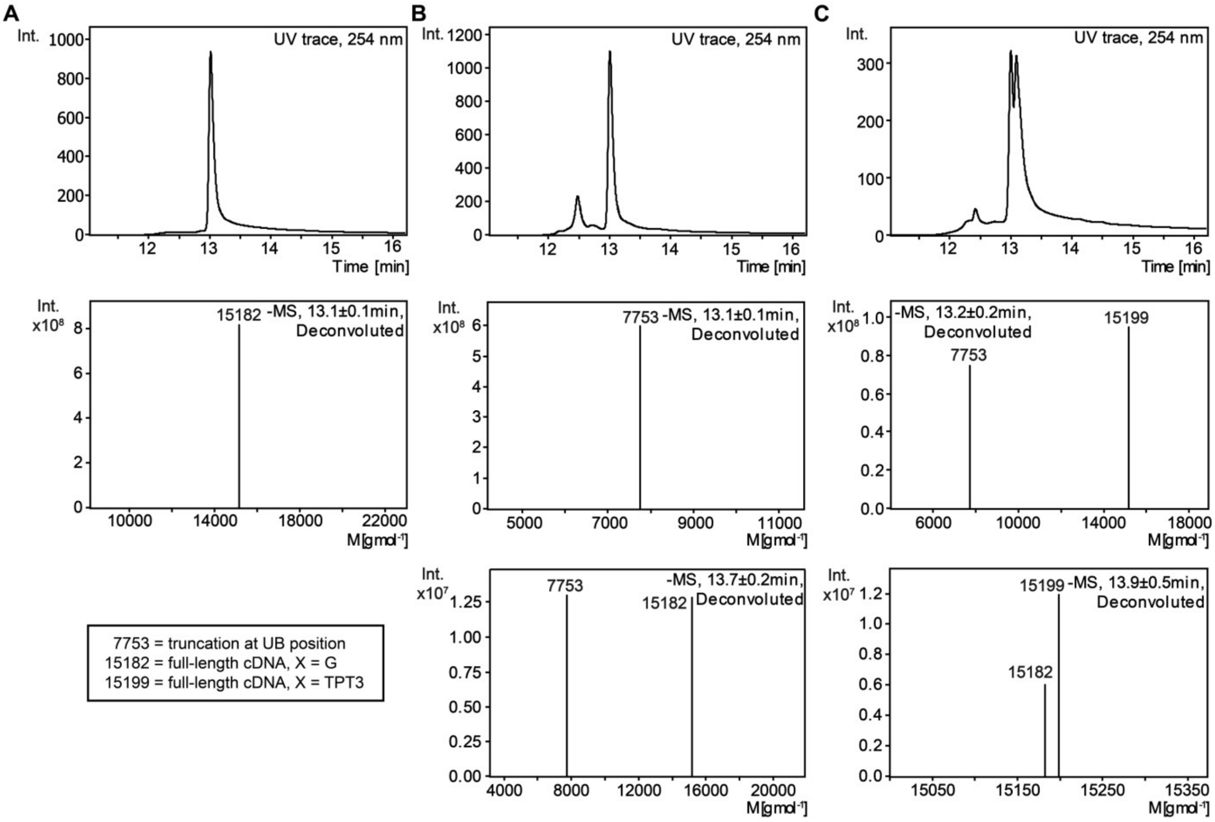

Supplementary Figure 8. HPLC-ESI-MS measurements of reverse transcriptions of 48nt RNA with MMLV RT. A) unmodified 48-nt RNA. B) 48-nt 1xNaM-RNA w/o dTPT3 TP. C) 48-nt 1xNaM-RNA w/ dTPT3 TP.

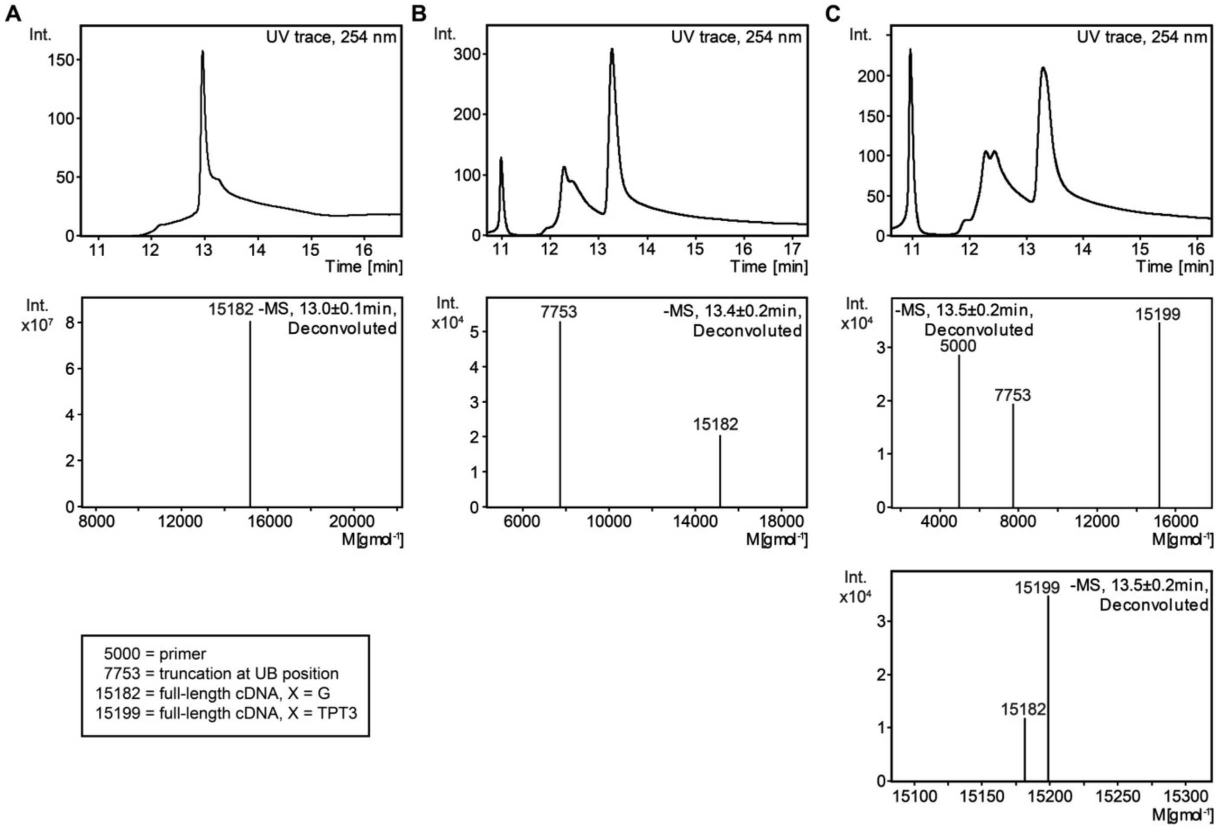

Supplementary Figure 9. HPLC-ESI-MS measurements of reverse transcriptions of 48-nt-1xrNaM-RNA with SS III RT. A) unmodified 48-nt RNA. B) 48-nt 1xNaM-RNA w/o dTPT3 TP. C) 48-nt 1xNaM-RNA w/ dTPT3 TP.

5. Sanger Sequencing

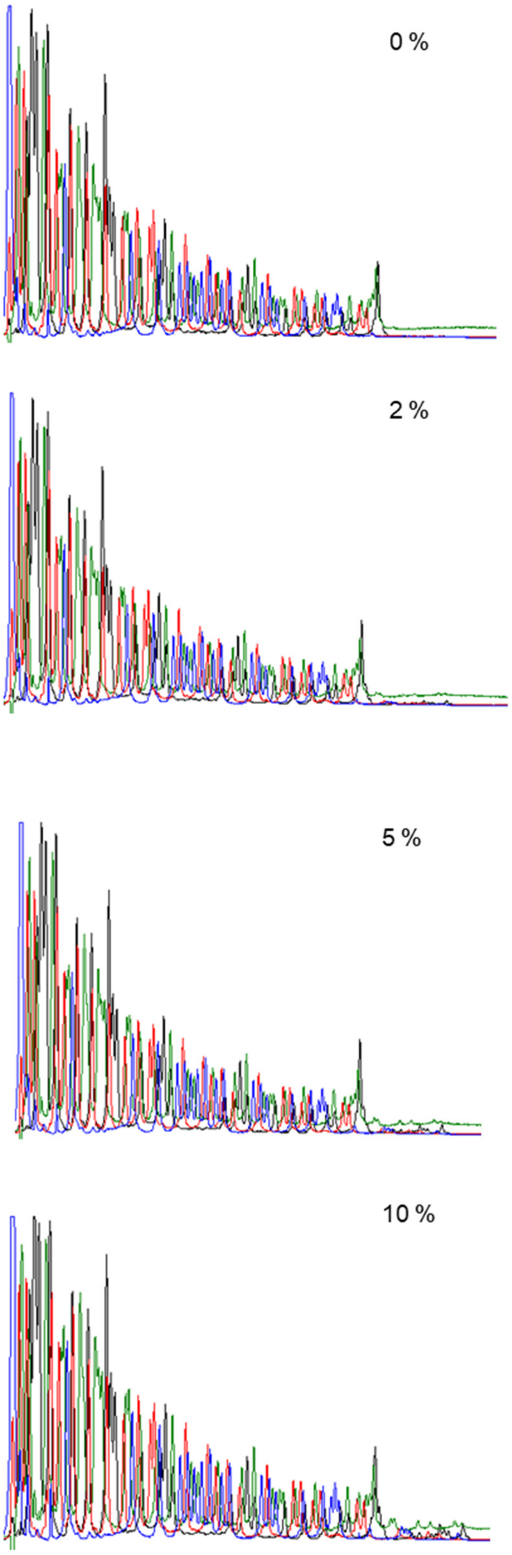

Supplementary Figure 10. Raw Sequencing data for different ratios of dNaM-modified DNA template and unmodified DNA template.

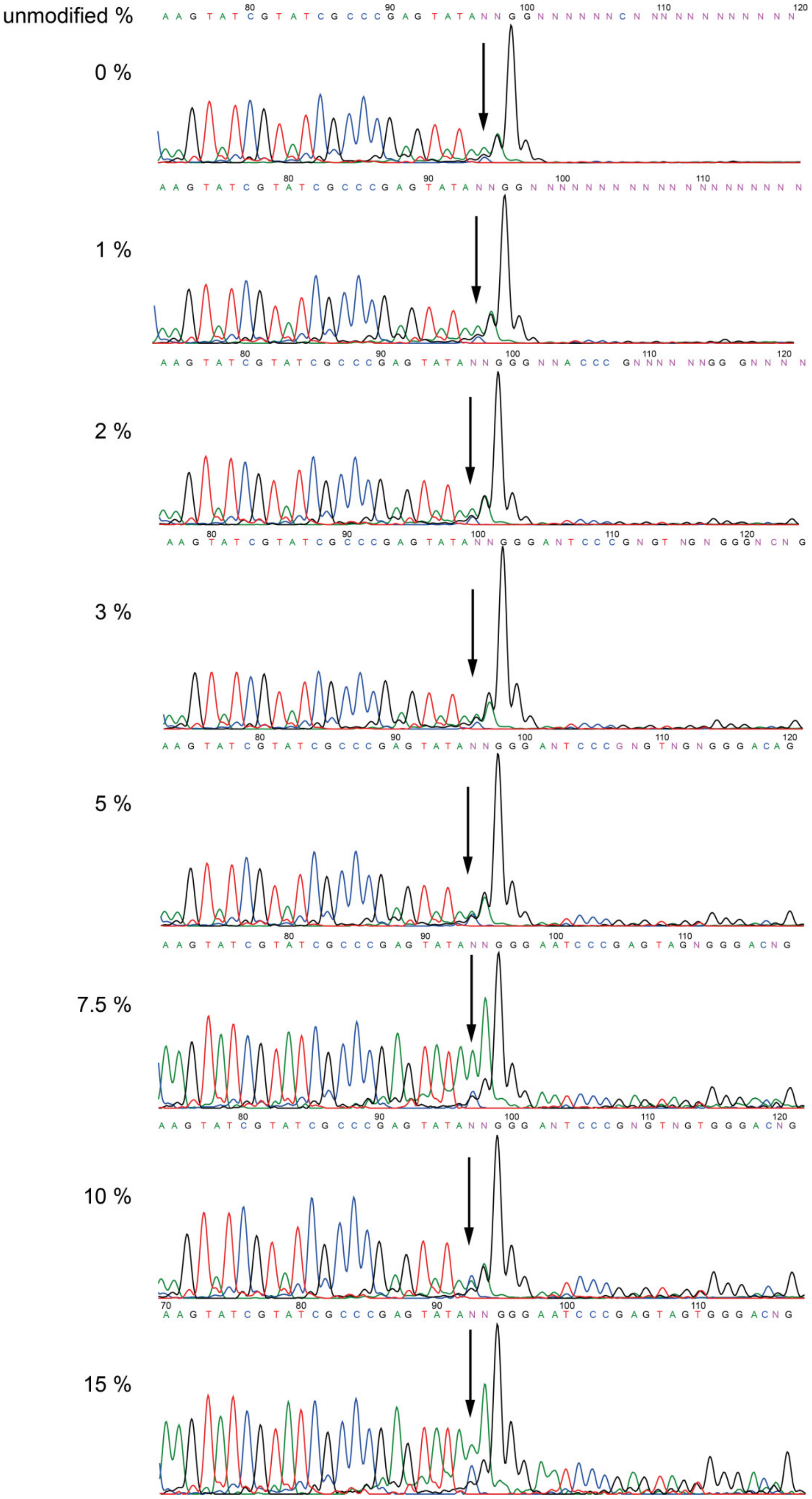

Supplementary Figure 11. Sequencing chromatograms of different ratios of dNaM-modified and unmodified DNA templates. The arrow indicates the position of dNaM in the sequence.

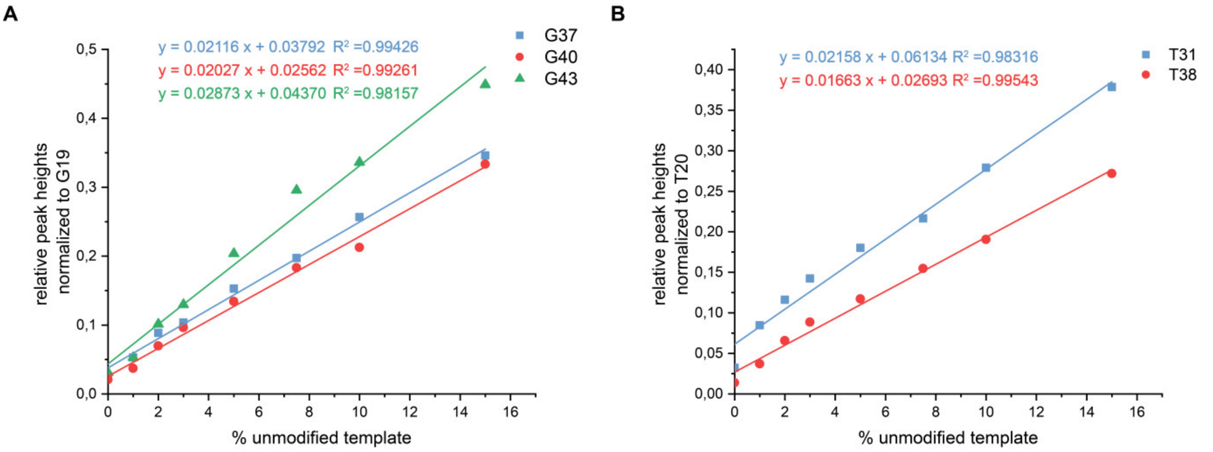

Supplementary Figure 12. Calibration line used for the calculation of the fidelity as described in literature [11, 12]. The peak heights of G19, T20, T31, G37, T38, G40 and G43 were determined with Sequence Scanner V2.0 (*Applied Biosystems*). The relative peak heights for G37, G40 and G43 were normalized to that the height of G19, peak heights for T31 and T8 were normalized to T20. The graphs were plotted, and the calibration line was generated applying a linear fit using Origin 2023.

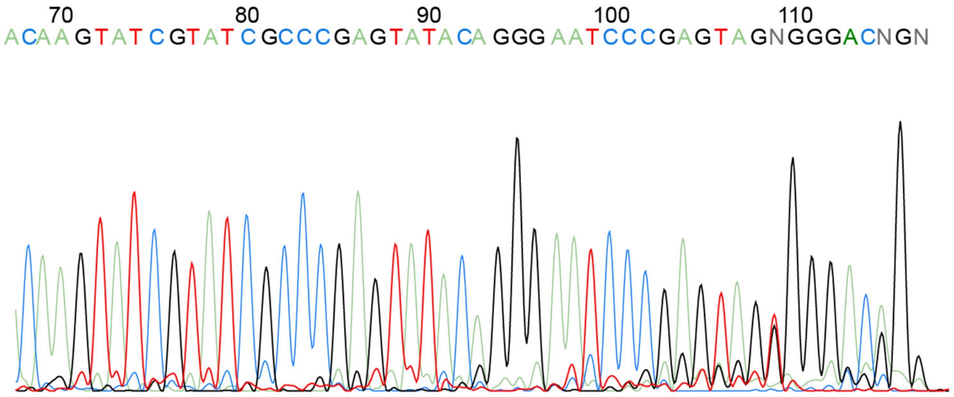

Supplementary Figure 13. Sequencing chromatogram for unmodified 48-nt cDNA.

6. SELEX cycle: iEDDA shift assay and AMV RT reverse transcription to determine UB retention

Modification of Lysozyme with Tetrazine-NHS-Ester

Lysozyme was modified with NHS-Ester PEG5 Tetrazine (*Sigma Aldrich*) according to literature [14]. Lysozyme was dissolved in PBS buffer (pH 7.4) containing 1 M NaHCO3 (v/v) to a concentration of 1 mg mL^{-1}. 25 eq. of NHS-ester PEG5 Tetrazine were added to the lysozyme and the reaction was incubated for 3 hours at room temperature and 300 rpm. Excess NHS-ester PEG5 Tetrazine was removed with Zeba spin desalting columns (7k MWKO, Thermo Scientific) using PBS buffer (ph 7.4) according to manufacturer's protocol. The concentration of the Tetrazine-modified lysozyme was determined by measuring the absorbance at 280 nm using a spectrophotometer DS-11 FX+ (*DeNovix*).

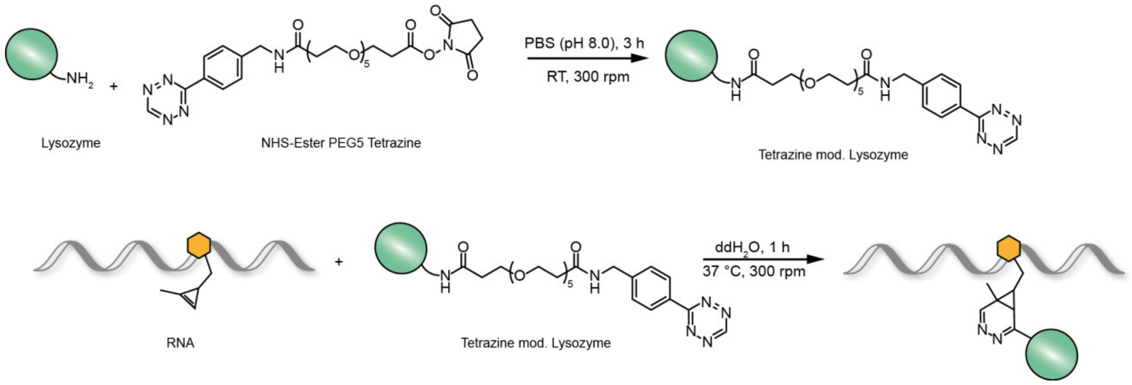

Supplementary Figure 14. Reaction scheme for the preparation of tetrazine modified lysozyme (above). Lysozyme is functionalized with NHS-ester PEG5 tetrazine via NHS ester chemistry. Modified lysozyme is then used to label $rTPT3^{CP}$-modified RNA via iEDDA click reaction resulting in a mobility shift of RNA during PAGE (below).

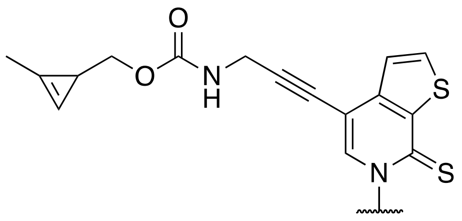

Supplementary Figure 15. Chemical structure of **$TPT3^{CP}$** [2].

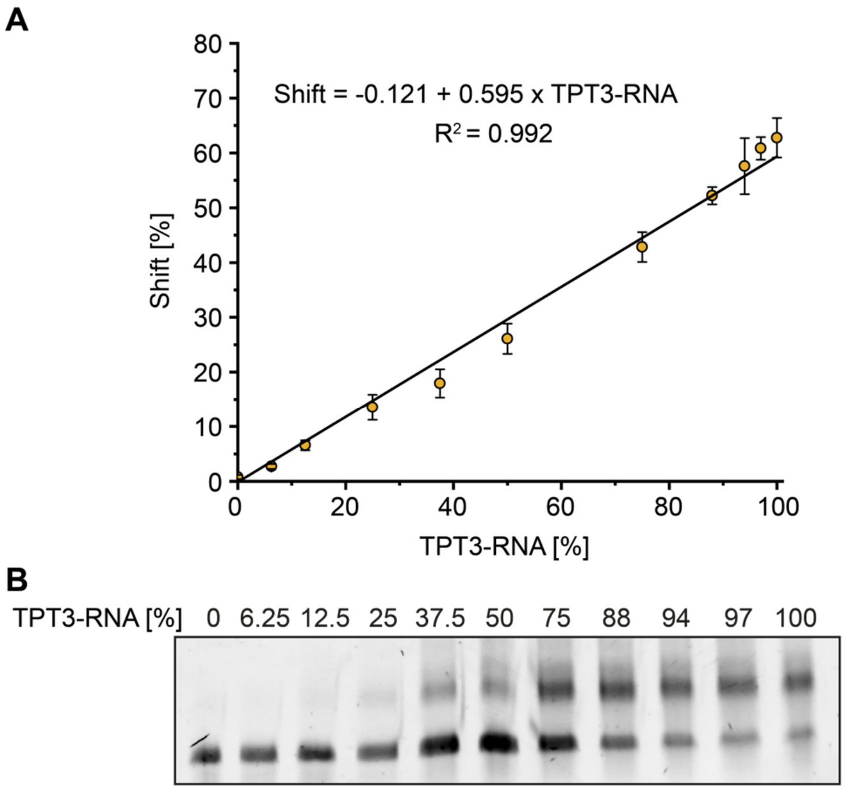

Supplementary Figure 16. A) Calibration line of the shift assay. The shift is plotted as a function of RNA containing rTPT3. The calibration line was generated by a linear fit model using Origin 2023. B) Representative polyacrylamide gel loaded with different rTPT3-RNA:unmodified RNA ratios which were incubated with tetrazine-modified lysozyme. n = 5.

Supplementary Table 8. rTPT3 retention values for rTPT3-SPINACH2-RNA after SELEX cycles using MMLV, SS III and SS IV RT. rTPT3 retention was calculated with the calibration line of the shift assay using the amount of shifted rTPT3^{CP}-SPINACH-RNA after iEDDA shift assay.

	**MMLV**			**SS III**			**SS IV**		
**Cycle**	**1**	**2**	**3**	**1**	**2**	**3**	**1**	**2**	**3**
**rTPT3 retention [%]**	80.41	63.78	43.93	88.08	71.61	38.99	87.91	83.37	38.01
	83.10	69.89	53.60	84.19	63.58	40.64	94.31	58.88	71.82
	79.27	71.52	47.64	71.67	64.99	45.44	83.66	75.73	77.73
**Mean**	80.93	68.40	48.39	81.31	66.73	41.69	88.63	72.67	62.52
**SD**	1.61	3.33	3.98	7.00	3.50	2.74	4.38	10.23	17.50

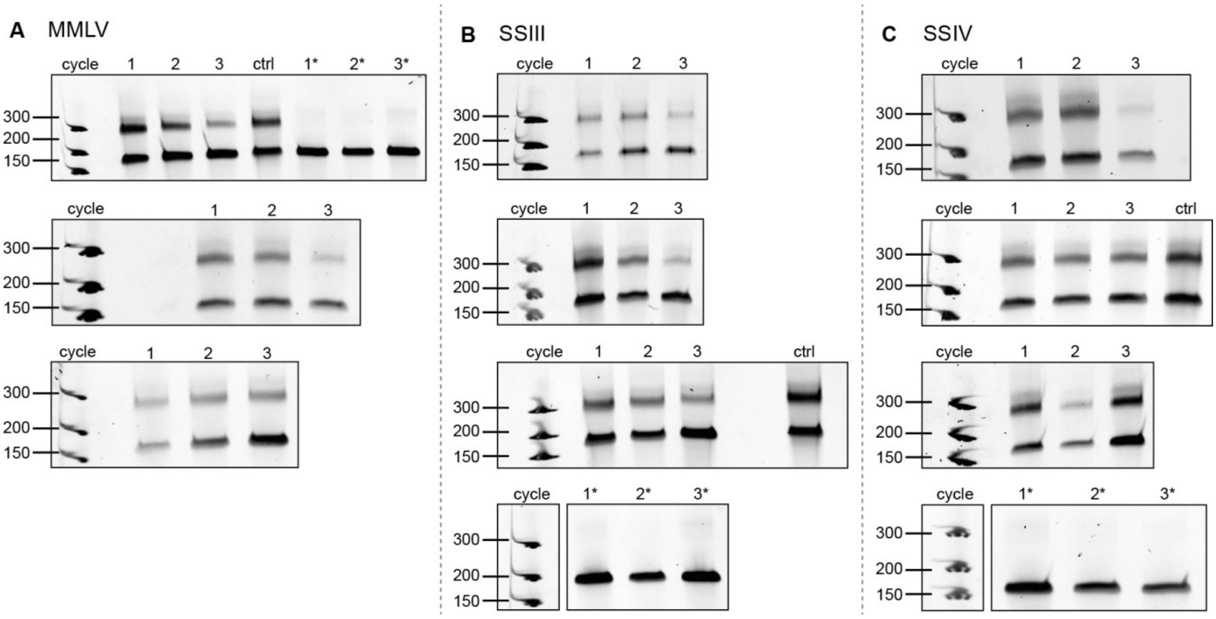

Supplementary Figure 17. 6 % Polyacrylamide gels of iEDDA shift assays with rTPT3^{CP}-SPINACH2-RNA after SELEX cycles 1-3. A) SELEX cycle with MMLV RT. B) SELEX cycle with SS III RT. C) SELEX cycle with SS IV RT. Ctrl: control with rTPT3^{CP}-SPINACH2-RNA transcribed from dNaM-SPINACH2 DNA template.*: SELEX cycle of unmodified DNA template and without addition of UB TP during reverse transcription.

Supplementary Table 9. Relative stop band intensities of reverse transcriptions with AMV RT without dNaM TP after SELEX cycles. Mean and standard deviation (SD) were normalized (normalized mean = Mean$^{norm.}$, normalized SD = SD$^{norm.}$) to AMV RT reverse transcriptions of chemical synthesized RNA based on the assumption that AMV RT quantitively stops at the rTPT3/rNaM-position in RNA if dNaM TP is absent in the reaction.

	MMLV				SS III				SS IV			
**Cycle**	**c**	**1**	**2**	**3**	**c**	**1**	**2**	**3**	**c**	**1**	**2**	**3**
**Stop [%]**	98.88	88.35	62.62	40.17	98.90	91.59	76.27	26.25	98.80	94.88	83.47	64.45
	99.04	89.56	74.13	46.61	99.45	91.95	76.13	36.52	98.66	94.39	74.41	56.15
	97.47	86.64	72.28	41.95	97.67	89.22	76.45	34.86	97.42	91.84	78.27	59.59
**Mean**	98.46	88.18	69.68	42.91	98.67	90.92	76.28	32.54	98.29	93.71	78.72	60.06
**SD**	0.70	1.20	5.05	2.71	0.75	1.21	0.13	4.50	0.62	1.33	3.71	3.41
**Mean$^{norm.}$**	99.42	89.04	70.36	43.33	99.64	91.81	77.02	32.85	99.25	94.63	79.49	60.64
**SD$^{norm.}$**	0.71	1.21	5.10	2.73	0.75	1.22	0.13	4.54	0.63	1.34	3.75	3.44

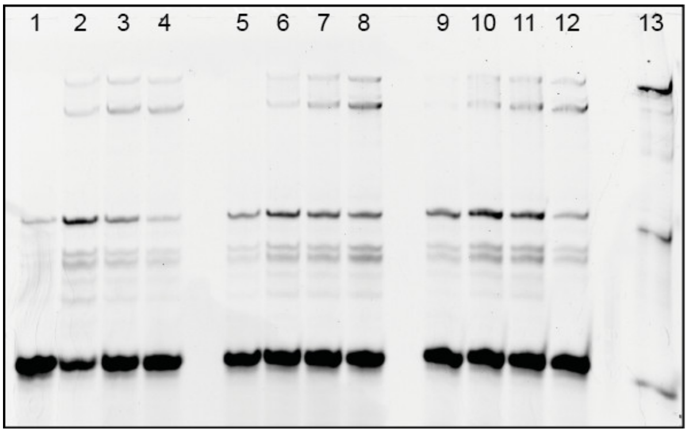

Supplementary Figure 18. 20 % sequencing gel of reverse transcription with AMV RT w/o dNaM TP of rTPT3-SPINACH2-RNA obtained from SELEX with MMLV RT. 1: RNA-TPT3_1, 2: RNA-TPT3_1 Round 1, 3: RNA-TPT3_1 Round 2, 4: RNA-TPT3_1 Round 3, 5: RNA-TPT3_2, 6: RNA-TPT3_2 Round 1, 7: RNA-TPT3_2 Round 2, 8: RNA-TPT3_2 Round 3, 9: RNA-TPT3_3, 10: RNA-TPT3_3 Round 1, 11: RNA-TPT3_3 Round 2, 12: RNA-TPT3_3 Round 3, 13: marker.

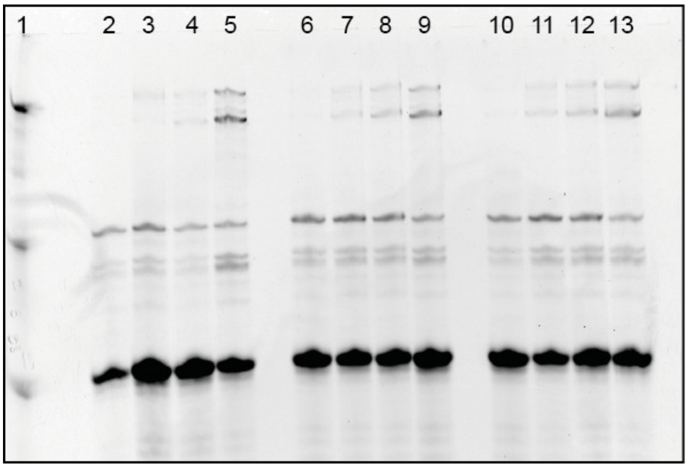

Supplementary Figure 19. 20 % sequencing gel of reverse transcription with AMV RT w/o dNaM TP of rTPT3-SPINACH2-RNA obtained from SELEX with SS III RT. 1: marker, 2: RNA-TPT3_1, 3: RNA-TPT3_1 Round 1, 4: RNA-TPT3_1 Round 2, 5: RNA-TPT3_1 Round 3, 6: RNA-TPT3_2, 7: RNA-TPT3_2 Round 1, 8: RNA-TPT3_2 Round 2, 9: RNA-TPT3_2 Round 3, 10: RNA-TPT3_3, 11: RNA-TPT3_3 Round 1, 12: RNA-TPT3_3 Round 2, 13: RNA-TPT3_3 Round 3.

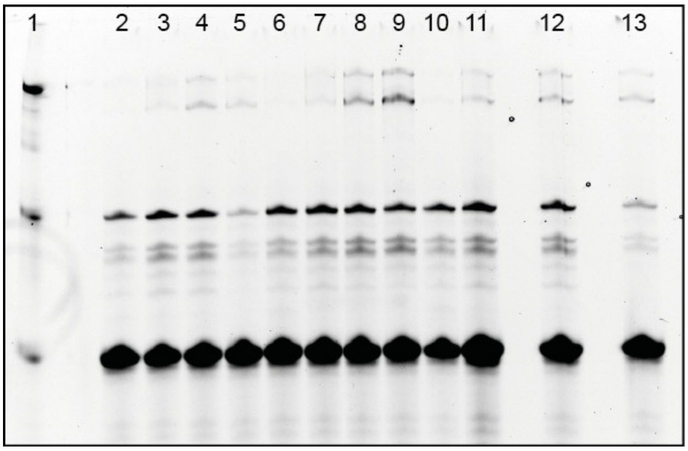

Supplementary Figure 20. 20 % sequencing gel of reverse transcription with AMV RT w/o dNaM TP of rTPT3-SPINACH2-RNA obtained from SELEX with SS IV RT. 1: marker, 2: RNA-TPT3_1, 3: RNA-TPT3_1 Round 1, 4: RNA-TPT3_1 Round 2, 5: RNA-TPT3_1 Round 3, 6: RNA-TPT3_2, 7: RNA-TPT3_2 Round 1, 8: RNA-TPT3_2 Round 2, 9: RNA-TPT3_2 Round 3, 10: RNA-TPT3_3, 11: RNA-TPT3_3 Round 1, 12: RNA-TPT3_3 Round 2, 13: RNA-TPT3_3 Round 3.

7. Sequencing gels used for data generation in this study:

48-nt TPT3-RNA:

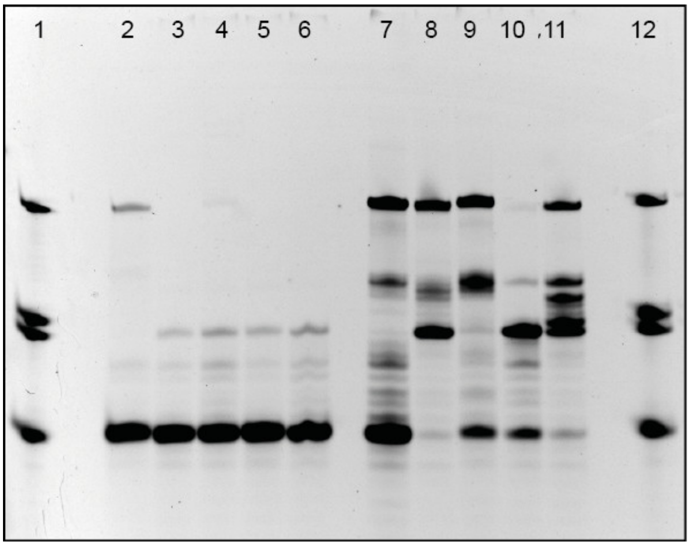

Supplementary Figure 21. 20 % sequencing gel of reverse transcription products of 48-nt RNA using AMV (2-6) and MMLV RT (8-12). 1: marker; 2: unmodified RNA; 3: 1xrTPT3-RNA w/o dNaM TP; 4: 1xrTPT3-RNA w/ dNaM TP; 5: 2xrTPT3-RNA w/o dNaM TP; 6: 2xrTPT3-RNA w/ dNaM TP; 7: unmodified RNA; 8: 1xrTPT3-RNA w/o dNaM TP; 9: 1xrTPT3-RNA w/ dNaM TP; 10: 2xrTPT3-RNA w/o dNaM TP; 11: 2xrTPT3-RNA w/ dNaM TP, 12: marker.

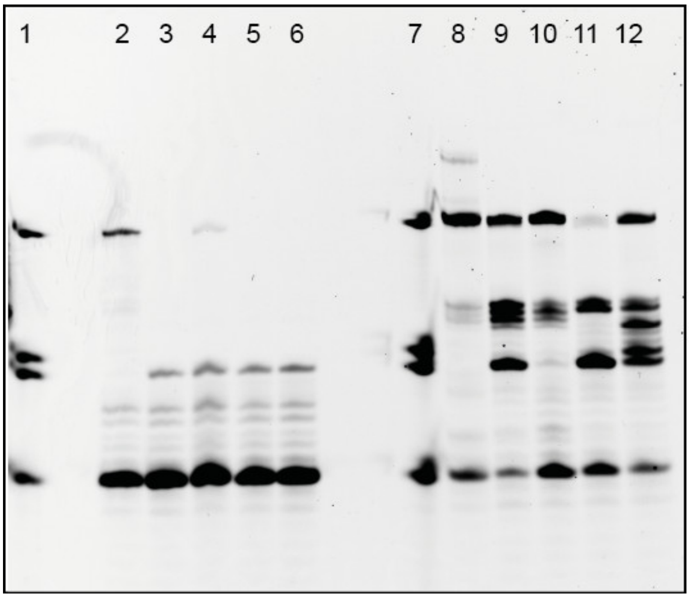

Supplementary Figure 22. 20 % sequencing gel of reverse transcription products of 48-nt RNA using AMV (2-6) and MMLV RT (8-12). 1: marker; 2: unmodified RNA; 3: 1xrTPT3-RNA w/o dNaM TP; 4: 1xrTPT3-RNA w/ dNaM TP; 5: 2xrTPT3-RNA w/o dNaM TP; 6: 2xrTPT3-RNA w/ dNaM TP; 7: marker; 8: unmodified RNA; 9: 1xrTPT3-RNA w/o dNaM TP; 10: 1xrTPT3-RNA w/ dNaM TP; 11: 2xrTPT3-RNA w/o dNaM TP; 12: 2xrTPT3-RNA w/ dNaM TP.

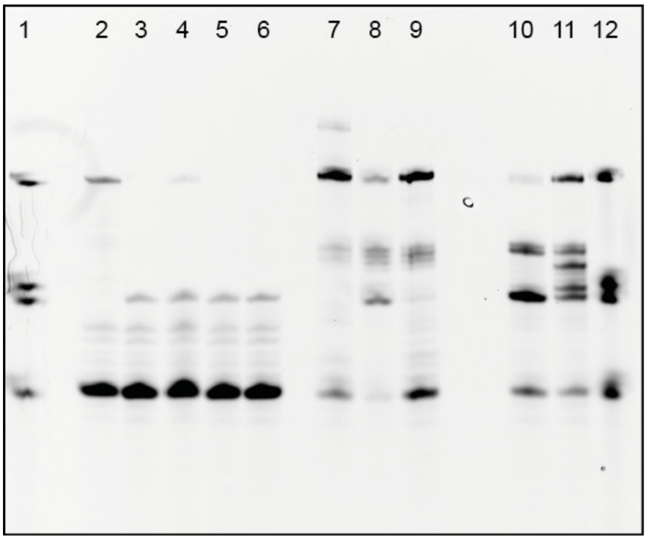

Supplementary Figure 23. 20 % sequencing gel of reverse transcription products of 48-nt RNA using AMV (2-6) and MMLV RT (7-11). 1: marker; 2: unmodified RNA; 3: 1xrTPT3-RNA w/o dNaM TP; 4: 1xrTPT3-RNA w/ dNaM TP; 5: 2xrTPT3-RNA w/o dNaM TP; 6: 2xrTPT3-RNA w/ dNaM TP; 7: unmodified RNA; 8: 1xrTPT3-RNA w/o dNaM TP; 9: 1xrTPT3-RNA w/ dNaM TP; 10: 2xrTPT3-RNA w/o dNaM TP; 11: 2xrTPT3-RNA w/ dNaM TP; 12: marker.

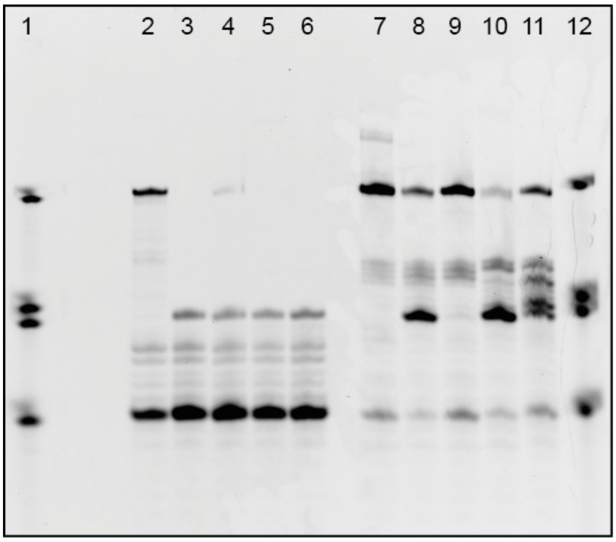

Supplementary Figure 24. 20 % sequencing gel of reverse transcription products of 48-nt RNA using AMV (2-6) and MMLV RT (7-11). 1: marker; 2: unmodified RNA; 3: 1xrTPT3-RNA w/o dNaM TP; 4: 1xrTPT3-RNA w/ dNaM TP; 5: 2xrTPT3-RNA w/o dNaM TP; 6: 2xrTPT3-RNA w/ dNaM TP; 7: unmodified RNA; 8: 1xrTPT3-RNA w/o dNaM TP; 9: 1xrTPT3-RNA w/ dNaM TP; 10: 2xrTPT3-RNA w/o dNaM TP; 11: 2xrTPT3-RNA w/ dNaM TP; 12: marker.

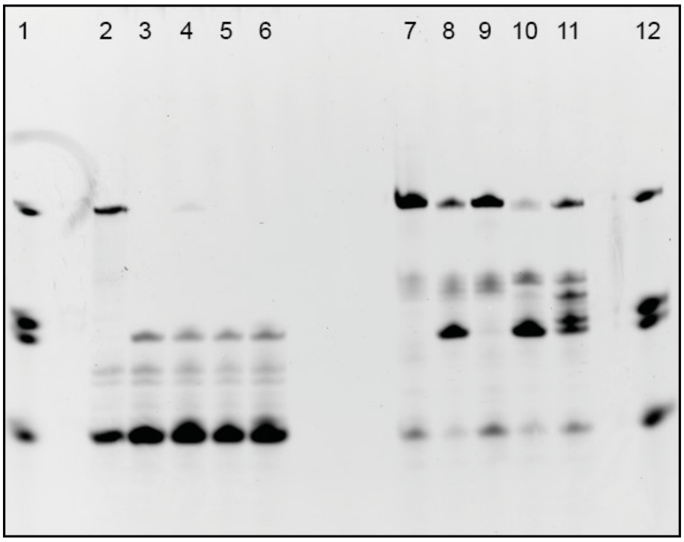

Supplementary Figure 25. 20 % sequencing gel of reverse transcription products of 48-nt RNA using AMV (2-6) and MMLV RT (7-11). 1: marker; 2: unmodified RNA; 3: 1xrTPT3-RNA w/o dNaM TP; 4: 1xrTPT3-RNA w/ dNaM TP; 5: 2xrTPT3-RNA w/o dNaM TP; 6: 2xrTPT3-RNA w/ dNaM TP; 7: unmodified RNA; 8: 1xrTPT3-RNA w/o dNaM TP; 9: 1xrTPT3-RNA w/ dNaM TP; 10: 2xrTPT3-RNA w/o dNaM TP; 11: 2xrTPT3-RNA w/ dNaM TP; 12: marker.

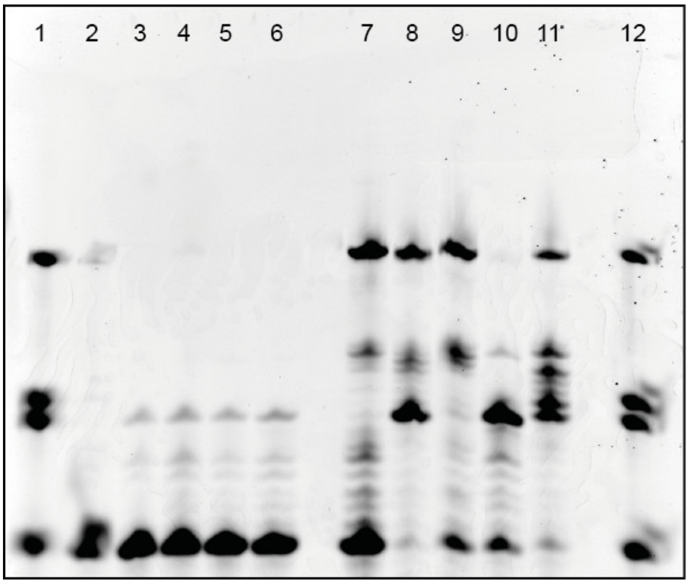

Supplementary Figure 26. 20 % sequencing gel of reverse transcription products of 48-nt RNA using AMV (2-6) and MMLV RT (7-11). 1: marker; 2: unmodified RNA; 3: 1xrTPT3-RNA w/o dNaM TP; 4: 1xrTPT3-RNA w/ dNaM TP; 5: 2xrTPT3-RNA w/o dNaM TP; 6: 2xrTPT3-RNA w/ dNaM TP; 7: unmodified RNA; 8: 1xrTPT3-RNA w/o dNaM TP; 9: 1xrTPT3-RNA w/ dNaM TP; 10: 2xrTPT3-RNA w/o dNaM TP; 11: 2xrTPT3-RNA w/ dNaM TP; 12: marker.

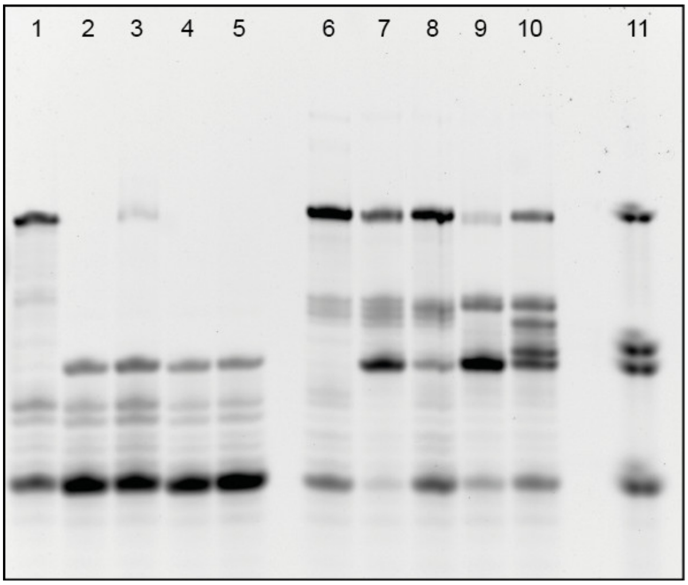

Supplementary Figure 27. 20 % sequencing gel of reverse transcription products of 48-nt RNA using AMV (2-6) and MMLV RT (7-11). 1: unmodified RNA; 2: 1xrTPT3-RNA w/o dNaM TP; 3: 1xrTPT3-RNA w/ dNaM TP; 4: 2xrTPT3-RNA w/o dNaM TP; 5: 2xrTPT3-RNA w/ dNaM TP; 6: unmodified RNA; 7: 1xrTPT3-RNA w/o dNaM TP; 8: 1xrTPT3-RNA w/ dNaM TP; 9: 2xrTPT3-RNA w/o dNaM TP; 10: 2xrTPT3-RNA w/ dNaM TP; 11: marker.

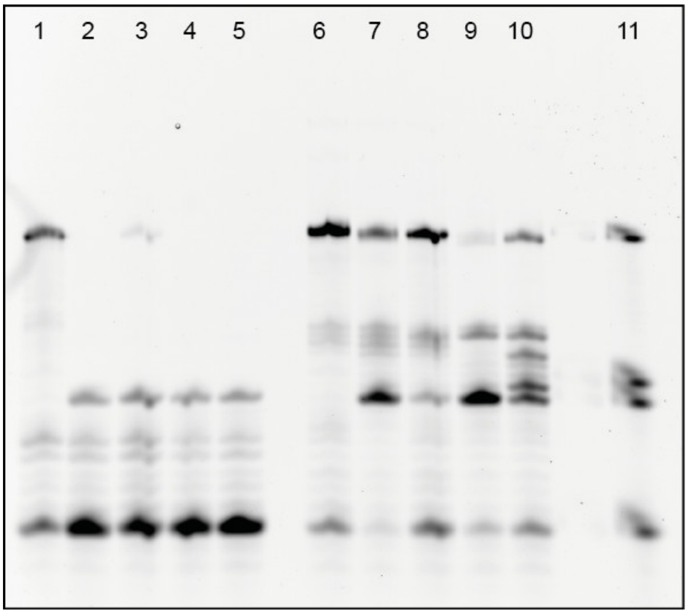

Supplementary Figure 28. 20 % sequencing gel of reverse transcription products of 48-nt RNA using AMV (2-6) and MMLV RT (7-11). 1: unmodified RNA; 2: 1xrTPT3-RNA w/o dNaM TP; 3: 1xrTPT3-RNA w/ dNaM TP; 4: 2xrTPT3-RNA w/o dNaM TP; 5: 2xrTPT3-RNA w/ dNaM TP; 6: unmodified RNA; 7: 1xrTPT3-RNA w/o dNaM TP; 8: 1xrTPT3-RNA w/ dNaM TP; 9: 2xrTPT3-RNA w/o dNaM TP; 10: 2xrTPT3-RNA w/ dNaM TP; 11: marker.

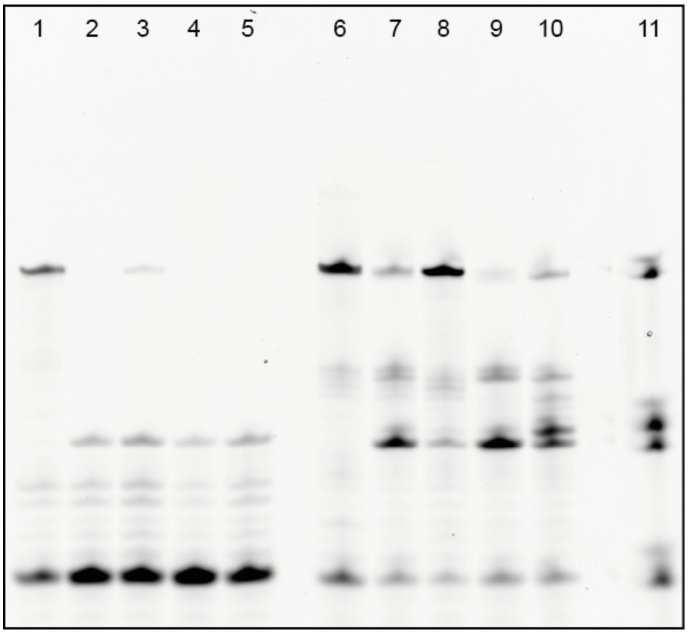

Supplementary Figure 29. 20 % sequencing gel of reverse transcription products of 48-nt RNA using AMV (2-6) and MMLV RT (7-11). 1: unmodified RNA; 2: 1xrTPT3-RNA w/o dNaM TP; 3: 1xrTPT3-RNA w/ dNaM TP; 4: 2xrTPT3-RNA w/o dNaM TP; 5: 2xrTPT3-RNA w/ dNaM TP; 6: unmodified RNA; 7: 1xrTPT3-RNA w/o dNaM TP; 8: 1xrTPT3-RNA w/ dNaM TP; 9: 2xrTPT3-RNA w/o dNaM TP; 10: 2xrTPT3-RNA w/ dNaM TP; 11: marker.

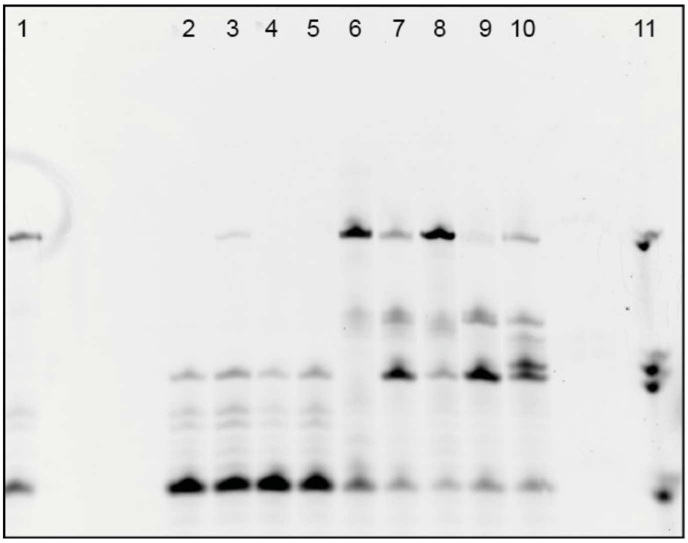

Supplementary Figure 30. 20 % sequencing gel of reverse transcription products of 48-nt RNA using AMV (2-6) and MMLV RT (7-11). 1: unmodified RNA; 2: 1xrTPT3-RNA w/o dNaM TP; 3: 1xrTPT3-RNA w/ dNaM TP; 4: 2xrTPT3-RNA w/o dNaM TP; 5: 2xrTPT3-RNA w/ dNaM TP; 6: unmodified RNA; 7: 1xrTPT3-RNA w/o dNaM TP; 8: 1xrTPT3-RNA w/ dNaM TP; 9: 2xrTPT3-RNA w/o dNaM TP; 10: 2xrTPT3-RNA w/ dNaM TP; 11: marker.

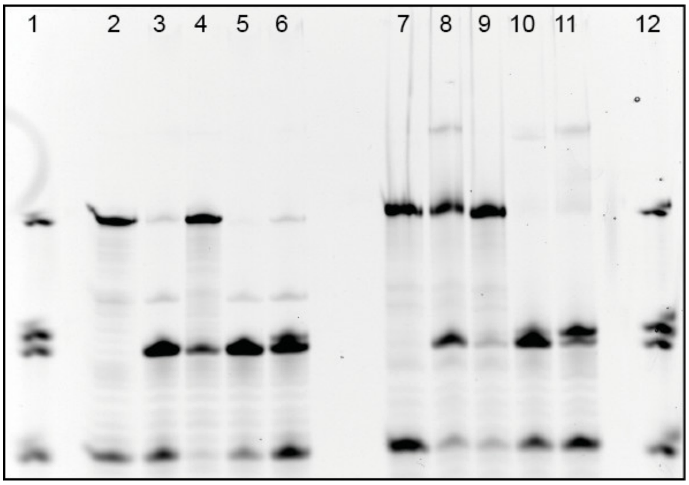

Supplementary Figure 31. 20 % sequencing gel of reverse transcription products of 48-nt RNA using SS III (2-6) and SS IV RT (8-12). 1: marker; 2: unmodified RNA; 3: 1xrTPT3-RNA w/o dNaM TP; 4: 1xrTPT3-RNA w/ dNaM TP; 5: 2xrTPT3-RNA w/o dNaM TP; 6: 2xrTPT3-RNA w/ dNaM TP; 7: unmodified RNA; 8: 1xrTPT3-RNA w/o dNaM TP; 9: 1xrTPT3-RNA w/ dNaM TP; 10: 2xrTPT3-RNA w/o dNaM TP; 11: 2xrTPT3-RNA w/ dNaM TP, 12: marker.

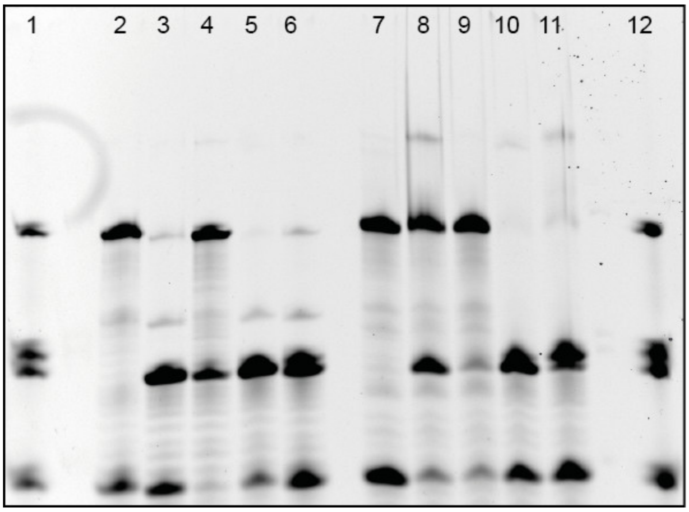

Supplementary Figure 32. 20 % sequencing gel of reverse transcription products of 48-nt RNA using SS III (2-6) and SS IV RT (8-12). 1: marker; 2: unmodified RNA; 3: 1xrTPT3-RNA w/o dNaM TP; 4: 1xrTPT3-RNA w/ dNaM TP; 5: 2xrTPT3-RNA w/o dNaM TP; 6: 2xrTPT3-RNA w/ dNaM TP; 7: unmodified RNA; 8: 1xrTPT3-RNA w/o dNaM TP; 9: 1xrTPT3-RNA w/ dNaM TP; 10: 2xrTPT3-RNA w/o dNaM TP; 11: 2xrTPT3-RNA w/ dNaM TP, 12: marker.

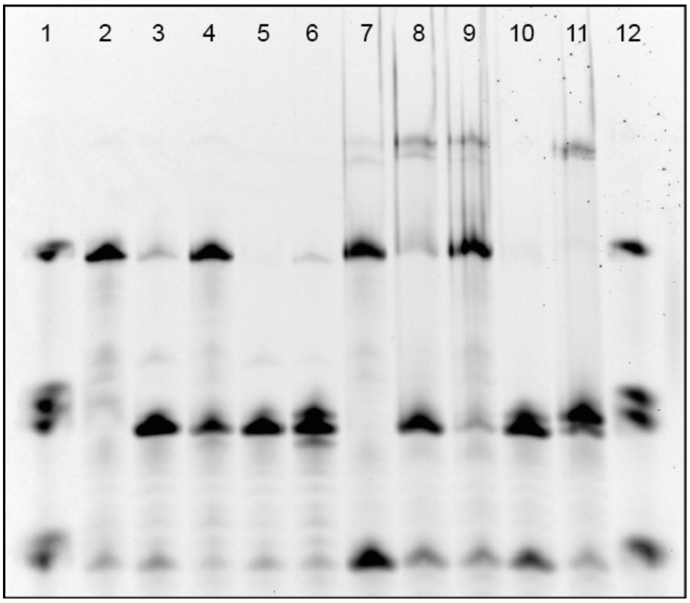

Supplementary Figure 33. 20 % sequencing gel of reverse transcription products of 48-nt RNA using SS III (2-6) and SS IV RT (8-12). 1: marker; 2: unmodified RNA; 3: 1xrTPT3-RNA w/o dNaM TP; 4: 1xrTPT3-RNA w/ dNaM TP; 5: 2xrTPT3-RNA w/o dNaM TP; 6: 2xrTPT3-RNA w/ dNaM TP; 7: unmodified RNA; 8: 1xrTPT3-RNA w/o dNaM TP; 9: 1xrTPT3-RNA w/ dNaM TP; 10: 2xrTPT3-RNA w/o dNaM TP; 11: 2xrTPT3-RNA w/ dNaM TP, 12: marker.

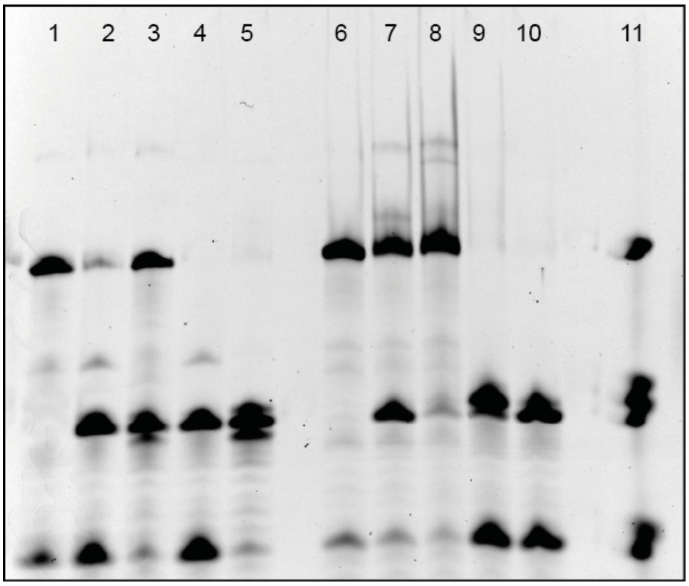

Supplementary Figure 34. 20 % sequencing gel of reverse transcription products of 48-nt RNA using SS III (2-6) and SS IV RT (8-12). 1: unmodified RNA; 2: 1xrTPT3-RNA w/o dNaM TP; 3: 1xrTPT3-RNA w/ dNaM TP; 4: 2xrTPT3-RNA w/o dNaM TP; 5: 2xrTPT3-RNA w/ dNaM TP; 6 : unmodified RNA; 7: 1xrTPT3-RNA w/o dNaM TP; 8: 1xrTPT3-RNA w/ dNaM TP; 9: 2xrTPT3-RNA w/o dNaM TP; 10: 2xrTPT3-RNA w/ dNaM TP, 11: marker.

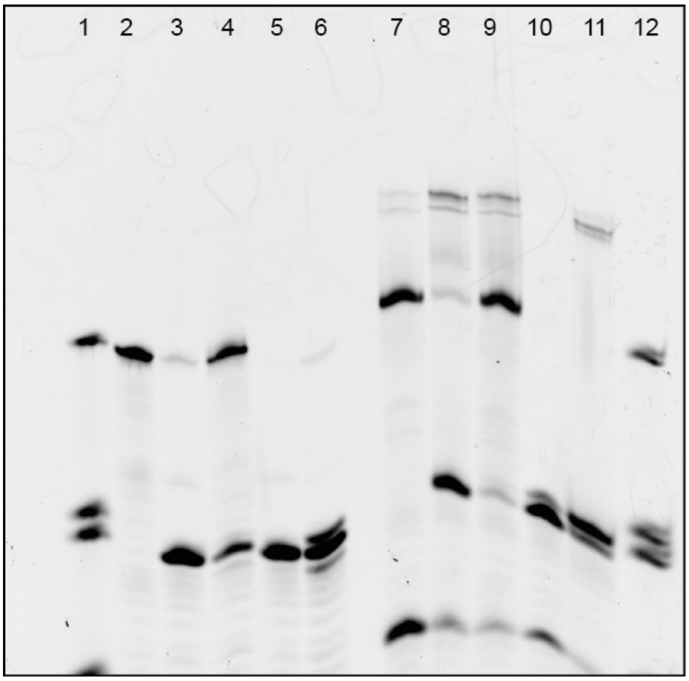

Supplementary Figure 35. 20 % sequencing gel of reverse transcription products of 48-nt RNA using SS III (2-6) and SS IV RT (8-12). 1: marker; 2: unmodified RNA; 3: 1xrTPT3-RNA w/o dNaM TP; 4: 1xrTPT3-RNA w/ dNaM TP; 5: 2xrTPT3-RNA w/o dNaM TP; 6: 2xrTPT3-RNA w/ dNaM TP; 7: unmodified RNA; 8: 1xrTPT3-RNA w/o dNaM TP; 9: 1xrTPT3-RNA w/ dNaM TP; 10: 2xrTPT3-RNA w/o dNaM TP; 11: 2xrTPT3-RNA w/ dNaM TP, 12: marker.

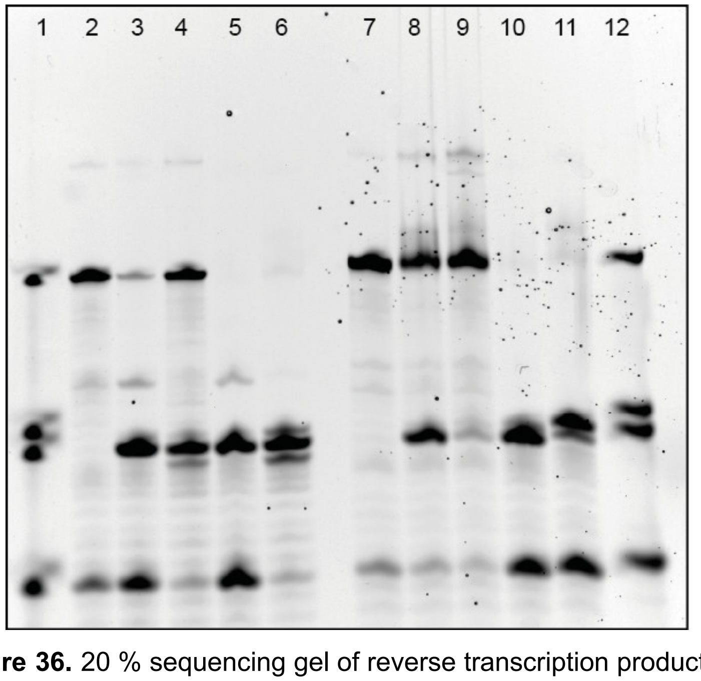

Supplementary Figure 36. 20 % sequencing gel of reverse transcription products of 48-nt RNA using SS III (2-6) and SS IV RT (8-12). 1: marker; 2: unmodified RNA; 3: 1xrTPT3-RNA w/o dNaM TP; 4: 1xrTPT3-RNA w/ dNaM TP; 5: 2xrTPT3-RNA w/o dNaM TP; 6: 2xrTPT3-RNA w/ dNaM TP; 7: unmodified RNA; 8: 1xrTPT3-RNA w/o dNaM TP; 9: 1xrTPT3-RNA w/ dNaM TP; 10: 2xrTPT3-RNA w/o dNaM TP; 11: 2xrTPT3-RNA w/ dNaM TP, 12: marker.

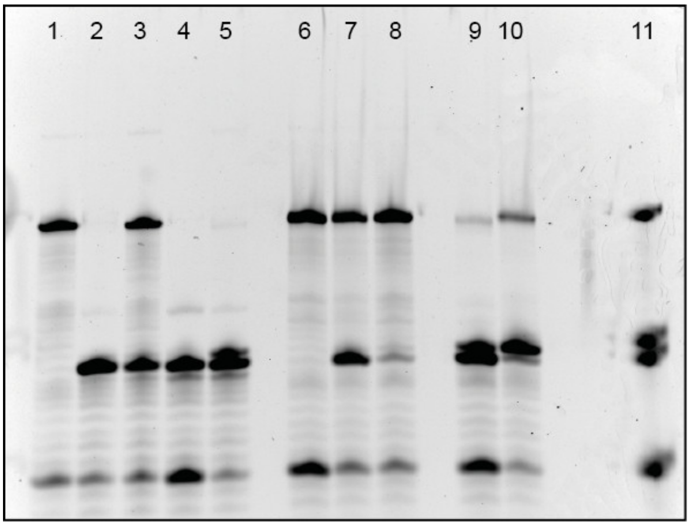

Supplementary Figure 37. 20 % sequencing gel of reverse transcription products of 48-nt RNA using SS III (2-6) and SS IV RT (8-12). 1: unmodified RNA; 2: 1xrTPT3-RNA w/o dNaM TP; 3: 1xrTPT3-RNA w/ dNaM TP; 4: 2xrTPT3-RNA w/o dNaM TP; 5: 2xrTPT3-RNA w/ dNaM TP; 6: unmodified RNA; 7: 1xrTPT3-RNA w/o dNaM TP; 8: 1xrTPT3-RNA w/ dNaM TP; 9: 2xrTPT3-RNA w/o dNaM TP; 10: 2xrTPT3-RNA w/ dNaM TP, 11: marker.

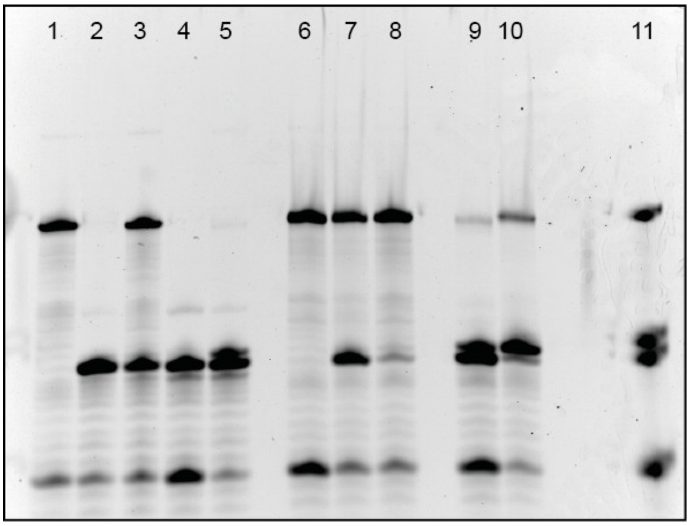

Supplementary Figure 38. 20 % sequencing gel of reverse transcription products of 48-nt RNA using SS III (2-6) and SS IV RT (8-12). 1: unmodified RNA; 2: 1xrTPT3-RNA w/o dNaM TP; 3: 1xrTPT3-RNA w/ dNaM TP; 4: 2xrTPT3-RNA w/o dNaM TP; 5: 2xrTPT3-RNA w/ dNaM TP; 6: unmodified RNA; 7: 1xrTPT3-RNA w/o dNaM TP; 8: 1xrTPT3-RNA w/ dNaM TP; 9: 2xrTPT3-RNA w/o dNaM TP; 10: 2xrTPT3-RNA w/ dNaM TP, 11: marker.

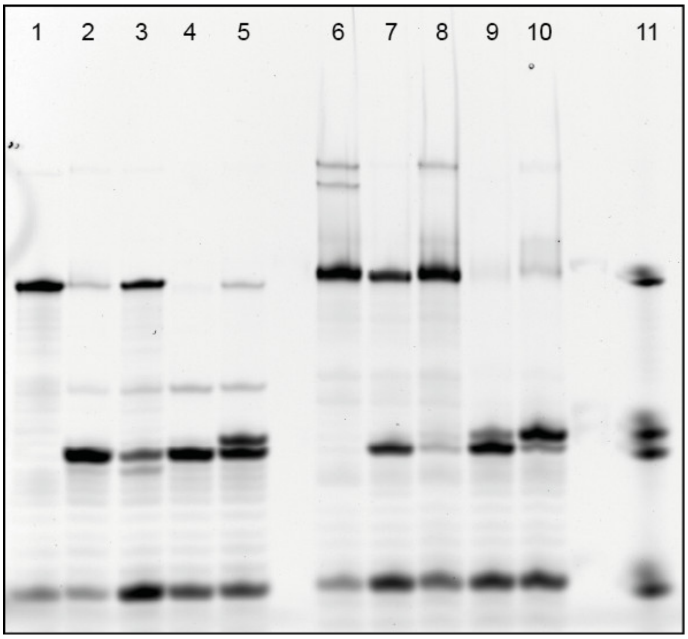

Supplementary Figure 39. 20 % sequencing gel of reverse transcription products of 48-nt RNA using SS III (2-6) and SS IV RT (8-12). 1: unmodified RNA; 2: 1xrTPT3-RNA w/o dNaM TP; 3: 1xrTPT3-RNA w/ dNaM TP; 4: 2xrTPT3-RNA w/o dNaM TP; 5: 2xrTPT3-RNA w/ dNaM TP; 6: unmodified RNA; 7: 1xrTPT3-RNA w/o dNaM TP; 8: 1xrTPT3-RNA w/ dNaM TP; 9: 2xrTPT3-RNA w/o dNaM TP; 10: 2xrTPT3-RNA w/ dNaM TP, 11: marker.

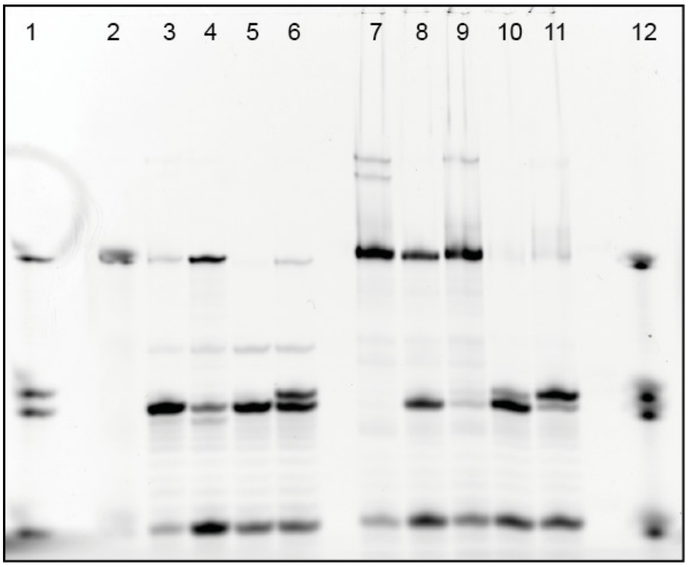

Supplementary Figure 40. 20 % sequencing gel of reverse transcription products of 48-nt RNA using SS III (2-6) and SS IV RT (8-12). 1: unmodified RNA; 2: 1xrTPT3-RNA w/o dNaM TP; 3: 1xrTPT3-RNA w/ dNaM TP; 4: 2xrTPT3-RNA w/o dNaM TP; 5: 2xrTPT3-RNA w/ dNaM TP; 6: unmodified RNA; 7: 1xrTPT3-RNA w/o dNaM TP; 8: 1xrTPT3-RNA w/ dNaM TP; 9: 2xrTPT3-RNA w/o dNaM TP; 10: 2xrTPT3-RNA w/ dNaM TP, 11: marker.

48-nt NaM-RNA:

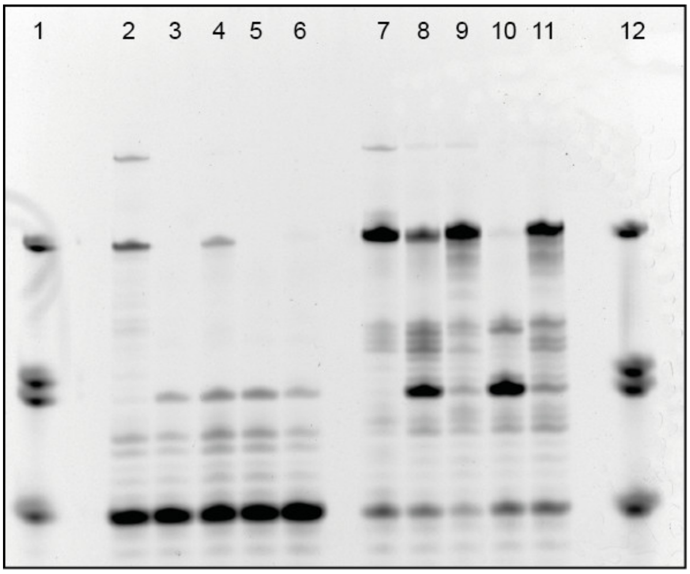

Supplementary Figure 41. 20 % sequencing gel of reverse transcription products of 48-nt RNA using AMV (2-6) and MMLV RT (7-11). 1: marker; 2: unmodified RNA; 3: 1xrNaM-RNA w/o dTPT3 TP; 4: 1xrNaM-RNA w/ dTPT3 TP; 5: 2xrNaM-RNA w/o dTPT3 TP; 6: 2xrNaM-RNA w/ dTPT3 TP; 7: unmodified RNA; 8: 1xrNaM-RNA w/o dTPT3 TP; 9: 1xrNaM-RNA w/ dTPT3 TP; 10: 2xrNaM-RNA w/o dTPT3 TP; 11: 2xrNaM-RNA w/ dTPT3 TP; 12: marker.

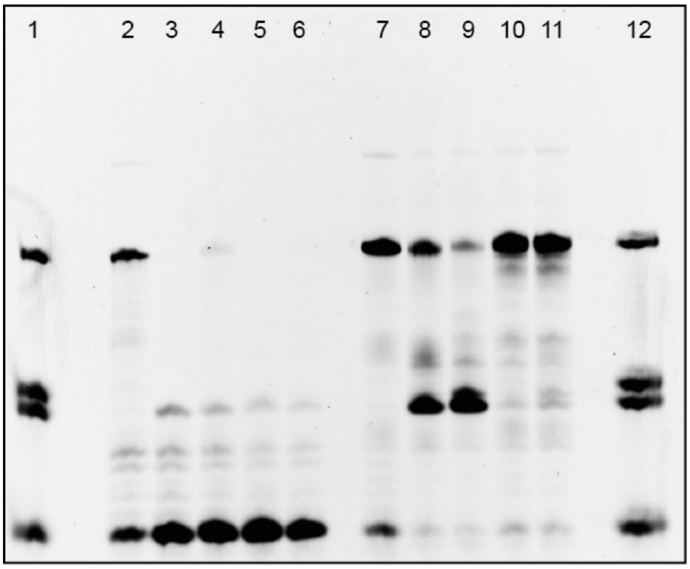

Supplementary Figure 42. 20 % sequencing gel of reverse transcription products of 48-nt RNA using AMV (2-6) and MMLV RT (7-11). 1: marker; 2: unmodified RNA; 3: 1xrNaM-RNA w/o dTPT3 TP; 4: 1xrNaM-RNA w/ dTPT3 TP; 5: 2xrNaM-RNA w/o dTPT3 TP; 6: 2xrNaM-RNA w/ dTPT3 TP; 7: unmodified RNA; 8: 1xrNaM-RNA w/o dTPT3 TP; 9: 1xrNaM-RNA w/ dTPT3 TP; 10: 2xrNaM-RNA w/o dTPT3 TP; 11: 2xrNaM-RNA w/ dTPT3 TP; 12: marker.

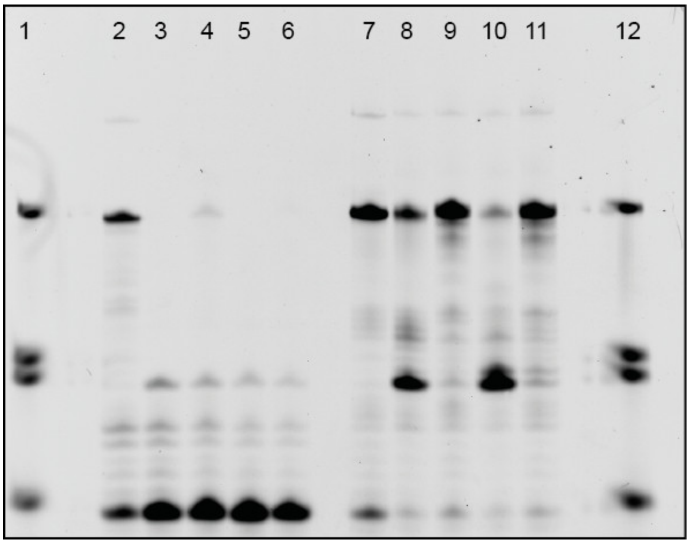

Supplementary Figure 43. 20 % sequencing gel of reverse transcription products of 48-nt RNA using AMV (2-6) and MMLV RT (7-11). 1: marker; 2: unmodified RNA; 3: 1xrNaM-RNA w/o dTPT3 TP; 4: 1xrNaM-RNA w/ dTPT3 TP; 5: 2xrNaM-RNA w/o dTPT3 TP; 6: 2xrNaM-RNA w/ dTPT3 TP; 7: unmodified RNA; 8: 1xrNaM-RNA w/o dTPT3 TP; 9: 1xrNaM-RNA w/ dTPT3 TP; 10: 2xrNaM-RNA w/o dTPT3 TP; 11: 2xrNaM-RNA w/ dTPT3 TP; 12: marker.

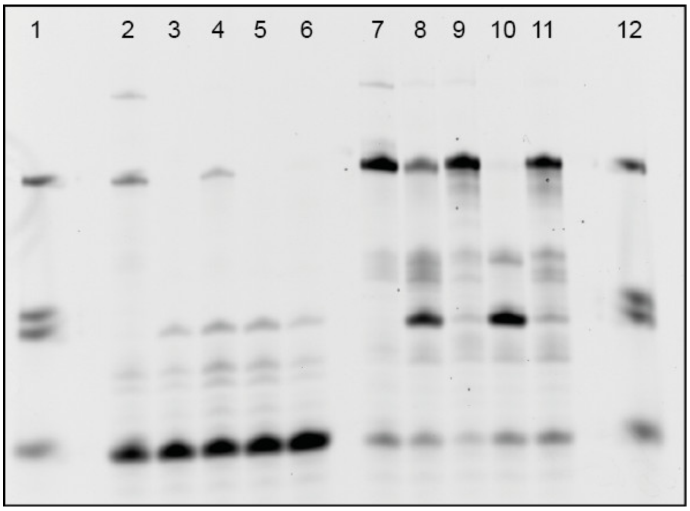

Supplementary Figure 44. 20 % sequencing gel of reverse transcription products of 48-nt RNA using AMV (2-6) and MMLV RT (7-11). 1: marker; 2: unmodified RNA; 3: 1xrNaM-RNA w/o dTPT3 TP; 4: 1xrNaM-RNA w/ dTPT3 TP; 5: 2xrNaM-RNA w/o dTPT3 TP; 6: 2xrNaM-RNA w/ dTPT3 TP; 7: unmodified RNA; 8: 1xrNaM-RNA w/o dTPT3 TP; 9: 1xrNaM-RNA w/ dTPT3 TP; 10: 2xrNaM-RNA w/o dTPT3 TP; 11: 2xrNaM-RNA w/ dTPT3 TP; 12: marker.

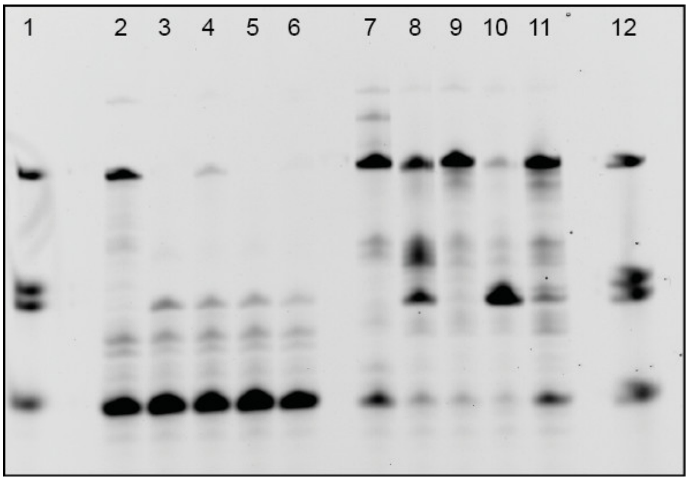

Supplementary Figure 45. 20 % sequencing gel of reverse transcription products of 48-nt RNA using AMV (2-6) and MMLV RT (7-11). 1: marker; 2: unmodified RNA; 3: 1xrNaM-RNA w/o dTPT3 TP; 4: 1xrNaM-RNA w/ dTPT3 TP; 5: 2xrNaM-RNA w/o dTPT3 TP; 6: 2xrNaM-RNA w/ dTPT3 TP; 7: unmodified RNA; 8: 1xrNaM-RNA w/o dTPT3 TP; 9: 1xrNaM-RNA w/ dTPT3 TP; 10: 2xrNaM-RNA w/o dTPT3 TP; 11: 2xrNaM-RNA w/ dTPT3 TP; 12: marker.

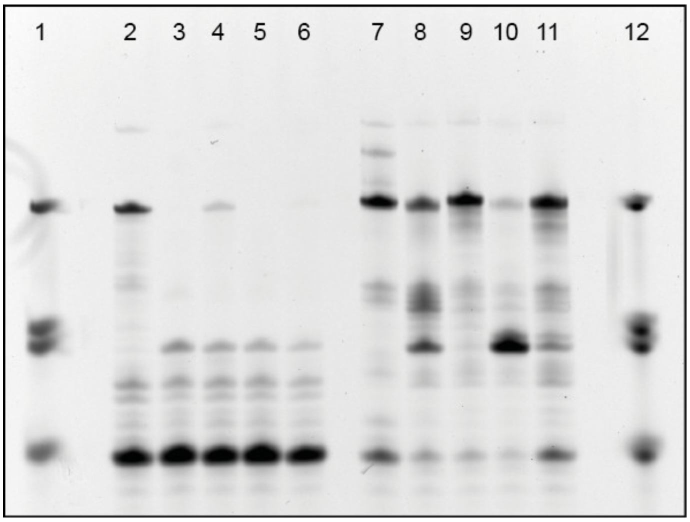

Supplementary Figure 46. 20 % sequencing gel of reverse transcription products of 48-nt RNA using AMV (2-6) and MMLV RT (7-11). 1: marker; 2: unmodified RNA; 3: 1xrNaM-RNA w/o dTPT3 TP; 4: 1xrNaM-RNA w/ dTPT3 TP; 5: 2xrNaM-RNA w/o dTPT3 TP; 6: 2xrNaM-RNA w/ dTPT3 TP; 7: unmodified RNA; 8: 1xrNaM-RNA w/o dTPT3 TP; 9: 1xrNaM-RNA w/ dTPT3 TP; 10: 2xrNaM-RNA w/o dTPT3 TP; 11: 2xrNaM-RNA w/ dTPT3 TP; 12: marker.

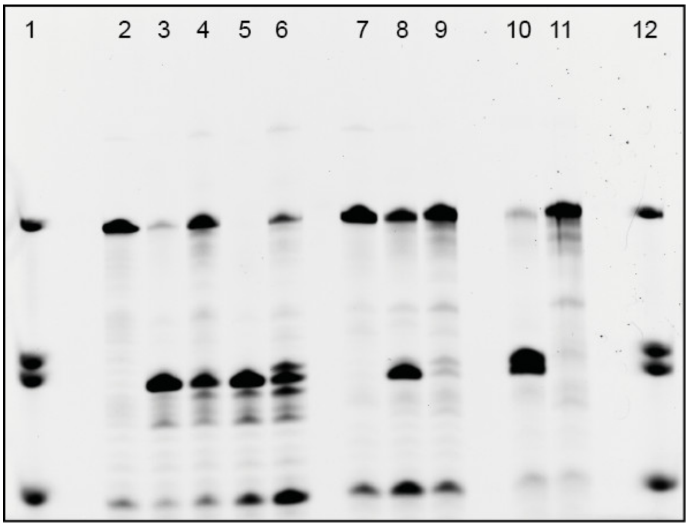

Supplementary Figure 47. 20 % sequencing gel of reverse transcription products of 48-nt RNA using SS III (2-6) and SS IV RT (8-12). 1: marker; 2: unmodified RNA; 3: 1xrNaM-RNA w/o dTPT3 TP; 4: 1xrNaM-RNA w/ dTPT3 TP; 5: 2xrNaM-RNA w/o dTPT3 TP; 6: 2xrNaM-RNA w/ dTPT3 TP; 7: unmodified RNA; 8: 1xrNaM-RNA w/o dTPT3 TP; 9: 1xrNaM-RNA w/ dTPT3 TP; 10: 2xrNaM-RNA w/o dTPT3 TP; 11: 2xrNaM-RNA w/ dTPT3 TP, 12: marker.

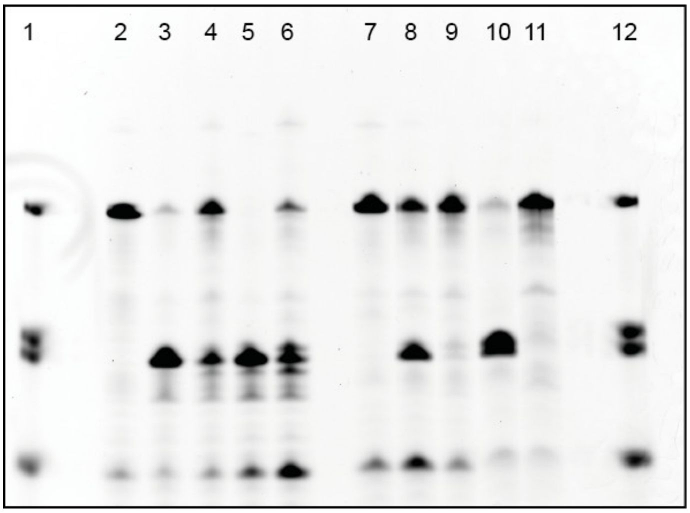

Supplementary Figure 48. 20 % sequencing gel of reverse transcription products of 48-nt RNA using SS III (2-6) and SS IV RT (8-12). 1: marker; 2: unmodified RNA; 3: 1xrNaM-RNA w/o dTPT3 TP; 4: 1xrNaM-RNA w/ dTPT3 TP; 5: 2xrNaM-RNA w/o dTPT3 TP; 6: 2xrNaM-RNA w/ dTPT3 TP; 7: unmodified RNA; 8: 1xrNaM-RNA w/o dTPT3 TP; 9: 1xrNaM-RNA w/ dTPT3 TP; 10: 2xrNaM-RNA w/o dTPT3 TP; 11: 2xrNaM-RNA w/ dTPT3 TP, 12: marker.

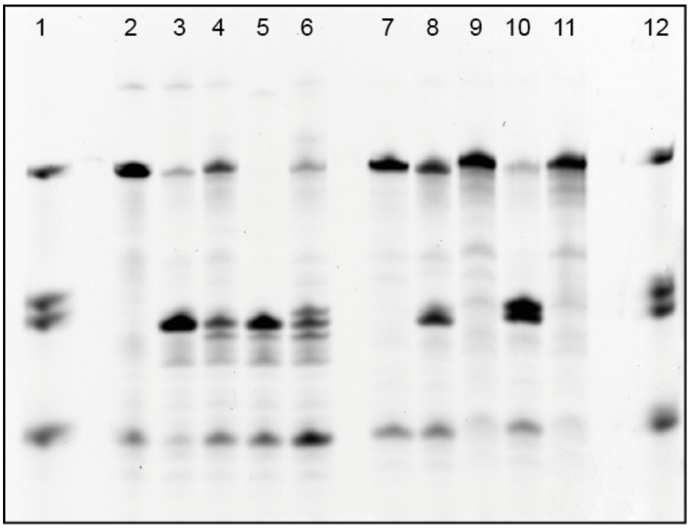

Supplementary Figure 49. 20 % sequencing gel of reverse transcription products of 48-nt RNA using SS III (2-6) and SS IV RT (8-12). 1: marker; 2: unmodified RNA; 3: 1xrNaM-RNA w/o dTPT3 TP; 4: 1xrNaM-RNA w/ dTPT3 TP; 5: 2xrNaM-RNA w/o dTPT3 TP; 6: 2xrNaM-RNA w/ dTPT3 TP; 7: unmodified RNA; 8: 1xrNaM-RNA w/o dTPT3 TP; 9: 1xrNaM-RNA w/ dTPT3 TP; 10: 2xrNaM-RNA w/o dTPT3 TP; 11: 2xrNaM-RNA w/ dTPT3 TP, 12: marker.

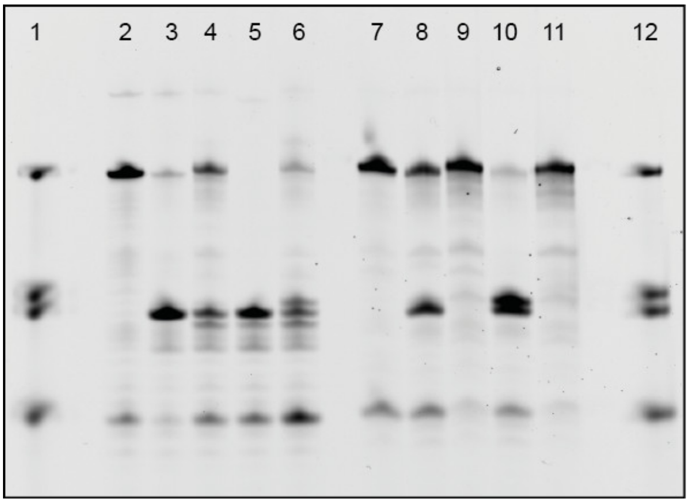

Supplementary Figure 50. 20 % sequencing gel of reverse transcription products of 48-nt RNA using SS III (2-6) and SS IV RT (8-12). 1: marker; 2: unmodified RNA; 3: 1xrNaM-RNA w/o dTPT3 TP; 4: 1xrNaM-RNA w/ dTPT3 TP; 5: 2xrNaM-RNA w/o dTPT3 TP; 6: 2xrNaM-RNA w/ dTPT3 TP; 7: unmodified RNA; 8: 1xrNaM-RNA w/o dTPT3 TP; 9: 1xrNaM-RNA w/ dTPT3 TP; 10: 2xrNaM-RNA w/o dTPT3 TP; 11: 2xrNaM-RNA w/ dTPT3 TP, 12: marker.

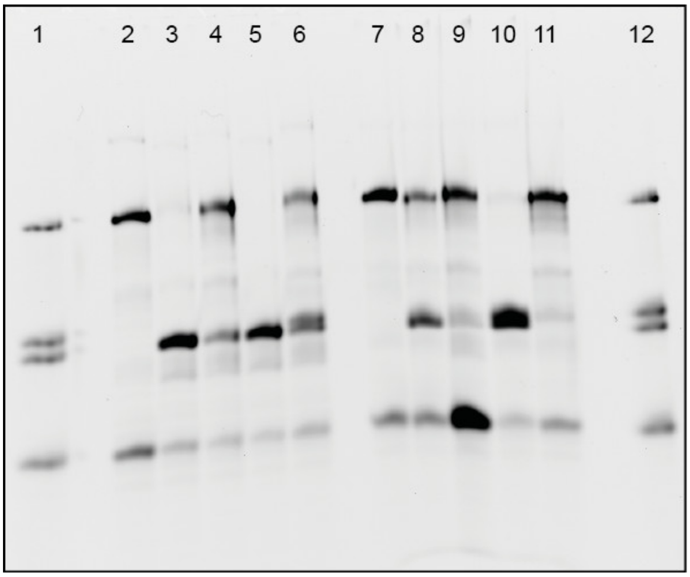

Supplementary Figure 51. 20 % sequencing gel of reverse transcription products of 48-nt RNA using SS III (2-6) and SS IV RT (8-12). 1: marker; 2: unmodified RNA; 3: 1xrNaM-RNA w/o dTPT3 TP; 4: 1xrNaM-RNA w/ dTPT3 TP; 5: 2xrNaM-RNA w/o dTPT3 TP; 6: 2xrNaM-RNA w/ dTPT3 TP; 7: unmodified RNA; 8: 1xrNaM-RNA w/o dTPT3 TP; 9: 1xrNaM-RNA w/ dTPT3 TP; 10: 2xrNaM-RNA w/o dTPT3 TP; 11: 2xrNaM-RNA w/ dTPT3 TP, 12: marker.

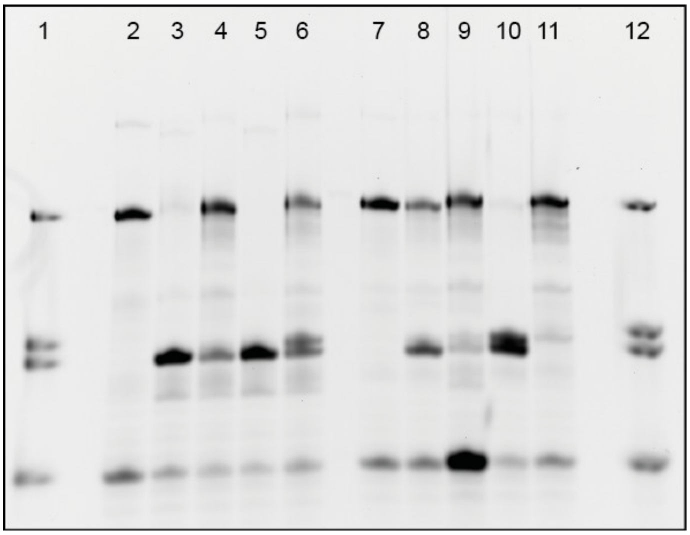

Supplementary Figure 52. 20 % sequencing gel of reverse transcription products of 48-nt RNA using SS III (2-6) and SS IV RT (8-12). 1: marker; 2: unmodified RNA; 3: 1xrNaM-RNA w/o dTPT3 TP; 4: 1xrNaM-RNA- w/ dTPT3 TP; 5: 2xrNaM-RNA w/o dTPT3 TP; 6: 2xrNaM-RNA w/ dTPT3 TP; 7: unmodified RNA; 8: 1xrNaM-RNA w/o dTPT3 TP; 9: 1xrNaM-RNA w/ dTPT3 TP; 10: 2xrNaM-RNA w/o dTPT3 TP; 11: 2xrNaM-RNA w/ dTPT3 TP, 12: marker.

rTPT3-SPINACH2 RNA:

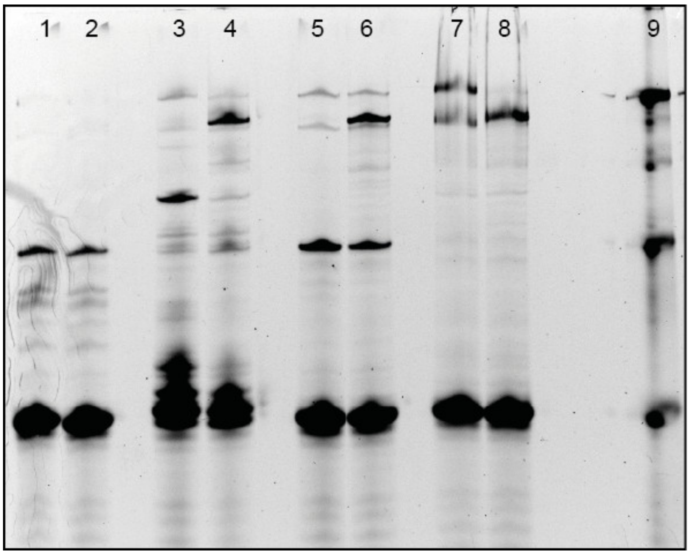

Supplementary Figure 53. 20 % sequencing gel of reverse transcription products of rTPT3-SPINACH2 RNA. 1: AMV RT w/o dNaM TP, 2: AMV RT w/ dNaM TP, 3: MMLV RT w/o dNaM TP, 4: MMLV RT w/ dNaM TP, 5: SS III RT w/o dNaM TP, 5: SS III RT w/ dNaM TP, 7: SS IV RT w/o dNaM TP, 8: SS IV RT w/o dNaM TP, 9: marker.

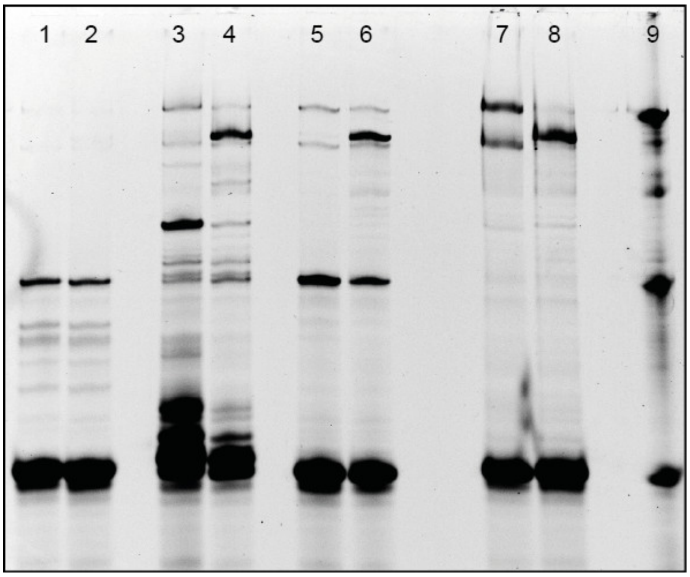

Supplementary Figure 54. 20 % sequencing gel of reverse transcription products of rTPT3-SPINACH2 RNA. 1: AMV RT w/o dNaM TP, 2: AMV RT w/ dNaM TP, 3: MMLV RT w/o dNaM TP, 4: MMLV RT w/ dNaM TP, 5: SS III RT w/o dNaM TP, 5: SS III RT w/ dNaM TP, 7: SS IV RT w/o dNaM TP, 8: SS IV RT w/o dNaM TP, 9: marker.

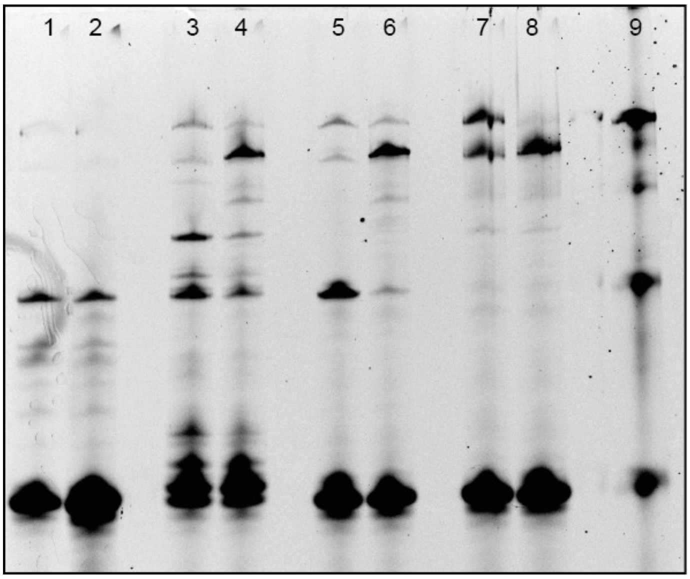

Supplementary Figure 55. 20 % sequencing gel of reverse transcription products of rTPT3-SPINACH2 RNA. 1: AMV RT w/o dNaM TP, 2: AMV RT w/ dNaM TP, 3: MMLV RT w/o dNaM TP, 4: MMLV RT w/ dNaM TP, 5: SS III RT w/o dNaM TP, 5: SS III RT w/ dNaM TP, 7: SS IV RT w/o dNaM TP, 8: SS IV RT w/o dNaM TP, 9: marker.

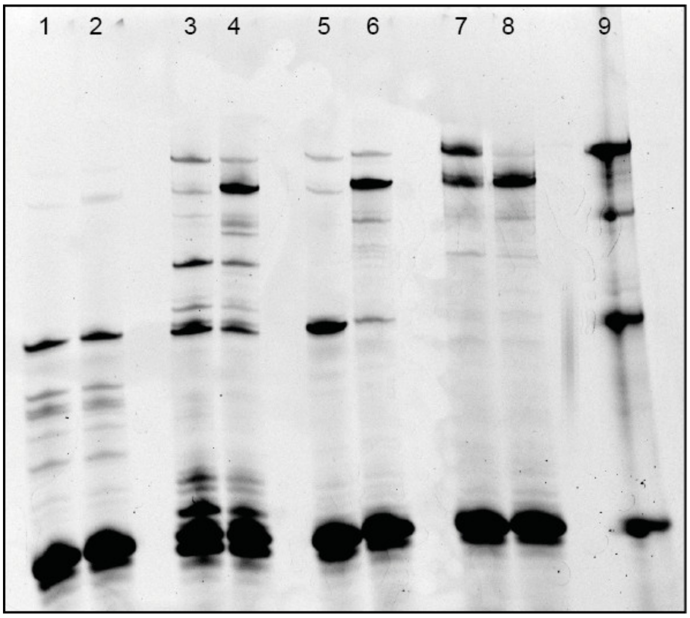

Supplementary Figure 56. 20 % sequencing gel of reverse transcription products of rTPT3-SPINACH2 RNA. 1: AMV RT w/o dNaM TP, 2: AMV RT w/ dNaM TP, 3: MMLV RT w/o dNaM TP, 4: MMLV RT w/ dNaM TP, 5: SS III RT w/o dNaM TP, 5: SS III RT w/ dNaM TP, 7: SS IV RT w/o dNaM TP, 8: SS IV RT w/o dNaM TP, 9: marker.

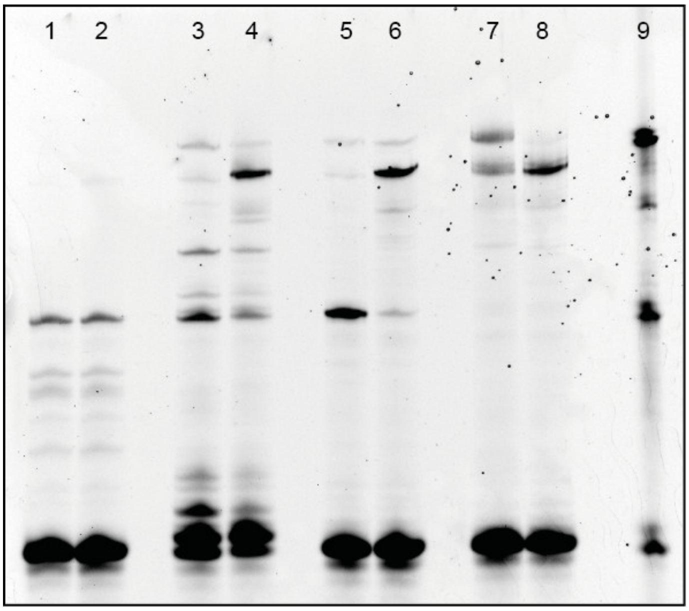

Supplementary Figure 57. 20 % sequencing gel of reverse transcription products of rTPT3-SPINACH2 RNA. 1: AMV RT w/o dNaM TP, 2: AMV RT w/ dNaM TP, 3: MMLV RT w/o dNaM TP, 4: MMLV RT w/ dNaM TP, 5: SS III RT w/o dNaM TP, 5: SS III RT w/ dNaM TP, 7: SS IV RT w/o dNaM TP, 8: SS IV RT w/o dNaM TP, 9: marker.

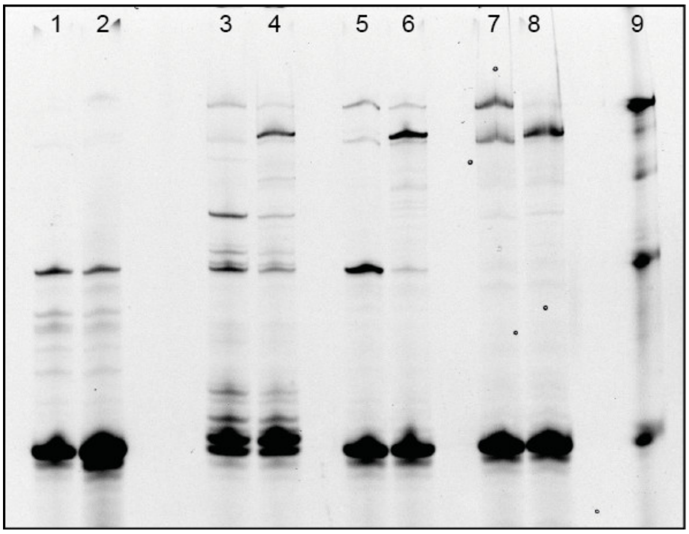

Supplementary Figure 58. 20 % sequencing gel of reverse transcription products of rTPT3-SPINACH2 RNA. 1: AMV RT w/o dNaM TP, 2: AMV RT w/ dNaM TP, 3: MMLV RT w/o dNaM TP, 4: MMLV RT w/ dNaM TP, 5: SS III RT w/o dNaM TP, 5: SS III RT w/ dNaM TP, 7: SS IV RT w/o dNaM TP, 8: SS IV RT w/o dNaM TP, 9: marker.

80-nt rTPT3-RNA:

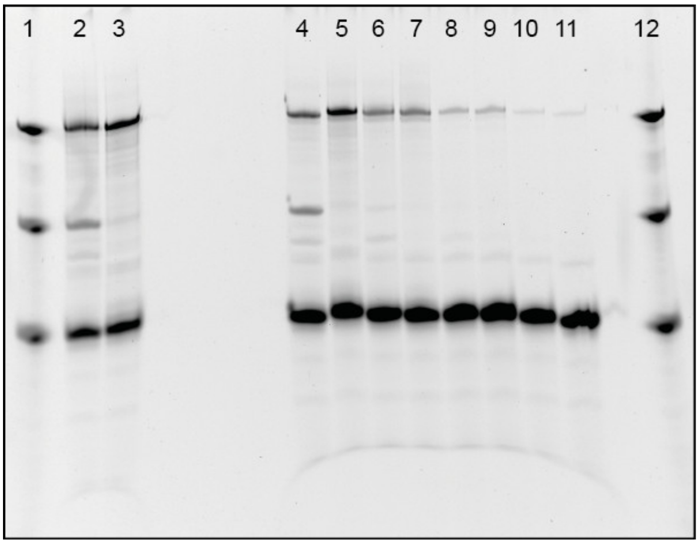

Supplementary Figure 59. 20 % sequencing gel of reverse transcription products of 80-nt rTPT3-RNA using SS IV RT and primer_RTC1. 1: marker; 2: 2500 ng RNA w/o dNaM TP; 3: 2500 ng RNA w/ dNaM TP; 4: 1250 ng RNA w/o dNaM TP; 5: 1250 ng RNA w/ dNaM TP; 6: 500 ng RNA w/o dNaM TP; 7: 500 ng RNA w/ dNaM TP; 8: 100 ng RNA w/o dNaM TP; 9: 100 ng RNA w/ dNaM TP; 10: 25 ng RNA w/o dNaM TP; 11: 25 ng RNA w/ dNaM TP; 12: marker

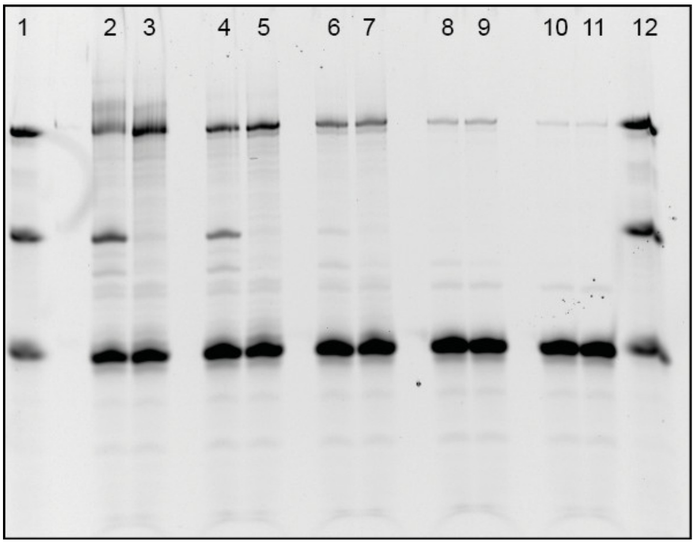

Supplementary Figure 60. 20 % sequencing gel of reverse transcription products of 80-nt rTPT3-RNA using SS IV RT and primer_RTC1. 1: marker; 2: 2500 ng RNA w/o dNaM TP; 3: 2500 ng RNA w/ dNaM TP; 4: 1250 ng RNA w/o dNaM TP; 5: 1250 ng RNA w/ dNaM TP; 6: 500 ng RNA w/o dNaM TP; 7: 500 ng RNA w/ dNaM TP; 8: 100 ng RNA w/o dNaM TP; 9: 100 ng RNA w/ dNaM TP; 10: 25 ng RNA w/o dNaM TP; 11: 25 ng RNA w/ dNaM TP; 12: marker

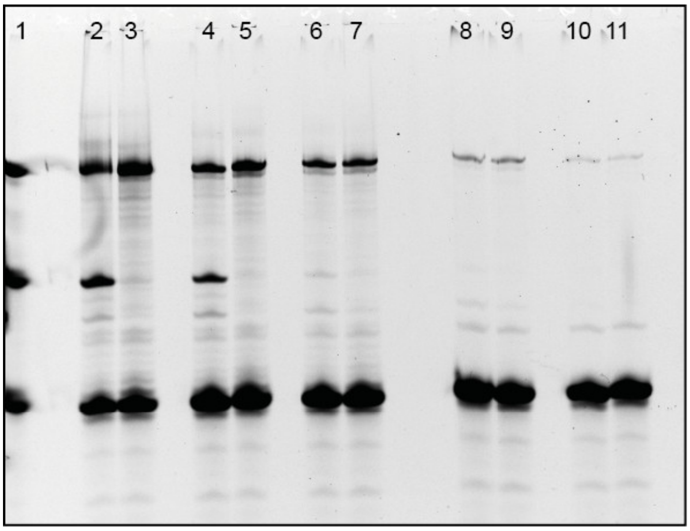

Supplementary Figure 61. 20 % sequencing gel of reverse transcription products of 80-nt rTPT3-RNA using SS IV RT and primer_RTC1. 1: marker; 2: 2500 ng RNA w/o dNaM TP; 3: 2500 ng RNA w/ dNaM TP; 4: 1250 ng RNA w/o dNaM TP; 5: 1250 ng RNA w/ dNaM TP; 6: 500 ng RNA w/o dNaM TP; 7: 500 ng RNA w/ dNaM TP; 8: 100 ng RNA w/o dNaM TP; 9: 100 ng RNA w/ dNaM TP; 10: 25 ng RNA w/o dNaM TP; 11: 25 ng RNA w/ dNaM TP

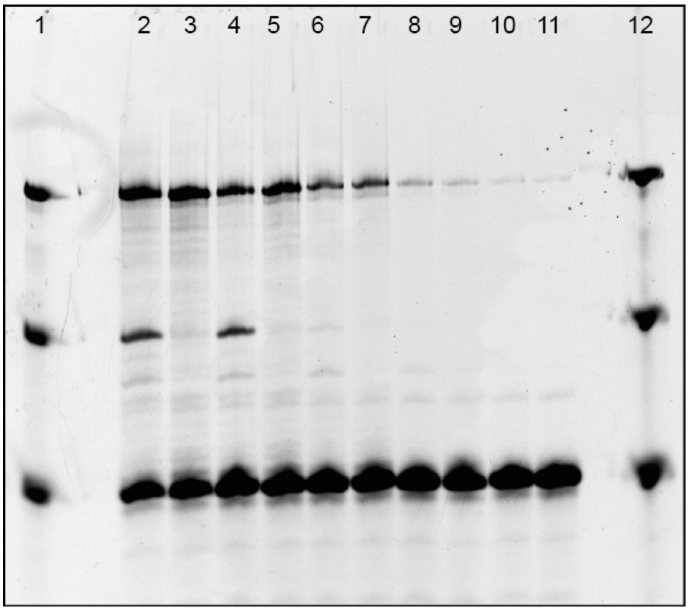

Supplementary Figure 62. 20 % sequencing gel of reverse transcription products of 80-nt rTPT3-RNA using SS IV RT and primer_RTC1. 1: marker; 2: 2500 ng RNA w/o dNaM TP; 3: 2500 ng RNA w/ dNaM TP; 4: 1250 ng RNA w/o dNaM TP; 5: 1250 ng RNA w/ dNaM TP; 6: 500 ng RNA w/o dNaM TP; 7: 500 ng RNA w/ dNaM TP; 8: 100 ng RNA w/o dNaM TP; 9: 100 ng RNA w/ dNaM TP; 10: 25 ng RNA w/o dNaM TP; 11: 25 ng RNA w/ dNaM TP; 12: marker

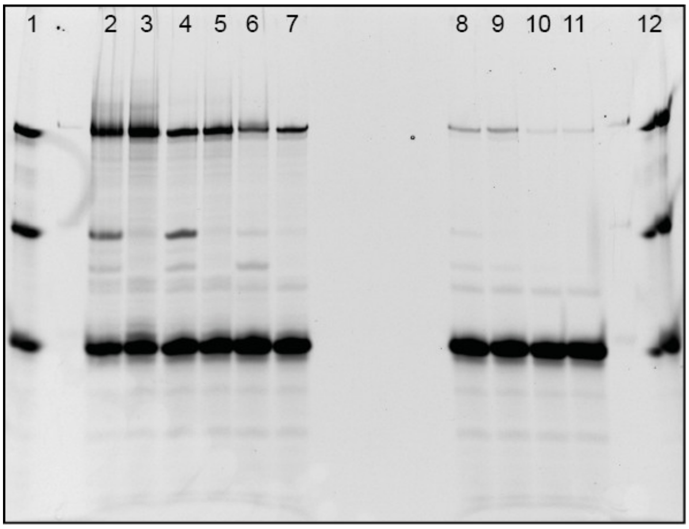

Supplementary Figure 63. 20 % sequencing gel of reverse transcription products of 80-nt rTPT3-RNA using SS IV RT and primer_RTC1. 1: marker; 2: 2500 ng RNA w/o dNaM TP; 3: 2500 ng RNA w/ dNaM TP; 4: 1250 ng RNA w/o dNaM TP; 5: 1250 ng RNA w/ dNaM TP; 6: 500 ng RNA w/o dNaM TP; 7: 500 ng RNA w/ dNaM TP; 8: 100 ng RNA w/o dNaM TP; 9: 100 ng RNA w/ dNaM TP; 10: 25 ng RNA w/o dNaM TP; 11: 25 ng RNA w/ dNaM TP; 12: marker

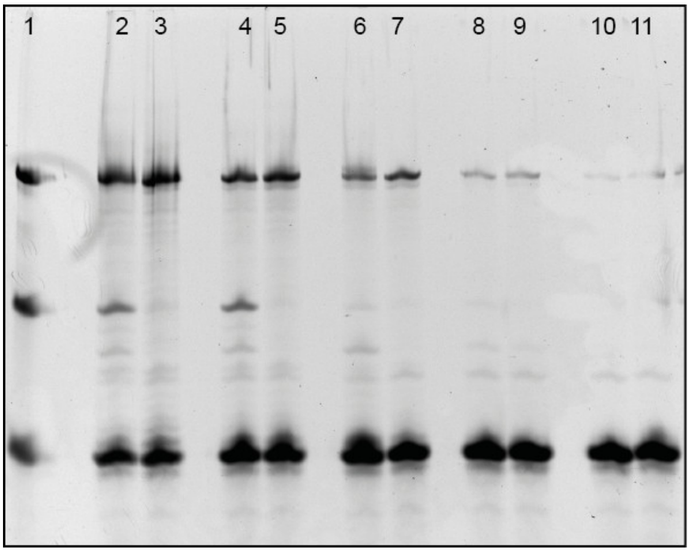

Supplementary Figure 64. 20 % sequencing gel of reverse transcription products of 80-nt rTPT3-RNA using SS IV RT and primer_RTC1. 1: marker; 2: 2500 ng RNA w/o dNaM TP; 3: 2500 ng RNA w/ dNaM TP; 4: 1250 ng RNA w/o dNaM TP; 5: 1250 ng RNA w/ dNaM TP; 6: 500 ng RNA w/o dNaM TP; 7: 500 ng RNA w/ dNaM TP; 8: 100 ng RNA w/o dNaM TP; 9: 100 ng RNA w/ dNaM TP; 10: 25 ng RNA w/o dNaM TP; 11: 25 ng RNA w/ dNaM TP

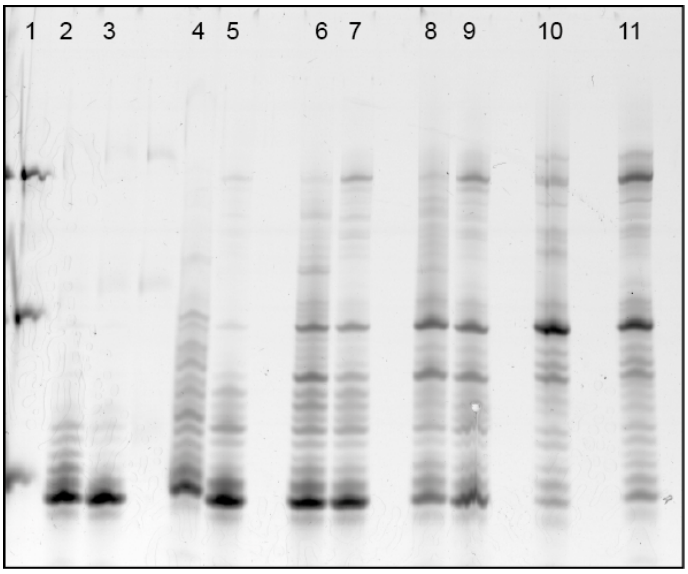

Supplementary Figure 65. 20 % sequencing gel of reverse transcription products of 80-nt rTPT3-RNA using MMLV RT and primer_RTC1. 1: marker; 2: 25 ng RNA w/o dNaM TP; 3: 25 ng RNA w/ dNaM TP; 4: 100 ng RNA w/o dNaM TP; 5: 100 ng RNA w/ dNaM TP; 6: 500 ng RNA w/o dNaM TP; 7: 500 ng RNA w/ dNaM TP; 8: 1250 ng RNA w/o dNaM TP; 9: 1250 ng RNA w/ dNaM TP; 10: 2500 ng RNA w/o dNaM TP; 11: 2500 ng RNA w/ dNaM TP.

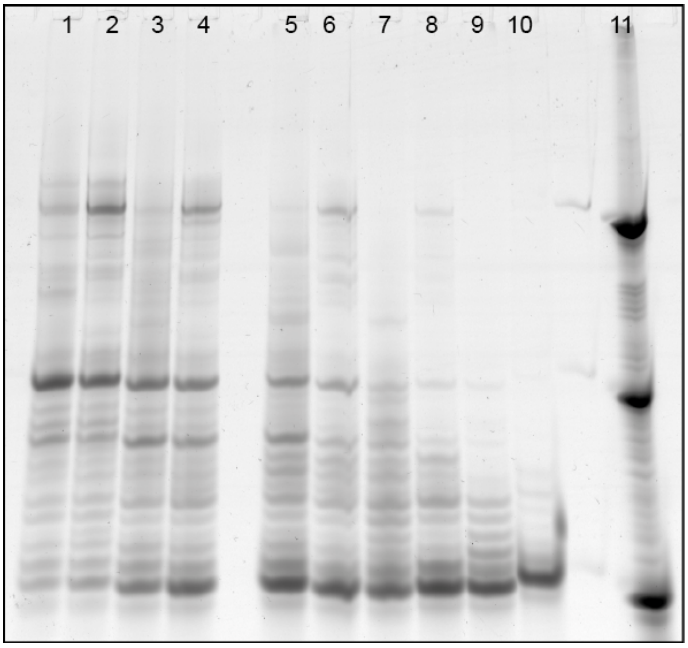

Supplementary Figure 66. 20 % sequencing gel of reverse transcription products of 80-nt rTPT3-RNA using MMLV RT and primer_RTC1. 1: 2500 ng RNA w/o dNaM TP; 2: 2500 ng RNA w/ dNaM TP; 3: 1250 ng RNA w/o dNaM TP; 4: 1250 ng RNA w/ dNaM TP; 5: 500 ng RNA w/o dNaM TP; 6: 500 ng RNA w/ dNaM TP; 7: 100 ng RNA w/o dNaM TP; 8: 100 ng RNA w/ dNaM TP; 9: 25 ng RNA w/o dNaM TP; 10: 25 ng RNA w/ dNaM TP; 11: marker.

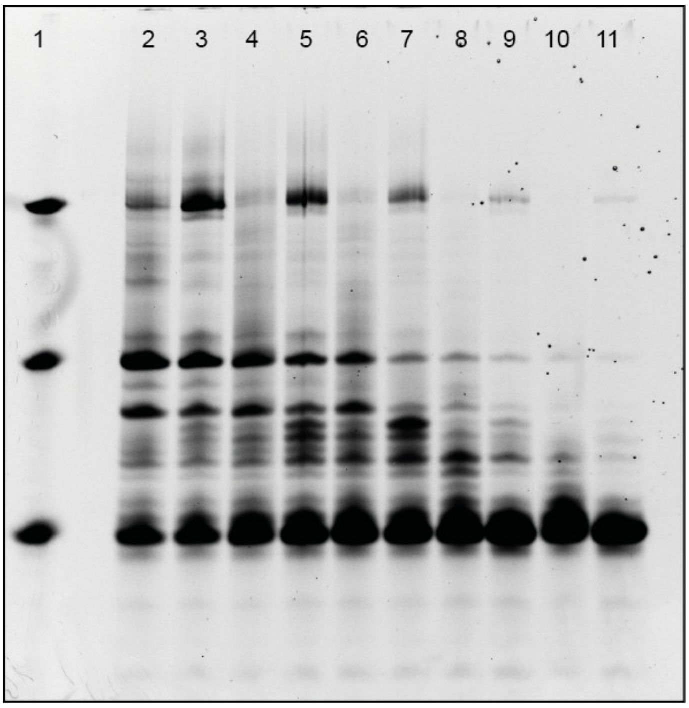

Supplementary Figure 67. 20 % sequencing gel of reverse transcription products of 80-nt rTPT3-RNA using MMLV RT and primer_RTC1. 1: marker; 2: 2500 ng RNA w/o dNaM TP; 3: 2500 ng RNA w/ dNaM TP; 4: 1250 ng RNA w/o dNaM TP; 5: 1250 ng RNA w/ dNaM TP; 6: 500 ng RNA w/o dNaM TP; 7: 500 ng RNA w/ dNaM TP; 8: 100 ng RNA w/o dNaM TP; 9: 100 ng RNA w/ dNaM TP; 10: 25 ng RNA w/o dNaM TP; 11: 25 ng RNA w/ dNaM TP.

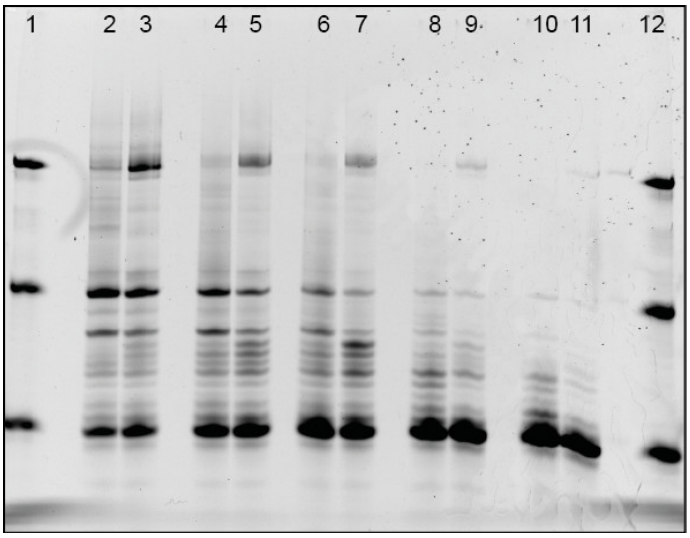

Supplementary Figure 68. 20 % sequencing gel of reverse transcription products of 80-nt rTPT3-RNA using MMLV RT and primer_RTC1. 1: marker; 2: 2500 ng RNA w/o dNaM TP; 3: 2500 ng RNA w/ dNaM TP; 4: 1250 ng RNA w/o dNaM TP; 5: 1250 ng RNA w/ dNaM TP; 6: 500 ng RNA w/o dNaM TP; 7: 500 ng RNA w/ dNaM TP; 8: 100 ng RNA w/o dNaM TP; 9: 100 ng RNA w/ dNaM TP; 10: 25 ng RNA w/o dNaM TP; 11: 25 ng RNA w/ dNaM TP; 12: marker.

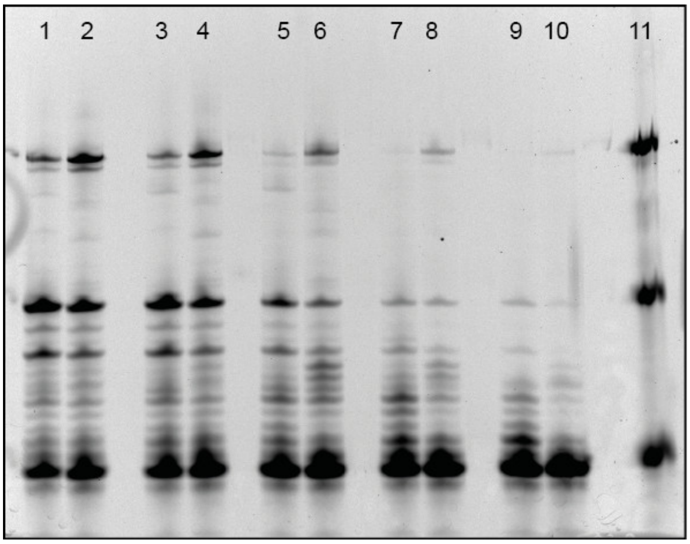

Supplementary Figure 69. 20 % sequencing gel of reverse transcription products of 80-nt rTPT3-RNA using MMLV RT and primer_RTC1. 1: 2500 ng RNA w/o dNaM TP; 2: 2500 ng RNA w/ dNaM TP; 3: 1250 ng RNA w/o dNaM TP; 4: 1250 ng RNA w/ dNaM TP; 5: 500 ng RNA w/o dNaM TP; 6: 500 ng RNA w/ dNaM TP; 7: 100 ng RNA w/o dNaM TP; 8: 100 ng RNA w/ dNaM TP; 9: 25 ng RNA w/o dNaM TP; 10: 25 ng RNA w/ dNaM TP; 11: marker

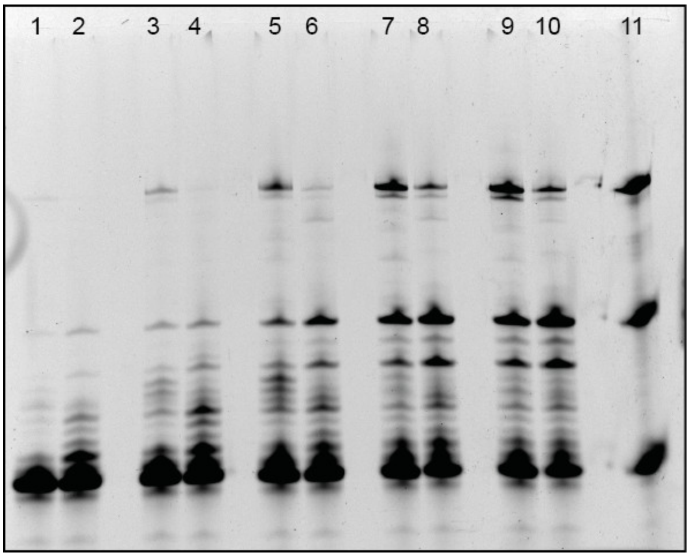

Supplementary Figure 70. 20 % sequencing gel of reverse transcription products of 80-nt rTPT3-RNA using MMLV RT and primer_RTC1. 1: 25 ng RNA w/o dNaM TP; 2: 25 ng RNA w/ dNaM TP; 3: 100 ng RNA w/o dNaM TP; 4: 100 ng RNA w/ dNaM TP; 5: 500 ng RNA w/o dNaM TP; 6: 500 ng RNA w/ dNaM TP; 7: 1250 ng RNA w/o dNaM TP; 8: 1250 ng RNA w/ dNaM TP; 9: 25 ng RNA w/o dNaM TP; 10: 25 ng RNA w/ dNaM TP; 11: marker

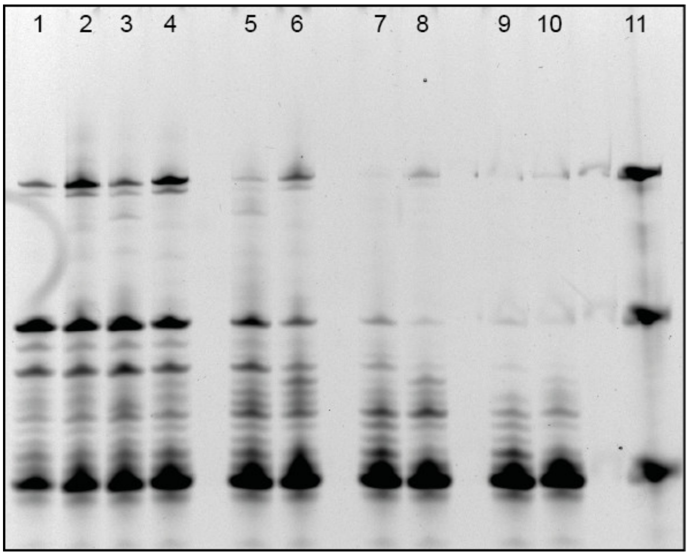

Supplementary Figure 71. 20 % sequencing gel of reverse transcription products of 80-nt rTPT3-RNA using MMLV RT and primer_RTC1. 1: 2500 ng RNA w/o dNaM TP; 2: 2500 ng RNA w/ dNaM TP; 3: 1250 ng RNA w/o dNaM TP; 4: 1250 ng RNA w/ dNaM TP; 5: 500 ng RNA w/o dNaM TP; 6: 500 ng RNA w/ dNaM TP; 7: 100 ng RNA w/o dNaM TP; 8: 100 ng RNA w/ dNaM TP; 9: 25 ng RNA w/o dNaM TP; 10: 25 ng RNA w/ dNaM TP; 11: marker

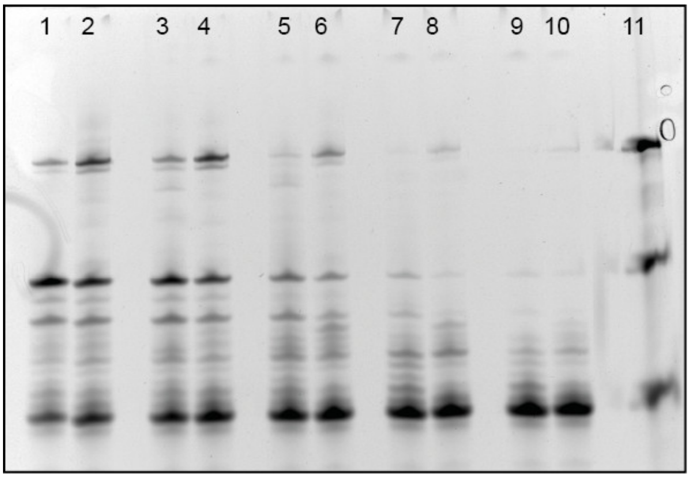

Supplementary Figure 72. 20 % sequencing gel of reverse transcription products of 80-nt rTPT3-RNA using MMLV RT and primer_RTC1. 1: 2500 ng RNA w/o dNaM TP; 2: 2500 ng RNA w/ dNaM TP; 3: 1250 ng RNA w/o dNaM TP; 4: 1250 ng RNA w/ dNaM TP; 5: 500 ng RNA w/o dNaM TP; 6: 500 ng RNA w/ dNaM TP; 7: 100 ng RNA w/o dNaM TP; 8: 100 ng RNA w/ dNaM TP; 9: 25 ng RNA w/o dNaM TP; 10: 25 ng RNA w/ dNaM TP; 11: marker

8. DNA and RNA oligonucleotides

All DNA and RNA oligonucleotides used in this study are presented in the following tables with X = NaM, Y= TPT3

Supplementary Table 10. Overview of the oligonucleotides that were used for reverse transcriptions and PAGE analysis of 80-nt TPT3-RNA. RNA was prepared by T7 *in vitro* transcription using chemically synthesized DNA templates.

Name	Sequence (5'-3')	End-Modification
DNA primer for T7 transcription	GGC GTA ATA CGA CTC ACT ATA GGG	
DNA template for preparation of 80-nt rTPT3-RNA	CA AGC TTA CTT GTC GTC GTC GTC CTT GTA GTC CXT CTT GTA CTT TTT GTA CAT GTT TTT CTC CTT CTT AAA GTT AAC CCT ATA GTG AGT CGT ATT ACG CC	
DNA primer for RTC and marker (PAGE analysis)	CGA AGC TTA CTT GTC GTC GTC GTC CTT GT	5'-6FaM
DNA for stop band in marker	CGA AGC TTA CTT GTC GTC GTC GTC CTT GTA GTC C	5'-6FaM
DNA for full-length band in marker	CGA AGC TTA CTT GTC GTC GTC GTC CTT GTA GTC CAT CTT GTA CTT TTT GTA CAT GTT TTT CTC CTT CTT AAA GTT AAC CC	5'-6FaM

Supplementary Table 11. Overview of the oligonucleotides that were used for experiments with 48-nt RNA

Name	Sequence (5'-3')	End-Modification
48-nt RNA	AAG UAU CGU AUC GCC CGA GUA UAC AGG GAA UCC CGA GUA GUG GGA CAG	
48-1xrTPT3 RNA	AAG UAU CGU AUC GCC CGA GUA UA**Y** AGG GAA UCC CGA GUA GUG GGA CAG	
48-1xrNaM RNA	AAG UAU CGU AUC GCC CGA GUA UA**X** AGG GAA UCC CGA GUA GUG GGA CAG	
48-2xrTPT3 RNA	AAG UAU CGU AUC GCC CGA GUA U**YY** AGG GAA UCC CGA GUA GUG GGA CAG	
48-2xrNaM RNA	AAG UAU CGU AUC GCC CGA GUA U**XX** AGG GAA UCC CGA GUA GUG GGA CAG	
Primer for RTC of 48-nt RNA for PAGE analysis and LC-MS	CTG TCC CAC TAC TCG	5'-6FaM
DNA for stop band at position 25 in marker	CTG TCC CAC TAC TCG GGA TTC CCT	5'-6FaM
DNA for stop band at position 26 in marker	CTG TCC CAC TAC TCG GGA TTC CCT G	5'-6FaM
DNA for full-length band in marker	CTG TCC CAC TAC TCG GGA TTC CCT GTA TAC TCG GGC GAT ACG ATA CTT	5'-6FaM
Primer for RTC for Sanger Sequencing	CTG TCC CAC TAC TCG GGA TTC CCT	
DNA for Overlap Extension PCR	CAA CGT TAC TTG TCG TCG TCG CTA TAG GGA TGT AAC TGA ATG AAA TGG GTA ACT AGT TAC GGA GCT CAC ACT CTA CTC GTA GGA GCT CAC AAG TAT CGT ATC GCC CG	
Forward Primer for Sequencing with PolyT overhang	TTT TTT TTT TTT TTT TTT TTT TTT TTT TTT TTT TTT TTT TTT TTT TTT TTT TTT TTT TTT AGT CAA CGT TAC TTG TCG TCG TCG	

Supplementary Table 11. continued.

Name	Sequence (5'-3')	End-Modification
DNA oligonucleotide with dNaM as reference for sequencing	AGT TAC GGA GCT CAC ACT CTA CTC AAC AAG TAG GCT GCC GAA GCA GCC **X**AC TGG ACC CGT CCT TCA CCA TTT CAT TCA GTT ACA TCC CTA TAG TGA GTC GTA TTA TAC TAG AGT CGC CGA CGA CGA CAA GTA ACG TTG	
Unmodified DNA oligonucleotide as reference for sequencing	CTG TCC CAC TAC TCG GGA TTC CCT GTA TAC TCG GGC GAT ACG ATA CTT GTG AGC TCC TAC GAG TAG AGT GTG AGC TCC GTA ACT AGT TAC CCA TTT CAT TCA GTT ACA TCC CTA TAG CGA CGA CGA CAA GTA ACG TTG	

Supplementary Table 12. Overview of the oligonucleotides that were used for real-time monitoring of reverse transcriptions using a heliX$^+$ biosensor.

Name	Sequence (5'-3')	End-Modification
Ligand Strand	ATC AGT ACT TGT CAA CAC GAG CAG CCC GTA TAT TCT CCT ACA GCA CTA	
24 nt	AGG GAA UCC CGA GUA GUG GGA CAG	5'-rhodamine derivative
48-nt	AAG UAU CGU AUC GCC CGA GUA UAC AGG GAA UCC CGA GUA GUG GGA CAG	5'-rhodamine derivative
48-1xrTPT3	AAG UAU CGU AUC GCC CGA GUA UA**Y** AGG GAA UCC CGA GUA GUG GGA CAG	5'-rhodamine derivative
48-2xrTPT3	AAG UAU CGU AUC GCC CGA GUA U**YY** AGG GAA UCC CGA GUA GUG GGA CAG	5'-rhodamine derivative

Supplementary Table 13. Overview of the oligonucleotides that were used for experiments with SPINACH2 RNA.

Name	Sequence (5'-3')	End-Modification
DNA primer for T7 transcription	GTA TAA TAC GAC TCA CTA TAG GG	
DNA template for preparation of unmodified SPINACH2 RNA	(OG*)(OA**)T GTA ACT AGT TAC GGA GCT CAC ACT CTA CTC AAC AAG TAG GCT GCC GAA GCA GCC TAC TGG ACC CGT CCT TCA CCA TTT CAT TCA GTT ACA TCC CTA TAG TGA GTC GTA TTA TAC	
DNA template for preparation of rTPT3/rTPT3^{CP}-SPINACH2 RNA	(OG*)(OA**)T GTA ACT AGT TAC GGA GCT CAC ACT CTA CTC AAC AAG TAG GCT GCC GAA GCA GCC **X**AC TGG ACC CGT CCT TCA CCA TTT CAT TCA GTT ACA TCC CTA TAG TGA GTC GTA TTA TAC	
Primer for RTC (PAGE analysis)	GAT GTA ACT AGT TAC GGA GCT CAC	5'-6FaM
DNA for stop band (PAGE analysis)	GAT GTA ACT AGT TAC GGA GCT CAC ACT CTA CTC AAC AAG TAG GCT GCC GAA GCA GCC	5'-6FaM
DNA for full-length band (PAGE analysis)	GAT GTA ACT AGT TAC GGA GCT CAC ACT CTA CTC AAC AAG TAG GCT GCC GAA GCA GCC TAC TGG ACC CGT CCT TCA CCA TTT CAT TCA GTT ACA TCC C	5'-6FaM
Forward primer for RTC and PCR (SELEX)	(OG*)(OA**)T GTA ACT AGT TAC GGA GCT CAC	
Reverse primer for PCR (SELEX)	GTA TAA TAC GAC TCA CTA TAG GGA TGT AAC TGA ATG AAA	

* OG = 2'-Methoxy-Guanine, **OA = 2'-Methoxy-Adenine

9. Synthesis of phosphoramidites for RNA synthesis

General Experimental Conditions and Analytical Methods

Working with Inert Gas

For reactions sensible to water or oxygen an inert gas/oil pump vacuum system was used. Reaction vessels were evacuated, heated, and flooded with argon three times before usage. As inert gas argon BIP® (99.9997 %) by *Air Products* was used without further purification.

Solvents and Reactants

Solvents and reactants with a purity >95% were used. If not specified otherwise, reactants were used without further purification. Solvents, such as EtOAc, *c*-Hex, DCM, MeOH and EtOH, were distilled before usage. For pentane, Et_2O, $CHCl_3$, toluene and MeCN HPLC grade solvents were used without further purification. Absolute solvents were bought (*ACRO Seal*, extra dry, stored under argon and over mole sieves) and used without further purification.

Chromatographic Methods

Thin-Layer Chromatography (TLC)

For thin-layer chromatography *Macherey-Nagel* POLYGRAM® Sil G/UV 254 foils were used. They were coated with silica gel (layer thickness 250 µm) and fluorescence indicator. For detection UV light (λ = 254 nm) was used. Additionally, a $KMnO_4$-staining solution (3 g $KMnO_4$, 20 g K_2CO_3, 5 mL 5% NaOH solution and 300 mL water) was used to stain spots which are not visible in UV light. Solvent mixtures are given in volume fraction.

Column Chromatography (CC)

For column chromatography Silica Gel 60 (0.035-0.07 mm) from *Acros* was used as stationary phase. Solvent mixtures are given in volume fraction at the appropriate positions. Flow rate was increased by excess pressure, which was applied via a hand pump.

Analytical Methods

Nuclear Magnetic Resonance (NMR)

For the recording of NMR spectra a *Bruker Avance II 300* (^{1}H: 300 MHz, ^{13}C: 75 MHz), a *Bruker Avance 400* (^{1}H: 400 MHz, ^{13}C: 101 MHz), a *Bruker Avance III 499* (^{1}H: 500 MHz, ^{13}C: 125 MHz) and *Bruker Avance III 500* (^{1}H: 500 MHz, ^{13}C: 125 MHz) were used. All measurements were recorded at room temperature, if not specified otherwise. Chemical shifts were measured relatively to the TMS, or solvent residual signal. For fine structure of the signals the following abbreviations were chosen: s = singlet, d = duplet, t = triplet, q = quartet, sept. = septet, dd = duplet of duplets, dt = duplet of triplets, qd = quartet of duplets, m = multiplet. Numbering of atoms does not mandatorily correlate to the IUPAC-nomenclature.

(High Resolution) Mass Spectroscopy [(HR)-MS]
For electron spray isonisation (ESI) measurements an *Agilent LC/MSD VL* (ESI-MS) or a *THERMO Scientific LTQ Orbitrap XY* with an *FTMS Analyzer* (ESI-HRMS) was used. Application of the sample was done by electrospray ionisation with a flow of 500 µL/min and a capillary tension of 3.6 kV. As solvent methanol with the addition of 0.5% acetic acid was used.

Fourier-Transform Infrared Spectroscopy (FT-IR)
The spectra were measured on a *Shimadazu IRAffinity-1 FT*-IR spectrometer using the ATR technique (ATR: Attenuated Total Reflectance). Position of the bands is given in wavenumbers [cm^{-1}]. The intensity is given with the following abbreviations: s = strong, m = medium, w = weak and b = broad.

Melting Point Measurement
A *MP50 Melting Point System* from *Mettler-Toledo* was used.

Gas Chromatography with a Mass Spectrometric Detector (GC-MS)
For GC-MS analysis either a *Hewlett Packard HP 6890 Series GC* system with a *Hewlett Packard 5973 Mass Selective* mass detector or an *Agilent Technologies 7890A* GC system with an *Agilent Technologies 5975 Triple Axis* mass detector were used. In both cases a *HP5 MS Crosslinked Silicone Gum Column* (30 m x 250 µm, film thickness: 250 µm) with H_2 as carrier gas and a flow rate of 1 mL/min was used. As temperature method STAND50 (injector: 180 °C (split ratio: 50:1), oven: 50 °C (5 min), 20 °C/min, 280 °C (10 min)) was used, if not stated otherwise.

Oligonucleotide Synthesis

Device:
For in house oligonucleotide synthesis a *H-6* synthesis robot from *K&A Labs GmbH* was used.

Reagents:
CPGs: Preloaded CPGs were purchased either from *Eurogentec* or *LGC Biosearch Technologies*. If not stated otherwise 200 nmol synthesis scale were used.

Phosphoramidites: Commercially available phosphoramidites were purchased either from *GlenResearch*, *LGC Biosearch Technologies* or *Hongene Biotech Germany GmbH*. If not stated otherwise, DNA phosphoramidites were used in a concentration of 0.05 M, RNA- and all unnatural phosphoramidites in 0.1 M. If not stated otherwise, all RNA-phosphoramidites were used as cyanoethyl-diisopropyl-phosphoramidites with a 2'-TBS protection group. For the nucleobases following protection groups were used: dA(bz), dC(Bz), dG(ibu), A(Bz), G(dmf), C(Ac)

Following reagents were used during solid support synthesis: acetonitrile: 99.9%, extra dry over molecular sieves from *Thermo Scientific;* Cap A: tetrahydrofuran/lutidine/acetic anhydride 8/1/1 (v/v/v) (L040030 from *Sigma-Aldrich*); Cap B: tetrahydrofuran/N-Methylimidazole 84/16 (v/v) (L050030 from *Sigma-Aldrich*); Oxidizer: tetrahyrofuran/water/pyridine/iodine 77/2/21/2,54 (v/v/v/w) (L060030 from *Sigma-Aldrich*); Activator: 0.25 M 5-(Ethylthio)-1*H*-tetrazole in acetonitrile (L030000 from *Sigma-Aldrich*);

Deblocking Mix: dichloromethane/trichloracetic acid 97/3 (v/v) (40-4140-61 from *Glen Research*).

Solid Support Synthesis: reaction parameters
If not stated otherwise, the following reaction sequences were used for oligonucleotide synthesis: detritylation, coupling (2 min for DNA, 10 min for RNA and all unnatural building blocks), capping, oxidation, capping. Capping was performed twice to guarantee complete capping of free OH-groups after coupling.

Oligonucleotide deprotection and purification
If not stated otherwise, the synthesis was carried out DMT-on and oligonucleotides were deprotected and purified with *Glen-Pak™ DNA purification cartridges* from *GlenResearch* according to the manufacturer's standard protocol. For CPG cleavage and deprotection of base labile groups, sat. aq. ammonia (24 h at r.t.) was used.

Synthesis procedures

Overview about performed reactions:

Synthesis of rTPT3-nucleoside

rTPT3 was synthesized according to literature.[1, 2]

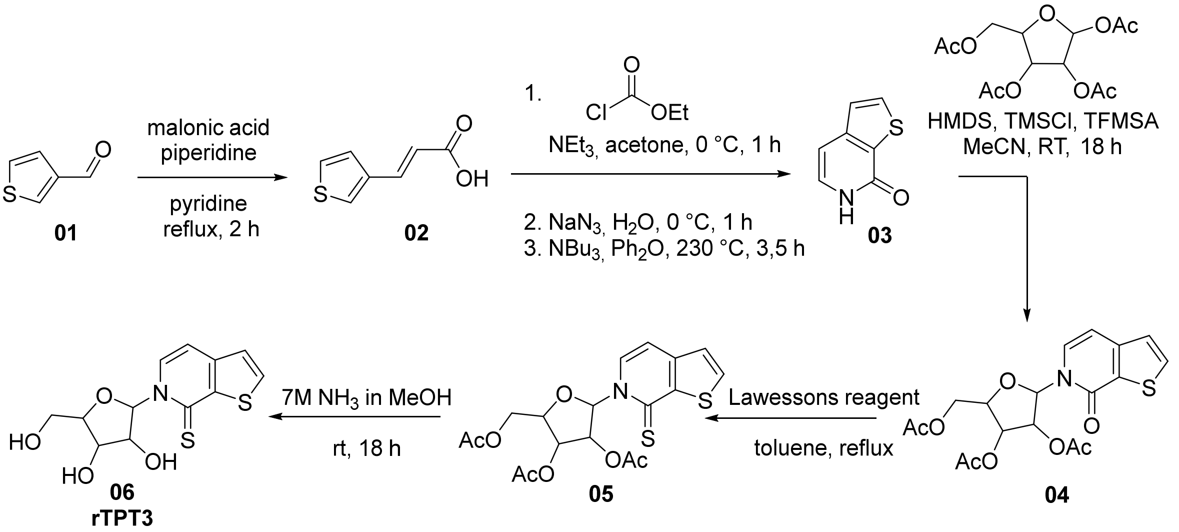

Supplementary Figure 73. rTPT3 was synthesized according to literature protocols.

Synthesis of rNaM-nucleoside

rNaM was synthesized according to an adapted literature protocol via a Ni-catalysed cross-coupling.[15]

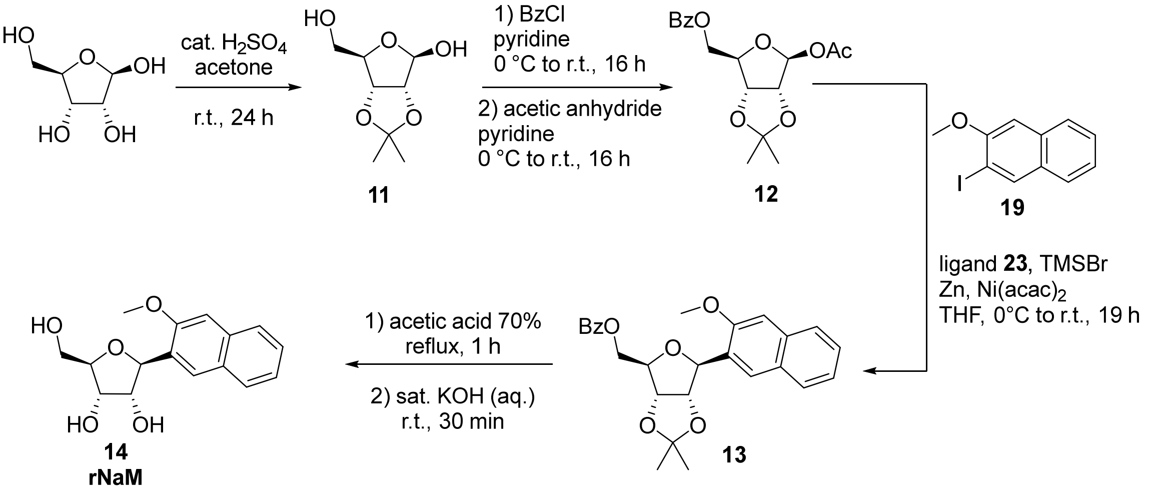

Supplementary Figure 74. rNaM was synthesized according to an adapted literature protocol via a Ni-catalysed cross-coupling.

Ligand **23**, used for the Ni-catalysed cross-coupling, was synthesized according to literature.[15, 16]

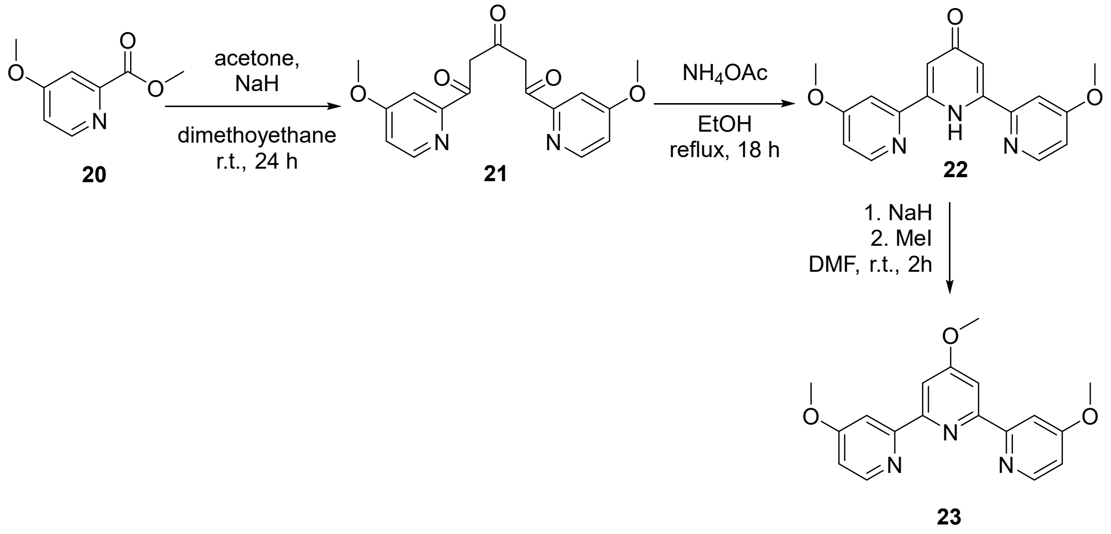

Figure 75: The ligand, used for the Ni-catalysed cross-coupling, was synthesized according to literature.

Phosphoramidite Synthesis

Phosphoramidite synthesis was performed according to literature using a 5'-3'-silyl clamp to achieve selective 2'-TBS protection.[17]

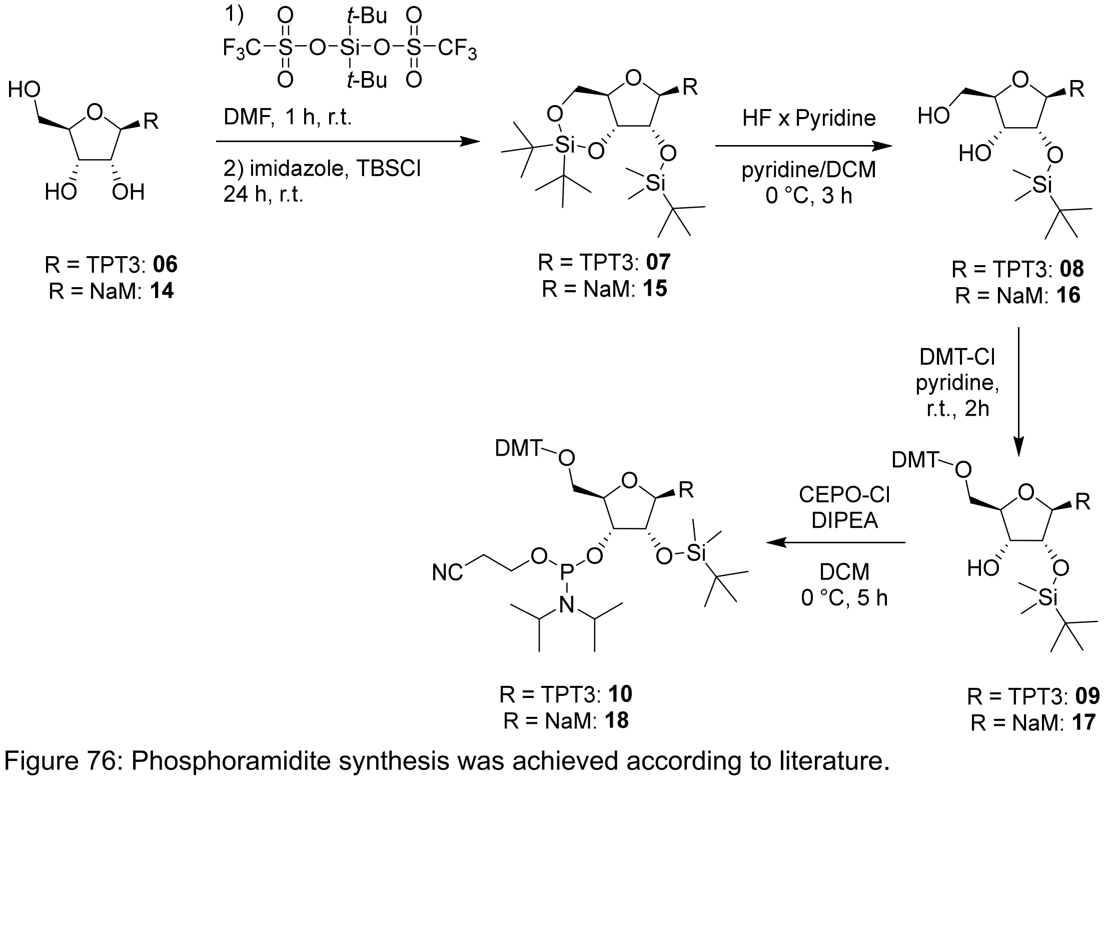

Figure 76: Phosphoramidite synthesis was achieved according to literature.

Synthesis of rTPT3-phosphoramidite

Synthesis of **02**

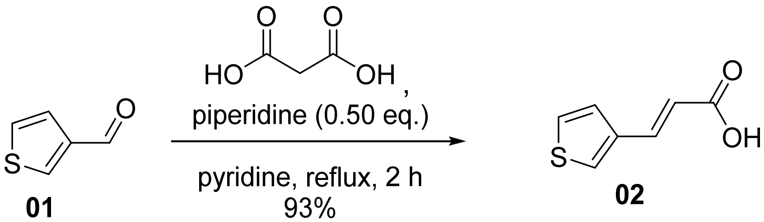

01 **02**

The reaction was carried out according to literature.[1, 18]
3-Thiophencarbaldehyde (**01**) (10.2 g, 91.3 mmol, 1.0 eq.) and malonic acid (9.50 g, 91.3 mmol, 1.0 eq.) were dissolved in pyridine (100 mL). After addition of piperidine (4.52 mL, 46.6 mmol, 0.5 eq.) the mixture was heated to reflux for 2 h. After allowing the mixture to cool to ambient temperature it was poured into ice-cold conc. HCl (100 mL). The mixture was then carefully extracted with EE (3x150 mL), dried over $MgSO_4$, and concentrated *in vacuo*. The product **02** was received as a colourless solid (13.09 g, 84.9 mmol, 93%).

^{1}H NMR (500 MHz, $CDCl_3$): δ [ppm] 11.61 (s, 1H, H-10), 7.78 (d, *J* = 15.9 Hz, 1H, H-7), 7.55 (dd, *J* = 3.0, 1.3 Hz, 1H, H-3), 7.36 (dd, *J* = 5.2, 2.9 Hz, 1H, H-1), 7.33 (dd, *J* = 5.1, 1.3 Hz, 1H, H-5), 6.28 (d, *J* = 15.9 Hz, 1H, H-6).
^{13}C NMR (126 MHz, $CDCl_3$): δ [ppm] = 173.02 (C-8), 140.59 (C-7), 137.42 (C-4), 129.22 (C-3), 127.32 (C-1), 125.36 (C-5), 117.05 (C-6).
IR ν^{-1} [cm^{-1}] = 2810 (w), 2581 (w), 2118 (w), 1676 (s), 1620 (s), 1427 (m), 1406 (w), 1315 (m), 1283 (s), 1213 (m), 1152 (m), 1040 (w), 978 (m), 937 (s), 868 (s), 772 (s), 721 (s), 662 (s).

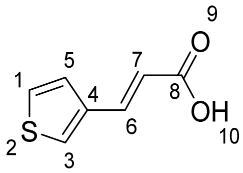

Synthesis of TPT3-nucleobase (**03**)

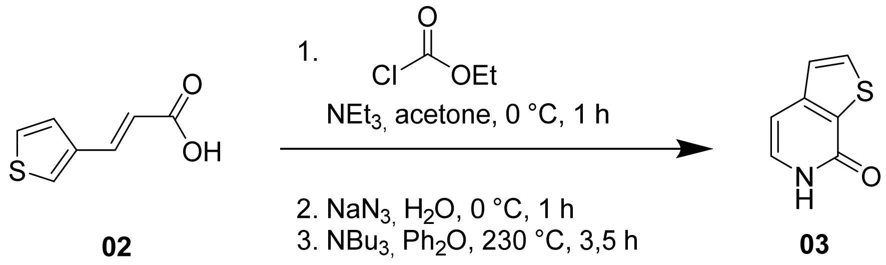

The reaction was carried out according to literature.[1, 18]

02 (12.3 g, 79.8 mmol, 1.0 eq.) was dissolved in acetone (100 mL) and cooled to 0 °C. Then ethyl carbonochloridate (8.35 mL, 87.8 mmol, 1.1 eq.) was added dropwise and stirred for 1 h at 0 °C. At this point a colourless solid precipitate, therefore a strong stirring bar is recommended. After 1 h of stirring NaN_3 (7.78 g, 112 mmol, 1.5 eq.), dissolved in water (20 mL), was added and the mixture was again stirred for 1 h at 0 °C. Then the mixture was treated with water (100 mL). The solids were filtered off, washed thoroughly with water, and dried in high vacuum for several hours. 12.78 g of the intermediate product were obtained and used without further purification and characterisation. Thorough drying is essential for this reaction as residues of water can cause problems while heating up in next reaction step. The intermediate was dissolved in Ph_2O (30 mL), heated to 230 °C and stirred 3.5 h at this temperature. While cooling down, a colourless solid precipitate, which is filtered of, washed with *n*-hexane, and dried under reduced pressure. The crude product was recrystallized in $CHCl_3$/*n*-hexane. The product **03** was obtained as a colourless solid (6.44 g, 42.9 mmol, 54%).

^{1}H NMR (300 MHz, DMSO-*d*$_6$): δ [ppm] = 11.49 (s, 1H), 8.01 (d, J=5.2 Hz, 1H), 7.37 (d, J=5.2 Hz, 1H), 7.26 (d, J=6.9 Hz, 1H), 6.71 (d, J=6.9 Hz, 1H).
^{13}C NMR (75 MHz, DMSO-*d*$_6$): δ [ppm] = 158.53, 146.22, 133.73, 130.16, 129.12, 124.96, 102.17.

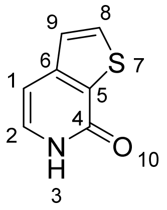

Synthesis of **04**

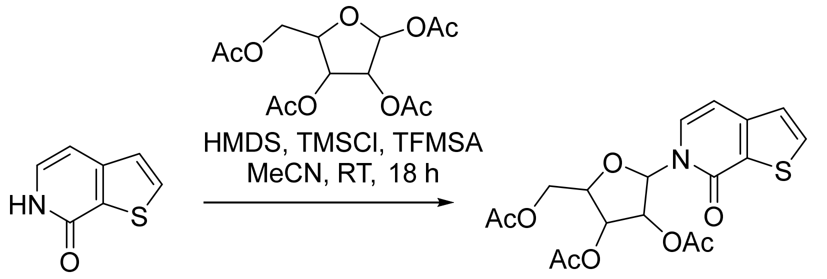

03 **04**

The reaction was carried out according to literature.[19]
TPT3-Base **03** (5.00 g, 33.1 mmol, 1.00 eq.) was dissolved in dry acetonitrile (200 mL). Bis(trimethylsilyl)amine (6.84 mL, 33.1 mmol, 1.00 eq.) and trimethylsilyl chloride (5.07 mL, 39.7 mmol, 1.2 eq.) were added and stirred for 1 h at room temperature. β-D-ribofuranose 1,2,3,5-tetraacetate (12.6 g, 39.7 mmol, 1.2 eq.) followed by trifluoromethanesulfonic acid (3.48 mL, 39.7 mmol, 1.2 eq.) were added and the mixture was stirred over night at room temperature.
Sat. $NaHCO_3$-solution (ca. 220 mL) were added to neutralize the reaction. Phases were separated and the aqueous phase was extracted with dichloromethane (3x 70 mL). Combined organic phases were dried over $MgSO_4$ and concentrated *in vacuo*. The crude product was purified via silica gel chromatography (CH/EE, ½, v/v, R_f = 0.61). Product **04** was received as brownish viscous oil (7.22 g, 17.6 mmol, 54%).

^{1}H NMR (300 MHz, $CDCl_3$): δ [ppm] = 7.71 (d, *J*=5.2 Hz, 1H, H-12), 7.36 (d, *J*=7.4 Hz, 1H, H-6), 7.19 (d, *J*=5.2 Hz, 1H, H-11), 6.69 (d, *J*=7.4 Hz, 1H, H-7), 6.47 (d, *J*=4.5 Hz, 1H, H-1), 5.49 – 5.45 (m, 1H, H-2/3), 5.43 – 5.38 (m, 1H, H-2/3), 4.43 – 4.36 (m, 3H, H-4&H-5), 2.15 (s, 3H, H-14), 2.10 (s, 3H, H-16/18), 2.09 (s, 3H, H-16/18).
^{13}C NMR (75 MHz, $CDCl_3$): δ [ppm] = 170.41 (C-13), 169.73 (C-15/17), 169.67 (C15/17), 158.30 (C-10), 145.27 (C-8), 134.39 (C-12), 127.81 (C-6), 124.39 (C-11), 103.87 (C-7), 87.86 (C-1), 79.59 (C-4), 74.03 (C-3), 70.13 (C-2), 63.22 (C-5), 21.16 (C-14), 20.92 (C-16/18), 20.63 (C-16/18).
HRMS (ESI) *m/z* calcd for $C_{18}H_{19}O_8NNaS$ $[M+Na]^+$: 432.07236 found: 432.07236.
IR: v^{-1} [cm^{-1}] = 3096 (w), 2947 (w), 1740 (s), 1653 (m), 1593 (w), 1435 (w), 1369 (m), 1211 (s), 1094 (m), 1042 (s), 1016 (s), 953 (m), 901 (m), 845 (w), 789 (m), 729 (m), 685 (w), 660 (m), 648 (w), 627 (w), 604 (m).

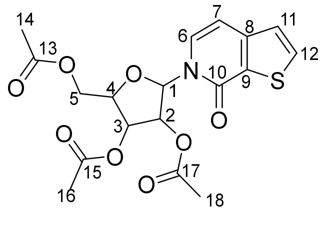

Synthesis of **05**

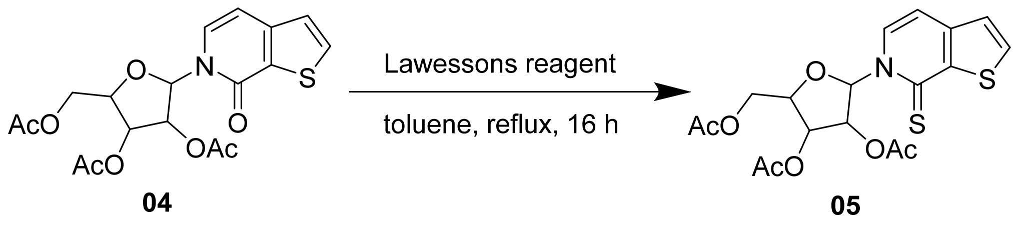

The reaction was carried out analogous to literature.[19]
The equipment was not flame dried and no argon was used. **04** (1.26 g, 3.07 mmol, 1.00 eq.) was dissolved in dry toluene (44 mL). Lawesson´s reagent (1.86 g, 4.61 mmol, 1.5 eq.) was added and the mixture was heated to reflux overnight. After cooling to ambient temperatures, all solids were removed by filtration- Toluene was removed under reduced pressure. The remaining solids were recrystallized from $CHCl_3$/CH. Alternatively purification can be carried out by silica gel chromatography (CH/EE, 3/2, v/v, R_f = 0.29).
Pure product **05** was received as an off-white solid (1.01 g, 2.35 mmol, 77%).

^{1}H NMR (500 MHz, $CDCl_3$): δ [ppm] = 8.03 (d, J=7.3 Hz, 1H, H-11), 7.83 (d, J=5.3 Hz, 1H, H-14), 7.32 (d, J=2.8 Hz, 1H, H-4), 7.29 (d, J=5.3 Hz, 1H, H-15), 7.11 (d, J=7.3 Hz, 1H, H-10), 5.57 (dd, J=5.3 Hz, 2.8 Hz, 1H, H-3), 5.28 (dd, J=7.6 Hz, 5.4 Hz, 1H, H-2), 4.53 (dt, J=7.6 Hz, 3.0 Hz, 1H, H-1), 4.47 (d, J=3.0 Hz, 2H, H-16), 2.20 (s, 3H, H-28), 2.17 (s, 3H, H-24), 2.08 (s, 3H, H-20).
^{13}C NMR (126 MHz, $CDCl_3$): δ [ppm] = 174.52 (C-7), 170.27 (C-27), 169.65 (C-19), 169.33 C-23), 145.92 (C-8), 138.95 (C-9), 138.22 (C-14), 129.12 (C-11), 124.44 (C-15), 108.99 (C-10), 91.89 (C-4), 79.41 (C-1), 74.63 (C-3), 68.62 (C-2), 62.20 (C-16), 20.95 (C-28), 20.65 (C-24), 20.60 (C-20).
HRMS (ESI) *m/z* calcd for $C_{18}H_{19}O_7NNaS_2$ $[M+Na]^+$: 448.04951; found: 448.04975.
IR: v^{-1} [cm^{-1}] = 2972 (m), 2361 (vs), 2342 (vs), 1749 (vs), 1599 (s), 1526 (s), 1439 (s), 1373 (s), 1225 (vs), 1206 (s), 1109 (s), 1090 (s), 1061 (s), 1047 (s).

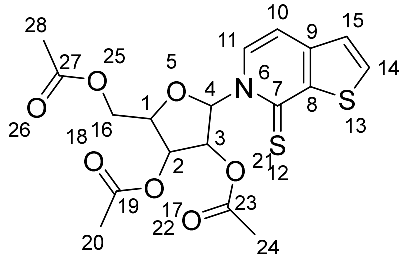

Synthesis of rTPT3-nucleoside (**06**)

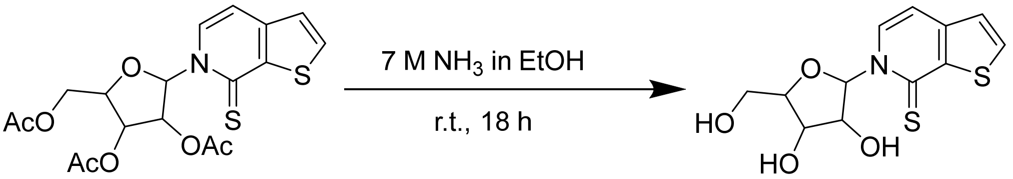

05 **06**

The reaction was carried out analogous to literature.[19]
05 (2.10 g, 4.94 mmol, 1.00 eq.) were treated with 7 M NH_3 in MeOH (35.3 mL, 247 mmol, 50 eq.) and stirred over night at room temperature. Volatiles were removed under reduced pressure and the crude product was purified via silica gel chromatography (DCM/MeOH, 9/1, v/v, R_f = 0.37). rTPT3 (**06**) was received as a colourless solid (1.35 g, 4.51 mmol, 92%)

^{1}H NMR (500 MHz, DMSO-d_6): δ [ppm] = 8.53 (d, J=7.2 Hz, 1H, H-11), 8.18 (d, J=5.3 Hz, 1H, H-14), 7.47 (d, J=5.3 Hz, 1H, H-15), 7.32 (d, J=7.2 Hz, 1H, H-10), 6.98 (d, J=2.5 Hz, 1H, H-4), 5.50 (s, 1H, H-17), 5.32 (t, J=4.9 Hz, 1H, H-19), 5.10 (d, J=5.3 Hz, 1H, H-16), 4.11 – 4.08 (m, 1H, H-3), 4.08 – 4.05 (m, 1H, H-2), 4.03 (dd, J=6.6 Hz, 2.6 Hz, 1H, H-1), 3.85 (dq, J=12.4 Hz, 2.4 Hz, 1H, H-18), 3.70 (dt, J=12.4 Hz, 3.5 Hz, 1H, H-18).
^{13}C NMR (126 MHz, DMSO-d_6): δ [ppm] = 172.77 (C-7), 144.24 (C8/C9), 139.11 (C8/C9), 138.90 (C-14), 131.54 (C-11), 124.93 (C-15), 108.61 (C-10), 93.89 (C-4), 84.40 (C-1), 75.69 (C-3), 68.32 (C-2), 59.52 (C-18).
IR: ν^{-1} [cm^{-1}] = 3319 (w), 2959 (w), 2585 (w), 2471 (m), 2359 (s), 2342 (s), 1734 (w), 1595 (w), 1528 (m), 1431 (s), 1327 (m), 1273 (m), 1128 (s), 1099 (m), 1076 (s), 1042 (s), 1013 (s), 974 (s), 939 (m), 901 (m), 880 (m), 824 (s), 797 (s), 758 (s), 745 (s), 698 (s), 652 (m), 611 (s).
HRMS (EI) *m/z* calcd for $C_{12}H_{13}O_4NNaS_2$ [M+Na]$^+$: 322.01782; found: 322.01835.

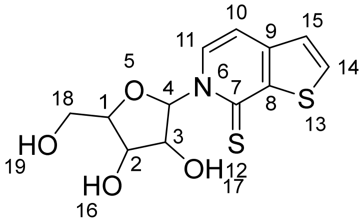

Synthesis of **07**

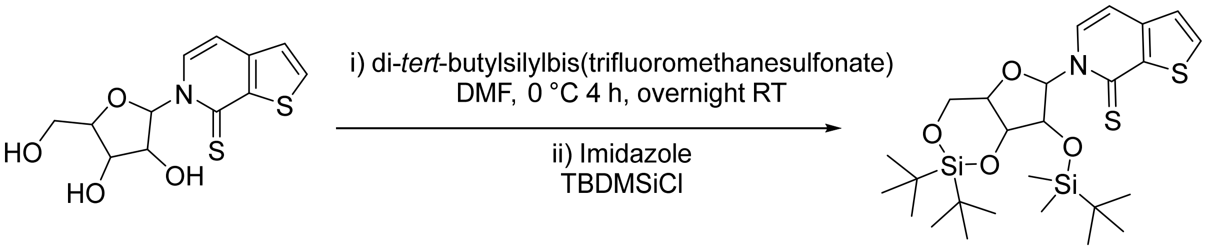

06 **07**

The reaction was carried out in a flame dried 100 ml *Schlenk*-flask under an atmosphere of argon according to literature.[17]

rTPT3-nucleoside 06 (1.20 g, 4.01 mmol, 1.00 eq.) was dissolved in dry acetonitrile (11,2 mL) and cooled to 0 °C. Di-*tert*-butylsilylbistrifluoromethansulfonate (1.96 mL, 6.02 mmol, 1.50 eq.) was added dropwise. The solution was stirred for 3 h at 0 °C followed by addition of imidazole (0.819 g, 12.0 mmol, 3.00 eq.). After stirring 10 min. at room temperature *tert*-butyldimethylchlorosilane (TBSCl) (1.21 g, 8.02 mmol, 2 eq.) was added to the solution. As TBSCl is highly hygroscopic, the reaction should be monitored via TLC and, if necessary, more TBSCl can be added. The mixture was stirred overnight at room temperature.

After adding water (30 mL) a sticky, yellow solid precipitates, which is filtered of, dissolved in dichloromethane, and washed with water two times. The organic phase was dried over $MgSO_4$ and the solvent was removed *in vacuo*. The crude product (3.81 g, >100%) was used in next reaction step without further purification.

Synthesis of **08**

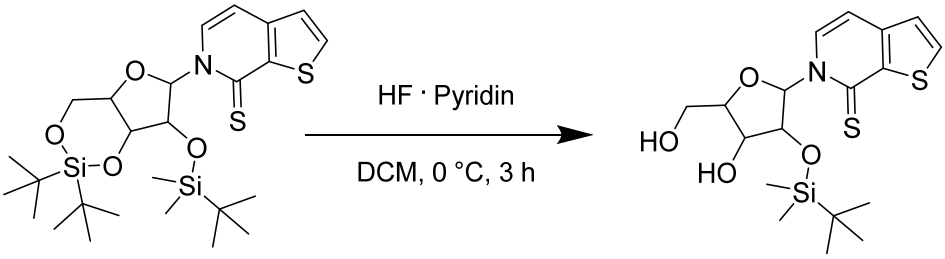

07 **08**

The reaction was carried out in a flame dried 100 ml *Schlenk*-flask under an atmosphere of argon according to literature.[17]
07 (2.20 g, 3.97 mmol, 1.00 eq.) was dissolved in dry dichloromethane (55 mL) and dry pyridine (0.3 mL) and cooled to 0 °C. HF in pyridine (70% w/v, 11.9 mmol, 3 eq.) was carefully added. If residues of TBSCl from previous reaction step is still present, a higher excess of HF in pyridine can be used, therefore the reaction can be monitored via TLC. After stirring 2 h at 0 °C, sat. $NaHCO_3$-solution was added, phases were separated, and the aqueous phase was extracted with dichloromethane (2x 30 mL). Combined organic phases were dried over $MgSO_4$. After removing the solvent *in vacuo,* the crude product was purified via silica gel chromatography (CH_2Cl_2/MeOH, 20/1, v/v, R_f = 0.30).
Final product 08 was received as a pale yellow foam (1.11 g, 2.68 mmol, 68%).
Reported analytical data are in accordance with literature.[17]

^{1}H NMR (600 MHz, acetonitrile): δ [ppm] = 8.39 (d, J = 7.2 Hz, 1H, H-6), 7.95 (d, J = 5.4 Hz, 1H, H-8), 7.37 (d, J = 5.3 Hz, 1H, H-9), 7.24 (d, J = 3.9 Hz, 1H, H-5), 7.21 (dd, J = 7.2, 0.5 Hz, 1H, H-1’), 4.40 (t, J = 4.3 Hz, 1H, H-2’), 4.16 (t, J = 5.1 Hz, 1H, H-3’), 4.12 (dt, J = 5.4, 2.6 Hz, 1H, H-4’), 3.91 (dd, J = 12.3, 2.6 Hz, 1H, H-5’), 3.80 (dd, J = 12.3, 2.7 Hz, 1H, H-5’), 3.40 (s, 1H, 5’-OH), 3.11 (s, 1H, 3’-OH), 0.88 (s, 9H, H-13), 0.04 (s, 3H, H-11/H-11’), 0.01 (s, 3H, H-11/H-11’).
^{13}C NMR (151 MHz, acetonitrile): δ [ppm] = 175.50 (C-2), 145.69 (C-3), 140.08 (C-4) 139.28 (C-8), 132.45 (C-6), 94.09 (C-1’), 85.76 (C-4’), 78.82 (C-2’), 70.90 (C-3’), 61.51 (C-5’), 26.20 (C-13), 18.81 (C-12), -4.37 (C-11/C-11’), -4.67 (C-11/C-11’).
HRMS (ESI) *m/z* calcd for $C_{18}H_{27}O_4NS_2Si$ $[M+H]^+$: 414.1224; found: 414.1227.
IR v^{-1} [cm^{-1}] = 3383 (w), 2949 (w), 2928 (w), 2855 (w), 1601 (w), 1526 (w), 1460 (w), 1437 (s), 1389 (w), 1375 (w), 1362 (w), 1337 (m), 1273 (w), 1252 (m), 1217 (w), 1188 (m), 1130 (s), 1105 (s), 1088 (s), 1047 (s), 995 (w), 974 (s), 939 (w), 916 (w), 889 (w), 829 (s), 812 (s), 779 (s), 739 (m), 704 (s), 669 (m), 635 (w), 608 (m).

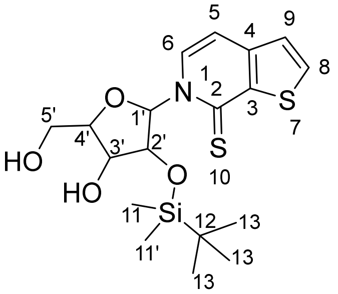

Synthesis of 09

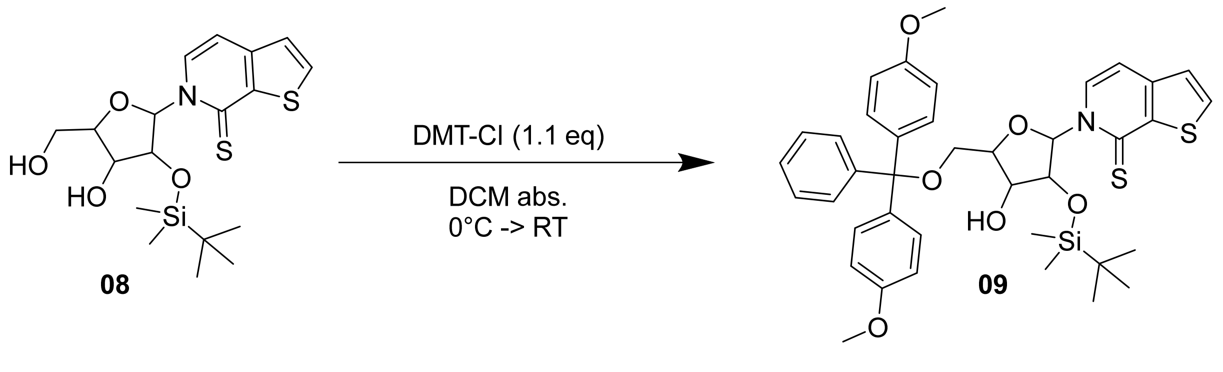

The reaction was carried out in a flame dried 100 ml *Schlenk*-flask under an atmosphere of argon according to literature.[17]
08 (1.10 g, 2.66 mmol, 1.00 eq.) was dissolved in dry pyridine (23 mL). DMAP (292 mg, 2.39 mol, 0.90 eq.) and DMT-Cl (1.08 g, 3.19 mmol, 1.2 eq.) were added. The mixture was stirred over night at room temperature. Methanol (5 mL) was added, followed by sat. $NaHCO_3$-solution. The mixture was extracted with EE (3x 30 mL). Combined organic phases were dried over $MgSO_4$ and concentrated under reduced pressure. The crude product was purified via silica gel chromatography (DCM neat, R_f = 0.40).
Product 09 was received as a colourless foam (888 mg, 1.24 mmol, 47%).

^{1}H NMR (500 MHz, MeCN-*d*$_3$): δ = 8.00 (s, 1H), 7.45 (dd, J=8.4, 1.2, 3H), 7.32 (dd, J=9.0, 0.8, 5H), 7.28 – 7.23 (m, 3H), 7.22 – 7.17 (m, 2H), 6.83 – 6.79 (m, 5H), 5.95 (d, J=5.2, 1H, H-4)), 4.99 (t, J=5.1, 1H, H-3), 4.37 (q, J=4.5, 1H, H-2), 4.17 (q, J=4.1, 1H, H-1), 3.73 (s, 6H, H-44 & H-55), 3.39 (dd, J=10.5, 3.7, 1H, H-9), 3.33 (dd, J=10.5, 4.7, 1H, H-9), 3.29 (d, J=5.0, 1H, H-7), 0.81 (s, 9H, H15-17), -0.03 (s, 3H, H-12/H-14), -0.14 (s, 3H, H-12/H-14).
^{13}C NMR (126 MHz, MeCN-*d*$_3$): δ = 159.62, 159.60, 146.06, 136.80, 131.04, 130.97, 128.98, 128.79, 127.81, 118.29, 114.01, 89.32 (C-4), 87.14, 84.72 (C-1), 76.13 (C-3), 72.20 (C-2), 64.43 (C-9), 55.85 (C-44 & C-55, 26.01 (C-15-17), 18.64 (C-13), -4.72 (C-12/C-14), -4.83 (C-12/C-14).
IR v^{-1} [cm^{-1}] = 2928 (m), 2359 (s), 2332 (m), 1618 (s), 1580 (m), 1508 (s), 1364 (w), 1298 (m), 1248 (s), 1229 (s), 1175 (s), 1136 (m), 1080 (s), 1036 (s), 1005 (m), 910 (m), 833 (s), 781 (s), 648 (m).

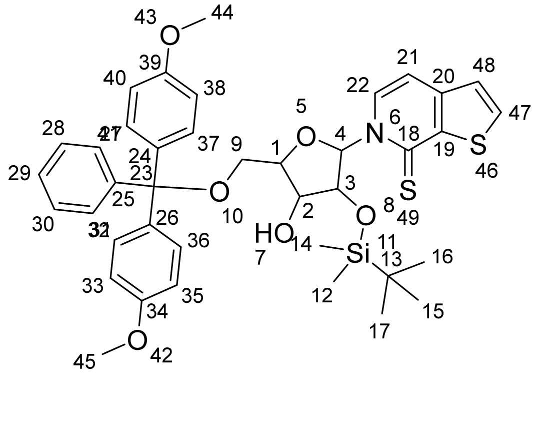

Synthesis of rTPT3-phosphoramidite **10**

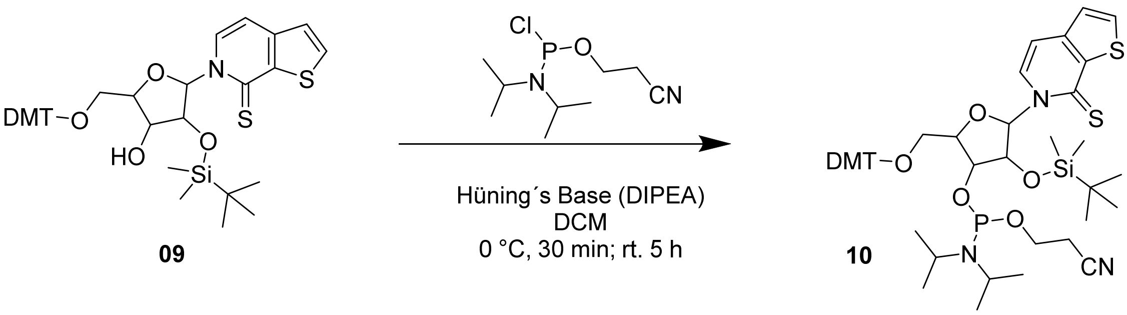

The reaction was carried out in a flame dried 100 ml *Schlenk*-flask under an atmosphere of argon according to literature.[17]

09 (860 mg, 1.20 mmol, 1.00 eq.) and *N,N*-Diisopropylethylamine (2.01 mL, 12.0 mmol, 10 eq.) were dissolved in dry dichloromethane (6 mL). The mixture was degassed three times before 2-Cyanethyl-*N,N*-diisopropylchlorphosphoramidite (670 µL, 3.00 mmol, 2.5 eq.) was added at 0 °C. After stirring for 30 min at 0 °C, the reaction was warmed to room temperature and stirred for additional 5 h. After removing all volatile compounds *in vacuo* (ca. 0.3 mbar, 30 min) the residue was purified via silica gel chromatography (CH/EE, 2/1, v/v, R_f = 0.50).

The final product **10** was received as a colourless foam (974 mg, 1.06 mmol, 89%).

Due to P-inversion and minor unknown impurities NMR-spectra cannot be assigned properly. Therefore, the overall sum of protons and carbon atoms do not match with the chemical formula.

^{1}H NMR (500 MHz, $CDCl_3$ filtered over basic Al_2O_3): δ [ppm] = 8.75 (d, *J*=7.3 Hz, 1H), 8.45 (d, *J*=7.3 Hz, 1H), 7.77 (dd, *J*=6.6 Hz, 5.3 Hz, 2H), 7.54 – 7.51 (m, 3H), 7.47 – 7.44 (m, 3H), 7.43 – 7.39 (m, 5H), 7.35 (d, *J*=2.1 Hz, 2H), 7.33 (dd, *J*=8.6 Hz, 1.9 Hz, 8H), 7.32 – 7.25 (m, 9H), 7.20 (d, *J*=5.3 Hz, 1H), 7.18 (d, *J*=5.3 Hz, 1H), 7.17 – 7.15 (m, 1H), 6.90 (d, *J*=1.0 Hz, 2H), 6.84 (td, *J*=8.6 Hz, 6.3 Hz, 10H), 6.61 (dd, *J*=16.1 Hz, 7.2 Hz, 2H), 4.64 (t, *J*=4.0 Hz, 1H), 4.54 (d, *J*=2.6 Hz, 1H), 4.47 (dt, *J*=8.5 Hz, 2.1 Hz, 2H), 4.44 (d, *J*=3.6 Hz, 0H), 4.42 (t, *J*=3.5 Hz, 1H), 4.40 – 4.34 (m, 3H), 3.81 – 3.78 (m, 12H), 3.70 (dd, *J*=11.1 Hz, 1.9 Hz , 1H), 3.63 – 3.47 (m, 8H), 3.43 (dd, *J*=10.9 Hz, 2.1 Hz, 1H), 2.73 – 2.58 (m, 2H), 2.40 (td, *J*=6.5 Hz, 1.7 Hz, 2H), 1.14 (t, *J*=6.5 Hz, 12H), 1.08 (d, *J*=6.8 Hz, 6H), 0.96 (d, *J*=6.8 Hz, 6H), 0.92 (s, 9H), 0.89 (s, 9H), 0.29 (s, 3H), 0.17 (s, 3H), 0.13 (s, 3H), 0.05 (s, 3H).

^{13}C NMR (126 MHz, $CDCl_3$ filtered over basic Al_2O_3): ^{13}C NMR (126 MHz, $CDCl_3$) δ 174.90, 173.47, 158.84, 158.83, 158.75, 139.59, 139.08, 138.63, 137.80, 137.31, 135.56, 135.47, 135.46, 135.36, 131.33, 131.19, 130.61, 130.58, 130.44, 129.25, 128.66, 128.48, 128.11, 128.01, 127.97, 127.89, 127.26, 127.23, 127.19, 124.44, 124.40, 117.91, 117.42, 113.40, 113.39, 113.29, 113.25, 108.64, 108.62, 95.18, 92.92, 87.41, 87.26, 82.90, 82.86, 81.62, 77.69, 76.45, 76.42, 72.37, 72.24, 69.59, 69.50, 62.63, 61.17, 58.96, 58.84, 58.66, 58.50, 55.40, 55.38, 43.29, 43.19, 43.14, 43.04, 27.03, 26.12, 25.01, 24.95, 24.85, 24.81, 24.79, 24.75, 24.68, 24.62, 20.70, 20.65, 20.25, 20.19, 18.51, 18.42, -3.56, -3.95, -4.58, -4.75, -4.77.

^{31}P NMR (202 MHz, $CDCl_3$ filtered over basic Al_2O_3): δ [ppm] = 150.07, 149.70.

IR v^{-1} [cm^{-1}] 3105 (w), 2959 (w), 2928 (w), 2857 (w), 2361 (w), 2344 (w), 1607 (m), 1508 (s), 1437 (s), 1337 (w), 1250 (s), 1175 (s), 1036 (s), 976 (s), 829 (s), 779 (s), 702 (s).

Synthesis of rNaM-phosphoramidite

Synthesis of **11**

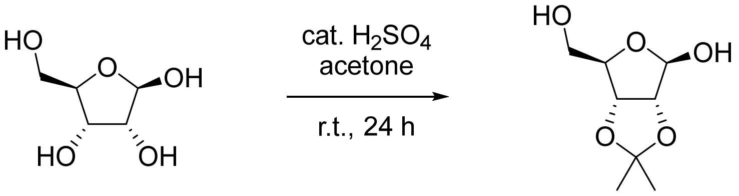

11

The reaction was carried out according to literature.[20]
D-ribose (50.0 g, 333.0 mmol, 1.00 eq.) was suspended in acetone (612 mL). After dropwise addition of conc. H_2SO_4 (1.5 mL) the reaction mixture was stirred at room temperature for 23 h. The reaction mixture was neutralized by addition of sodium bicarbonate. The solvent was evaporated, and the product purified by flash column chromatography (SiO_2, 1:2 CH : EE, R_f = 0.43) to give pure product **11** as a colourless oil (37.28 g, 196.88 mmol, 59 %).

^{1}H NMR (500 MHz, MeOD): δ [ppm] = 5.26 (s, 1H, H-1), 4.77 (d, J=5.9 Hz, 1H, H-3), 4.52 (d, J=6.0 Hz, 1H, H-2), 4.19 (t, J=4.9 Hz, 1H, H-4), 3.64 – 3.57 (m, 2H, H-5), 1.44 (s, 3H, H-7/8), 1.31 (s, 3H, H-7/8).
^{13}C NMR (75 MHz, MeOD): δ [ppm] = 113.16 (C-6), 103.95 (C-1), 88.57 (C-4), 87.90 (C-2), 83.43 (C-3), 64.31 (C-5), 26.74 (C-7/8), 24.95 (C-7/8).
HRMS (EI) *m/z* calcd for $C_7H_{12}O_5$ $[M+H-CH_3]^+$: 175.0601; found: 175.0600.

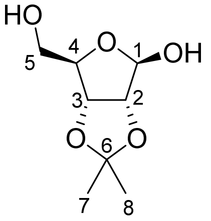

Synthesis of **12**

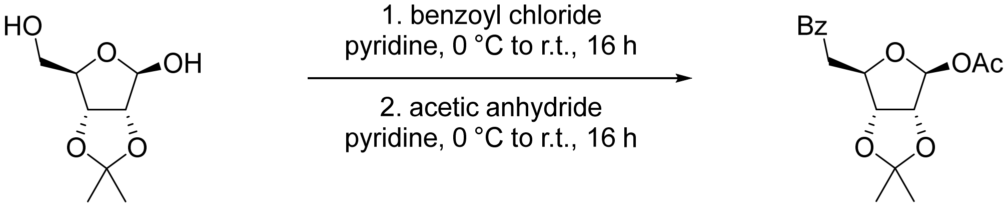

11 **12**

The synthesis was carried out according to literature.[15]

Under inert conditions, acetonide protected D-ribose 11 (37.28 g, 196.0 mmol, 1.00 eq.) was dissolved in dry pyridine (200 mL) and cooled to 0°C. Upon dropwise addition of benzoyl chloride (26 mL, 225.4 mmol, 1.15 eq.) a white precipitate was formed. The reaction mixture was stirred at room temperature overnight. After 24 h water (20 mL) was added, and the reaction mixture was concentrated in vacuo. The residue was solved in ethyl acetate and the organic layer was washed with brine and dried over MgSO4. After evaporation of the solvent under reduced pressure the intermediate product was again dissolved in dry pyridine (300 mL). While stirring at 0°C acetic anhydride (37.0 mL, 392.0 mmol, 2.0 eq.) was added. The reaction mixture was slowly warmed to room temperature and stirred overnight. After evaporation of the solvent under reduced pressure the mixture was coevaporated with toluene two times. After purifying via column chromatography (SiO_2, 10:1 to 2:1 CH: EE, R_f=0.64 (2:1)) pure product **12** was received in 58% yield as an off-white oil that slowly crystalizes.

^{1}H NMR (500 MHz, Chloroform-*d*) δ [ppm] = 8.08 – 8.04 (m, 2H,H-10 & H-10'), 7.60 – 7.55 (m, 1H, H-12), 7.47 – 7.43 (m, 2H, H-11 & H-11'), 6.25 (s, 1H, H-1), 4.85 (d, *J*=5.9 Hz, 1H, H-3), 4.79 (d, *J*=5.9 Hz, 1H, H-2), 4.63 (t, *J*=6.1 Hz, 1H, H-4), 4.42 (dd, *J*=11.6 Hz, 6.5 Hz, 1H, H-5), 4.37 (dd, *J*=11.6 Hz, 5.9 Hz, 1H, H-5), 1.97 (s, 3H, H-14), 1.52 (s, 3H, H-7/7'), 1.35 (s, 3H, H-7/7').

^{13}C NMR (75 MHz, $CDCl_3$): δ [ppm] = 169.42 (C-13), 166.04 (C-8), 133.37 (C-12), 129.75 (C-10 & C-10'), 128.49 (C-11 & C-11'), 113.29 (C-6), 102.38 (C-1), 85.51 (C-4), 85.30 (C-2), 81.66 (C-3), 64.56 (C-5), 26.50 (C-7/7'), 25.12 (C-7/7'), 21.12 (C-14).

IR: $\tilde{\nu}$[cm^{-1}]: 3063 (w), 2988 (w), 2940 (w), 1720 (s), 1601 (w), 1584 (w), 1452 (w), 1373 (w), 1315 (w), 1267 (s), 1225 (s), 1209 (s), 1177 (w), 1159 (w), 1092 (s), 1069 (s), 1026 (s), 1003 (s), 959 (s), 908 (w), 864 (s), 806 (w), 764 (w), 708 (s), 687 (m), 677 (w), 646 (w).

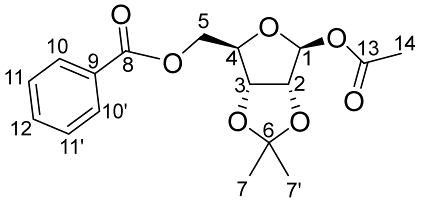

Synthesis of **13**

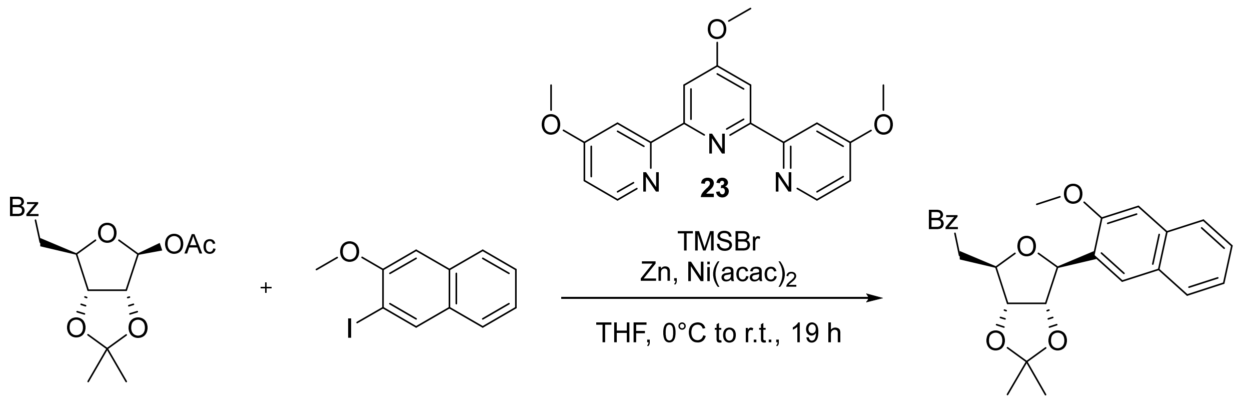

12 **19** **13**

The synthesis was carried out after an adapted protocol from literature.[15]

Under inert conditions, protected ribose **12** (5.70 g, 16.9 mmol, 1.00 eq.), 2-Iodo-3-methoxynaphthalene (19) (6.26 g, 22.0 mmol, 1.30 eq.), Zn (2.21 g, 33.9 mmol, 2.00 eq), $Ni(acac)_2$ (435 mg, 1.70 mmol, 0.10 eq.) and ligand 23 (548 mg, 1.70 eq., 0.10 eq.) were suspended in dry THF (70 ml) and cooled to 0 °C. TMSBr (9.00 mL, 67.8 mmol, 4.00 eq.) was added dropwise and the mixture was stirred overnight in the thawing ice bath.

The mixture was treated with sat. $NaHCO_3$-solution (50 ml) and extracted with DCM (3x 50 ml). The combined organic layers were dried over $MgSO_4$ and concentrated *in vacuo*. The crude product was purified via silica gel chromatography (CH/EE, 3/1, v/v, R_f = 0.43). Pure product **13** was received as a colourless viscous oil (2.12 g, 4.89mmol, 29%).

^{1}H NMR (500 MHz, $CDCl_3$) δ [ppm] = 8.09 – 8.05 (m, 2H, H-19&H-19‘), 7.89 (s, 1H, H-7), 7.71 (dd, *J* = 8.2, 1.1 Hz, 1H, H-12), 7.58 – 7.56 (m, 1H, H-10), 7.55 – 7.53 (m, 1H, H-21), 7.43 – 7.37 (m, 3H, H-11, H-20 & H-20‘), 7.29 – 7.27 (m, 1H), 7.14 (s, 1H, H-14), 5.47 (dd, *J* = 2.9, 1.0 Hz, 1H, H-1), 4.78 (dd, *J* = 6.4, 2.9 Hz, 1H, H-2), 4.77 – 4.73 (m, 2H, H-5 & H-3), 4.59 (dd, *J* = 11.9, 4.9 Hz, 1H, H-5), 4.45 (td, *J* = 5.0, 3.5 Hz, 1H, H-4), 3.97 (s, 3H, H-16), 1.67 (s, 3H, H-22/H-23), 1.38 (s, 3H, H-22/H-23).

^{13}C NMR (126 MHz, $CDCl_3$) δ [ppm] = 166.55 (C-17), 155.30 (C-15), 134.28 (C-13), 133.23 (C-21), 130.00 (C-18), 129.88 (C-19 & C-19‘), 129.49 (C-8), 128.57 (C-6), 128.54 (C-20 & C-20‘), 127.99 (C-10), 126.74 (C-7), 126.54 (C-11), 126.39 (C-12), 123.94 (C-9), 114.33 (C-24), 105.60 (C-14), 86.70 (C-2), 82.67 (C-1), 82.56 (C-4), 81.32 (C-3), 64.57 (C-5), 55.67 (C-16), 27.89 (C-22/23), 25.89 (C-22/23).

HRMS (ESI) *m/z* calcd for $C_{26}H_{26}O_6Na$ $[M+Na]^+$: 457.16216; found: 457.16230.

IR ṽ[cm^{-1}]: 2985 (w), 2928 (m), 2851 (w), 1719 (vs), 1636 (m), 1601 (m), 1450 (m), 1269 (s), 1248 (vs), 1067 (vs), 1024 (s), 860 (m), 708 (vs).

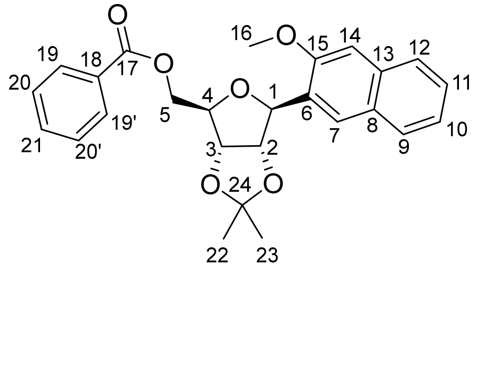

Synthesis of rNaM-nucleoside (**14**)

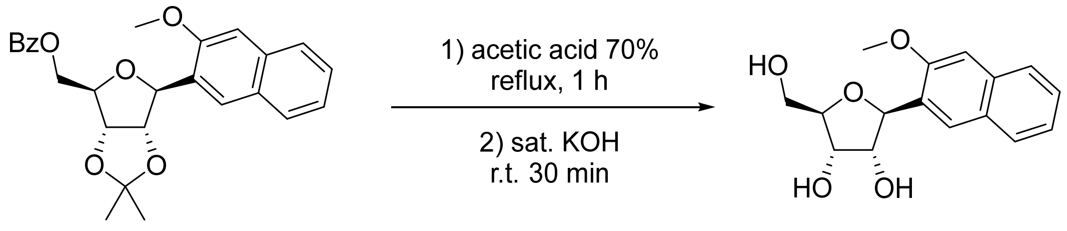

13 **14**

Protected rNaM **13** (4.60 g, 10.6 mmol, 1.00 eq) was dissolved in EE (5 mL) and refluxed with 70% acetic acid (100 mL) for 1 hour. After cooling to 0 °C sat. KOH-solution was added carefully until pH=11 and the mixture was stirred for 30 minutes at room temperature. The mixture was extracted with diethyl ether (3x 50 mL), combined organic layers were dried over $MgSO_4$ and concentrated *in vacuo*. The crude product was purified via silica gel chromatography (neat EE, R_f = 0.28). The final product **14** was received as a colourless powder (2.24 g, 7.72 mmol, 73%).

^{1}H NMR (500 MHz, acetonitrile) δ [ppm] = 7.98 (s, 1H, H-7), 7.81 – 7.78 (m, 1H, H-12), 7.80 – 7.75 (m, 1H, H-9), 7.43 (ddd, J = 8.2, 6.9, 1.3 Hz, 1H, H-11), 7.34 (ddd, J = 8.2, 6.8, 1.3 Hz, 1H, H-10), 7.27 (s, 1H, H-14), 5.13 (dd, J = 3.8, 1.0 Hz, 1H, H-1), 4.04 – 4.02 (m, 1H, H-2), 3.99 – 3.95 (m, 1H, H-3), 3.94 (s, 3H, H-16), 3.92 – 3.87 (m, 1H, H-4), 3.88 – 3.83 (m, 1H, H-5), 3.79 – 3.77 (m, 1H, OH-2), 3.77 – 3.73 (m, 1H, H-5), 3.50 – 3.45 (m, 1H, OH-3), 3.28 – 3.22 (m, 1H, OH-5).
^{13}C NMR (126 MHz, acetonitrile) δ [ppm] = 155.45 (C-15), 134.09 (C-8), 130.49 (C-6), 128.51 (C-13), 127.65 (C-12), 126.34 (C-7/9/11), 126.29 (C-7/9/11), 126.28 (C-7/9/11), 123.80 (C-10), 105.37 (C-14), 83.15 (C-4), 81.71 (C-1), 76.21 (C-2), 70.87 (C-3), 62.16 (C-5), 55.24 (C-16).
HRMS (EI) *m/z* calcd for $C_{16}H_{18}O_5$ $[M]^{\cdot+}$: 290.1149; found: 290.1147, 272.1042, 187.0752, 171.0802, 159.0803, 141.0698, 115.0542.
IR $\tilde{\nu}$[cm^{-1}]: 3356 (br), 3051 (w), 2928 (m), 2874 (w), 1634 (s), 1472 (s), 1248 (vs), 1103 (vs), 1015 (vs).

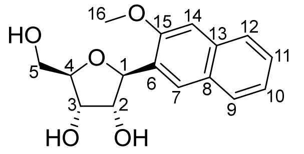

Synthesis of **15**

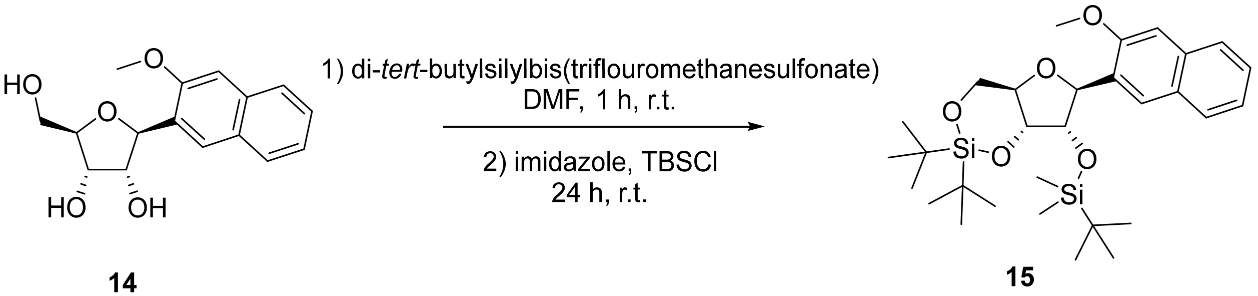

14 **15**

The reaction was carried out according to literature.[17]

Under inert conditions, rNaM (**14**) (2.24 g, 7.72 mmol, 1.00 eq.) was dissolved in dry DMF (25 mL) and cooled to 0 °C. Di-*tert*-butylsilylbis(trifluoromethanesulfonate) (3.77 mL, 11.6 mmol, 1.50 eq.) were slowly added and the ice bath was removed. After 1 h of stirring TLC showed complete conversion and the mixture was again cooled to 0 °C. Imidazole (1.58 g, 23.2 mmol, 3.00 eq.) and TBSCl (2.33 g, 15.4 mmol, 2.00 eq.) were added. As a foamy solid built after 15 minutes, additional DMF (25 mL) was added to facilitate stirring. The reaction mixture was stirred in the thawing ice bath overnight.

After adding water (50 mL) the mixture was extracted with EE (3x50 mL), combined organic layers were dried over $MgSO_4$ and concentrated *in vacuo*. The crude product was purified via silica gel chromatography (CH/EE, 10/1, R_f = 0,39). Product **15** was received as a colourless viscous oil (2.37 g, 3.81 mmol, 50%).

^{1}H NMR: (500 MHz, $CDCl_3$) δ [ppm] = 8.10 (s, 1H), 7.78 – 7.71 (m, 2H), 7.43 (ddd, *J*=8.1 Hz, 6.8 Hz, 1.3 Hz, 1H), 7.34 (ddd, *J*=8.1 Hz, 6.9 Hz, 1.2 Hz, 1H), 7.12 (s, 1H), 5.34 (s, 1H), 4.63 (dd, *J*=8.7 Hz, 4.7 Hz, 1H), 4.31 – 4.17 (m, 3H), 4.00 (dd, *J*=9.4 Hz, 4.2 Hz, 1H), 3.95 (s, 3H), 1.06 (s, 9H), 1.02 (s, 9H), 0.98 (s, 9H), 0.17 (s, 3H), 0.15 (s, 3H).

^{13}C NMR: (126 MHz, $CDCl_3$) δ [ppm] = 166.11, 154.86, 134.39, 134.00, 130.63, 129.64, 128.64, 128.03, 126.48, 126.39, 126.34, 126.33, 123.99, 105.18, 85.41, 85.37, 73.86, 68.88, 55.28, 55.24, 39.06, 27.74, 27.28, 26.09, 26.08, 22.87, 20.50, 18.45, -3.77, -4.87.

HRMS (ESI) *m/z* calcd for $C_{30}H_{48}O_5NaSi_2$ $[M+Na]^+$: 567.29325; found: 567.29320.

IR: ṽ [cm^{-1}]: 2932 (w), 2859 (w), 1466 (m), 1360 (w), 1246 (m), 1198 (w), 1140 (m), 1107 (m), 1055 (s), 997 (s), 932 (m), 901 (w), 829 (s), 781 (s), 745 (s), 687 (m).

Synthesis of **16**

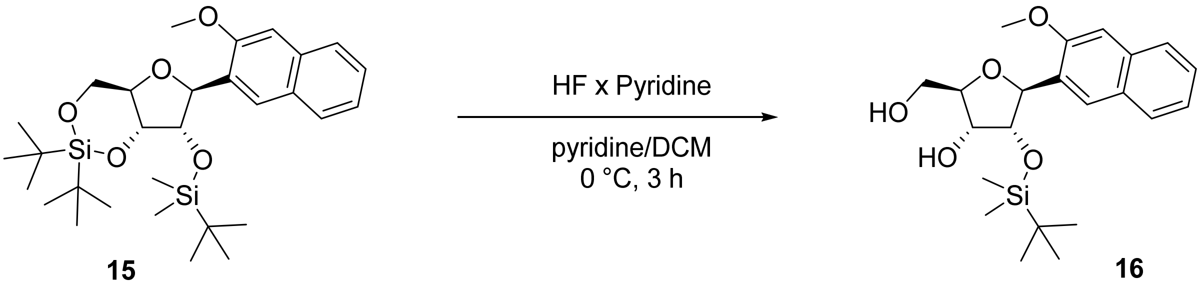

The reaction was carried out according to literature.[17]
Under inert conditions, **15** (2.37 g, 4.35 mmol, 1.00 eq.) was dissolved in DCM (65 mL) and pyridine (2.2 mL) and cooled to 0 °C. HF x Pyridine (70% HF, 0.34 mL, 13.0 mmol, 3.00 eq.) was added carefully. Reaction control via TLC was performed each hour and when no full conversion could be detected additional 600 µl HFxPyridine was added (2 times, 1.20 mL, 46.0 mmol, 10.6 eq.). After 3 hours TLC showed complete conversion and sat. $NaHCO_3$-solution (100 mL) was added. The mixture was extracted with DCM (3x60 mL), combined organic layers were dried over $MgSO_4$ and concentrated *in vacuo*. The crude product was purified via silica gel chromatography (CH/EE, 2/1, R_f = 0.23). Product **16** was received as a colourless foam (1.30 g, 2.70 mmol, 63%).
2'-3'-migration could be observed even under slightly basic and acidic conditions. Therefore, for proper analytical data, $CDCl_3$ was filtered over basic Al_2O_3 or other deuterated solvents like DCM were used.

^{1}H NMR (400 MHz, $CDCl_3$ (filtered over basic Al_2O_3)) δ = 7.79 (s, 1H, H-2), 7.76 (d, *J*=8.0 Hz, 1H, H-4), 7.74 (d, *J*=8.1 Hz, 1H, H-7), 7.46 (ddd, *J*=8.2 Hz, 6.9 Hz, 1.3 Hz, 1H, H-6), 7.36 (ddd, *J*=8.1 Hz, 6.9 Hz, 1.2 Hz, 1H, H-5), 7.17 (s, 1H, H-9), 5.05 (d, *J*=5.6 Hz, 1H, H-1'), 4.50 (t, *J*=5.6 Hz, 1H, H-2'), 4.18 (q, *J*=5.2 Hz, 1H, H-3'), 4.13 (dt, *J*=4.8 Hz, 3.0 Hz, 1H, H-4'), 4.00 (t, *J*=3.1 Hz, 1H, H-5'), 3.96 (s, 3H, H-11), 3.85 (ddd, *J*=11.9 Hz, 8.7 Hz, 3.2 Hz, 1H, H-5'), 2.82 (d, *J*=5.3 Hz, 1H, OH-3'), 2.80 (t, *J*=3.5 Hz, 1H, OH-5'), 0.85 (s, 9H, H-13), -0.07 (s, 3H, H12'), -0.17 (s, 3H, H-12).
^{13}C NMR (101 MHz, $CDCl_3$ (filtered over basic Al_2O_3)) δ 155.60 (C-10), 134.44 (C-3), 130.06 (C-2), 128.65 (C-8), 127.87 (C-4), 127.72 (C-1), 126.87 (C-6), 126.51 (C-7), 124.26 (C-5), 106.24 (C-9), 84.74 (C-4'), 84.50 (C-1'), 76.23 (C-2'), 72.35 (C-3'), 63.18 (C-5'), 55.36 (C-11), 25.79 (C-13), 18.10 (C-14), -4.70 (C-12), -5.20 (C-12').
HRMS (ESI) *m/z* calcd. for $C_{22}H_{32}O_5NaSi$ $[M+Na]^+$: 427.19112; found: 427.19093.
IR: ṽ[cm^{-1}]: 3314 (br), 2955 (w), 2928 (m), 2889 (w), 2855 (w), 2378 (w), 2311 (w), 1472 (m), 1250 (s), 1126 (s), 1115 (s), 1084 (s), 1045 (m), 986 (s), 943 (m), 912 (m), 824 (s), 812 (s), 775 (s), 745 (s), 702 (m), 675 (m).

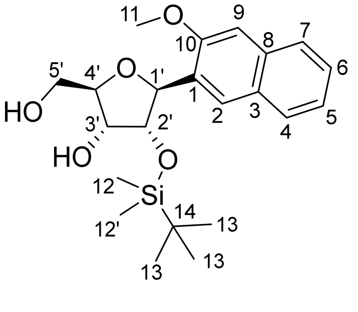

Synthesis of **17**

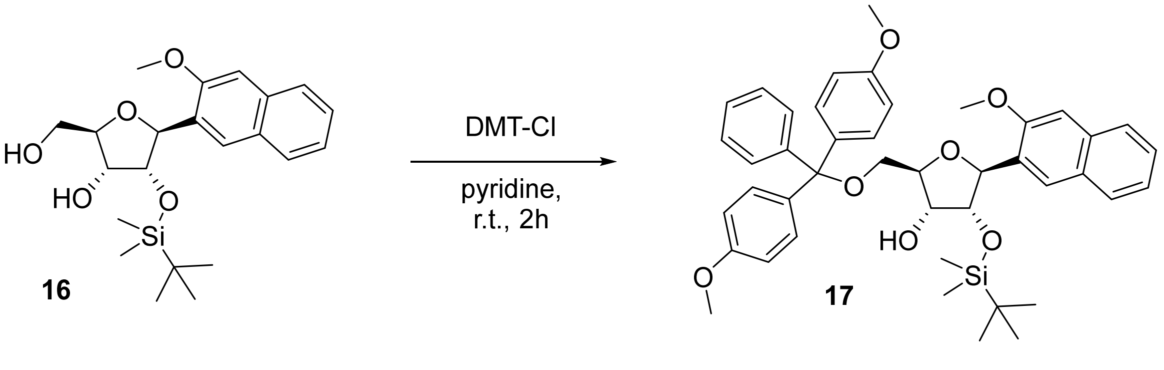

The reaction was carried out according to literature.[17]

Under inert conditions, 2'-TBS protected rNaM (**16**) (1.20 g, 2.97 mmol, 1.00 eq.) was dissolved in dry pyridine (30 mL). DMT-Cl (1.21 g, 3.56 mmol, 1.20 eq.) was added and the solution was stirred for 2 h. Sat. $NaHCO_3$-solution (20 mL) was added, and the mixture was extracted with EE (3x20 mL). Combined organic layers were dried over $MgSO_4$. The crude product was purified via silica gel chromatography (CH/EE, 4/1, R_f = 0,68). Product **17** was received as a colourless foam (2.00 g, 2.83 mmol, 96%).

^{1}H NMR (500 MHz, $CDCl_3$) δ [ppm] = 8.17 (s, 1H), 7.72 (d, *J*=8.1 Hz, 1H), 7.60 (dd, *J*=7.2 Hz, 1.4 Hz, 2H), 7.50 – 7.46 (m, 4H), 7.40 (ddd, *J*=8.2 Hz, 6.8 Hz, 1.3 Hz, 1H), 7.33 (d, *J*=8.0 Hz, 1H), 7.28 (t, *J*=7.5 Hz, 2H), 7.24 – 7.22 (m, 1H), 7.22 – 7.19 (m, 1H), 7.13 (s, 1H), 6.85 – 6.80 (m, 4H), 5.39 (d, *J*=2.6 Hz, 1H), 4.29 (dd, *J*=4.6 Hz, 3.0 Hz, 1H), 4.22 – 4.17 (m, 1H), 4.17 – 4.13 (m, 1H), 3.94 (s, 3H), 3.77 (s, 3H), 3.77 (s, 3H), 3.59 (dd, *J*=10.4 Hz, 2.3 Hz, 1H), 3.47 (dd, *J*=10.3 Hz, 4.5 Hz, 1H), 2.46 (d, *J*=8.1 Hz, 1H), 0.93 (s, 9H), 0.11 (s, 3H), 0.06 (s, 3H).

^{13}C NMR (126 MHz, $CDCl_3$) δ [ppm] = 158.59, 155.19, 145.21, 136.41, 134.08, 130.43, 130.41, 130.18, 128.73, 128.55, 128.30, 128.02, 127.38, 126.87, 126.40, 126.28, 123.73, 113.32, 105.09, 86.39, 82.22, 81.07, 78.42, 71.68, 63.91, 55.33, 55.18, 25.96, 18.24, -4.39, -5.23.

HRMS (ESI) *m/z* calcd for $C_{43}H_{50}O_7NaSi$ $[M+Na]^+$: 729.32180; found: 729.32168.

IR: ṽ[cm^{-1}]: 2928 (w), 2851 (w), 1607 (w), 1508 (m), 1464 (w), 1398 (w), 1302 (w), 1248 (s), 1225 (w), 1175 (s), 1155 (w), 1111 (s), 1092 (m), 1072 (m), 1034 (s), 997 (m), 934 (w), 862 (w), 827 (s), 779 (s), 748 (m), 700 (m), 675 (w).

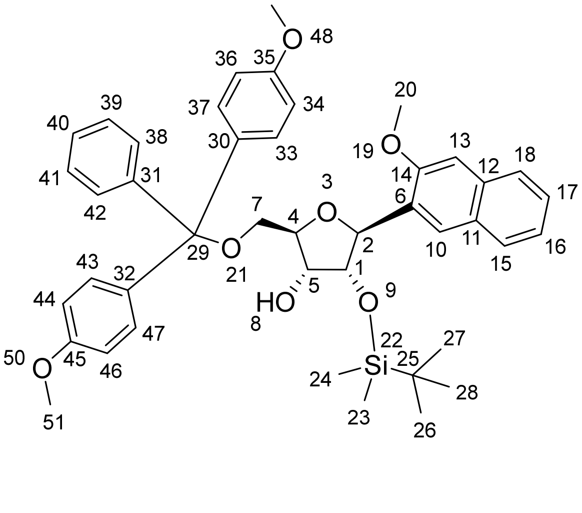

Synthesis of rNaM-phosphoramidite **18**

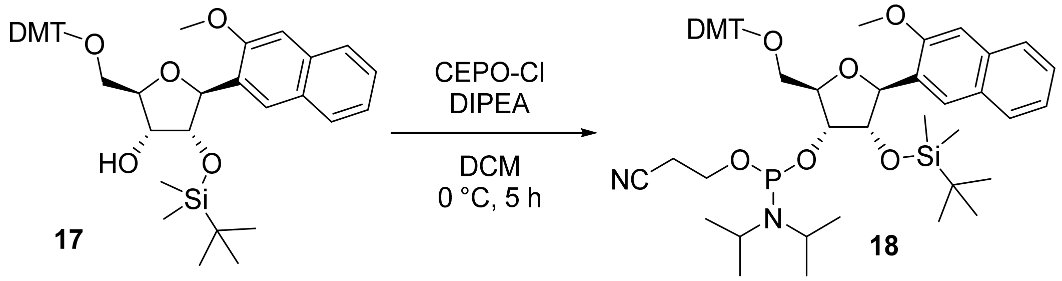

The reaction was carried out according to literature.[17]

Under inert conditions, **17** (1.30 g, 1.84 mmol, 1.00 eq.) was dissolved in dry DCM (17 mL) und treated with diisopropylethylamine (1.28 mL, 7.40 mmol, 4.00 eq.) The solution was degassed 2x before cooling to 0 °C and adding 2-Cyanoethyl-*N,N*-diisopropylchlorphosphoramidite (821 µL, 3.70 mmol, 2.00 eq.). The reaction mixture was stirred 5 hours at 0 °C and was then concentrated *in vacuo* under inert conditions. The crude product was purified via silica gel chromatography (CH/EE, 4/1, +1% Net_3 R_f = 0,44). Product **18** was received as a colourless foam (2.00 g, 2.83 mmol, 96%).

Due to P-inversion and minor unknown impurities the NMR-spectra cannot be assigned properly. Therefore, the overall sum of protons does not match with the chemical formula.

^{1}H NMR (300 MHz, acetonitrile-d_3) δ [ppm] = 8.07 (d, *J*=6.9 Hz, 2H), 7.77 (d, *J*=8.2 Hz, 2H), 7.64 – 7.54 (m, 3H), 7.50 – 7.39 (m, 7H), 7.35 – 7.21 (m, 9H), 6.87 (ddd, *J*=9.0 Hz, 2.9 Hz, 2.0 Hz, 7H), 5.32 (d, *J*=5.2 Hz, 1H), 5.31 – 5.29 (m, 1H), 4.43 (dd, *J*=5.2 Hz, 4.1, 1H), 4.33 (t, *J*=3.7 Hz, 1H), 4.28 (q, *J*=4.7 Hz, 1H), 4.12 (dq, *J*=11.6 Hz, 4.3 Hz, 2H), 3.91 (d, *J*=5.5 Hz, 5H), 3.75 (t, *J*=1.2 Hz, 10H), 3.65 – 3.42 (m, 6H), 3.35 (ddd, *J*=17.0 Hz, 10.4 Hz, 5.1 Hz, 2H), 2.63 (td, *J*=6.2 Hz, 2.2 Hz, 2H), 2.47 (q, *J*=7.1 Hz, 2H), 2.36 (dd, *J*=6.5 Hz, 5.6 Hz, 2H), 1.09 (t, *J*=6.4 Hz, 10H), 0.99 (s, 7H), 0.96 (s, 7H), 0.90 (d, *J*=2.4 Hz, 9H), 0.85 (s, 8H), 0.05 (s, 3H), 0.04 (s, 3H), -0.04 (s, 2H), -0.07 (s, 2H).

^{31}P NMR (122 MHz, acetonitrile-d_3) δ [ppm] = 148.27 (s), 148.17 (s).

IR: ṽ[cm^{-1}]: 3057 (w), 2957 (w), 2928 (w), 2855 (w), 1724 (w), 1607 (w), 1508 (s), 1462 (m), 1447 (w), 1398 (w), 1362 (w), 1335 (w), 1296 (w), 1248 (s), 1227 (w), 1200 (w), 1177 (s), 1157 (m), 1115 (s), 1070 (s), 1032 (s), 1011 (s), 976 (m), 947 (m), 908 (m), 881 (m), 858 (s), 829 (s), 812 (s), 775 (s), 727 (s), 700 (s), 669 (m), 638 (m), 623 (w).

Synthesis of iodinated rNaM-nucleobase

Synthesis of $ZnCl_2$-TMEDA

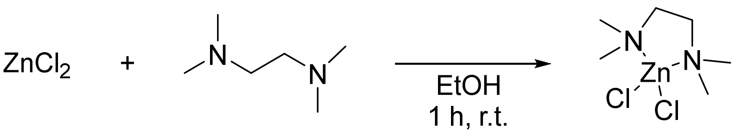

The reaction was carried out according to literature.[21]
$ZnCl_2$ (13.6 g, 99.8 mmol, 1.00 eq.) was dissolved in ethanol (100 mL). TMEDA (13.9 g, 120 mmol, 1.20 eq.) was added and the reaction mixture was stirred for one hour at room temperature. The resulting precipitate was filtered off and washed with cyclohexane. Pure product was received after recrystallization from THF as colourless needles (17.0 g, 67.4 mmol, 68%).

^{1}H NMR (300 MHz, $CDCl_3$) δ [ppm] = 2.73 (s, 4H, H-1), 2.61 (s, 12H, H-2).
^{13}C NMR (75 MHz, $CDCl_3$) δ [ppm] = 56.99 (C-1), 47.88 (C-2).
melting point: 178 °C

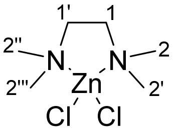

Synthesis of 3-Iodo-2-methoxynaphthalene (**19**):

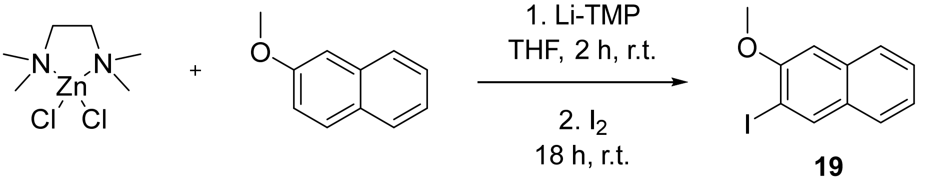

The reaction was carried out according to literature.[22]

Under inert conditions, tetramethylpiperidine (1.33 g, 9.48 mmol, 3.00 eq.) was dissolved in dry THF (9 mL) and cooled to 0 °C. *n*-buthyllithium (1.6 M, 5.92 mL, 9.48 mmol, 3.00 eq.) and $ZnCl_2$·TMEDA (0.79 g, 3.16 mmol, 1.00 eq.) was added. The mixture was stirred for 10 minutes while the colour changes to red. 2-methoxynaphtalene (0.50 g, 3.16 mmol, 1.00 eq.) was added at 0 °C and the mixture was stirred for additional 2 h at room temperature. In a separate *Schlenk flask* iodine (2.40 g, 9.48 mmol, 3.00 eq.) was dissolved in dry THF (15mL). After the 2-methoxynaphtalene mixture completed 2 hours of stirring it was cooled to 0 °C and the iodine solution was added dropwise. Afterwards the mixture was allowed to warm to room temperature and stirred overnight.

The reaction mixture was deactivated by the addition of sat. $Na_2S_2O_3$-solution and afterwards extracted with EE (2x 30 mL) and Et_2O (1x 30 mL). The combined organic phases were dried over $MgSO_4$ and concentrated *in vacuo*. The crude product was purified via silica gel chromatography (pentane/EE, 6/1, v/v, R_f = 0.80). Pure product **19** was received as an off-white solid (0.73 g, 2.57 mmol, 81%).

^{1}H NMR: (300 MHz, $CDCl_3$) δ [ppm] = 8.33 (s, 1H, H-3), 7.72 (d, *J*=8.2 Hz, 1H, H-9 or H-8), 7.67 (d, *J*=8.1 Hz, 1H, H-9 or H-8), 7.46 (ddd, *J*=8.2 Hz, 7.0, 1.2, 1H, H-6), 7.35 (ddd, *J*=8.0 Hz, 7.1 Hz, 1.1 Hz, 1H, H-7), 7.08 (s, 1H, H-10), 3.99 (s, 3H, H-11).

^{13}C NMR: (75 MHz, $CDCl_3$) δ [ppm] = 155.22, 139.36, 134.39, 130.47, 127.02, 126.75, 126.69, 124.40, 105.60, 88.24, 56.47.

GC-MS: (STAND50) T_r = 13.8 min, m/z = 284.10 ($[M]^+$)

IR: ṽ [cm^{-1}]: 3053 (w), 2999 (w), 2959 (w), 2936 (w), 2909 (w), 2868 (w), 2832 (w), 2428 (w), 2290 (w), 1884 (w), 1832 (w), 1809 (w), 1732 (w), 1620 (w), 1582 (m), 1495 (w), 1462 (s), 1427 (m), 1381 (w), 1356 (m), 1321 (m), 1267 (w), 1248 (s), 1213 (s), 1192 (m), 1175 (s), 1148 (w), 1130 (m), 1036 (s), 1018 (s), 966 (m), 949 (m), 883 (m), 858 (s), 826 (s), 791 (m), 743 (s), 704 (s), 671 (m), 621 (m).

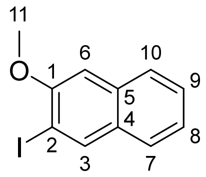

Synthesis of ligand 23 for Ni-catalysed cross-coupling

Synthesis of **21**

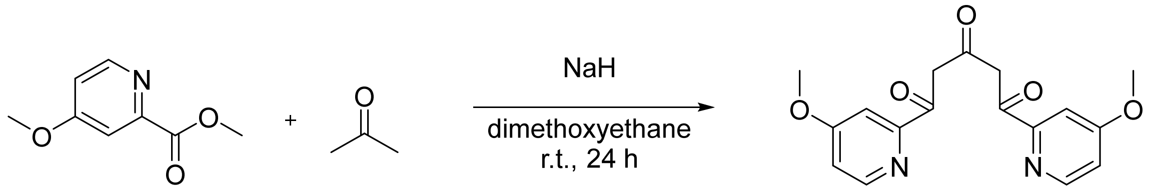

20 **21**

The synthesis was carried out according to literature.[16]

Under inert conditions, NaH (60% in mineral oil, 2.44 g, 61.0 mmol, 5.00 eq.) was suspended in dry dimethoxyethane (25 mL). In a separate *Schlenk* flask methyl-4-Methoxypyridine-2-carboxylate (**20**) (5.01 g, 30.0 mmol, 2.50 eq.) and acetone (0.88 mL, 12.0 mmol, 1.00 eq.) was dissolved in dry dimethoxyethane (30 mL) and was then carefully added to the NaH-suspension. The resulting mixture was stirred for 24 hours at room temperature.

The solvent was evaporated under reduced pressure and the mixture was carefully redissolved in cold water (120 mL) and afterwards acidified with aq. HCl (5M) to ca. pH 6.5. The precipitate was filtered off and washed with water. After freeze drying the product was received as a yellow solid (2.34 g, 7.13 mmol, 24%) and used in the next reaction step without further purification.

IR: $\tilde{\nu}$[cm^{-1}]: 3003 (w), 2953 (w), 2845 (w), 2361 (w), 1628 (w), 1584 (s), 1566 (m), 1487 (w), 1439 (w), 1395 (w), 1377 (w), 1306 (m), 1283 (w), 1260 (w), 1217 (m), 1179 (m), 1042 (m), 1030 (m), 986 (w), 870 (w), 822 (s), 729 (m), 675 (w).

Melting point: 160 °C

Synthesis of **22**

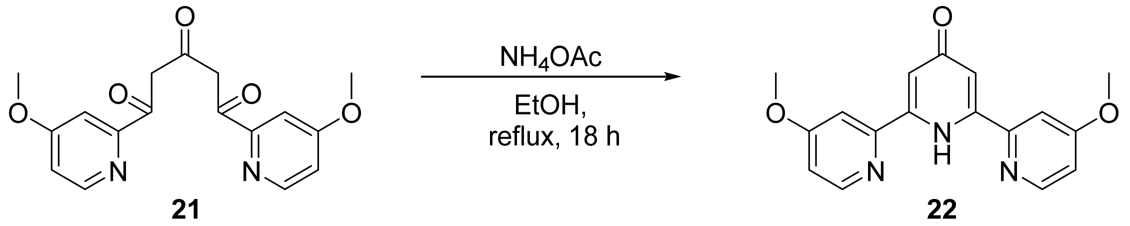

The synthesis was carried out according to literature.[16]

Under inert conditions, triketone **21** (2.00 g, 6.09 mmol, 1.00 eq.) was suspended in dry ethanol (80 mL). Ammonium acetate (9.28 g, 42.6 mmol, 7.00 eq.) was added and the reaction mixture was refluxed for 18 hours. The suspension was cooled to 0 °C, the precipitate filtered off and washed with Et_2O. Pure product **22** was obtained as an orange powder (1.20 g, 3.87 mmol, 64%).

^{1}H NMR (300 MHz, DMSO-d_6) δ [ppm] = 10.93 (s, 1H, H-10), 8.50 (d, *J*=5.6 Hz, 2H, H-3 & H-3'), 8.09 (d, *J*=2.6 Hz, 2H, H-5 & H-5'), 7.85 (s, 2H, H-7 & H-7'), 7.05 (dd, *J*=5.7 Hz, 2.6 Hz, 2H, H-2 & H-2'), 3.95 (s, 6H, H-9 & H-9').

^{13}C NMR (75 MHz, DMSO-d_6) δ [ppm] = 166.14 (C-8), 165.72 (C-1 & C-1'), 156.91 (C-4 & C-4'), 156.18 (C-6 & C-6'), 150.54 (C-3 & C-3'), 110.30 (C-2 & C-2'), 108.43 (C-7 & C-7'), 106.36 (C-5 & C-5'), 55.27 (C-9 & C-9').

IR: $\tilde{\nu}$[cm^{-1}]: 3279 (w), 3235 (w), 3055 (w), 2361 (w), 1639 (m), 1597 (s), 1578 (s), 1560 (s), 1508 (s), 1470 (s), 1441 (s), 1317 (m), 1279 (m), 1221 (w), 1192 (s), 1173 (s), 1105 (m), 1080 (w), 1028 (s), 993 (m), 939 (m), 891 (w), 841 (s), 775 (s).

Melting point: 260 °C

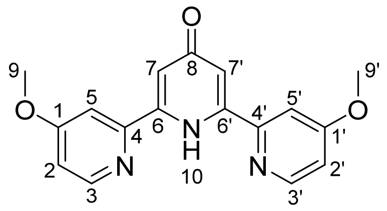

Synthesis of ligand **23**

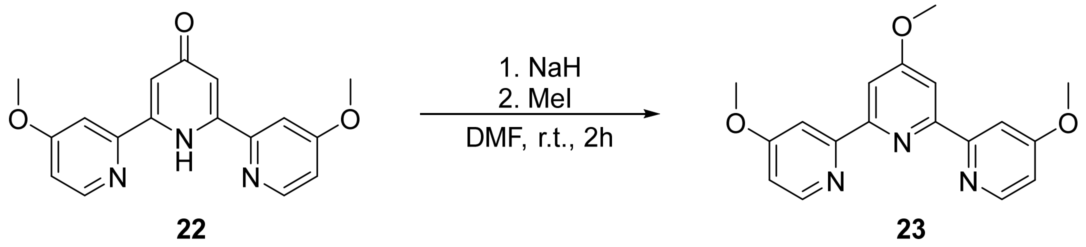

The reaction was carried out according to literature.[16]

Under inert conditions, NaH (60% in mineral oil, 18.6 mg, 0.75 mmol, 1.20 eq.) was suspended in dry DMF (10 mL) and cooled to 0 °C. **22** (200 mg, 0.64 mmol, 1.00 eq.) was added at 0 °C and the mixture was stirred for 20 minutes at room temperature. After again cooling to 0 °C MeI (110 mg, 0.77 mmol, 1.20 eq.) was added. The reaction mixture was stirred for additional two hours at room temperature. Water (20 mL) was added, the resulting precipitate filtered off and washed with cold water. The precipitate was then freeze dried. The final product **23** was received was obtained as an off-white powder (209 mg, 0.64 mmol, 100%).

^{1}H NMR (500 MHz, $CDCl_3$) δ [ppm] = 8.49 (d, *J*=5.5 Hz, 2H, H-3 & H-3'), 8.13 (d, *J*=2.6 Hz, 2H, H-5 & H-5'), 7.99 (s, 2H, H-7 & H-7'), 6.83 (dd, *J*=5.6 Hz, 2.6 Hz, 2H, H-2 & H-2'), 4.00 (s, 3H, H-9), 3.94 (s, 6H, H-10 & H-10').

^{13}C-NMR (75 MHz, $CDCl_3$) δ [ppm] = 167.79 (C-8), 166.55 (C-1 & C-1'), 157.90 (C-4 & C-4'), 156.84 (C-6 & C-6'), 150.31 (C-3 & C-3'), 110.02 (C-2 & C-2'), 107.33 (C-7 & C-7'), 107.17 (C-5 & C-5'), 55.59 (C-9), 55.20 (C-10 & C-10').

IR: ṽ[cm^{-1}]: 3009 (w), 2955 (w), 2922 (m), 2853 (w), 1582 (m), 1560 (s), 1458 (m), 1406 (m), 1379 (m), 1346 (w), 1304 (m), 1288 (m), 1258 (w), 1234 (s), 1155 (w), 1109 (w), 1059 (w), 1026 (s), 988 (m), 881 (s), 858 (s), 841 (s), 820 (s), 777 (m).

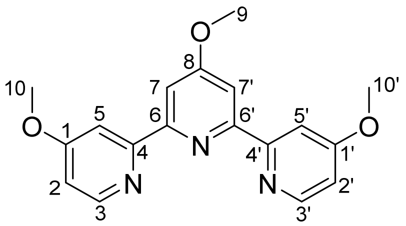

NMR-Spectra

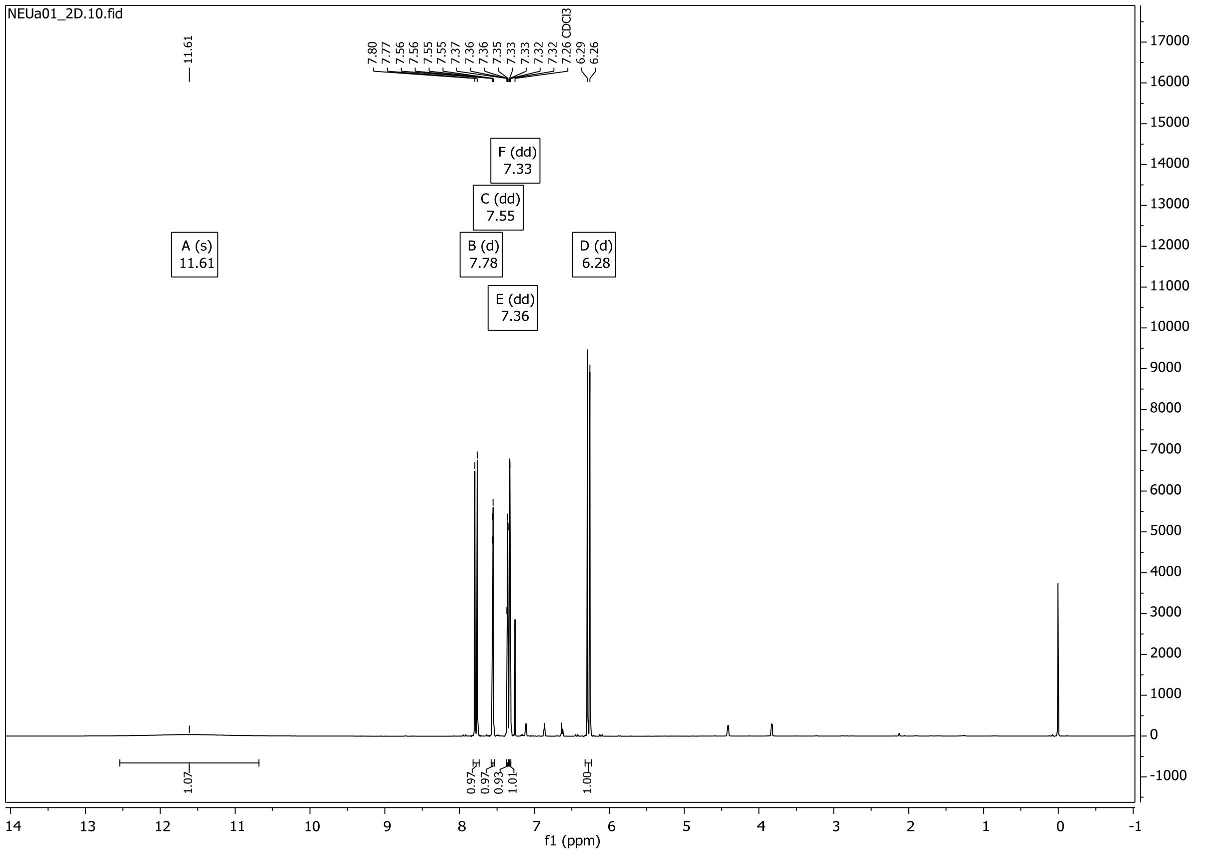

Spectrum 1. ^{1}H-NMR of compound **02** (300 MHz, $CDCl_3$, r.t.).

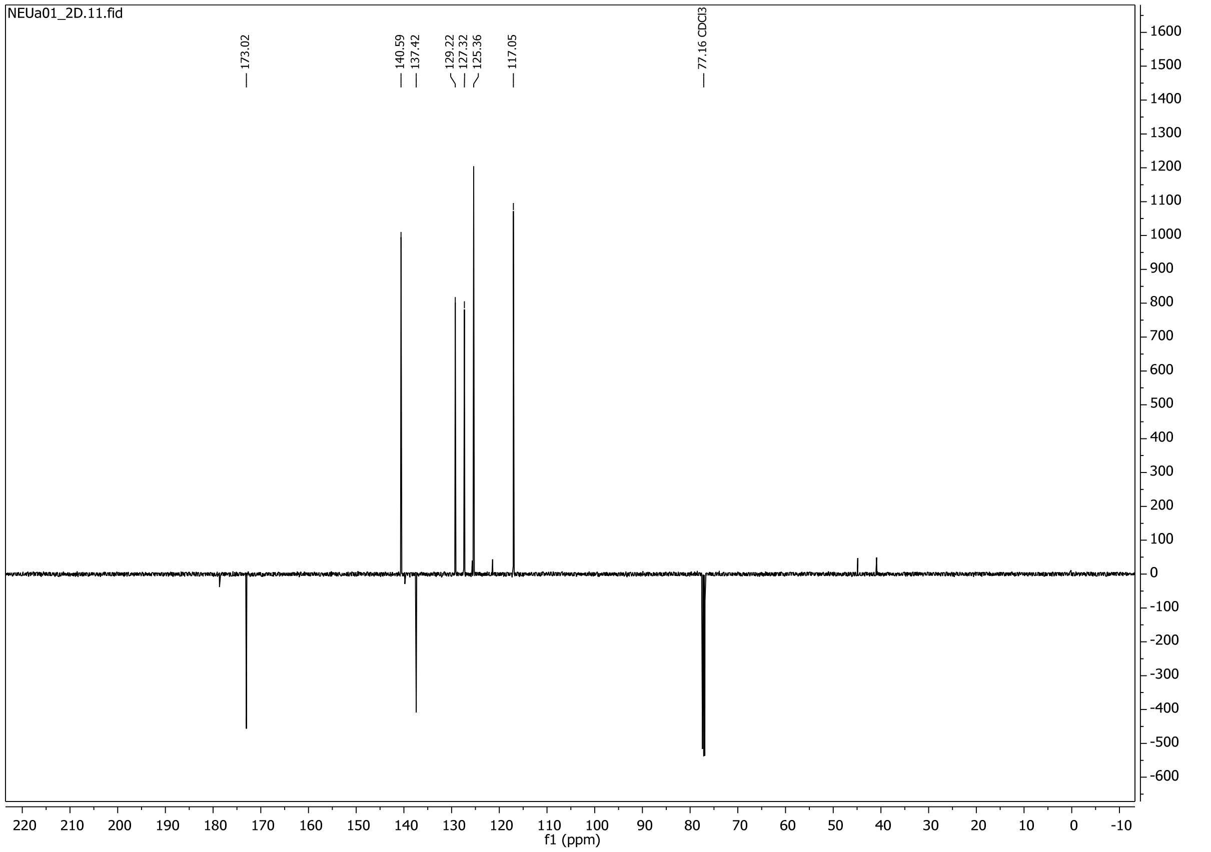

Spectrum 2. ^{13}C-NMR of compound 02 (75 MHz, CDCl3, r.t.).

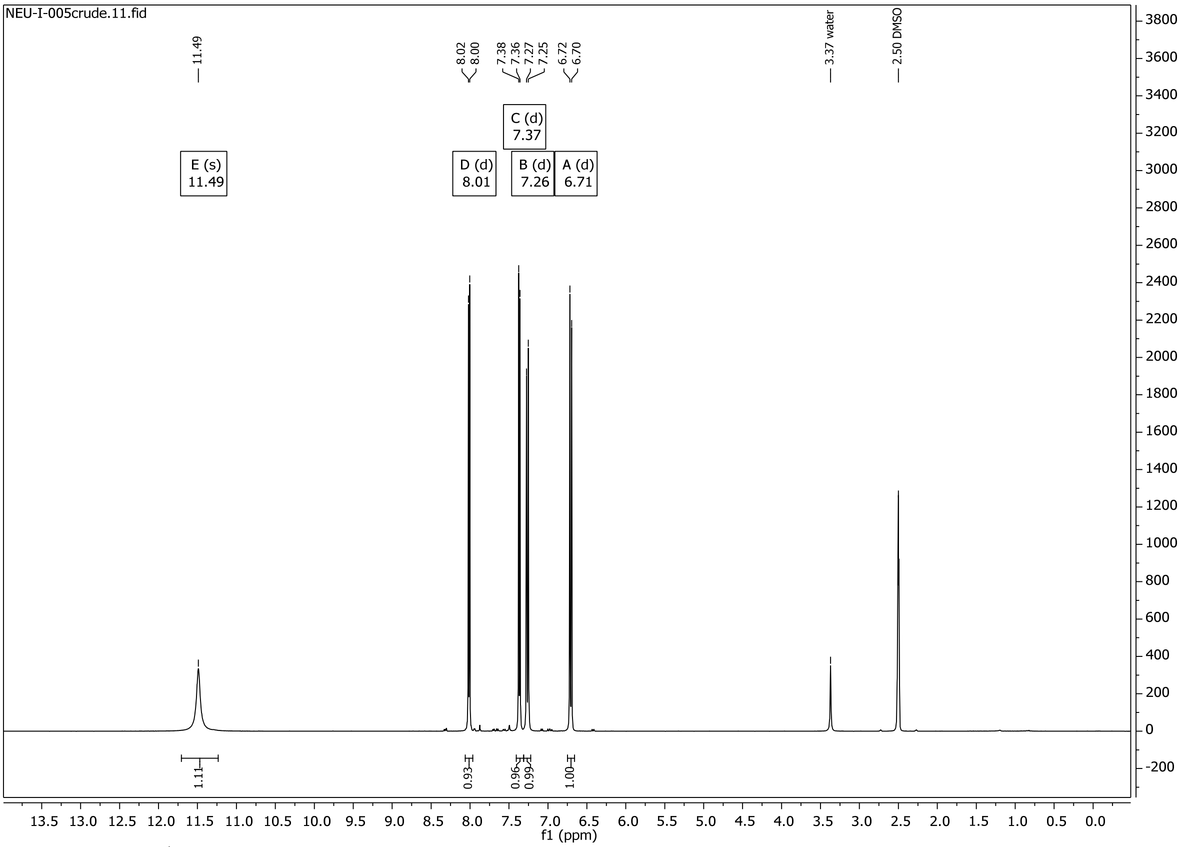

Spectrum 3. ^{1}H-NMR of compound **03** (300 MHz, DMSO, r.t.).

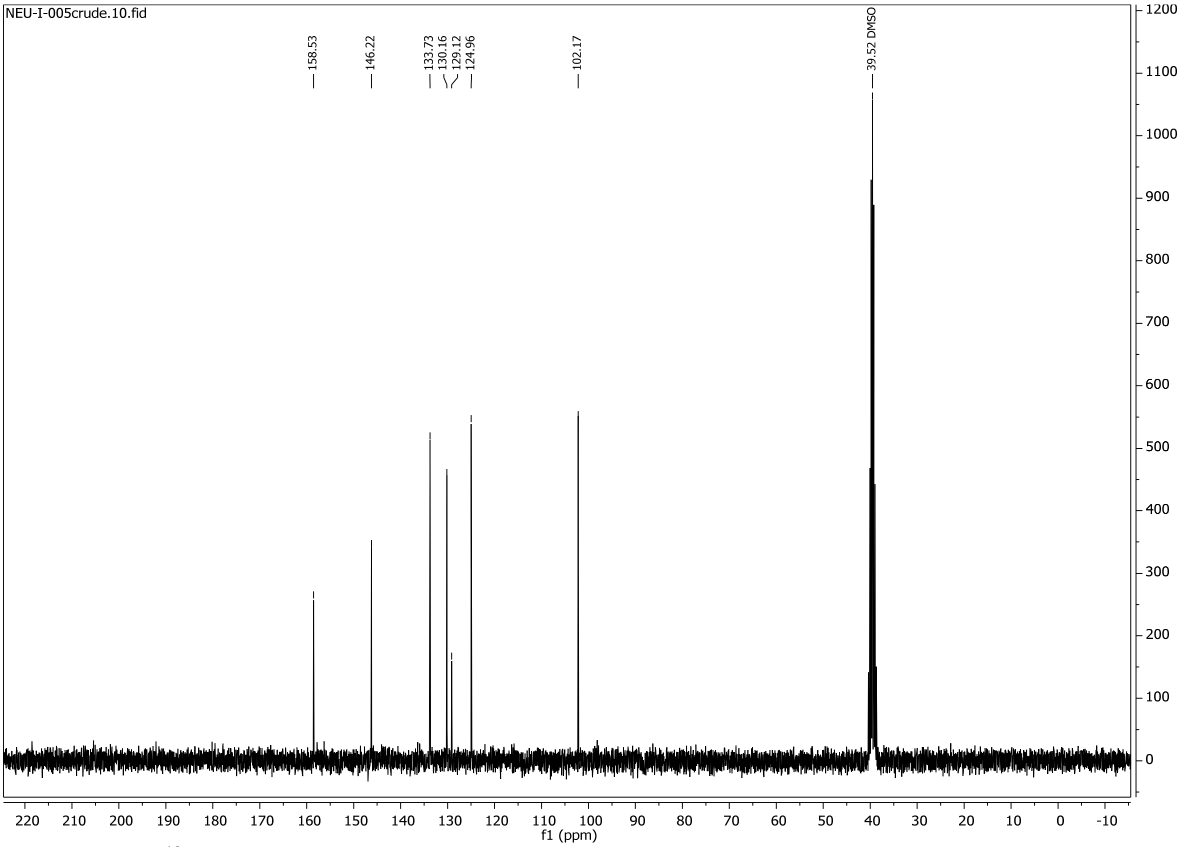

Spectrum 4. ^{13}C-NMR of compound **03** (75 MHz, DMSO, r.t.).

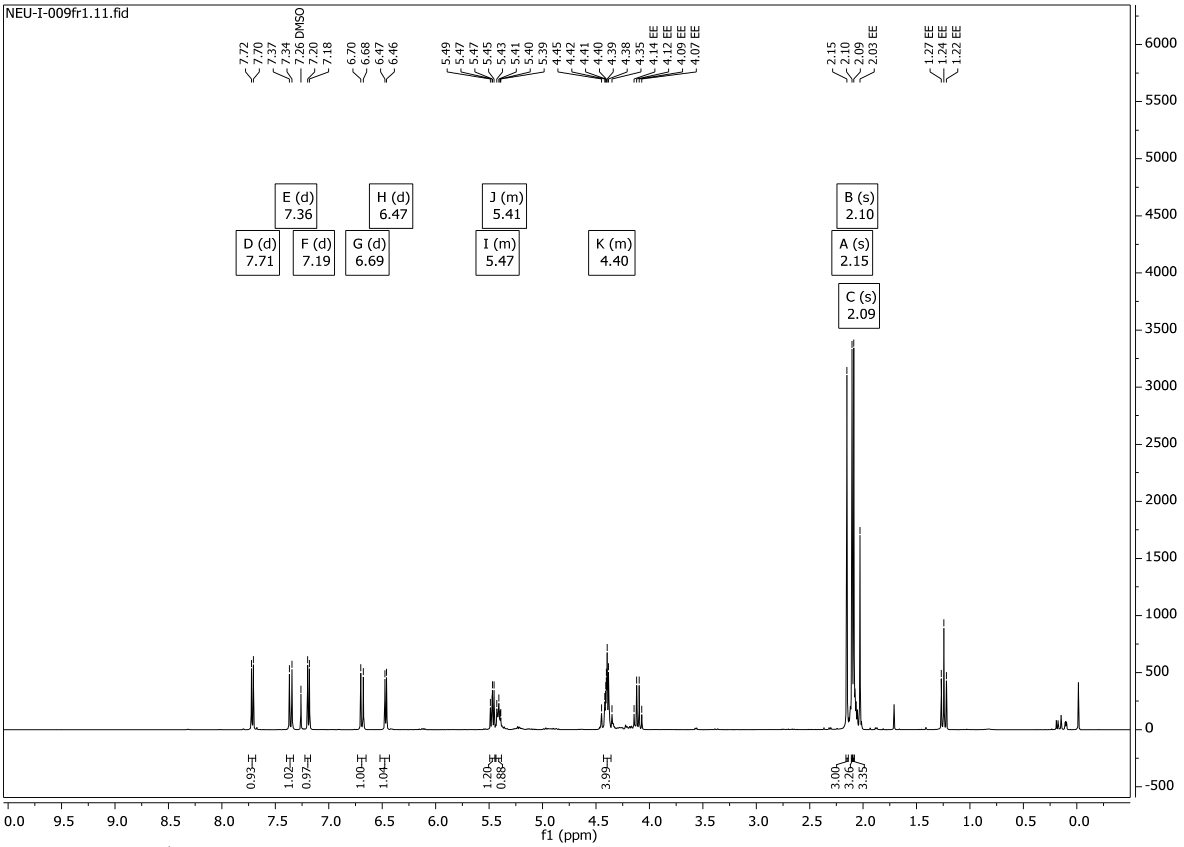

Spectrum 5. ^{1}H-NMR of compound **04** (300 MHz, $CDCl_3$, r.t.).

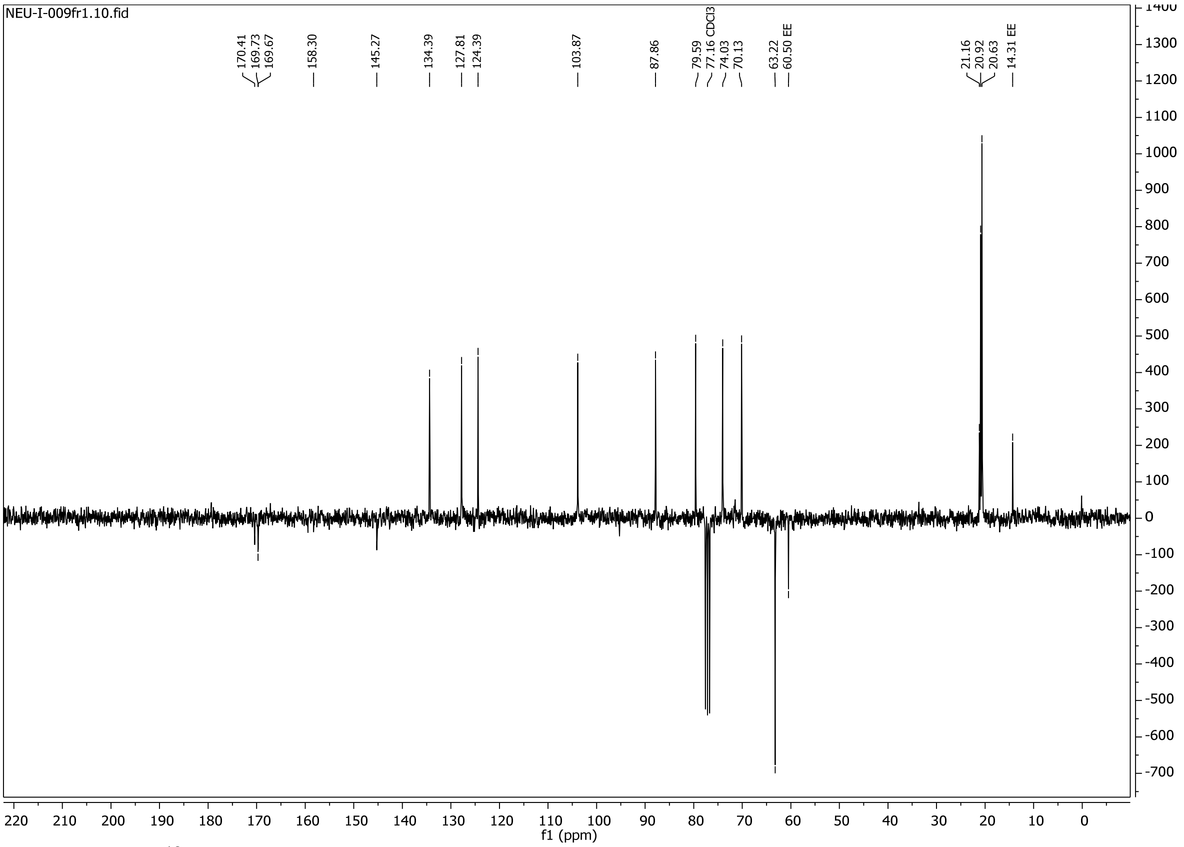

Spectrum 6. ^{13}C-NMR of compound **04** (75 MHz, $CDCl_3$, r.t.).

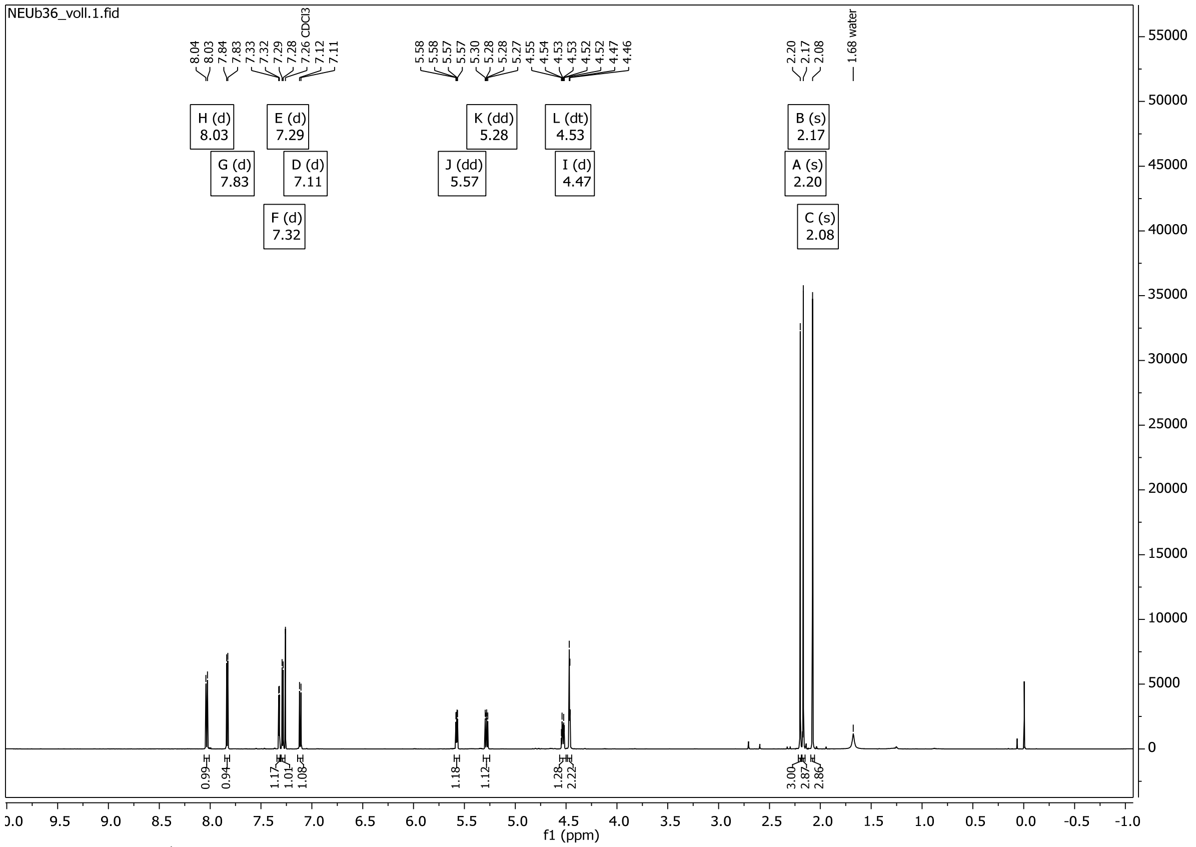

Spectrum 7. ^{1}H-NMR of compound **05** (500 MHz, $CDCl_3$, r.t.).

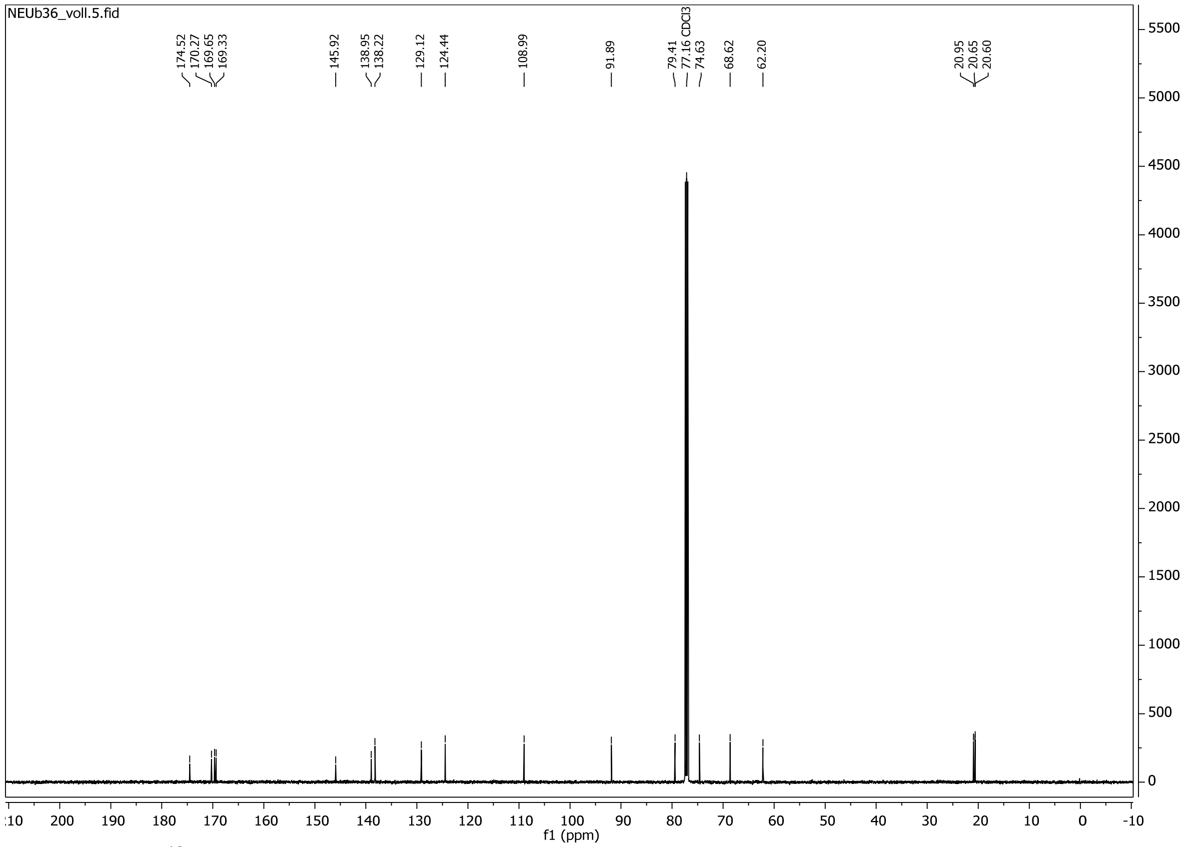

Spectrum 8. ^{13}C-NMR of compound **05** (126 MHz, $CDCl_3$, r.t.).

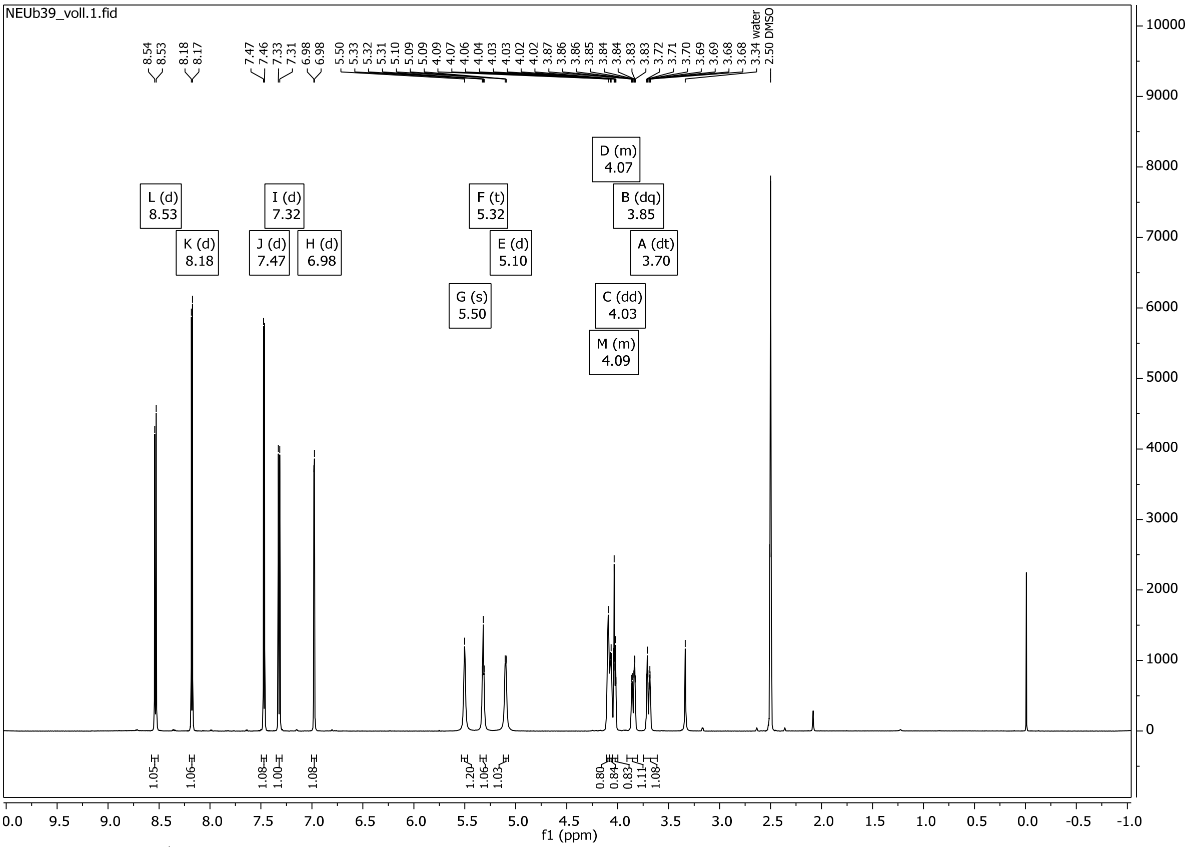

Spectrum 9. ^{1}H-NMR of compound **06** (500 MHz, DMSO, r.t.).

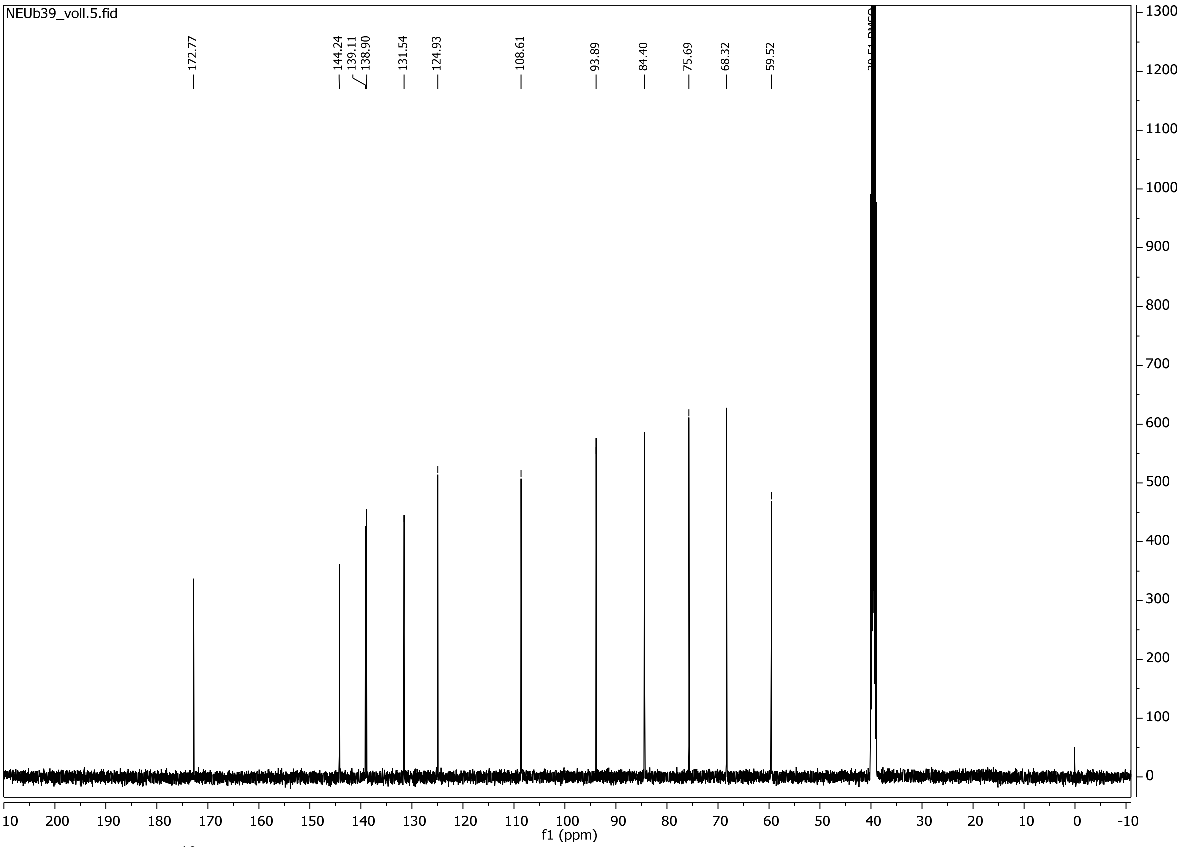

Spectrum 10. ^{13}C-NMR of compound **06** (126 MHz, DMSO, r.t.).

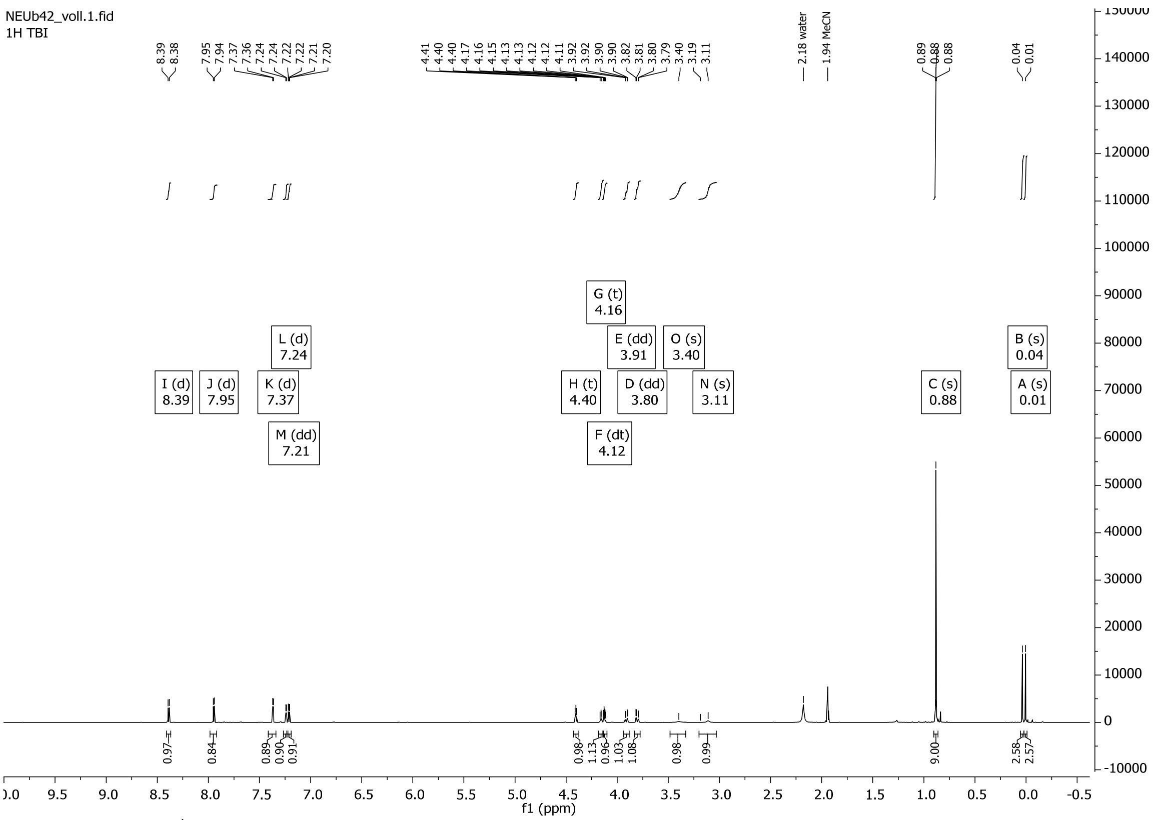

Spectrum 11. ^{1}H-NMR of compound **08** (600 MHz, acetonitrile, r.t.).

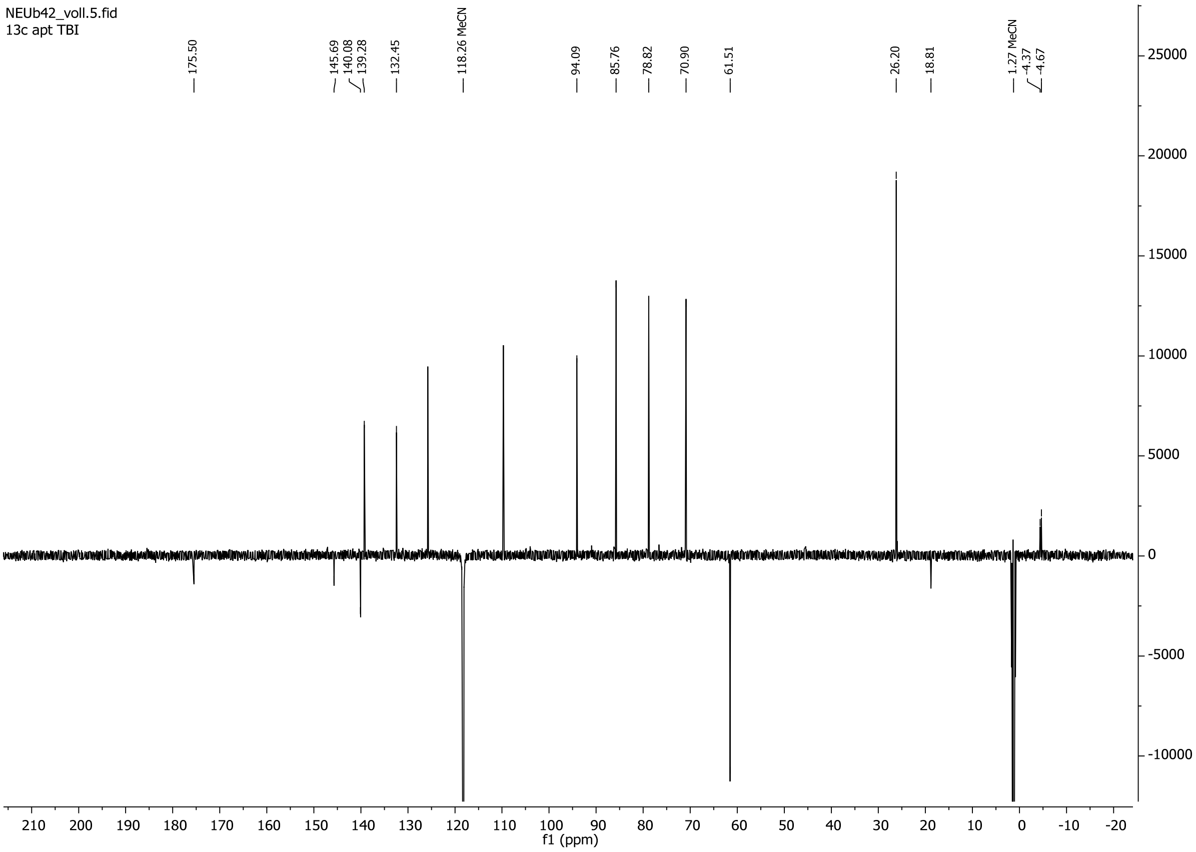

Spectrum 12. ^{13}C-NMR of compound **08** (151 MHz, acetonitrile, r.t.).

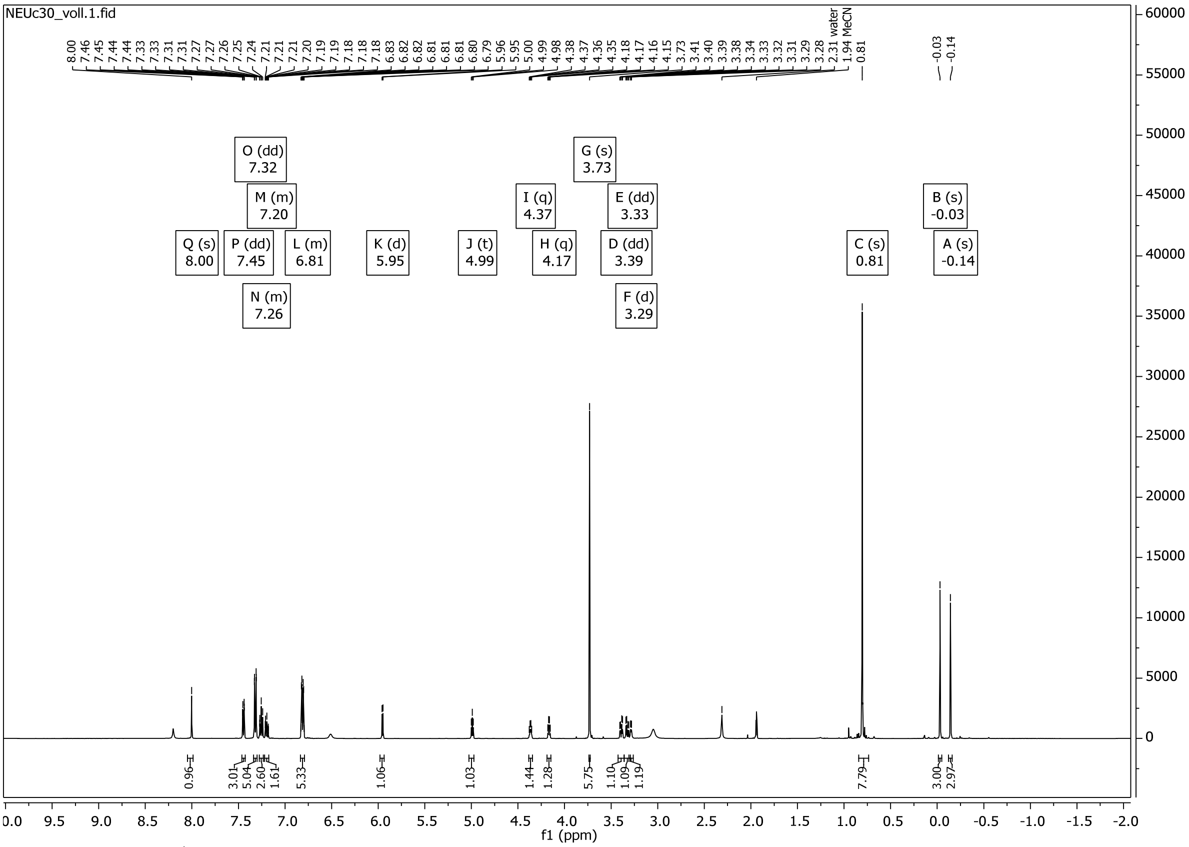

Spectrum 13. ^{1}H-NMR of compound **09** (500 MHz, acetonitrile, r.t.).

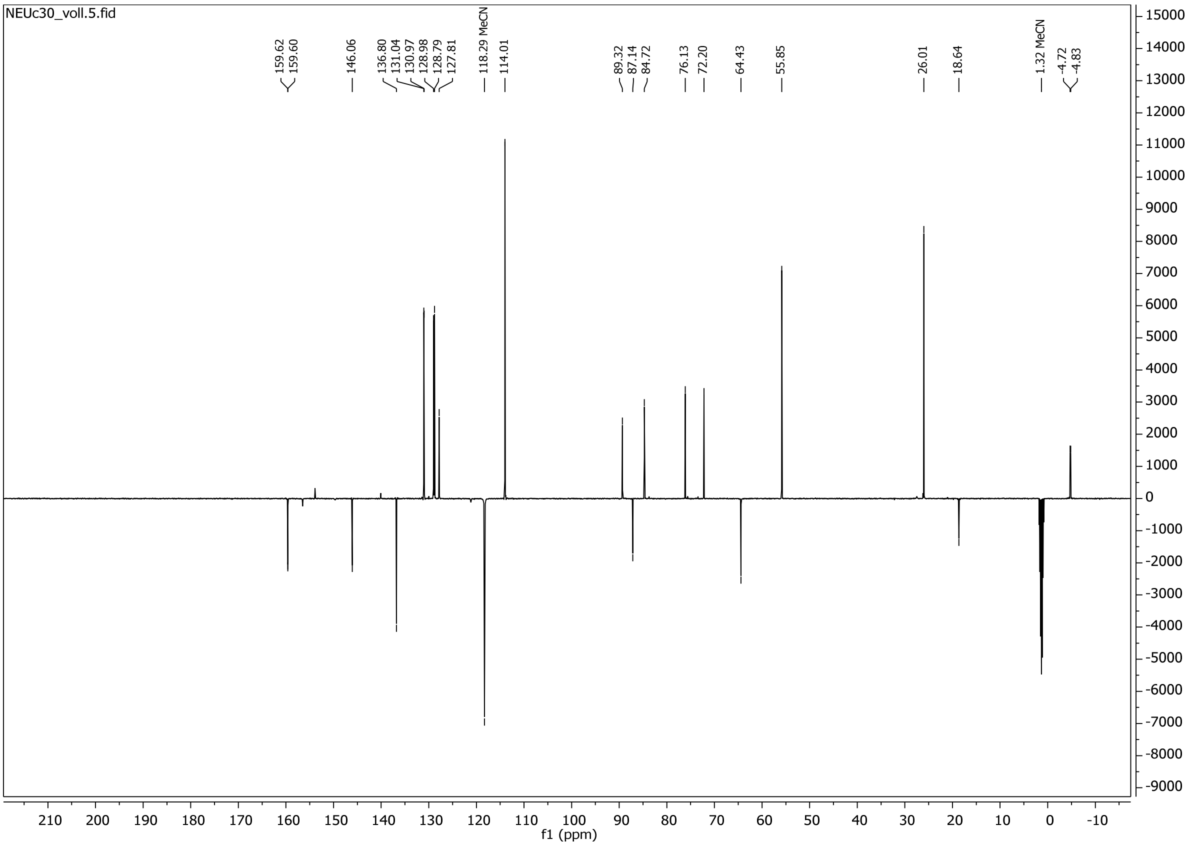

Spectrum 14. ^{13}C-NMR of compound **09** (126 MHz, acetonitrile, r.t.).

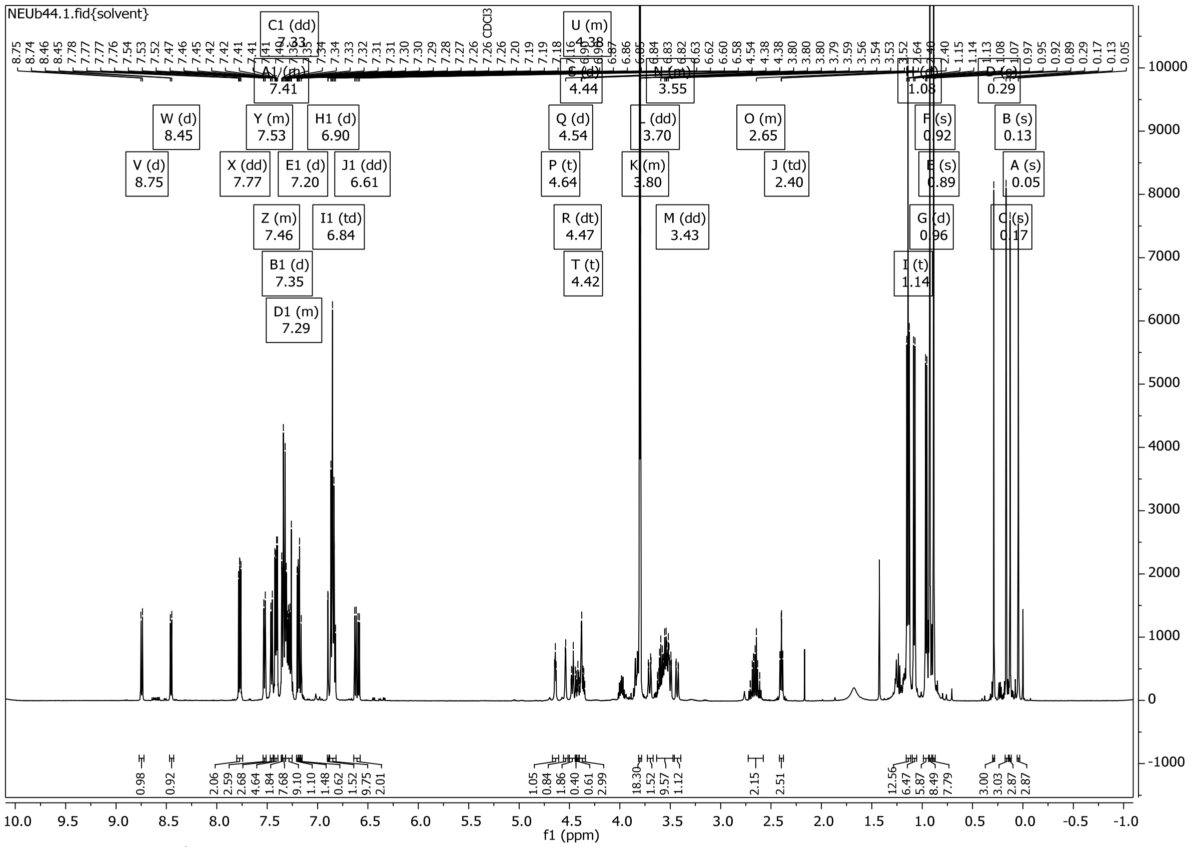

Spectrum 15. ^{1}H NMR of compound **10** (500 MHz, $CDCl_3$ filtered over basic Al_2O_3, r.t.)

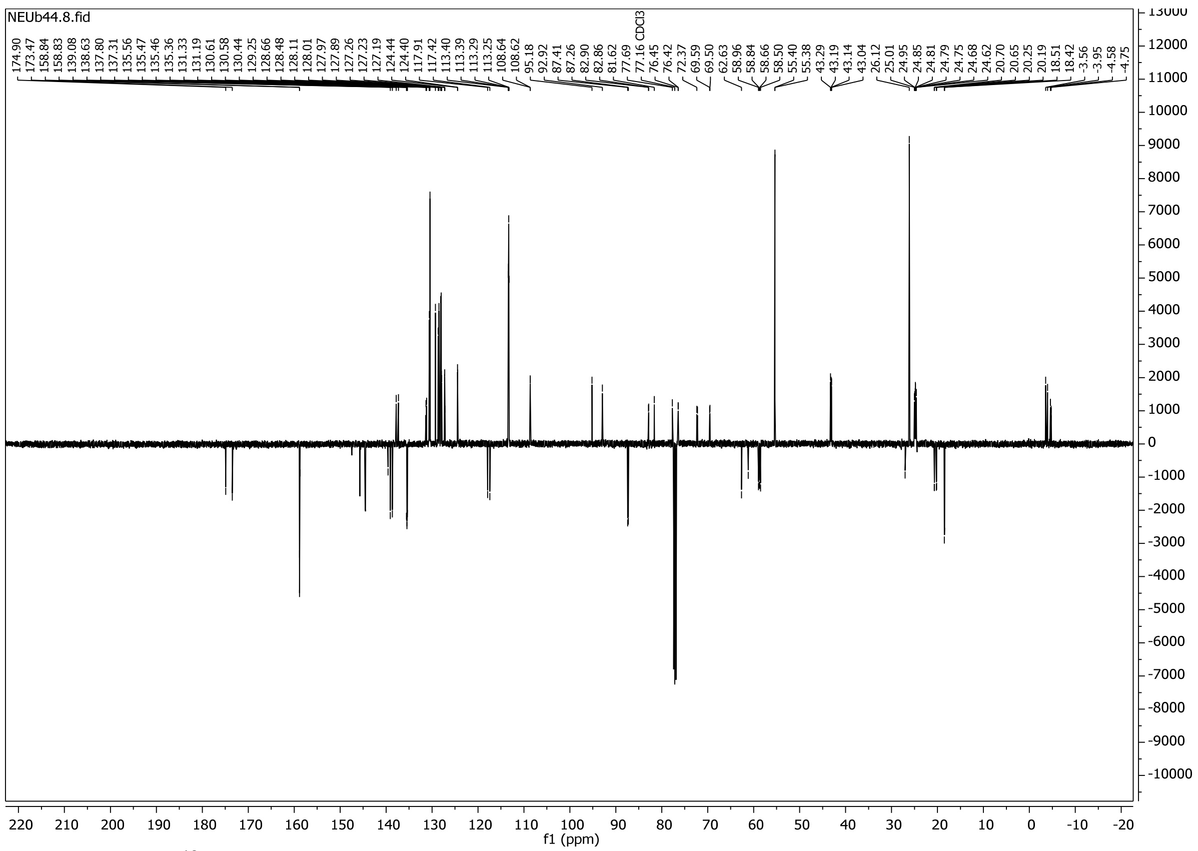

Spectrum 16. ^{13}C-NMR of compound **10** (126 MHz, $CDCl_3$ filtered over basic Al_2O_3, r.t.)

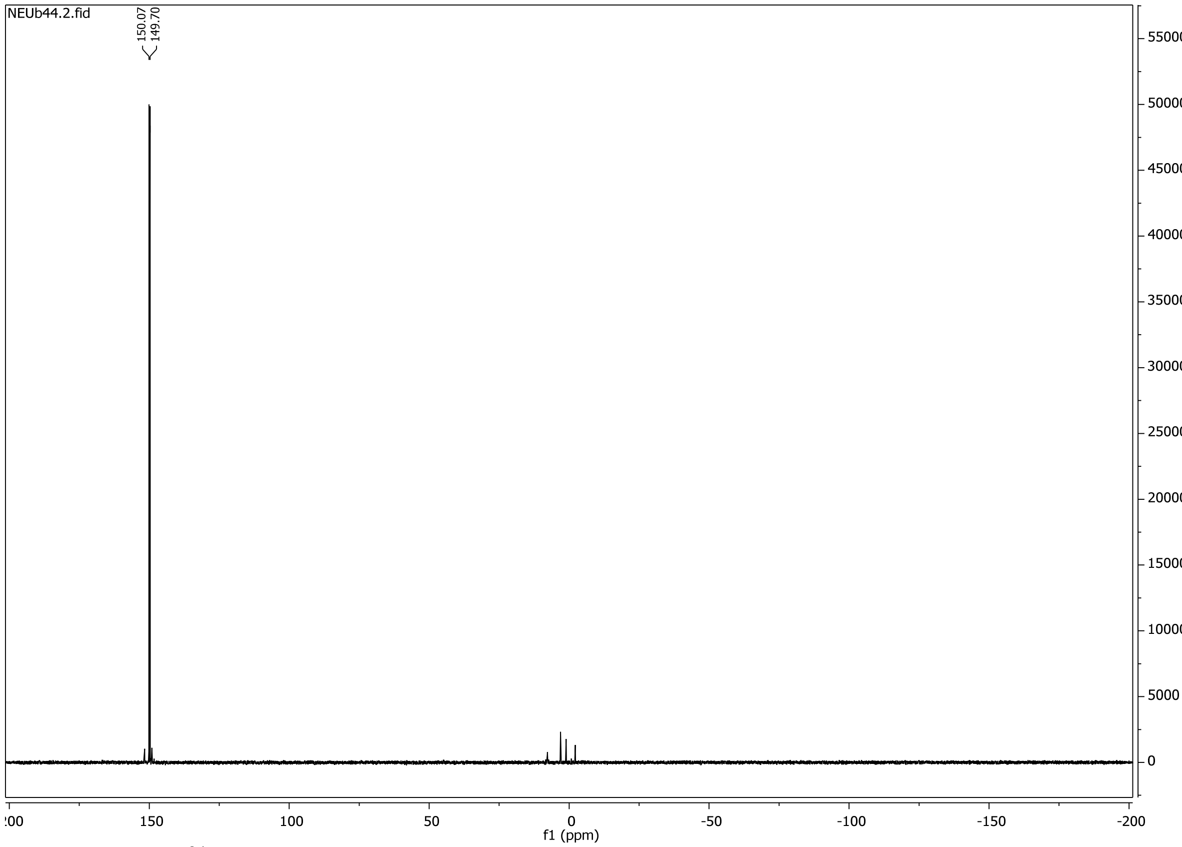

Spectrum 17. ^{31}P-NMR of compound **10** (202 MHz, $CDCl_3$ filtered over basic Al_2O_3, r.t.)

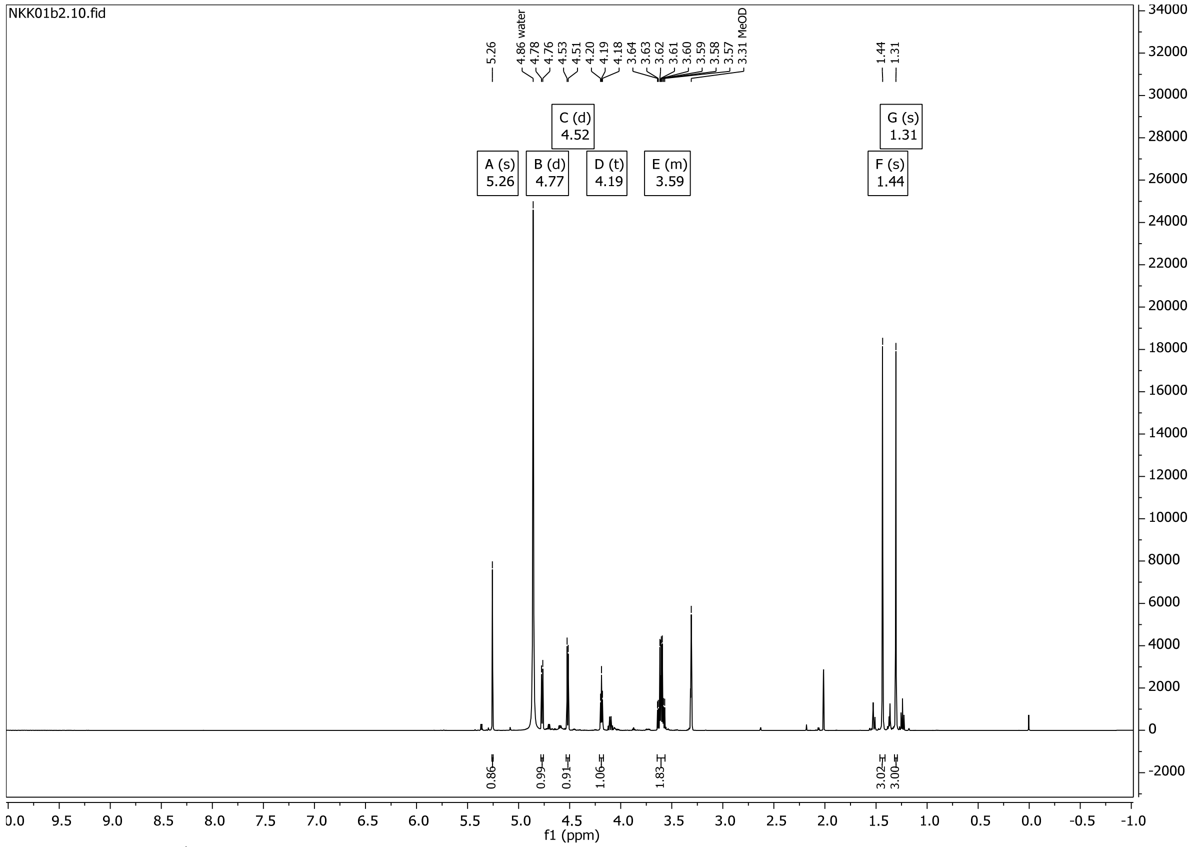

Spectrum 18. ^{1}H NMR of compound **11** (500 MHz, MeOD, r.t.)

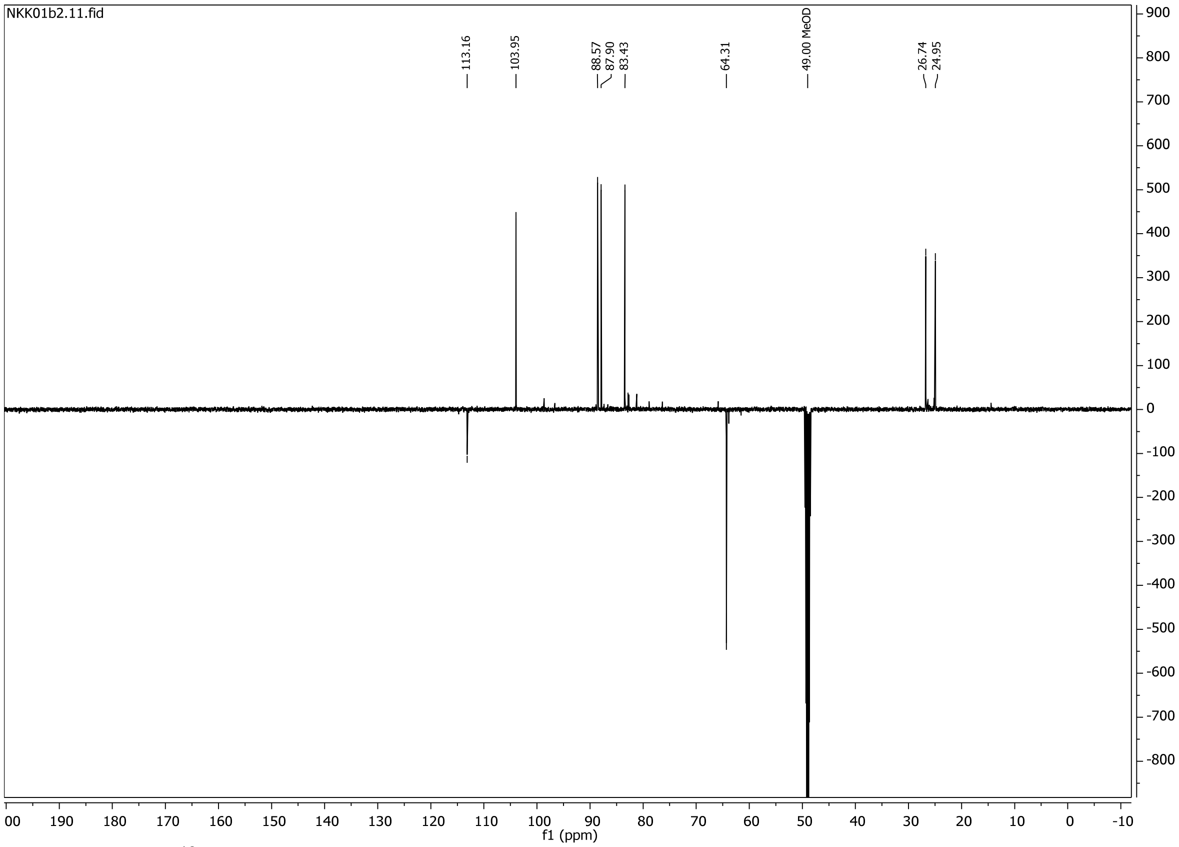

Spectrum 19. ^{13}C- NMR of compound **11** (75 MHz, MeOD, r.t.)

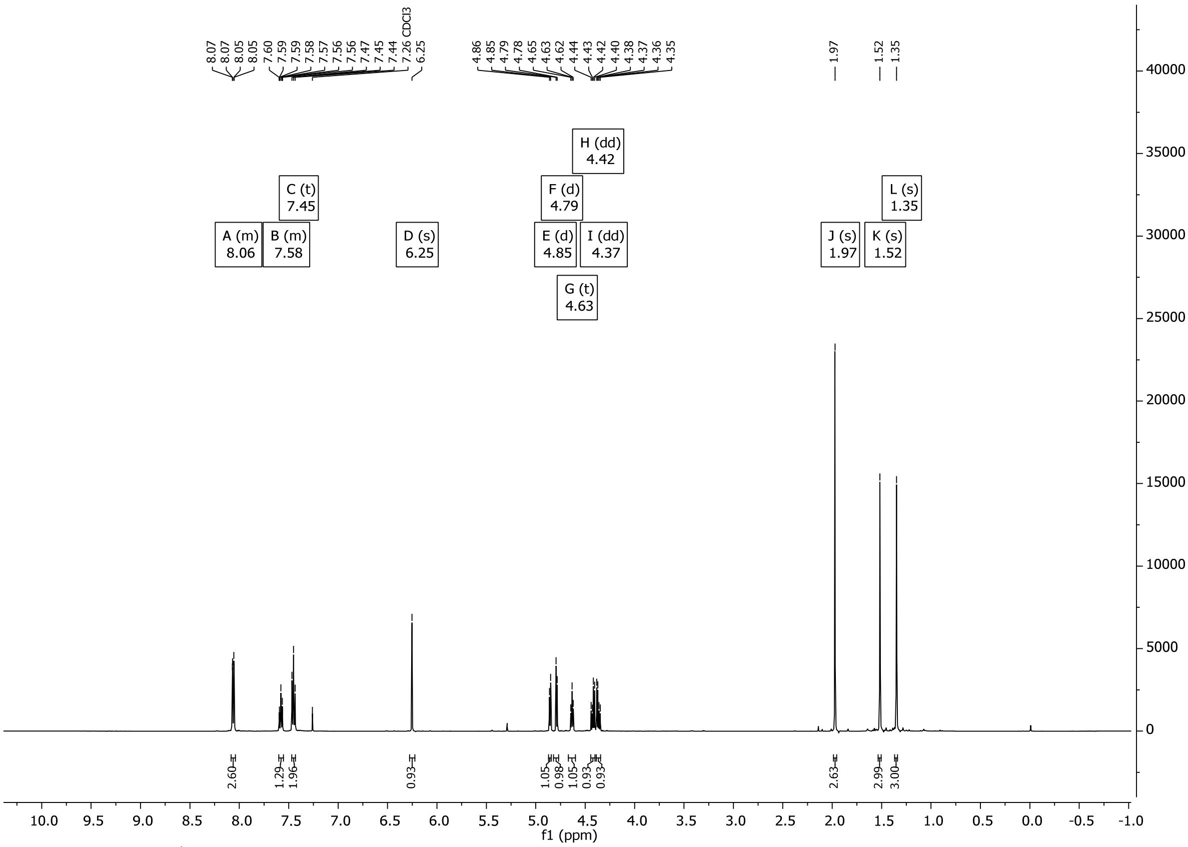

Spectrum 20. ^{1}H NMR of compound **12** (500 MHz, $CDCl_3$, r.t.)

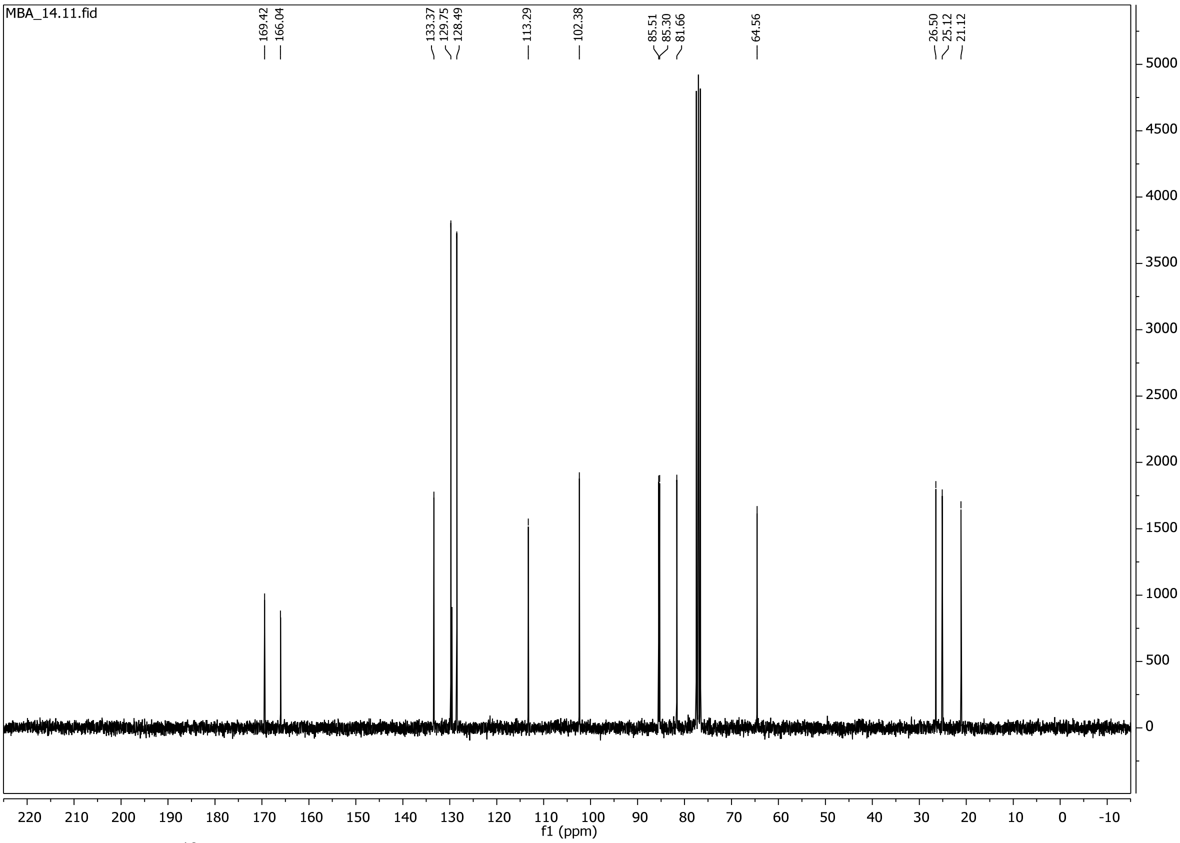

Spectrum 21: ^{13}C-NMR of compound **12** (75 MHz, $CDCl_3$, r.t.)

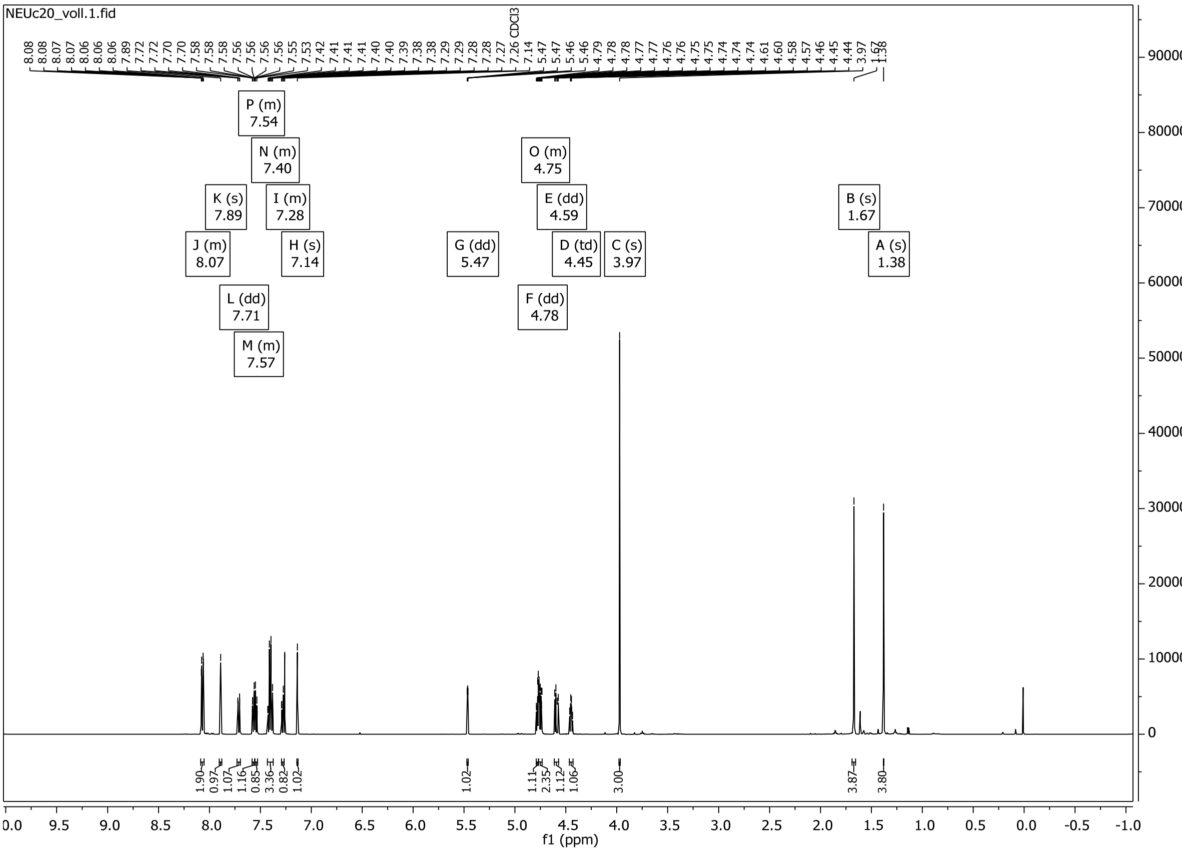

Spectrum 22. ^{1}H NMR of compound **13** (500 MHz, $CDCl_3$, r.t.)

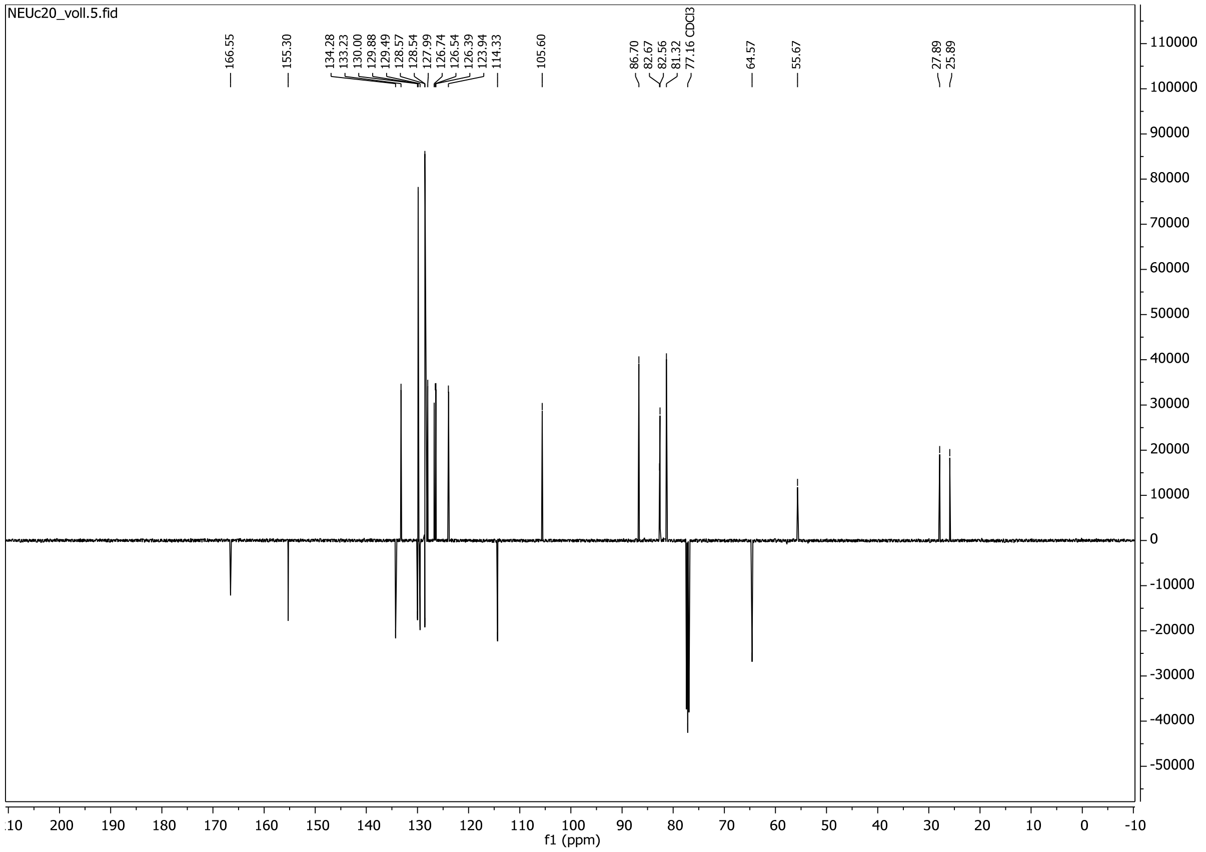

Spectrum 23. ^{13}C- NMR of compound **13** (75 MHz, $CDCl_3$, r.t.)

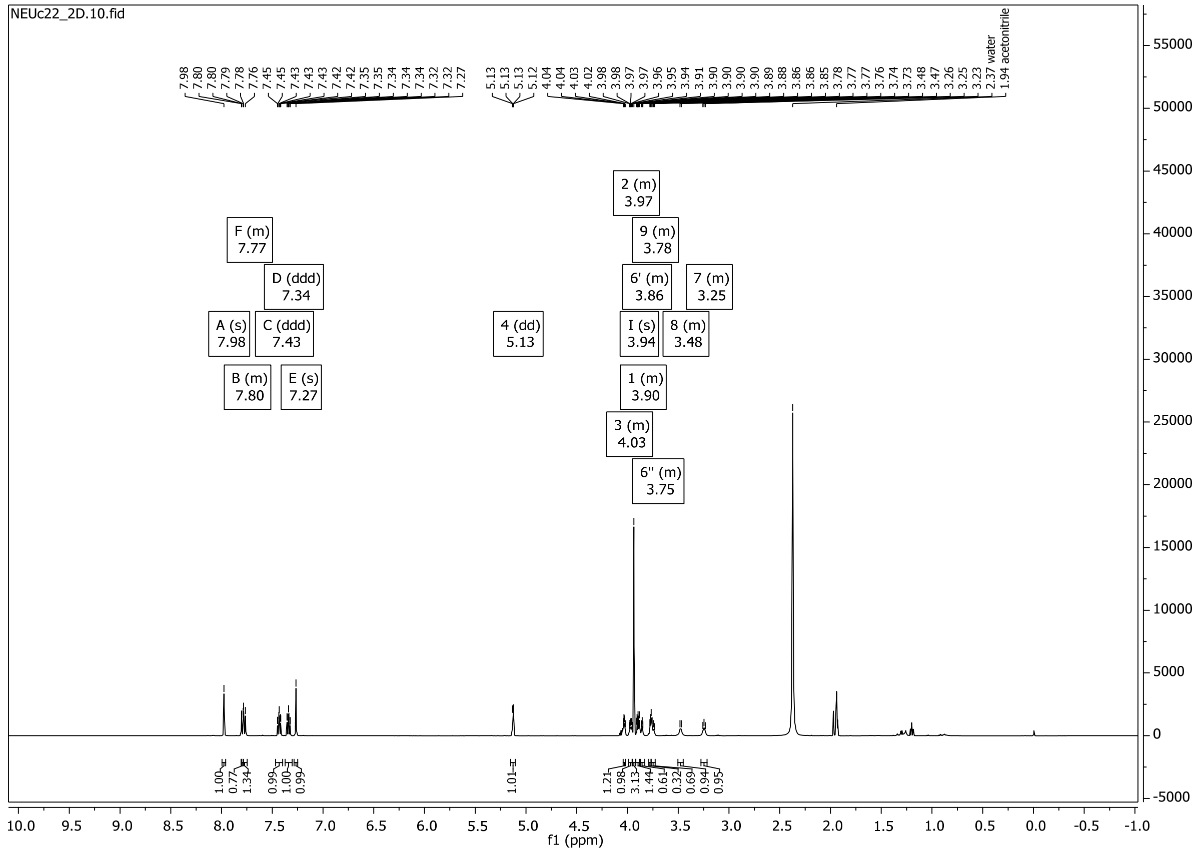

Spectrum 24. ^{1}H NMR of compound **14** (500 MHz, acetonitrile, r.t.).

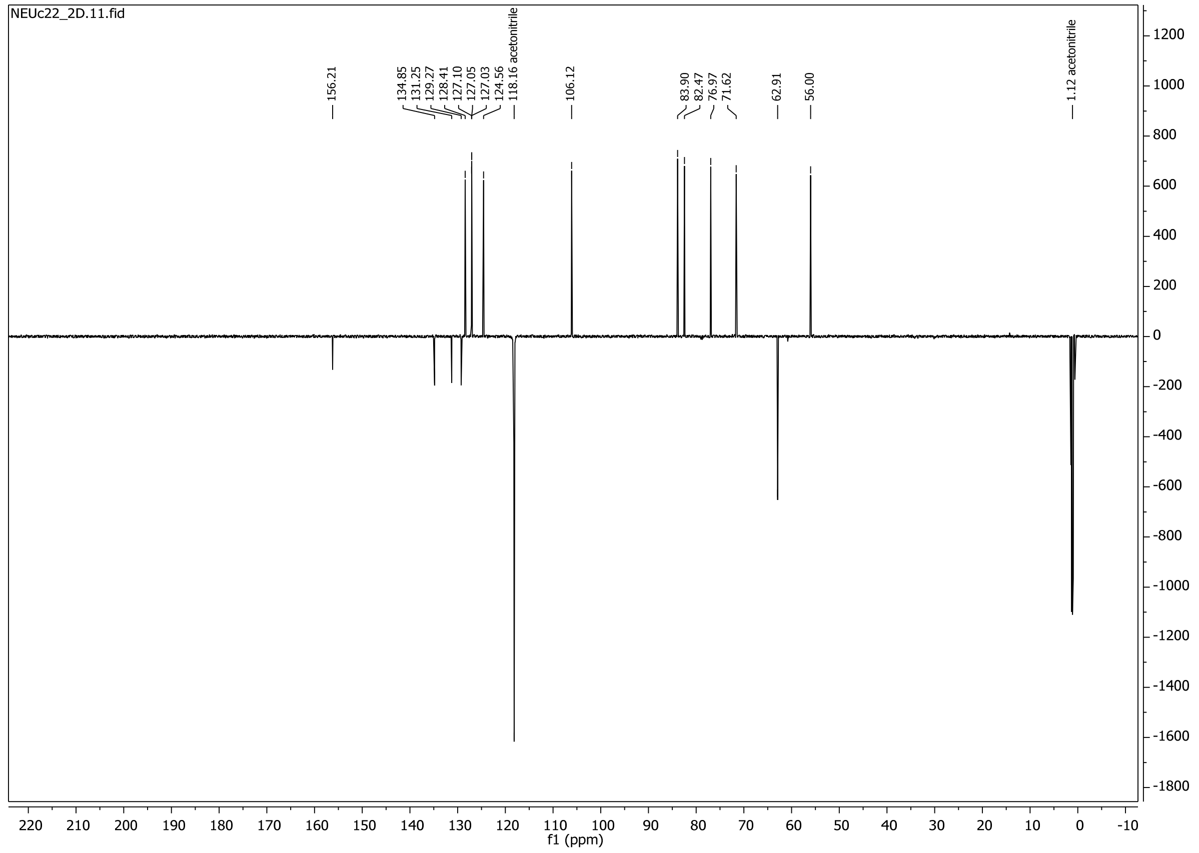

Spectrum 25. ^{13}C NMR of compound **14** (126 MHz, acetonitrile, r.t.)

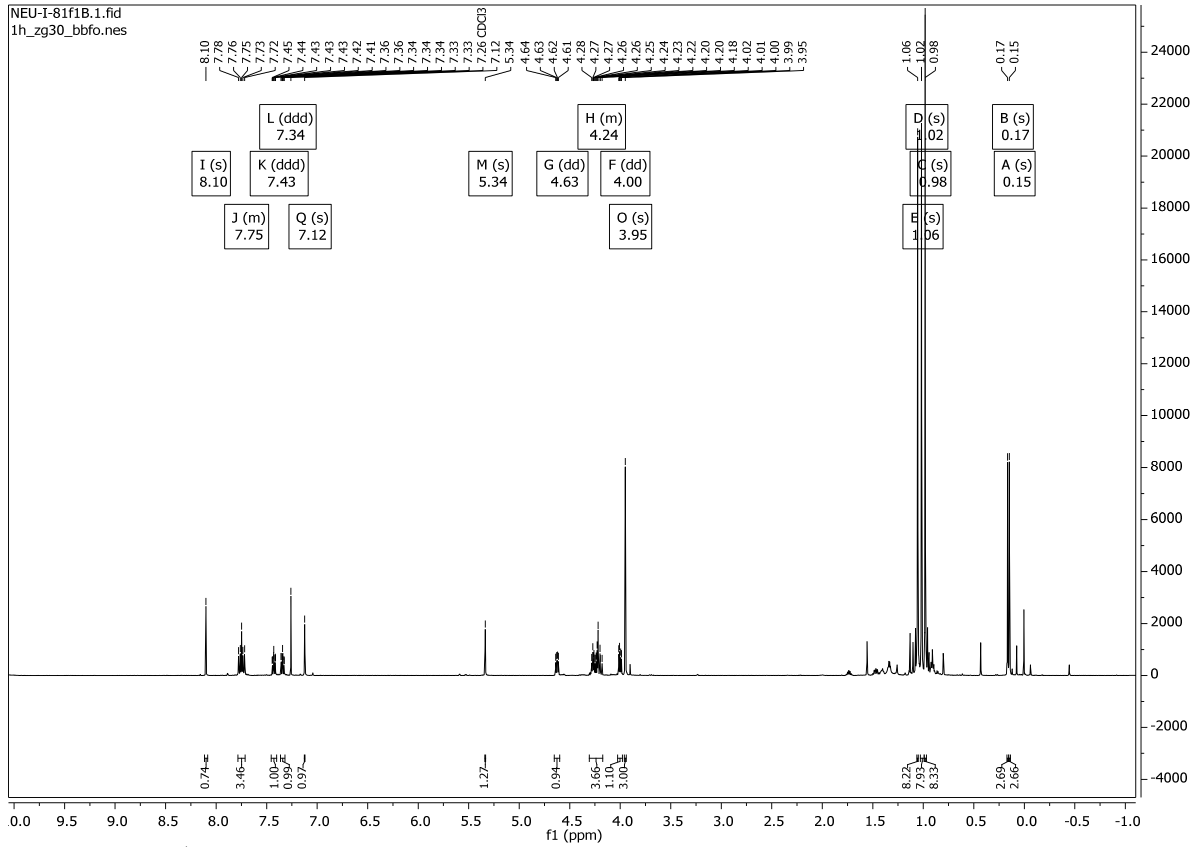

Spectrum 26. ^{1}H NMR of compound **15** (500 MHz, $CDCl_3$, r.t.).

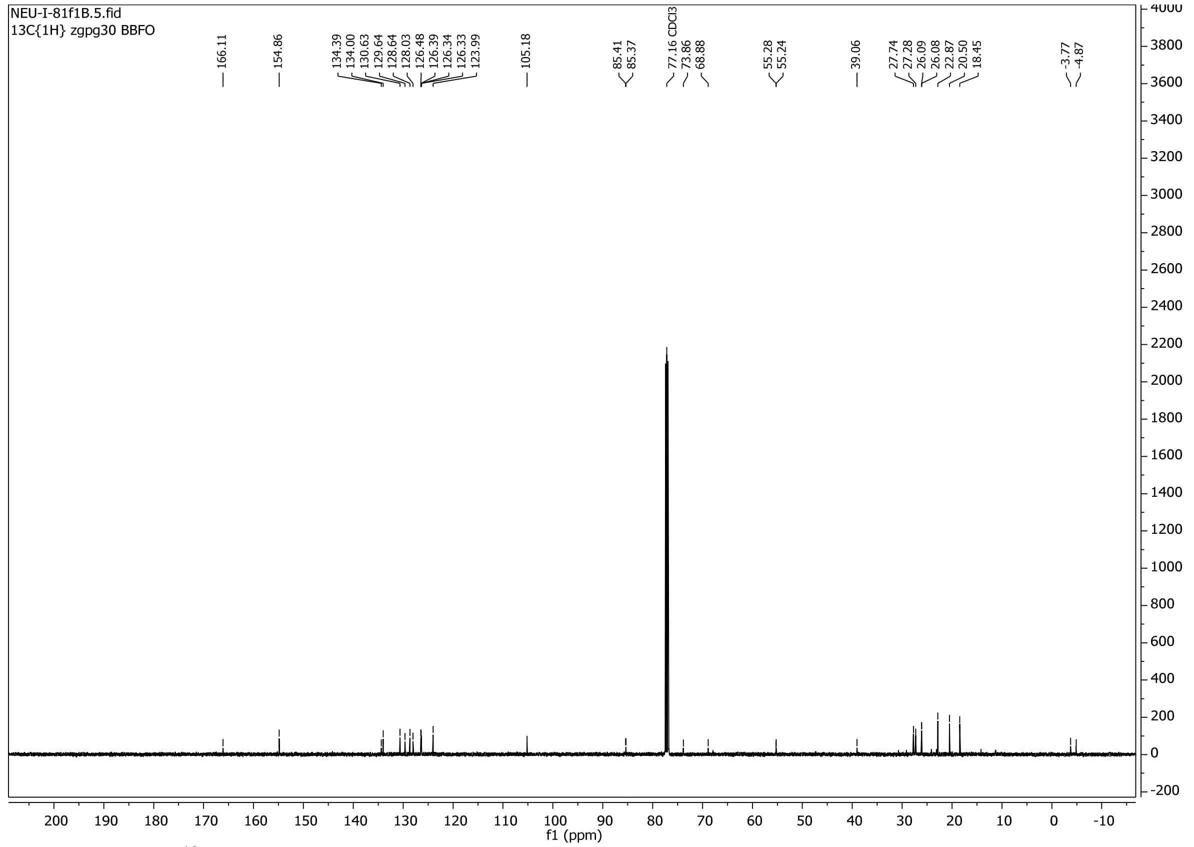

Spectrum 27. ^{13}C-NMR of compound **15** (126 MHz, $CDCl_3$, r.t.).

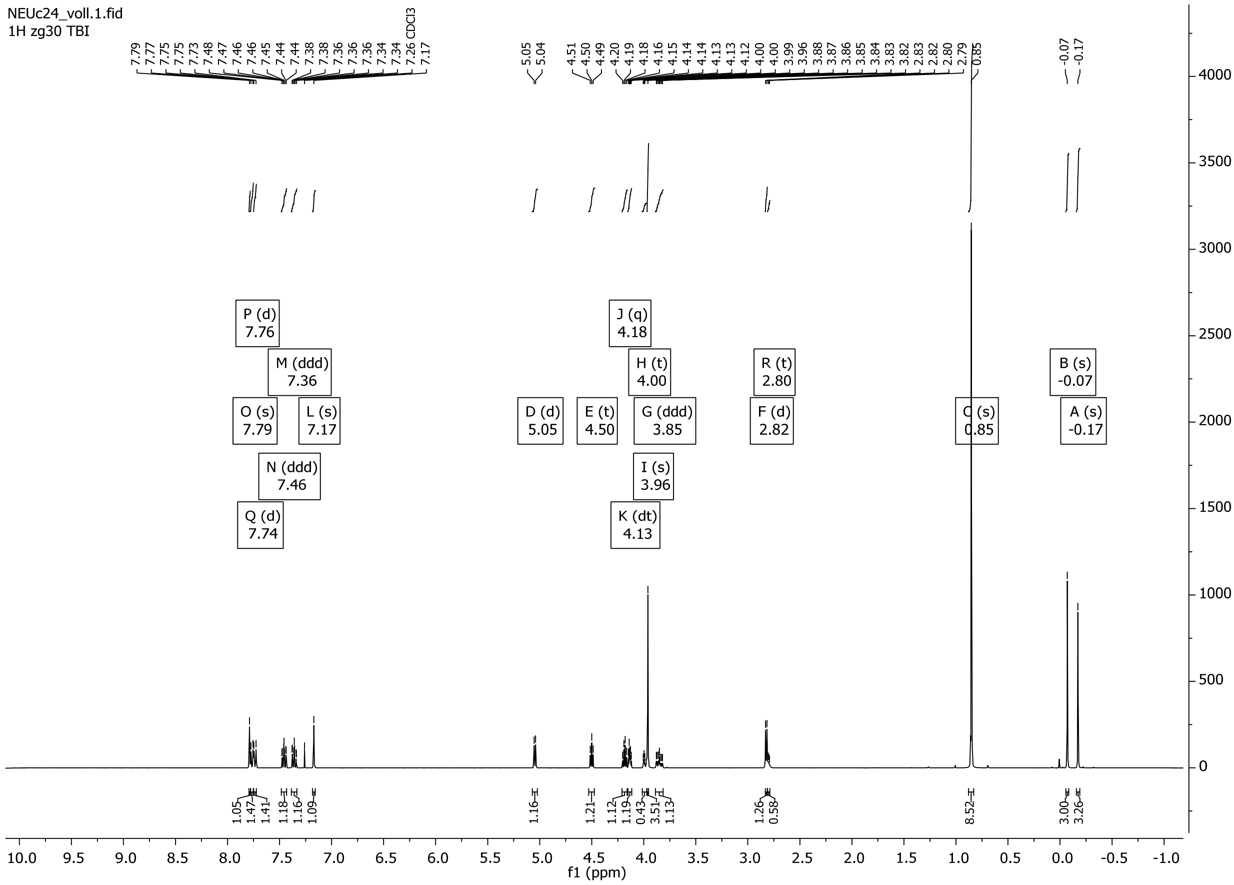

Spectrum 28. ^{1}H NMR of compound **16** (400 MHz, $CDCl_3$, r.t.).

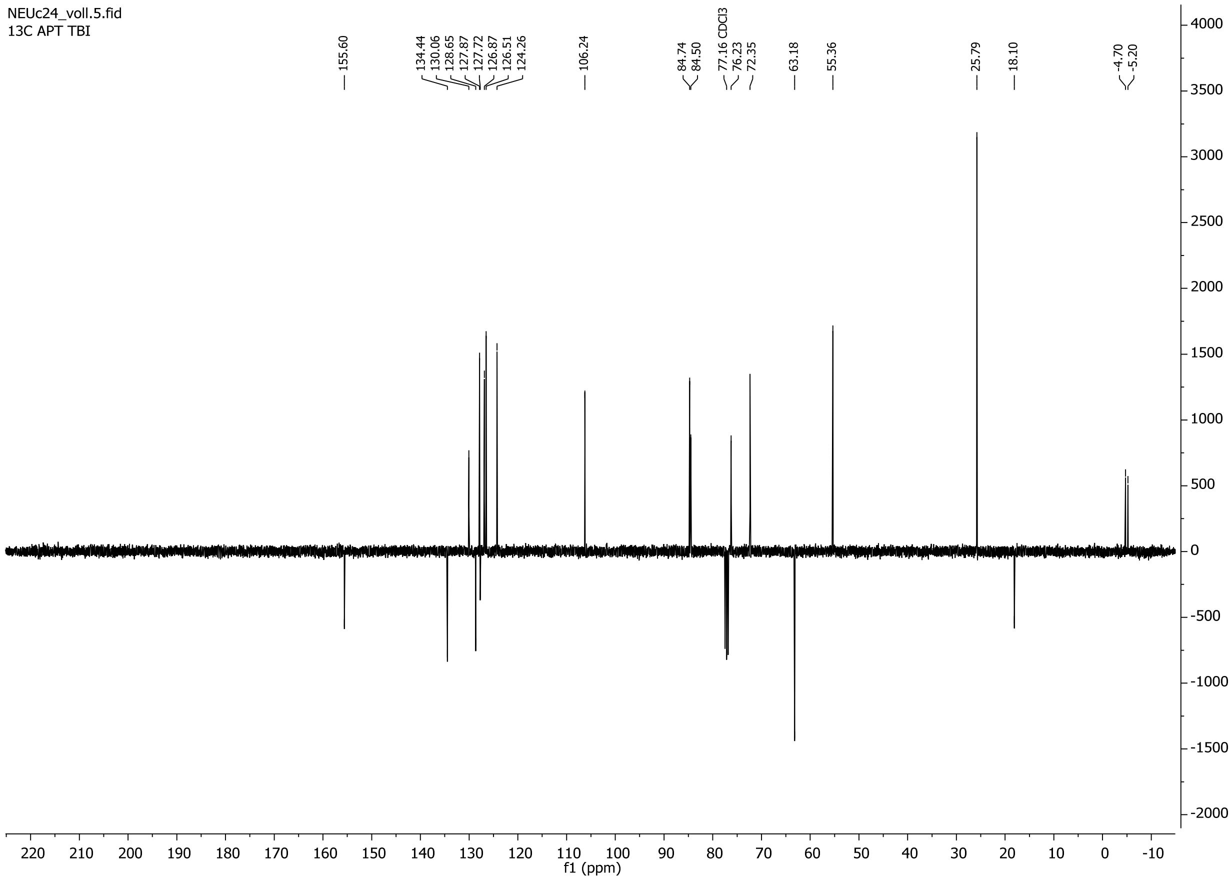

Spectrum 29. ^{13}C NMR of compound **16** (101 MHz, $CDCl_3$, r.t.).

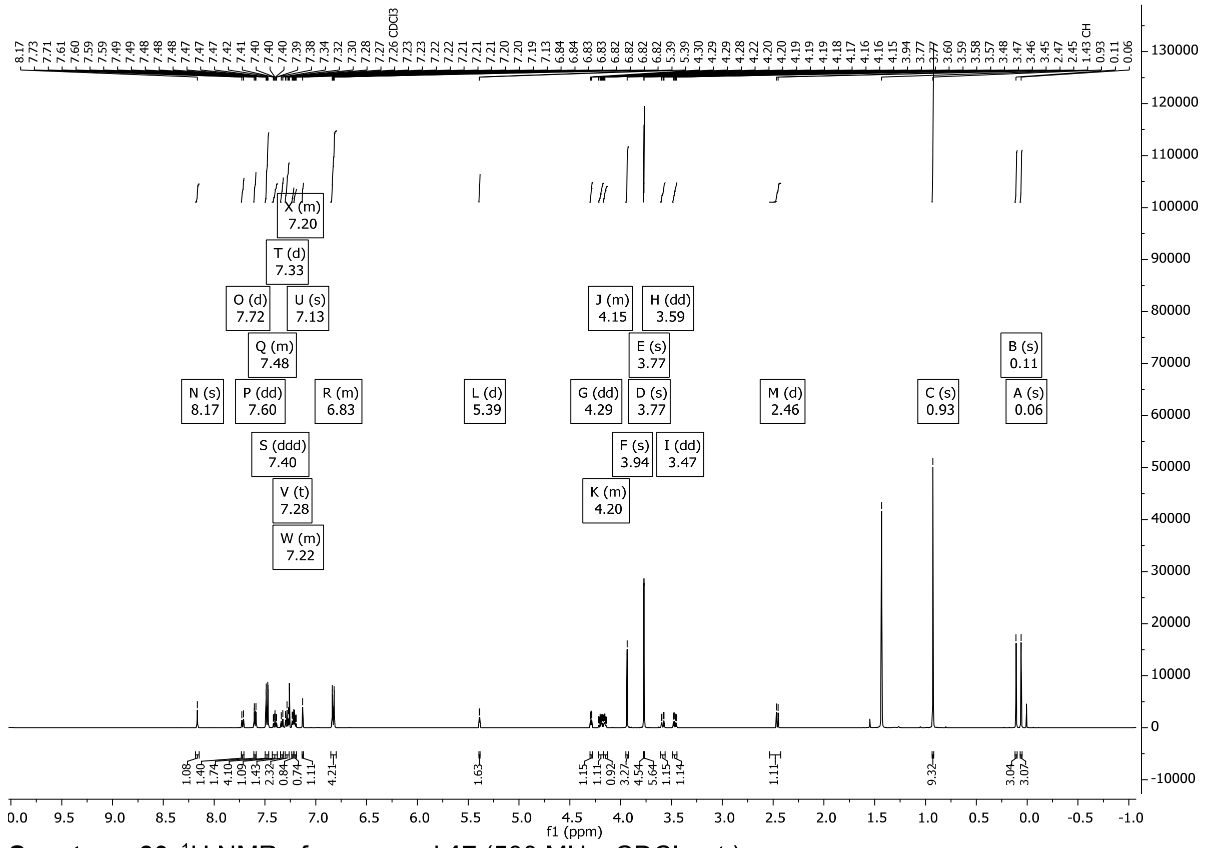

Spectrum 30. ^{1}H NMR of compound **17** (500 MHz, $CDCl_3$, r.t.).

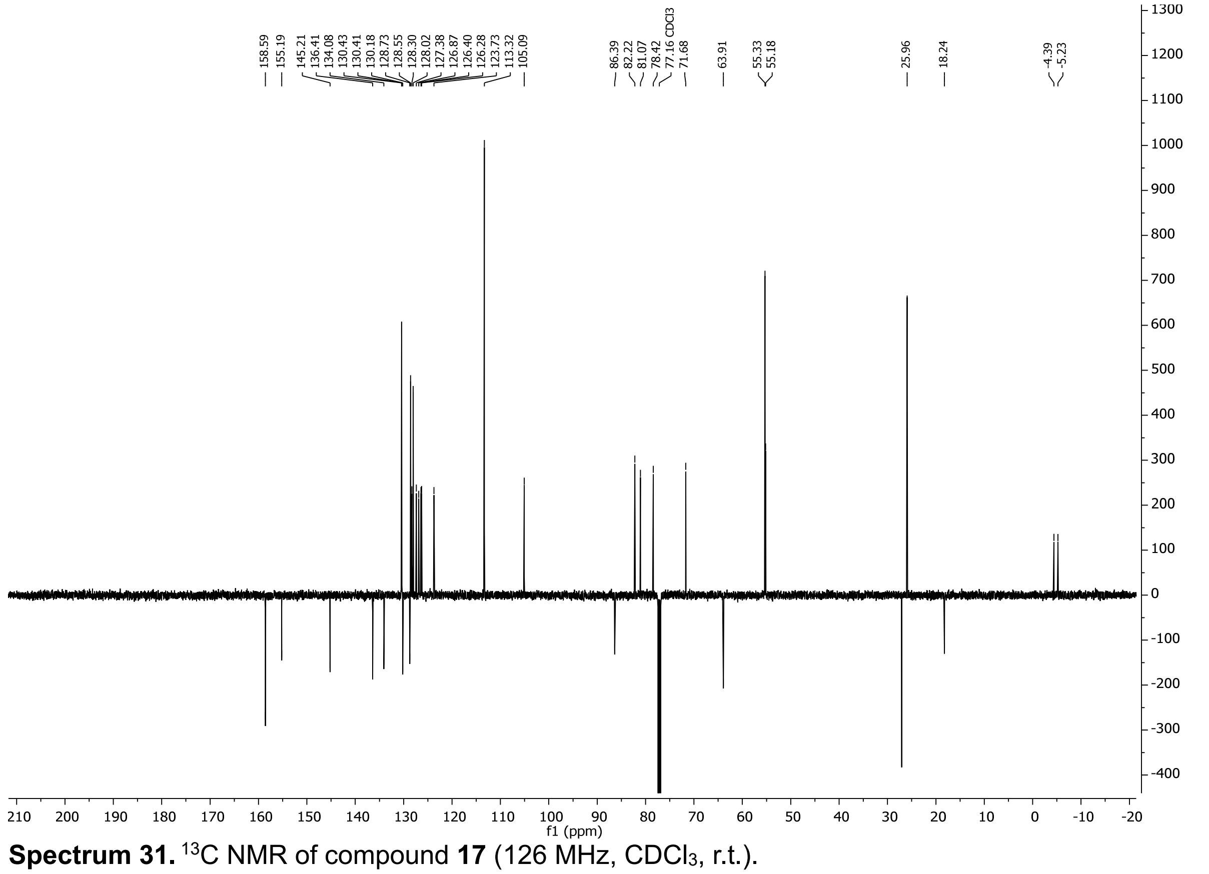

Spectrum 31. ^{13}C NMR of compound **17** (126 MHz, $CDCl_3$, r.t.).

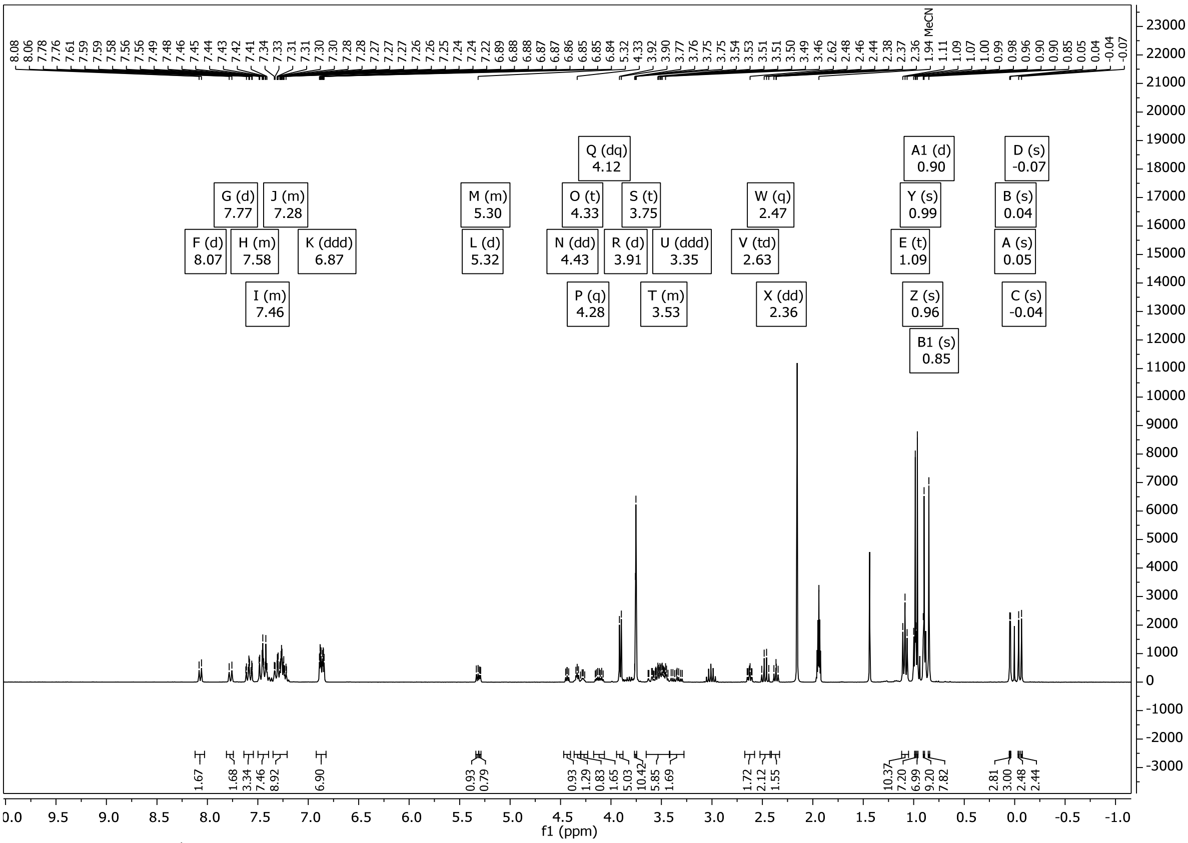

Spectrum 32. ^{1}H NMR of compound **18** (300 MHz, acetonitrile, r.t.)

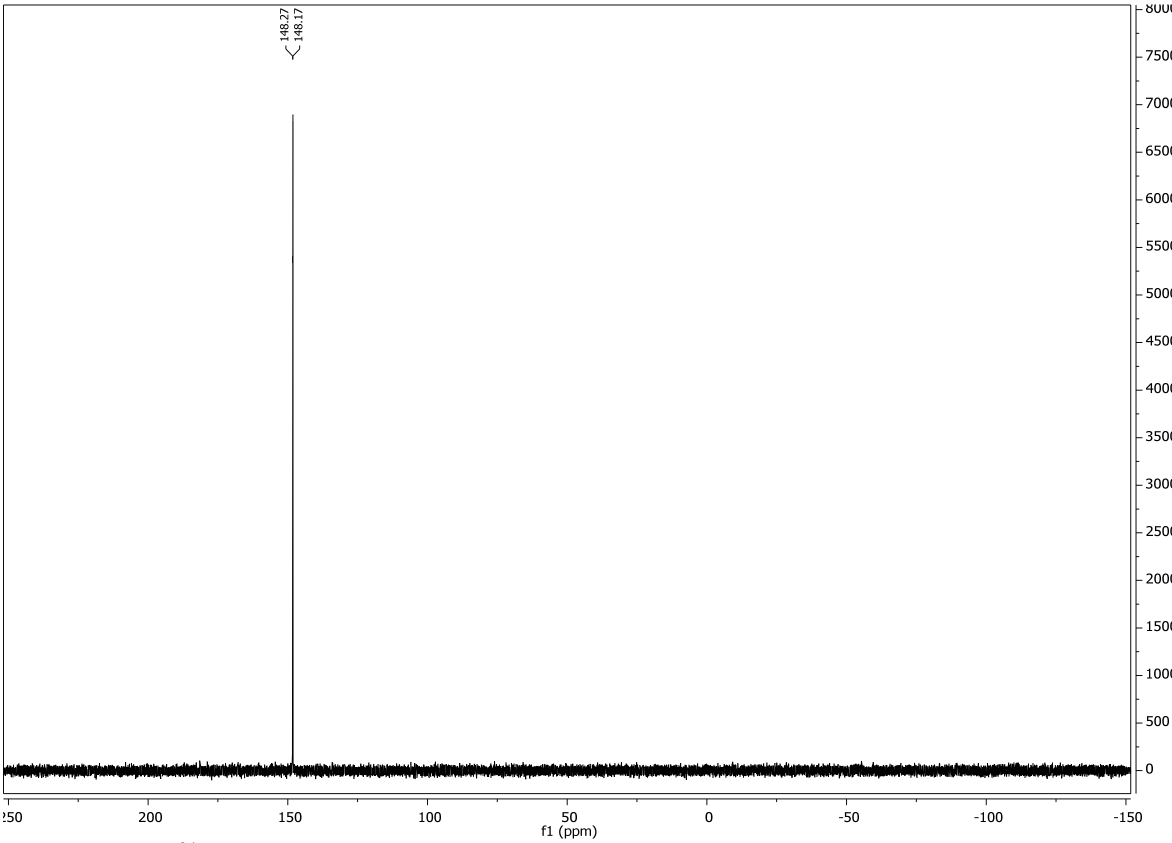

Spectrum 33. ^{31}P-NMR of compound **18** (122 MHz, acetonitrile, r.t.).

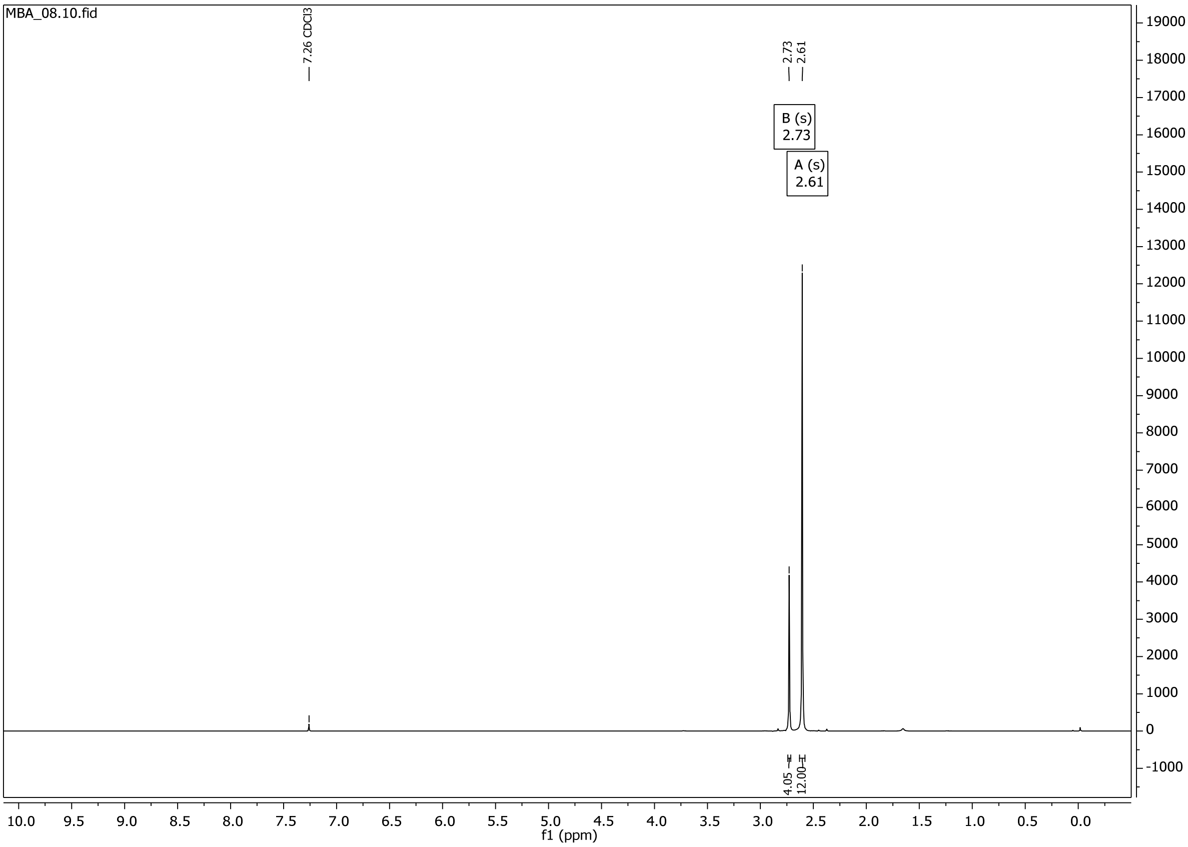

Spectrum 34. ^{1}H NMR of **$ZnCl_2$-TMEDA** (300 MHz, $CDCl_3$, r.t.).

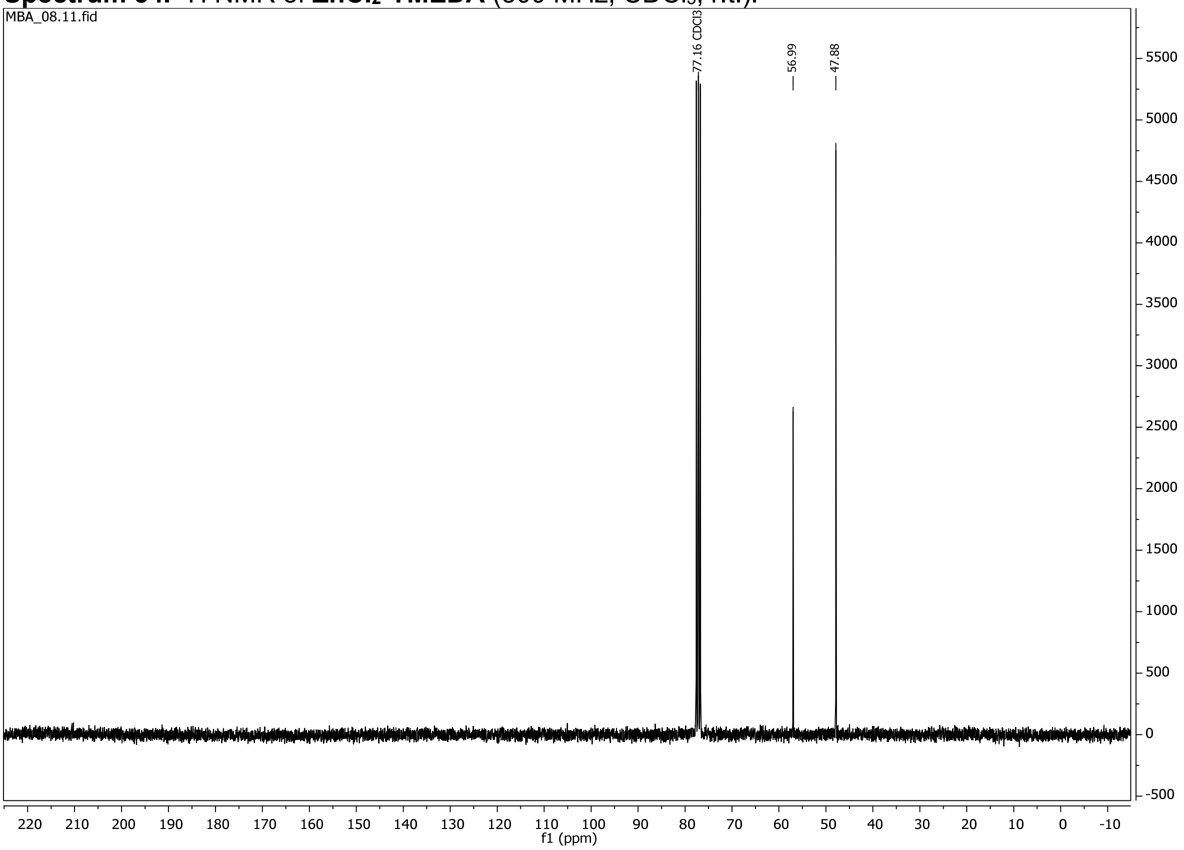

Spectrum 35. ^{13}C-NMR of **$ZnCl_2$-TMEDA** (75 MHz, $CDCl_3$, r.t.).

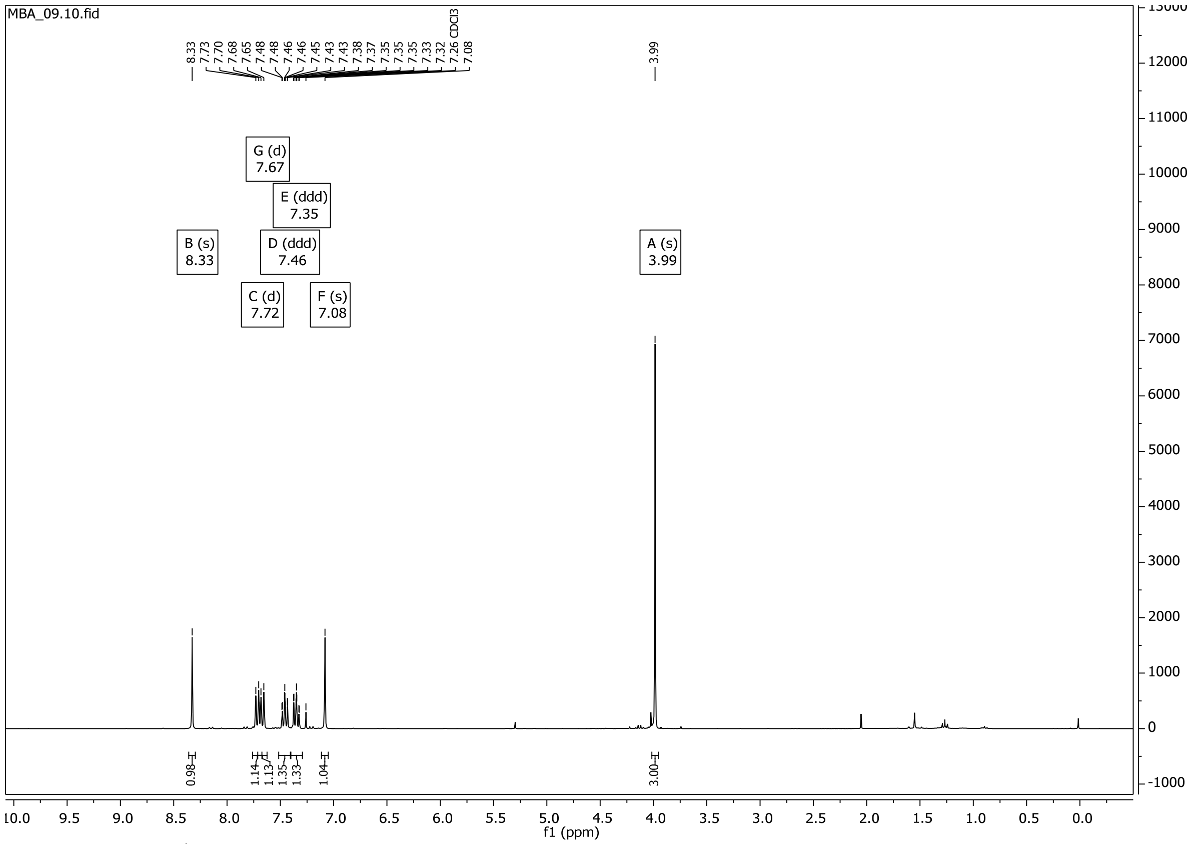

Spectrum 36. ^{1}H NMR of compound **19** (300 MHz, $CDCl_3$, r.t.).

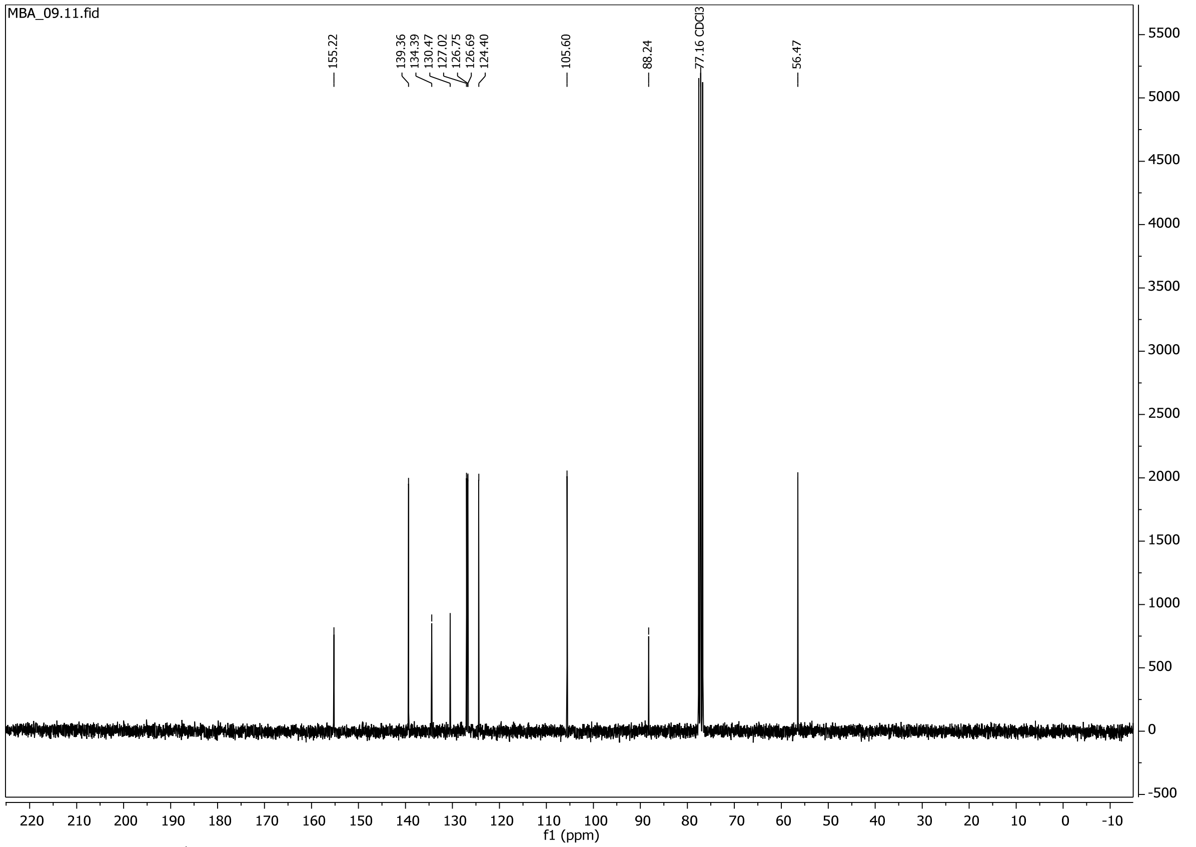

Spectrum 37. ^{1}H NMR of compound **19** (75 MHz, $CDCl_3$, r.t.).

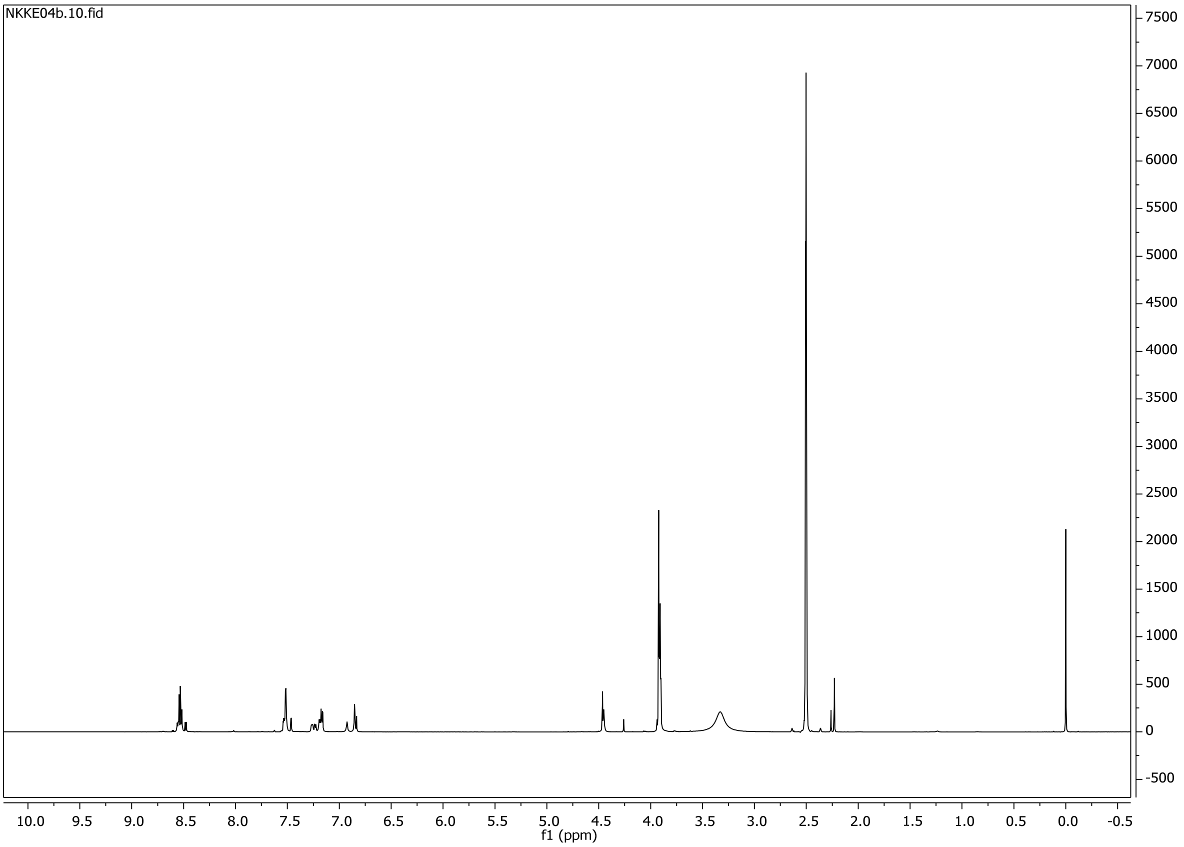

Spectrum 38. ^{1}H-NMR of compound **21** (500 MHz, DMSO, r.t.).

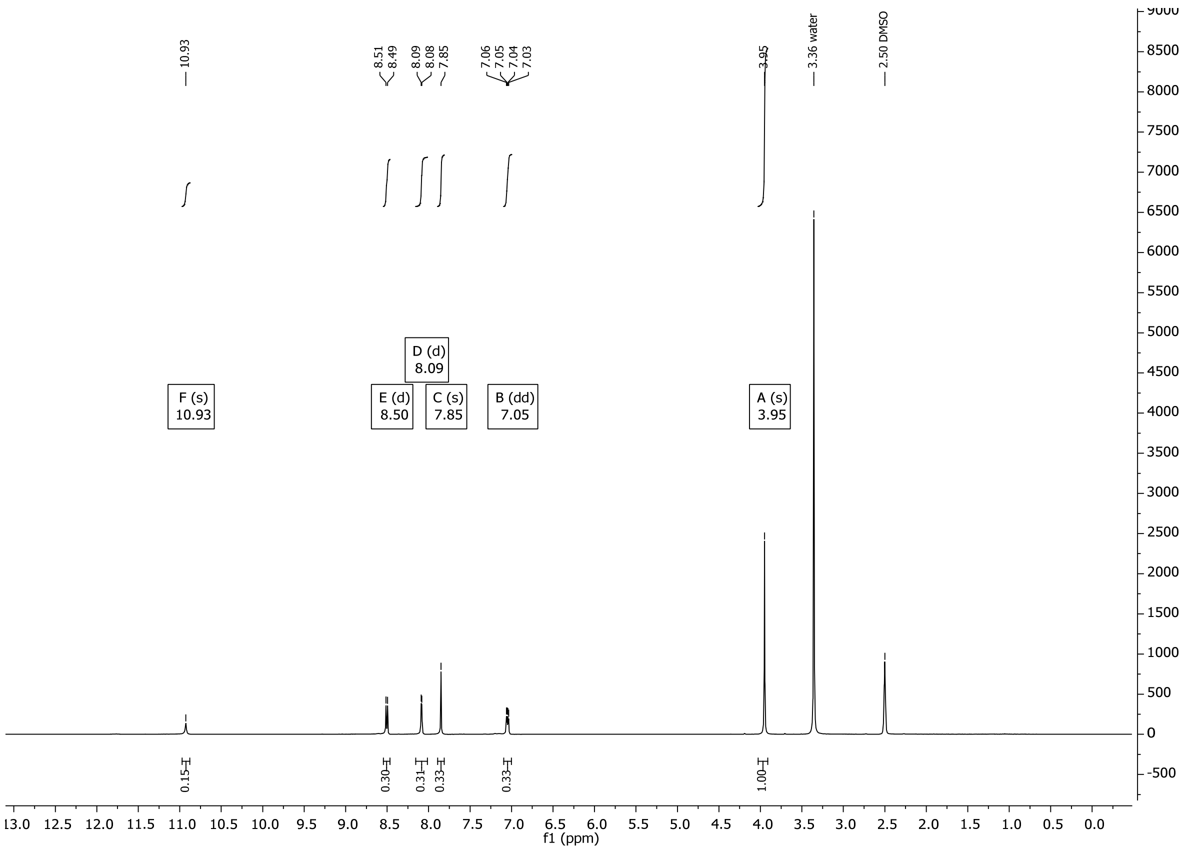

Spectrum 39. ^{1}H NMR of compound **22** (300 MHz, DMSO, r.t.).

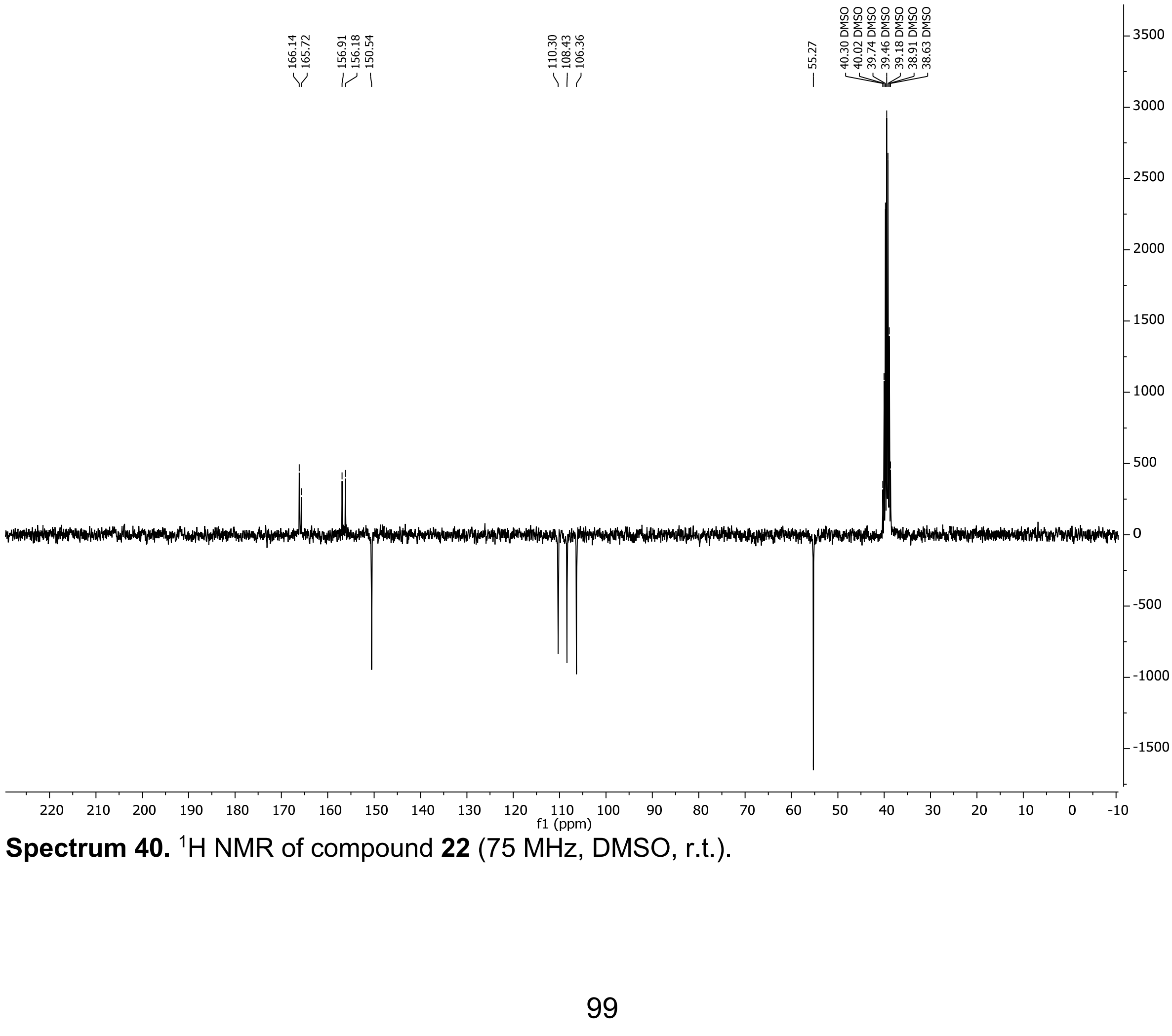

Spectrum 40. ^{1}H NMR of compound **22** (75 MHz, DMSO, r.t.).

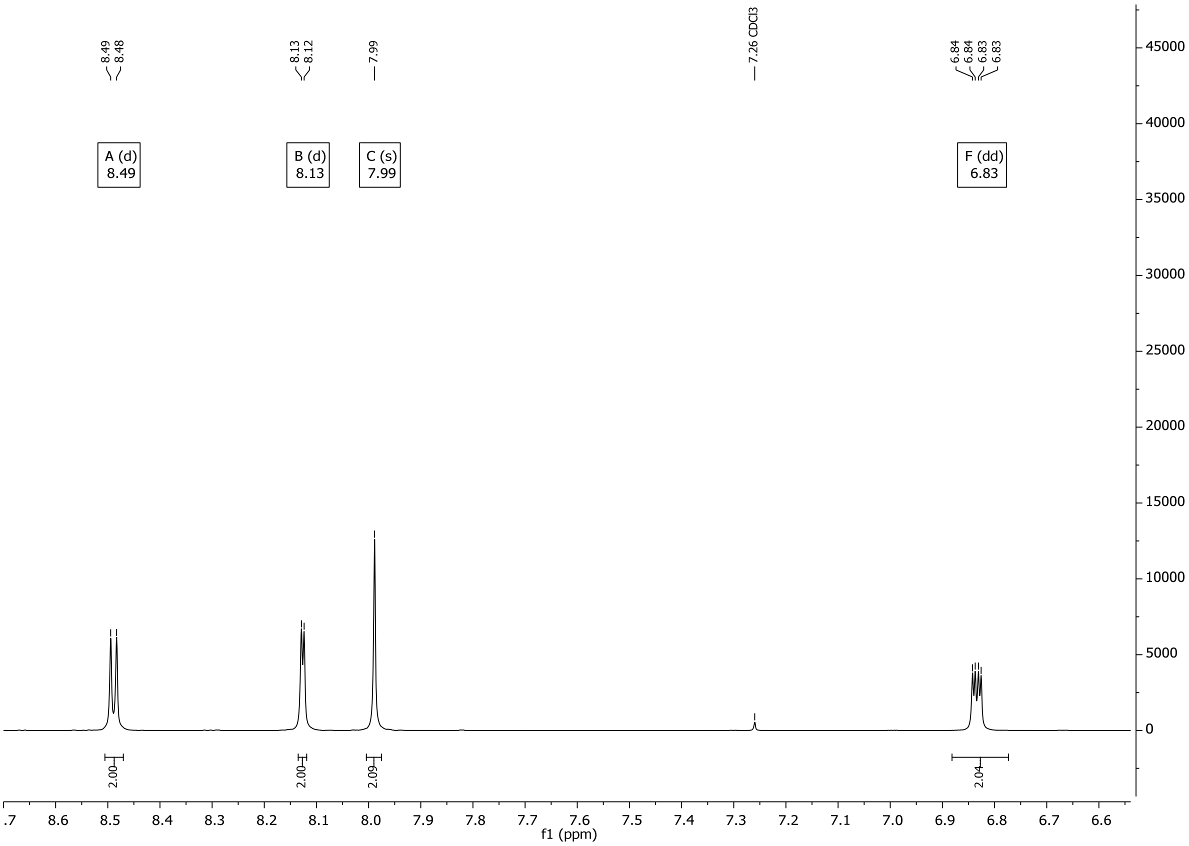

Spectrum 41. ^{1}H NMR of compound **23** (500 MHz, $CDCl_3$, r.t.).

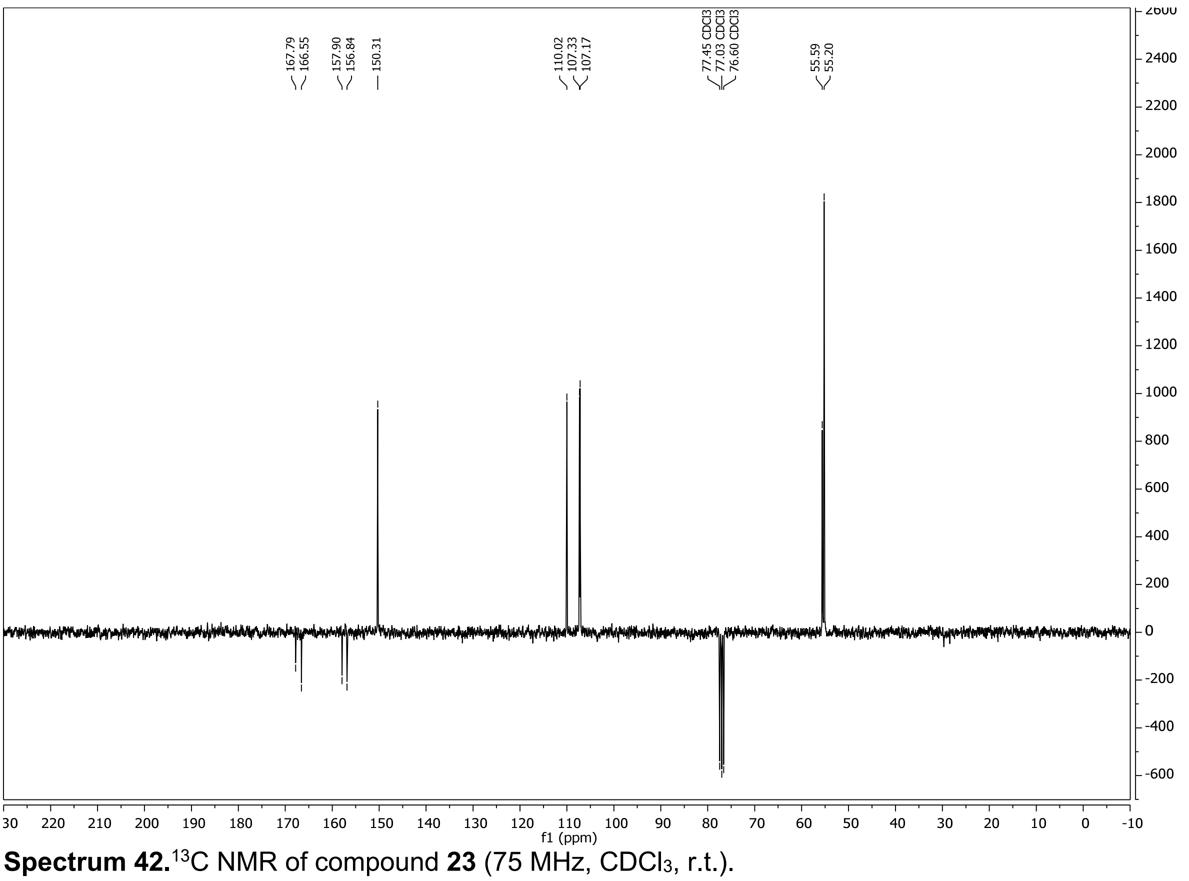

Spectrum 42. ^{13}C NMR of compound **23** (75 MHz, $CDCl_3$, r.t.).

IR-spectra

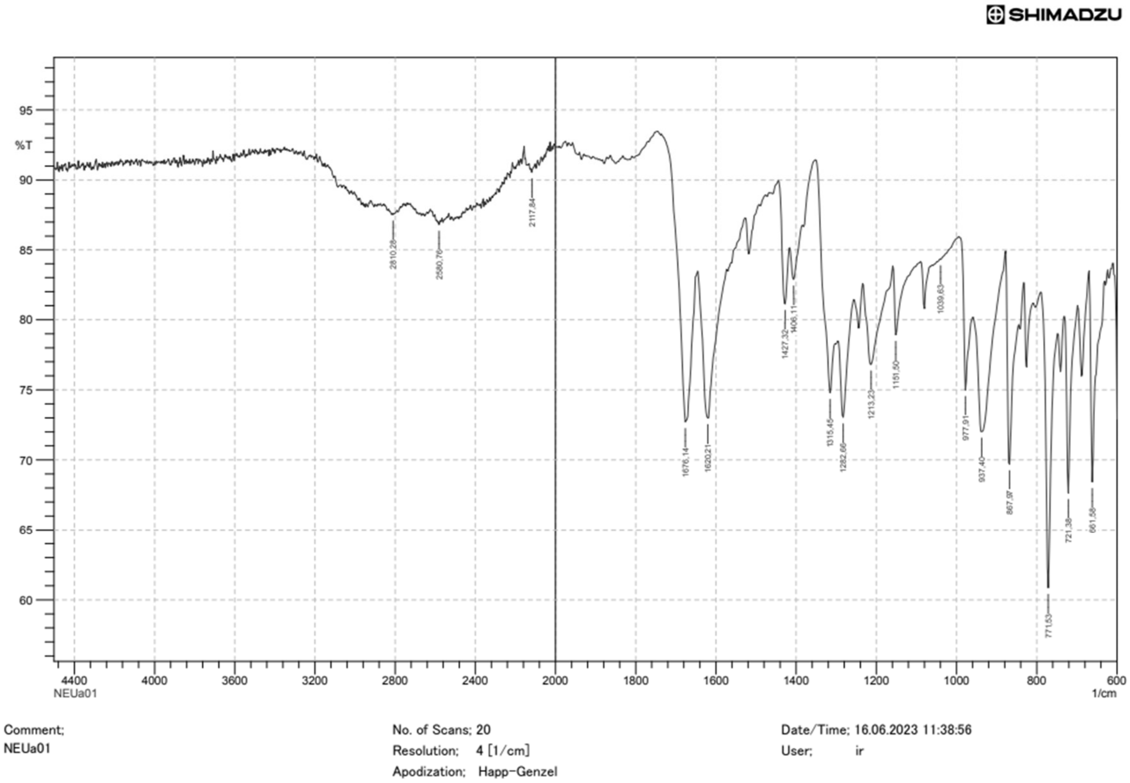

Spectrum 43. IR of compound **02.**

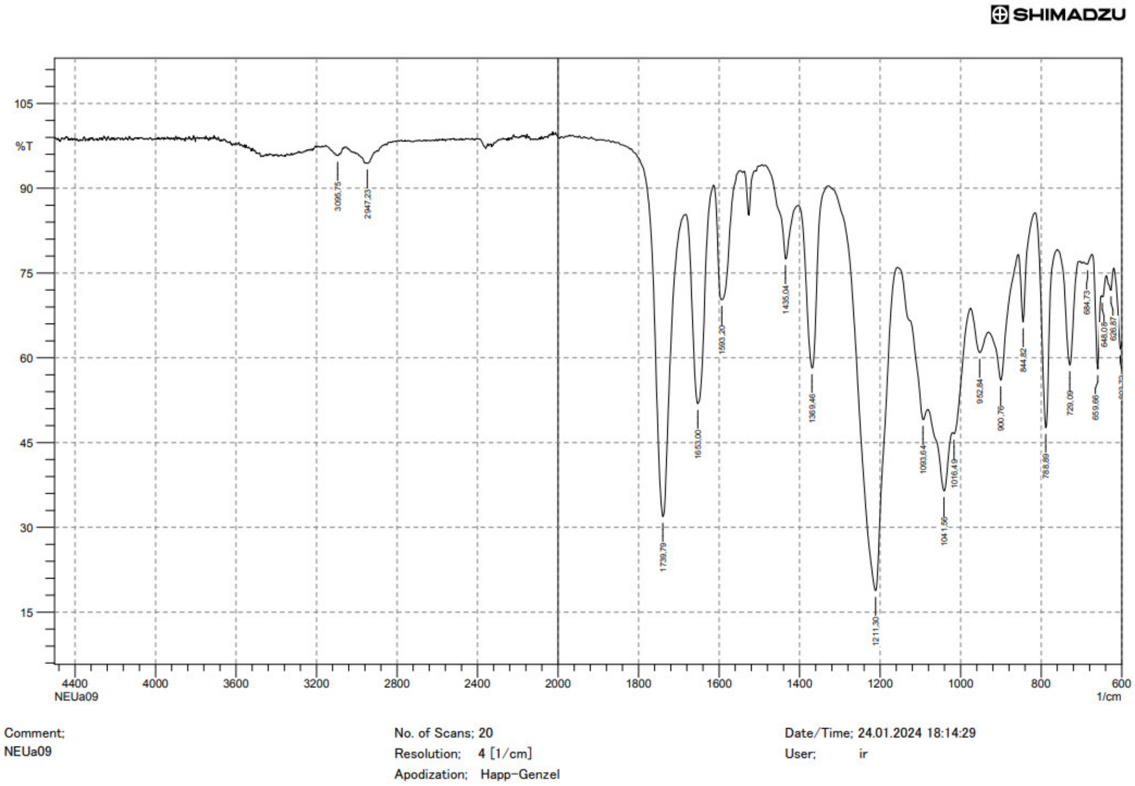

Spectrum 44. IR of compound **04**.

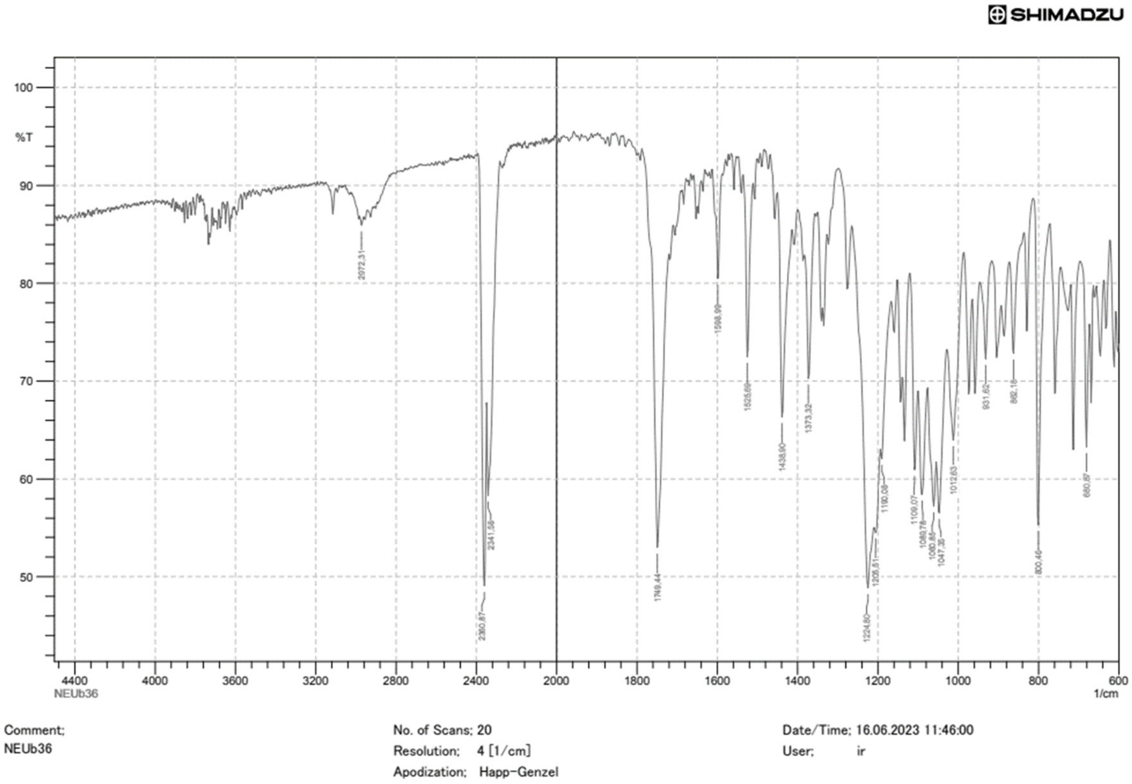

Spectrum 45. IR of compound **05**.

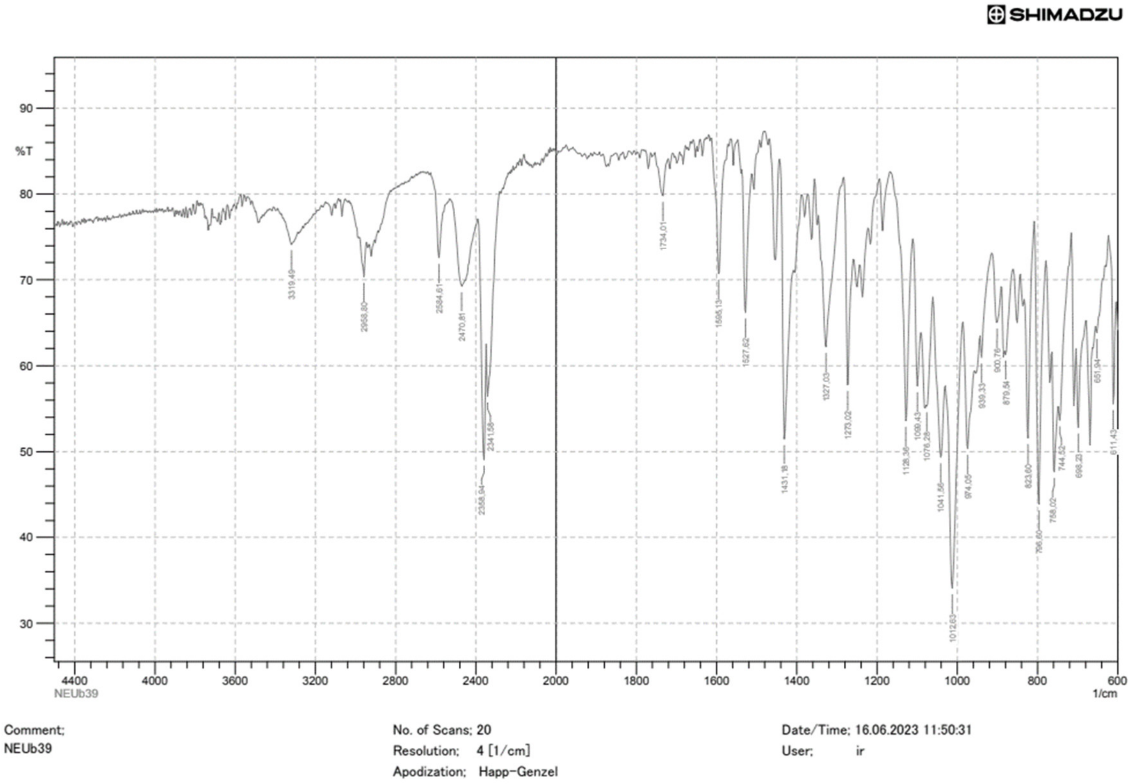

S**pectrum 46.** IR of compound **06**.

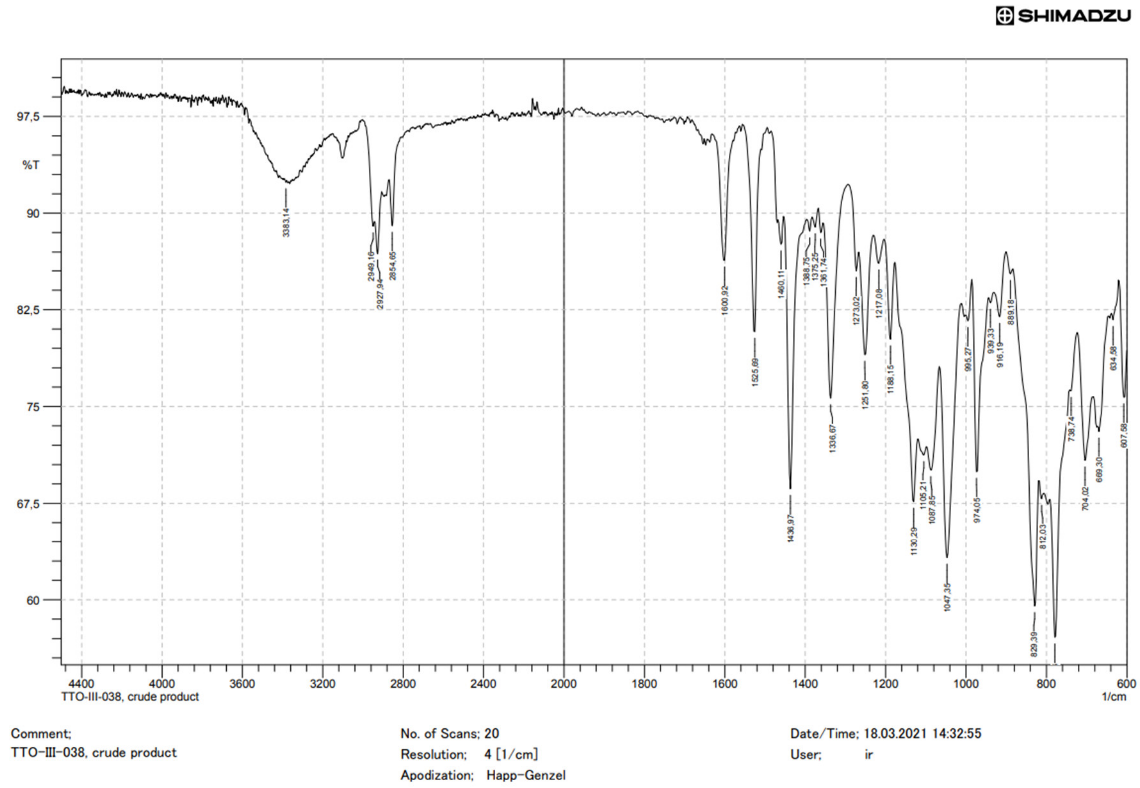

Spectrum 47. IR of compound **08**.

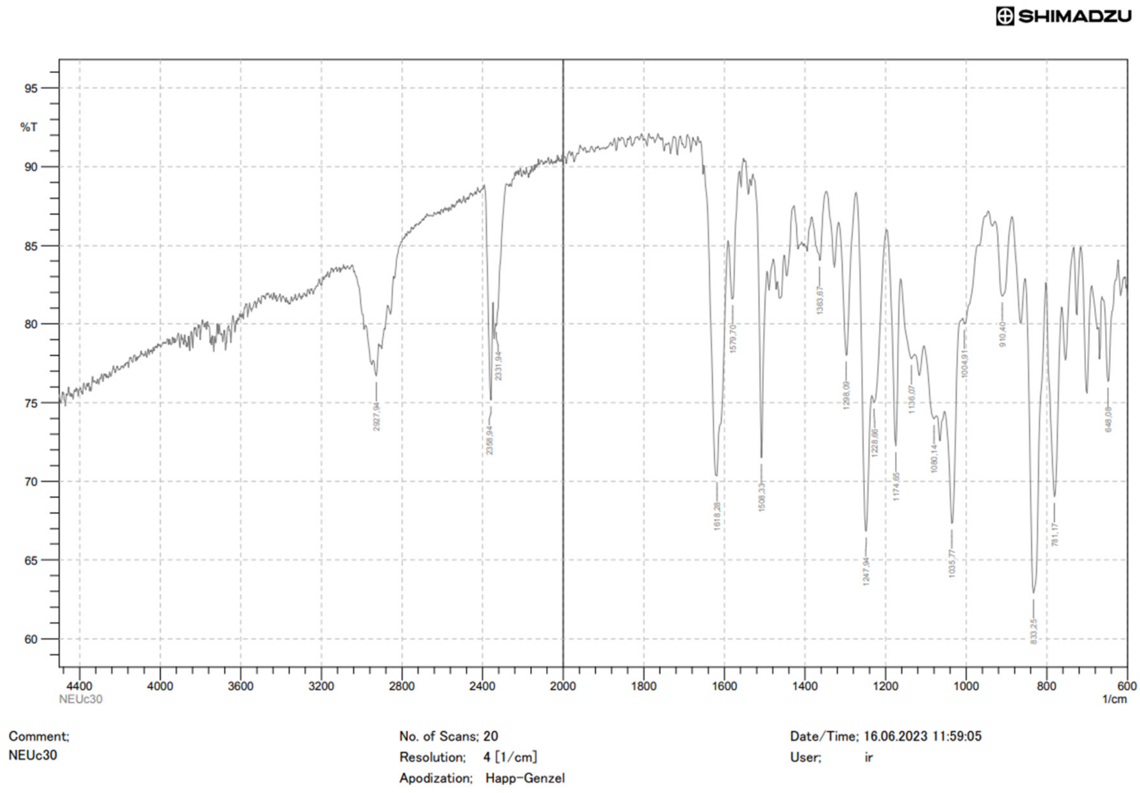

Spectrum 48. IR of compound **09**.

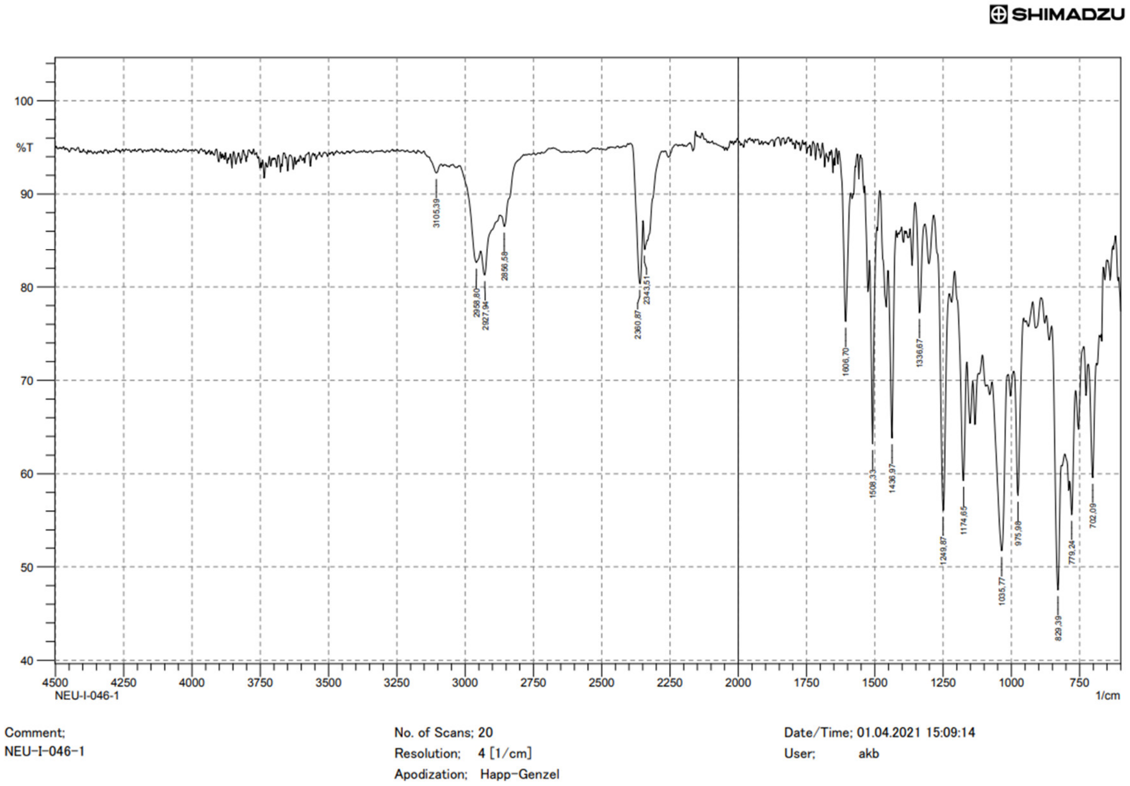

Spectrum 49. IR of compound **10**.

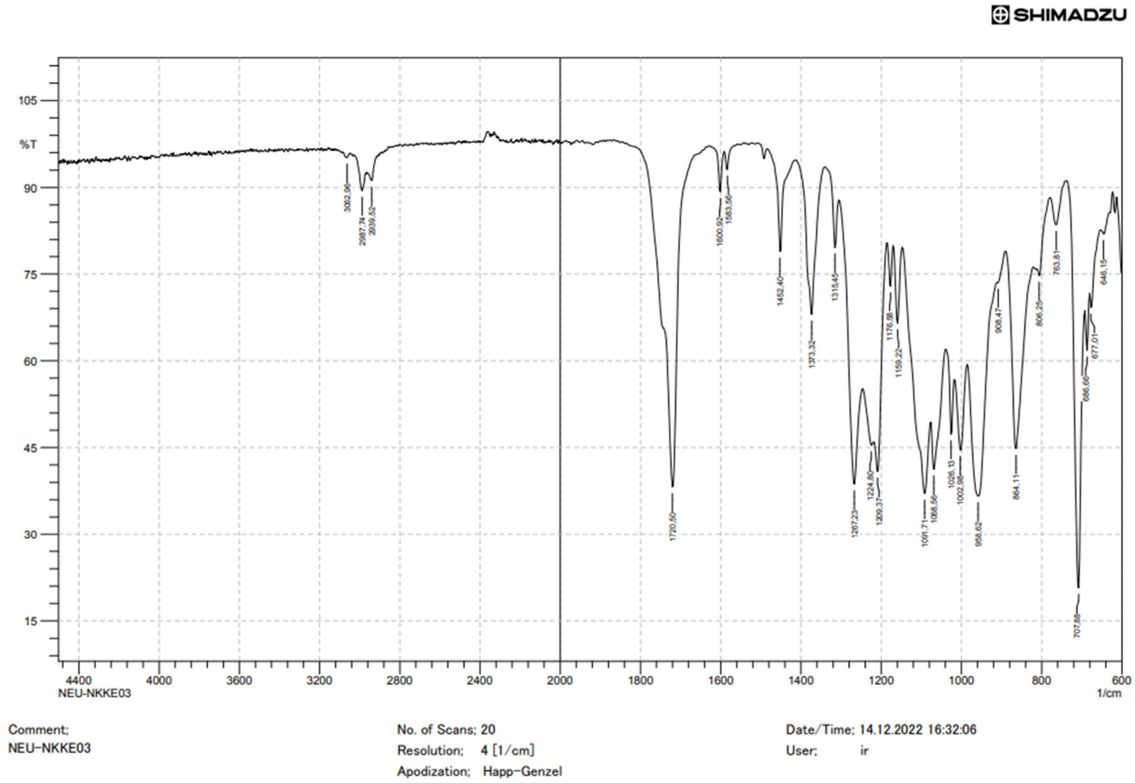

Spectrum 50. IR of compound **12**.

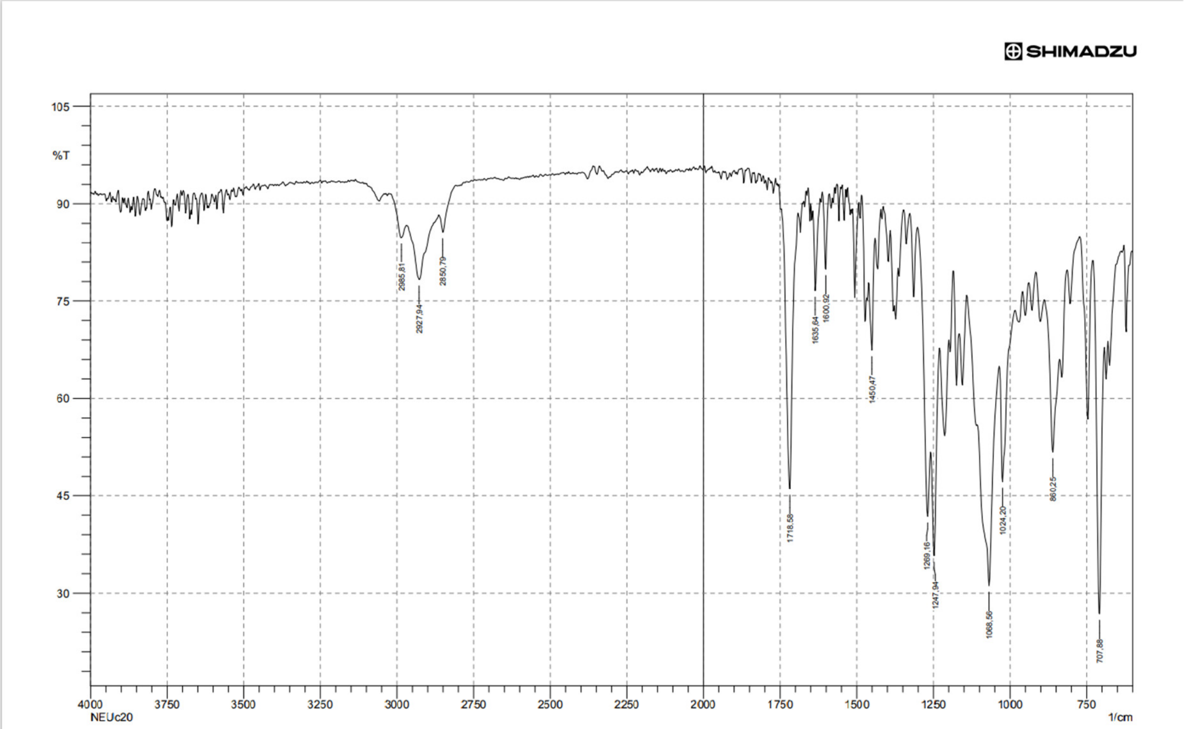

Spectrum 51. IR of compound **13**.

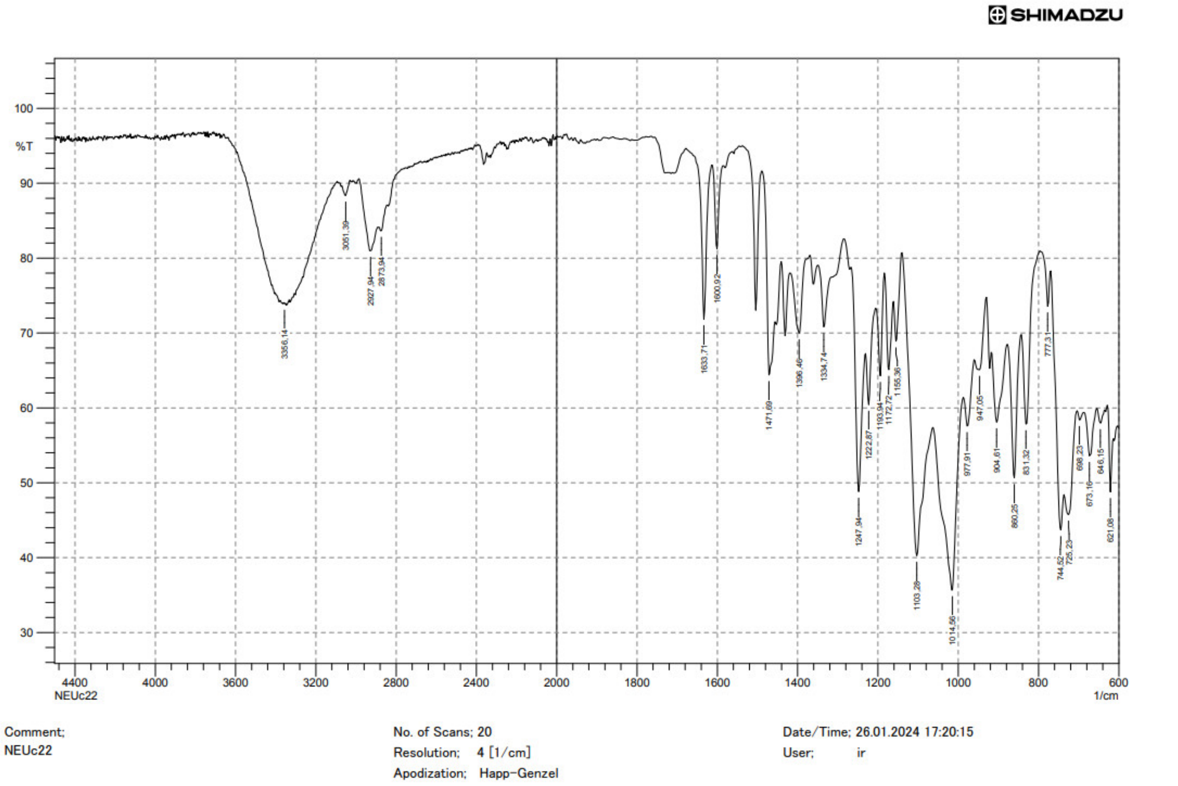

Spectrum 52. IR of compound **14**.

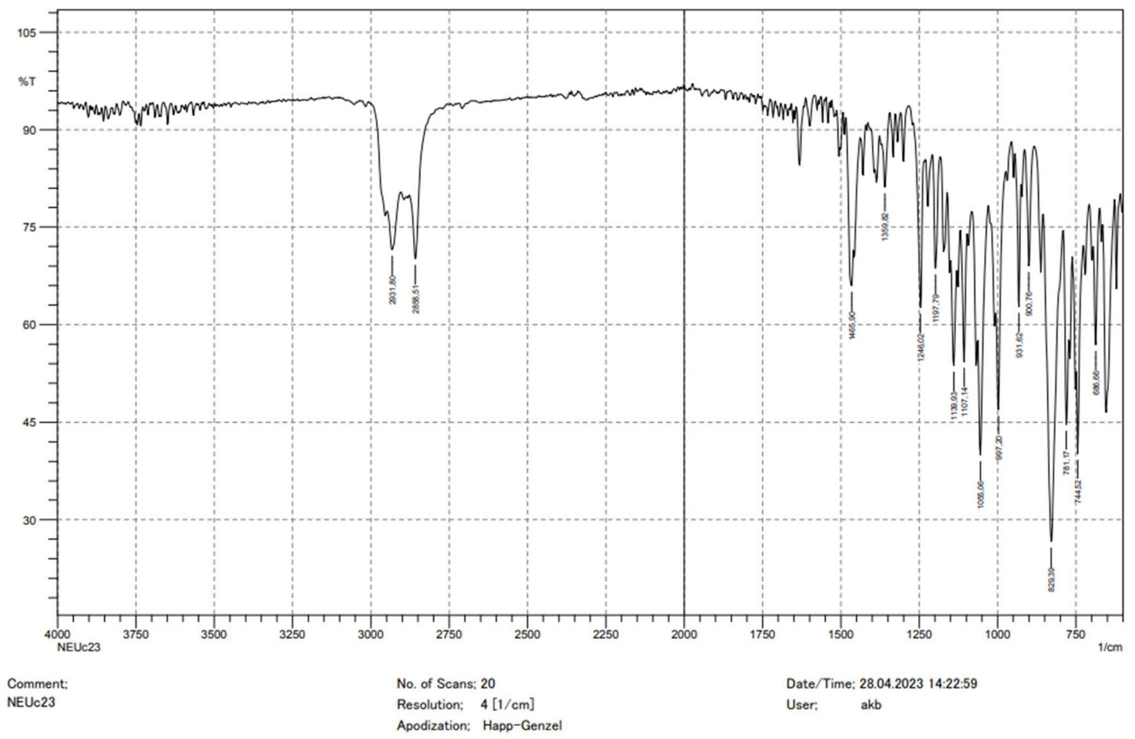

Spectrum 53. IR of compound **15**.

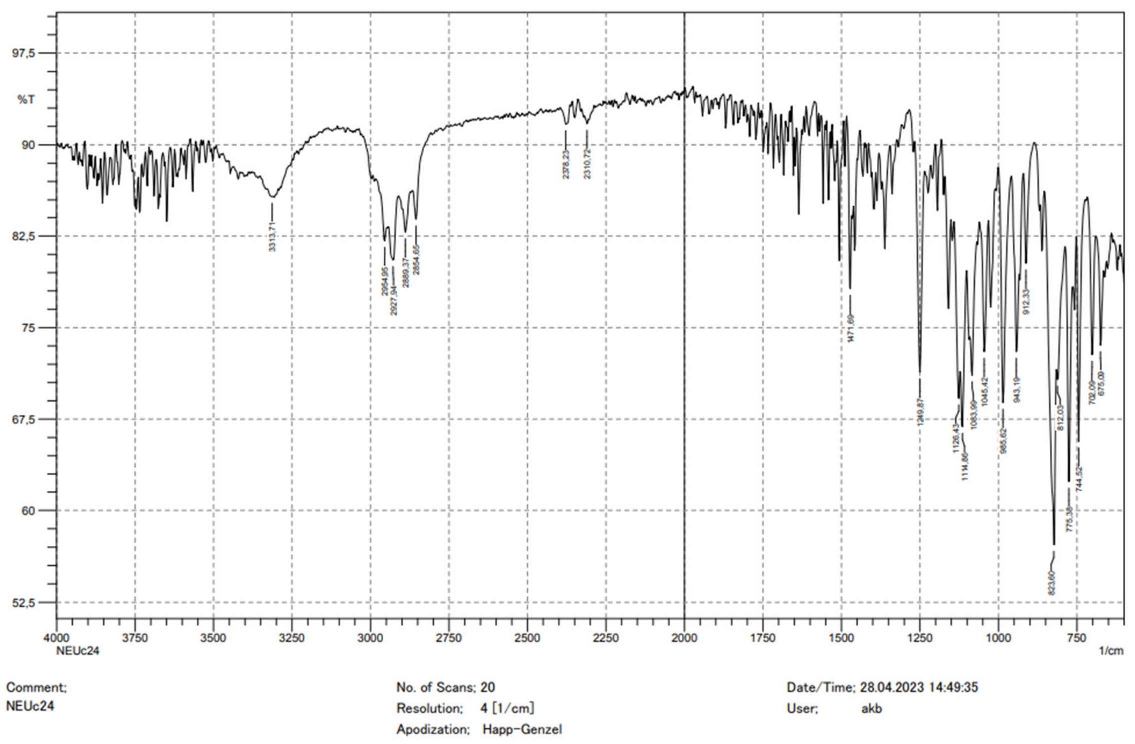

Spectrum 54. IR of compound **16**.

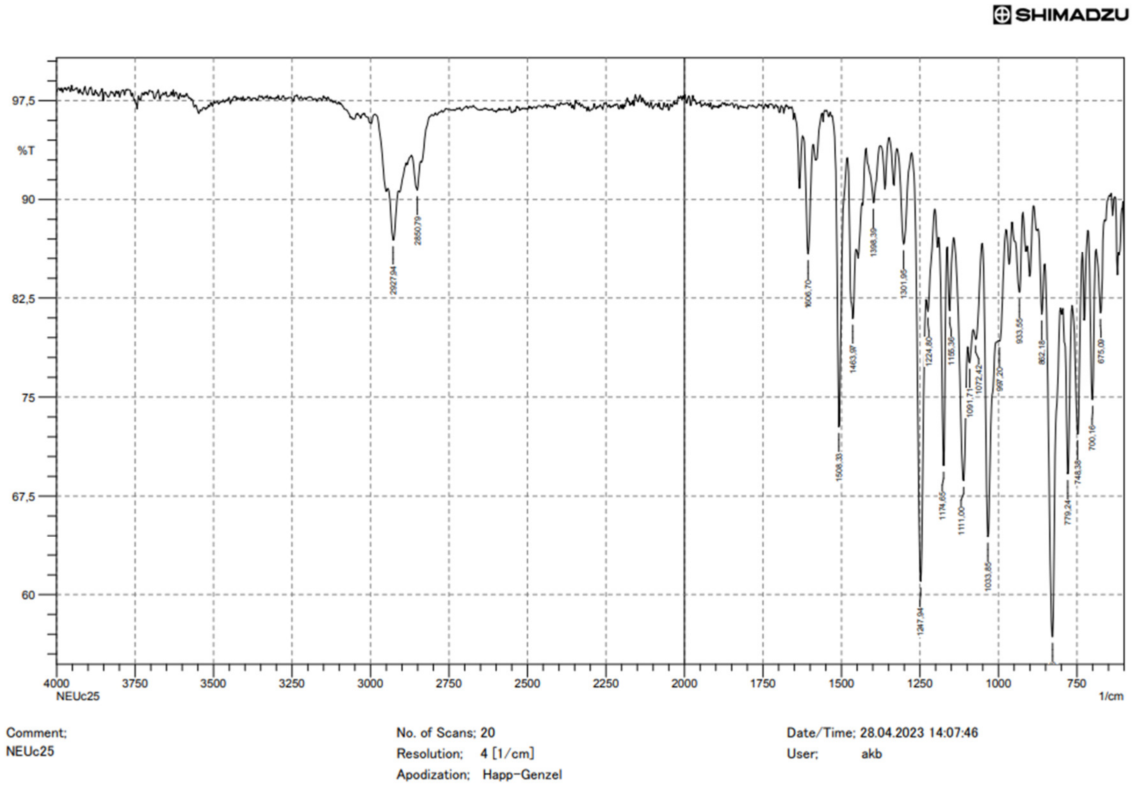

Spectrum 55. IR of compund **17**.

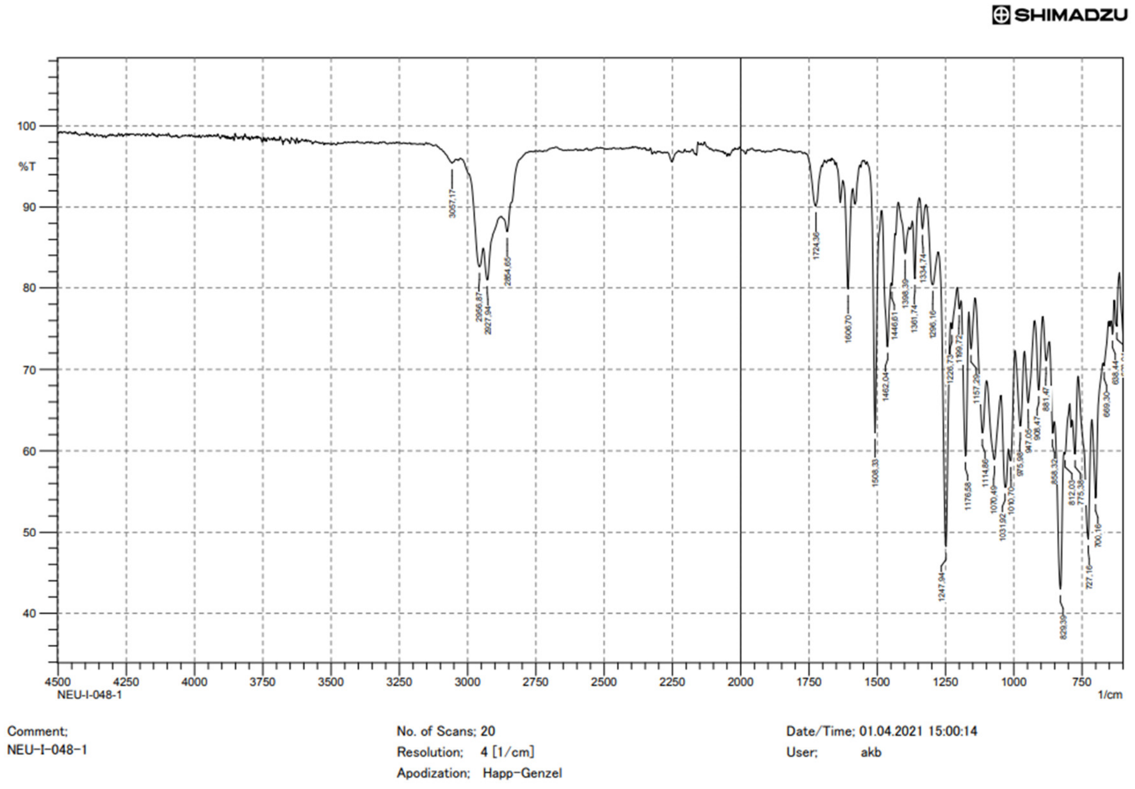

Spectrum 56. IR of compound **18**.

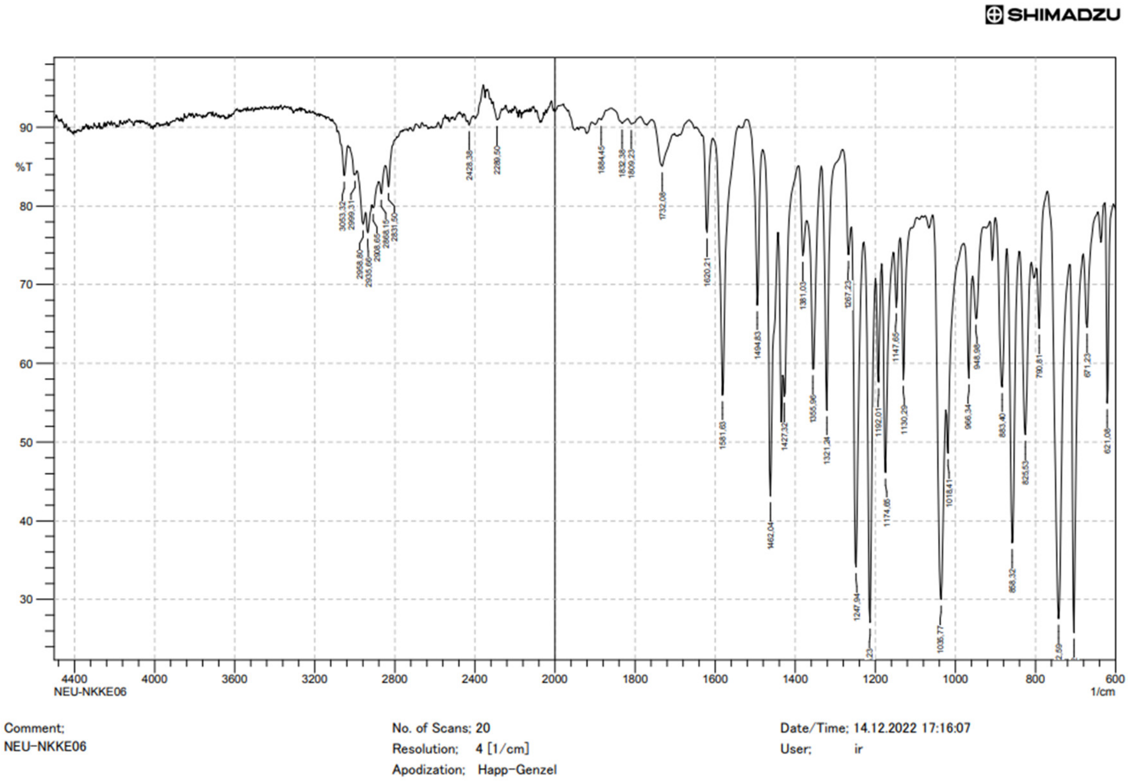

Spectrum 57. IR of compound **19**.

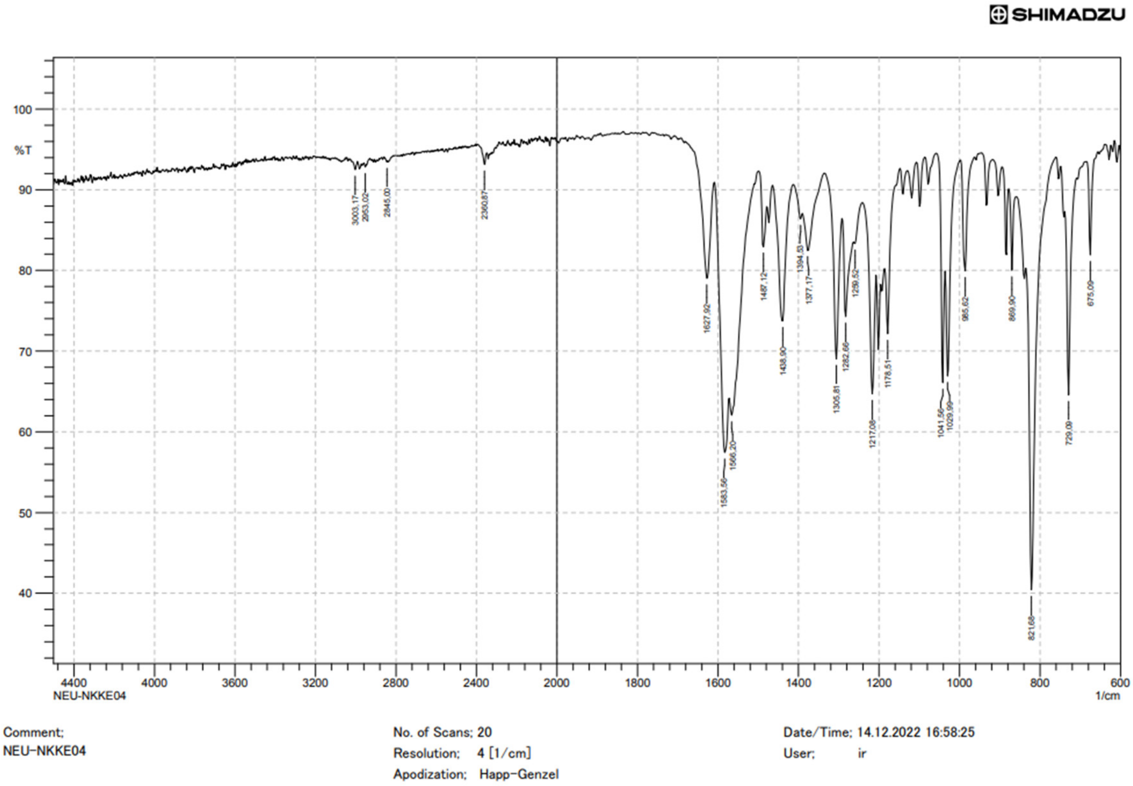

Spectrum 58. IR of compound **21**.

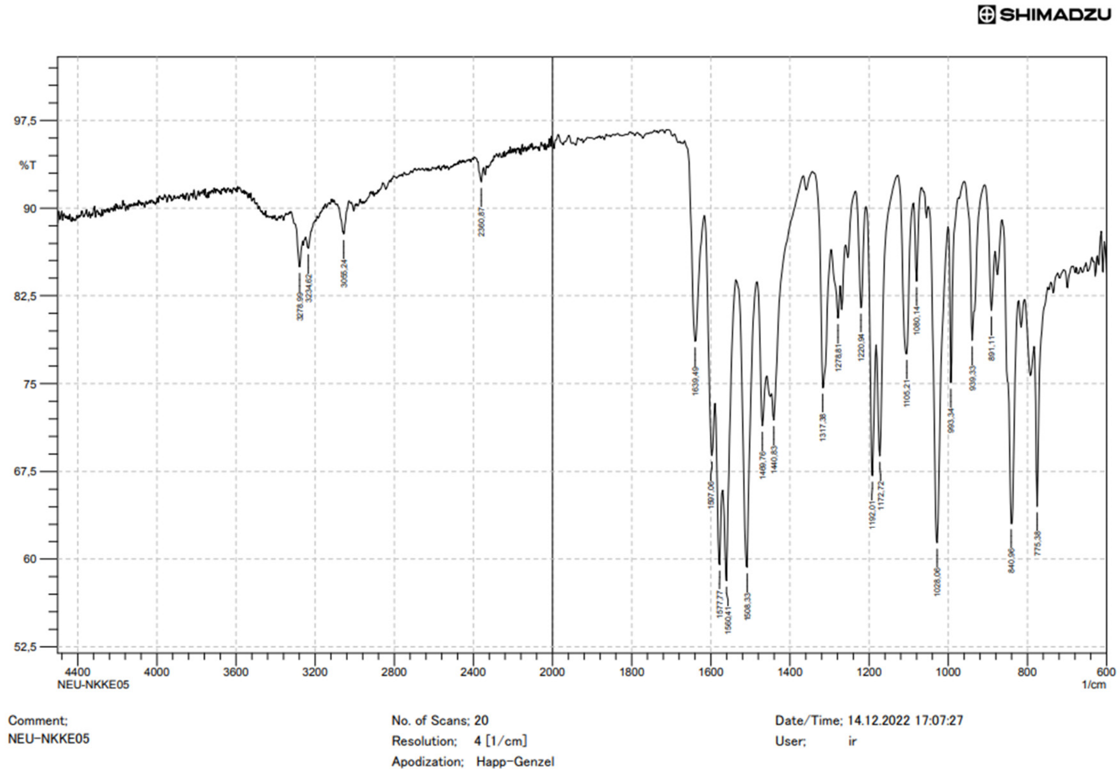

Spectrum 59. IR of compound **22**.

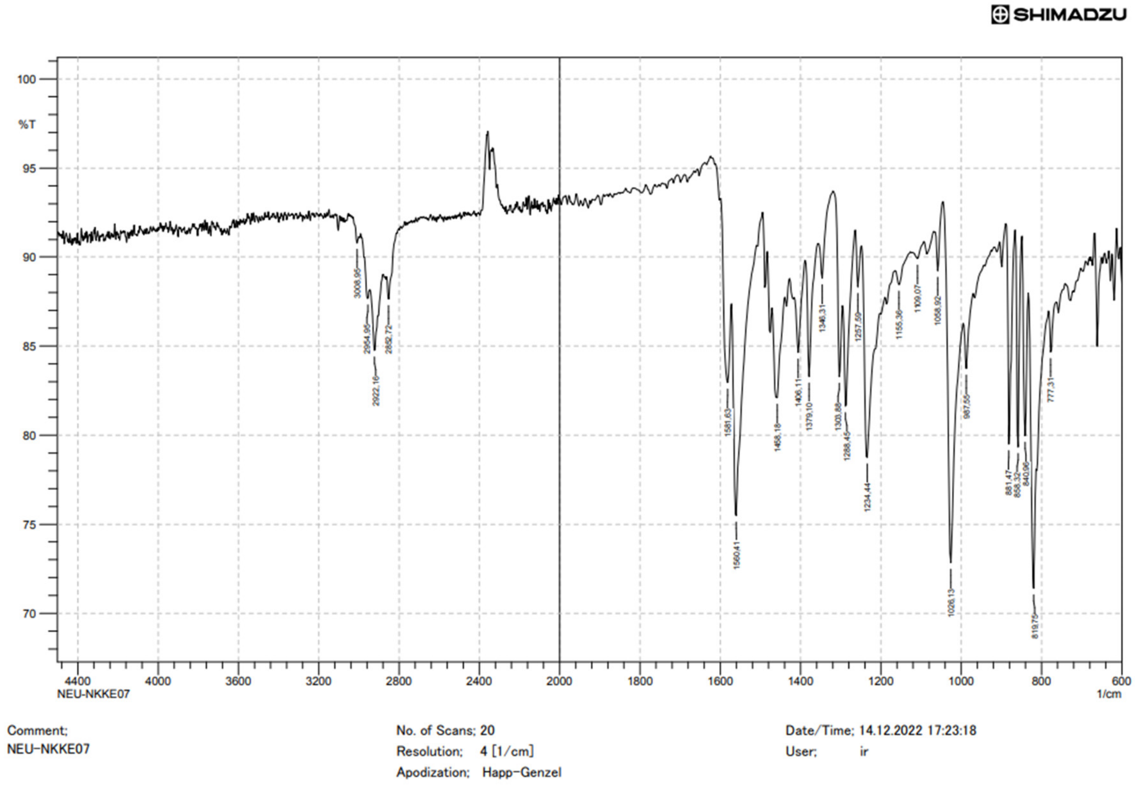

Spectrum 60. IR of compound **23**.

10. References

1. L. Li, M. Degardin, T. Lavergne, D. A. Malyshev, K. Dhami, P. Ordoukhanian and F. E. Romesberg, Natural-like replication of an unnatural base pair for the expansion of the genetic alphabet and biotechnology applications, *J Am Chem Soc*, 2014, **136**, 826-829.
2. F. Eggert and S. Kath-Schorr, A cyclopropene-modified nucleotide for site-specific RNA labeling using genetic alphabet expansion transcription, *Chem Commun (Camb)*, 2016, **52**, 7284-7287.
3. F. Eggert, K. Kurscheidt, E. Hoffmann and S. Kath-Schorr, Towards Reverse Transcription with an Expanded Genetic Alphabet, *Chembiochem*, 2019, **20**, 1642-1645.
4. Y. J. Seo, S. Matsuda and F. E. Romesberg, Transcription of an expanded genetic alphabet, *J Am Chem Soc*, 2009, **131**, 5046-5047.
5. C. Schulte, A. Solda, S. Spanig, N. Adams, I. Bekic, W. Streicher, D. Heider, R. Strasser and H. M. Maric, Multivalent binding kinetics resolved by fluorescence proximity sensing, *Commun Biol*, 2022, **5**, 1070.
6. R. A. Higuera-Rodriguez, M. C. De Pascali, M. Aziz, M. Sattler, U. Rant and W. Kaiser, Kinetic FRET Assay to Measure Binding-Induced Conformational Changes of Nucleic Acids, *ACS Sens*, 2023, **8**, 4597-4606.
7. U. Rant, K. Arinaga, S. Fujita, N. Yokoyama, G. Abstreiter and M. Tornow, Structural properties of oligonucleotide monolayers on gold surfaces probed by fluorescence investigations, *Langmuir*, 2004, **20**, 10086-10092.
8. W. Kaiser and U. Rant, Conformations of end-tethered DNA molecules on gold surfaces: influences of applied electric potential, electrolyte screening, and temperature, *J Am Chem Soc*, 2010, **132**, 7935-7945.
9. M. T. Walsh, E. E. Roller, K. S. Ko and X. Huang, Measurement of DNA Polymerase Incorporation Kinetics of Dye-Labeled Nucleotides Using Total Internal Reflection Fluorescence Microscopy, *Biochemistry*, 2015, **54**, 4019-4021.
10. D. A. Malyshev, Y. J. Seo, P. Ordoukhanian and F. E. Romesberg, PCR with an expanded genetic alphabet, *J Am Chem Soc*, 2009, **131**, 14620-14621.
11. R. Yamashige, M. Kimoto, Y. Takezawa, A. Sato, T. Mitsui, S. Yokoyama and I. Hirao, Highly specific unnatural base pair systems as a third base pair for PCR amplification, *Nucleic Acids Res*, 2012, **40**, 2793–2806.
12. I. Okamoto, Y. Miyatake, M. Kimoto and I. Hirao, High Fidelity, Efficiency and Functionalization of Ds-Px Unnatural Base Pairs in PCR Amplification for a Genetic Alphabet Expansion System, *ACS Synth Biol*, 2016, **5**, 1220-1230.
13. R. L. Strack, M. D. Disney and S. R. Jaffrey, A superfolding Spinach2 reveals the dynamic nature of trinucleotide repeat-containing RNA, *Nat Methods*, 2013, **10**, 1219-1224.
14. S. S. Agasti, Y. Wang, F. Schueder, A. Sukumar, R. Jungmann and P. Yin, DNA-barcoded labeling probes for highly multiplexed Exchange-PAINT imaging, *Chem Sci*, 2017, **8**, 3080-3091.
15. Y. Li, Z. Wang, L. Li, X. Tian, F. Shao and C. Li, Chemoselective and Diastereoselective Synthesis of C-Aryl Nucleoside Analogues by Nickel-Catalyzed Cross-Coupling of Furanosyl Acetates with Aryl Iodides, *Angew Chem Int Ed Engl*, 2022, **61**, e202110391.
16. C.-P. Lin, P. Florio, E. M. Campi, C. Zhang, D. P. Fredericks, K. Saito, W. R. Jackson and M. T. Hearn, Synthesis of substituted terpyridine ligands for use in protein purification, *Tetrahedron*, 2014, **70**, 8520-8531.

17. R. J. Karadeema, S. E. Morris, L. L. Lairson and R. Krishnamurthy, Towards an Understanding of the Molecular Mechanisms of Variable Unnatural Base-Pair Behavior: A Biophysical Analysis of dNaM-dTPT3, *Chem-Eur J*, 2021, **27**, 13991-13997.
18. J. S. New, W. L. Christopher, J. P. Yevich, R. Butler, R. F. Schlemmer, C. P. VanderMaelen and J. A. Cipollina, The thieno3,2-cpyridine and furo3,2-cpyridine rings: new pharmacophores with potential antipsychotic activity, *Journal of medicinal chemistry*, 1989, **32**, 1147-1156.
19. C. Domnick, F. Eggert and S. Kath-Schorr, Site-specific enzymatic introduction of a norbornene modified unnatural base into RNA and application in post-transcriptional labeling, *Chem Commun (Camb)*, 2015, **51**, 8253-8256.
20. B. Y. Michel and P. Strazewski, Synthesis of (−)-neplanocin A with the highest overall yield via an efficient Mitsunobu coupling, *Tetrahedron*, 2007, **63**, 9836-9841.
21. K. Inoue, Y. Feng, A. Mori and K. Okano, "Snapshot" Trapping of Multiple Transient Azolyllithiums in Batch, *Chemistry*, 2021, **27**, 10214.
22. R. R. Kadiyala, D. Tilly, E. Nagaradja, T. Roisnel, V. E. Matulis, O. A. Ivashkevich, Y. S. Halauko, F. Chevallier, P. C. Gros and F. Mongin, Computed CH acidity of biaryl compounds and their deprotonative metalation by using a mixed lithium/zinc-TMP base, *Chem Eur J*, 2013, **19**, 7944-7960.
